# Transfer Hydrogenation in Open-Shell Nucleotides — A Theoretical Survey

**DOI:** 10.3390/molecules191221489

**Published:** 2014-12-22

**Authors:** Florian Achrainer, Hendrik Zipse

**Affiliations:** Department of Chemistry, Ludwig-Maximilians-Universität Munich, Butenandtstr. 5–13, Munich 81377, Germany

**Keywords:** transfer hydrogenation, open-shell nucleotides, thermochemistry, heats of hydrogenation, radical stabilization energy

## Abstract

The potential of a larger number of sugar models to act as dihydrogen donors in transfer hydrogenation reactions has been quantified through the calculation of hydrogenation energies of the respective oxidized products. Comparison of the calculated energies to hydrogenation energies of nucleobases shows that many sugar fragment radicals can reduce pyrimidine bases such as uracil in a strongly exothermic fashion. The most potent reducing agent is the C3' ribosyl radical. The energetics of intramolecular transfer hydrogenation processes has also been calculated for a number of uridinyl radicals. The largest driving force for such a process is found for the uridin-C3'-yl radical, whose rearrangement to the C2'-oxidized derivative carrying a dihydrouracil is predicted to be exothermic by 61.1 kJ/mol in the gas phase.

## 1. Introduction

**Scheme 1:**
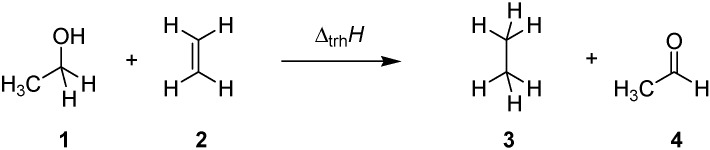
Transfer hydrogenation between ethanol (**1**) and ethylene (**2**).

Transfer hydrogenation between alcohols and alkenes represents a synthetically and technically important process for the hydrogenation of alkenes [1]. Over the past years numerous variants ranging from transition metal catalysis [2], to metal-free routes [3], or organocatalytic approaches [4] have been developed to be compatible with sensitive starting materials or to induce high enantioselectivities. The driving force for this type of process derives from the systematically higher heats of hydrogenation (∆_hyd_*H*) for alkenes as compared to structurally related aldehydes and ketones. Taking the reaction of ethanol (**1**) and ethylene (**2**) to ethane (**3**) and acetaldehyde (**4**) (Scheme 1) as an example, the driving force amounts to ∆_trh_*H*(1) = −67.8 kJ/mol when using experimentally measured heats of formation [5], and to −68.4 kJ/mol using theoretical calculations at G3(MP2)-RAD level [6].

**Figure 1:**
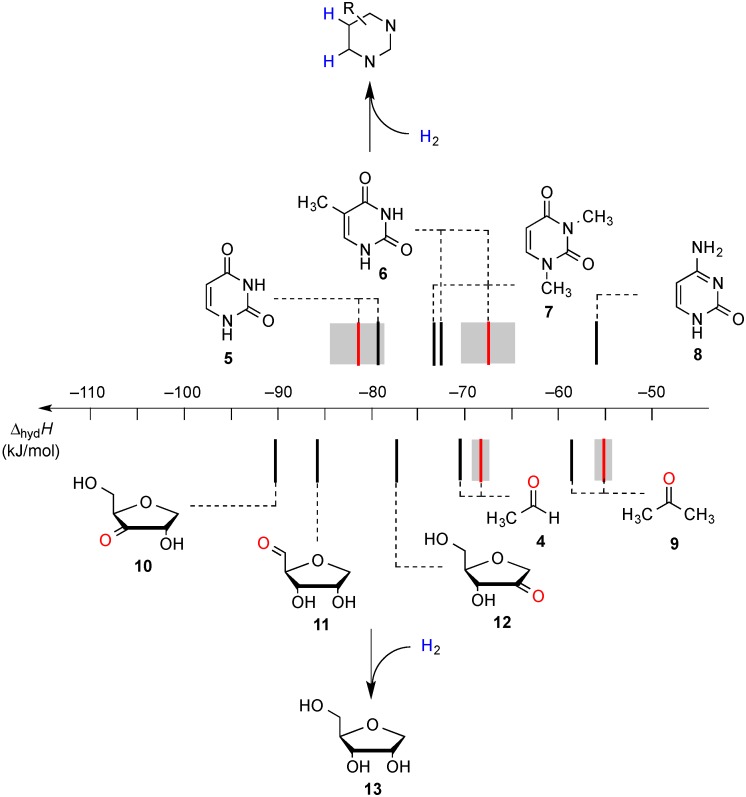
Gas phase heats of hydrogenation ∆_hyd_*H* at 298.15 K (G3(MP2)-RAD, in kJ/mol) of selected pyrimidine bases and carbonyl compounds. Experimental hydrogenation enthalpies are shown as red lines together with their standard deviation as grey bars.

Recent results obtained in a combined theoretical and experimental study to determine the heats of hydrogenation of the pyrimidine and purine bases indicate that the hydrogenation enthalpies of ketones and aldehydes derived from sugar models are, in part, closely similar to those of the pyrimidine bases [7]. The hydrogenation enthalpies are shown in Figure 1 such that a side-by-side comparison of all possible hydrogen transfer reactions is possible in a graphical way. From this representation it is apparent that uracil (**5**) as the most easily reduced nucleotide base with ∆_hyd_*H*(**5**) = −81.5 kJ/mol can react exothermically with sugar models such as 1'-anhydroribose **13** in a formal transfer hydrogen reaction to form ketone **12**.

**Scheme 2:**
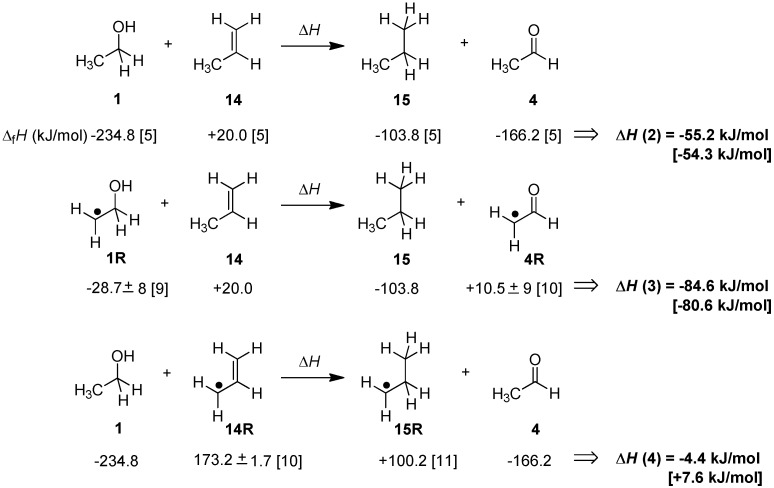
Experimentally determined transfer hydrogenation enthalpies ∆*H* for selected open- and closed-shell systems at 298.15 K in the gas phase (G3(MP2)-RAD values in brackets).

How will these energetics change on introducing a radical center in direct neighborhood to the reacting π-systems in the hydrogen-donor or -acceptor? This can in principle be discussed with reference to the reaction of propene (**14**) as the alkene receiving a hydrogen equivalent from either ethanol (**1**) or ethanol-2-yl radical **1R** (Scheme 2). While the former reaction involving closed-shell reactants and products is exothermic by −55.2 kJ/mol, the latter is significantly more exothermic by −84.6 kJ/mol. This increase in thermochemical driving force of 29.4 kJ/mol for dihydrogen transfer implies that ethanol-2-yl radical **1R** is a significantly better dihydrogen donor than its closed shell parent ethanol. On closer inspection of the reactant and product radicals involved it also becomes evident that the increased driving force is exactly identical to the difference in radical stabilization energies (RSE) of the reactant and product radicals ethanol-2-yl radical **1R** and acetaldehyde-2-yl radical **4R** [8]. In contrast, installation of a radical center in the alkene reaction partner as in allyl radical **14R** leads to a substantial reduction of the driving force for transfer hydrogenation with ethanol (**1**) to only −4.4 kJ/mol. This change can again be rationalized with reference to the RSE values, the stability of the reactant allyl radical **14R** now being much larger than that of the product radical **15R**. Taken together the data collected in Scheme 2 indicate that the energetics of the dihydrogen transfer processes involving open shell reactants are intimately connected to the stabilities of the radicals involved. As indicated in Scheme 2 this is also supported by calculations at G3(MP2)-RAD level, even though we note that the theoretically predicted reaction energies are somewhat smaller than those obtained from experimental data for the system selected here.

The radical-induced changes in dihydrogen transfer energetics are likely to impact the chemistry of oligonucleotide radicals in such way that radicals located at the sugar phosphate backbone become much better dihydrogen donors than their closed-shell parents. This may be particularly relevant in cases where oxidations of (oligo)nucleotide radicals have been observed under otherwise reducing conditions. One such case concerns the outcome of substrate reactions of the E441Q mutant of *E. coli* class I ribonucleotide reductase (RNR) [12,13,14,15].

**Scheme 3:**
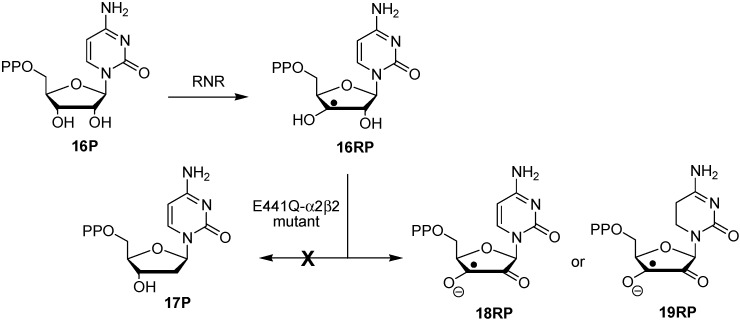
Substrate reaction of wild type class I RNR and its E441Q mutant.

Wild type class I RNR is known to convert cytidine diphosphate (**16P**) to the respective C2'-desoxynucleotide building block **17P** through a complex reaction sequence involving initial formation of C3' radical **16RP** (Scheme 3) [16,17,18]. The *E. coli* E441Q mutant does not yield any of the reduced product **17P**, but provides, in a characteristic time-dependent manner, signals of new open shell intermediates not observed in the wild type system. Using a combination of high-field EPR and ENDOR measurements and computational predictions of EPR parameters, one of these intermediates has been identified as semidione radical anion **18RP**. How this oxidized intermediate can be formed is not immediately obvious considering the reductive conditions present in the experiment [19]. The hydrogenation enthalpies for pyrimidine bases collected in Figure 1 together with the radical-induced increase in transfer hydrogenation energetics described in Scheme 2 now indicate that the cytosine base present in radical **16RP** can potentially act as an internal redox partner to the adjacent C3' ribosyl radical, thus generating product radical **19RP** (rather than **18RP**) through a transfer hydrogenation process. In order to explore the energetics of such a redox process, we have now studied the stabilities of reactant and product nucleoside radicals with the most relevant variations in the nucleobases and the location of the sugar radical center. Comparison is also made to the same transfer hydrogen processes in the respective closed-shell parent systems.

## 2. Results and Discussion

### 2.1. Closed-Shell Systems

Reaction energies for the transfer hydrogenation between the sugar phosphate backbone and the nucleobases as described in Scheme 3 for the example of cytosine are currently not available, due to the lack of thermochemical data. As a first step the hydrogenation enthalpies of the individual redox components were analyzed. These include the individual pyrimidine and purine bases present in DNA and RNA and for the sake of convenience simple alkenes and carbonyls from Scheme 2. The required enthalpies have been obtained through combination of single point energies calculated at the (RO)MP2(FC)/6-311+G(3df,2p) level of theory in combination with B3LYP/6-31G(d) optimized structures and thermochemical corrections to 298.15 K using the rigid rotor/harmonic oscillator model. This level of theory has recently been used to assess the stability of a wide variety of radicals and non-radicals [20,21,22]. Improved energies were obtained using the already mentioned G3(MP2)-RAD composite model [6] with experimental data for well-known compounds such as ethylene (**2**) and are summarized in Table 1.

**Table 1:** Calculated and experimentally determined heats of hydrogenation ∆_hyd_*H* at 298.15 K in the gas phase for pyrimidine bases and sugar models shown in Figure 1 (in kJ/mol).

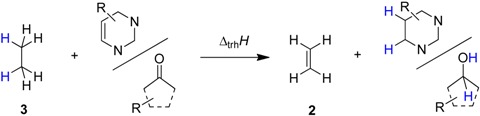						
Reactants	MP2(FC)/ 6-311 + G(3df,2p)		G3(MP2)-RAD		Exp.	
	∆_trh_*H* ^a^	∆_hyd_*H* ^b^	∆_trh_*H* ^a^	∆_hyd_*H* ^b^	∆_trh_*H* ^a^	∆_hyd_*H* ^b^
Ethylene (**2**)	0.0	−136.3	0.0	−136.3	0.0 ^c^	−136.3 ± 0.2 [23]
Propene (**14**)	+12.4	−123.9	+10.8	−125.5	+12.6 ^c^	−125.0 ± 0.2 [24]
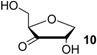	+44.5	−91.8	+44.9	−91.4	n/a	n/a
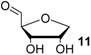	+50.3	−86.0	+50.2	−86.1	n/a	n/a
Uracil (**5**)	+56.1	−80.2	+57.0	−79.3	+53.6 ± 2.1 [7]	−82.7 ± 2.1 [7]
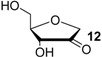	+58.3	−78.0	+58.8	−77.5	n/a	n/a
1,3-Dimethyluracil (**7**)	+62.4	−73.9	+62.7	−73.6	+68.5 ± 2.1 [7]	−67.8 ± 2.1 [7]
Thymine (**6**)	+64.2	−72.1	+63.8	−72.5	+68.8 ± 4.2 [7]	−67.5 ± 2.3 [7]
Acetaldehyde (**4**)	+65.8	−70.5	+65.1	−71.2	+67.8 ^c^	−69.1 ± 0.4 [25]
Acetone (**9**)	+77.8	−58.5	+76.5	−59.8	+80.9 ^c^	−55.6 ± 0.4 [26]
Cytosine (**8**)	+82.4	−53.9	+80.2	−56.1	n/a	n/a
Adenine (**25**)	+152.6	+16.3	+139.0	+2.7	n/a	n/a
Guanine (**26**)	+155.2	+18.9	+141.2	+4.9	n/a	n/a

^a^ Defined as ∆_trh_*H* = ∆_f_*H*( C_2_H_4_) + ∆_f_*H* (R_2_CH-OH) − ∆_f_*H* (R_2_C=O) − ∆_f_*H* (C_2_H_6_) and ∆_trh_*H* = ∆_f_*H*( C_2_H_4_) + ∆_f_*H* (RCH_2_-CH_2_R) − ∆_f_*H* (RHC=CHR) − ∆_f_*H* (C_2_H_6_), respectively; ^b^ Addition of the reaction enthalpies ∆_trh_*H* to the experimentally determined hydrogenation enthalpy of ethylene ∆_hyd_*H* (**2**) = −136.3 ± 0.2 kJ/mol [23] yields the hydrogenation enthalpy ∆_hyd_*H* of the respective double bond; ^c^ Using the following heats of formation: ∆_f_*H*^0^ (C_2_H_6_, **3**) = −84.0 kJ/mol; ∆_f_*H^0^* (C_2_H_4_, **2**) = +52.4 kJ/mol; ∆_f_*H*^0^ (CH_3_CH=CH_2_, **14**) = +20.0 kJ/mol; ∆_f_*H*^0^ (C_3_H_8_, **15**) = −103.8 kJ/mol; ∆_f_*H*^0^ (C_2_H_5_OH, **1**) = −234.8 kJ/mol; ∆_f_*H*^0^ (CH_3_CHO, **4**) = −166.2 kJ/mol; ∆_f_*H*^0^ ((CH_3_)_2_CHOH, **20**) = −272.6 kJ/mol; ∆_f_*H*^0^ ((CH_3_)_2_C=O, **9**) = −217.1 kJ/mol from ref. [5].

We note at this point that hydrogenation energies ∆_hyd_*H* obtained through combination of theoretically calculated reaction energies ∆_trh_*H* for the transfer hydrogenation process (5) with the experimentally measured hydrogenation enthalpy of ethylene ∆_hyd_*H*(**2**) = −136.3 ± 0.2 kJ/mol [23] are significantly more accurate than hydrogenation energies ∆_hyd_*H* calculated for the direct reaction of H_2_ with the respective alkenes [7].

**Figure 2:**
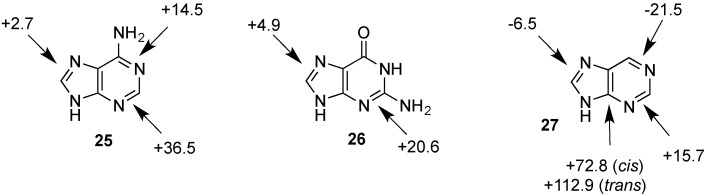
Hydrogenation enthalpies ∆_hyd_*H* of adenine (**25**), guanine (**26**) and their parent lead structure purine (**27**) at G3(MP2)-RAD level of theory according to Equation (5) (gas phase, 298.15 K, in kJ/mol).

As is readily seen in Table 1 and Figure 1, the most easily reduced base is uracil (**5**) with ∆_hyd_*H* = −79.3 kJ/mol at G3(MP2)-RAD level of theory. Introduction of two methyl groups present in *N*,*N'*-dimethyluracil (**7**) leads to ∆_hyd_*H* = −73.6 kJ/mol, which is almost the same result as obtained for thymine (**6**, ∆_hyd_*H* = −72.5 kJ/mol) where a methyl group is attached to C5 position. The reduction of the hydrogenation enthalpy through addition of a methyl substituent to the reacting double bond of around +7 kJ/mol (∆_hyd_*H*(**5**/**6**)) is also observed in other systems such as ethylene/propene (∆_hyd_*H*(exp., **2**/**14**) = +11.3 kJ/mol), cyclohexene [27]/1-methylcyclohexene [28] (∆_hyd_*H*(exp., **21**/**22**) = +7.4 kJ/mol) and cyclopentene [27]/1-methylcyclopentene [28] (∆_hyd_*H*(exp., **23**/**24** = +11.3 kJ/mol) and can therefore be considered as a general phenomenon. The most difficult pyrimidine base to reduce is cytosine (**8**) with ∆_hyd_*H* = −56.1 kJ/mol due to the different substitution pattern. Hydrogenation of the purine bases adenine (**25**) and guanine (**26**) is significantly more difficult, a result of the intrinsically large differences in reductions of C-C and C-N double bonds [5,29] (Figure 2). For instance the hydrogenation enthalpies for the canonical structures of adenine and guanine are all endothermic with energetically best values of ∆_hyd_*H* (**25**) = +2.7 kJ/mol and ∆_hyd_*H* (**26**) = +4.9 kJ/mol, respectively.

The reduction of the oxidized sugar models **10**, **11** and **12** (Figure 1, Table 1) to give 1’-anhydroribose (**13**) are all located in a range from −77.5 to −91.4 kJ/mol at G3(MP2)-RAD level. As a result, transfer hydrogenation to yield dihydrouracil (**5**) and 2'-oxo sugar **12** is predicted to be slightly exothermic by −79.3 − (−77.5) = −1.8 kJ/mol. The other two hydroxyl substituents in sugar model **13** yielding the C3' oxidized product **10** or the C5' oxidized product **11** are, in contrast, not effective enough as dihydrogen donors to reduce uracil in an exothermic fashion.

### 2.2. Open-Shell Systems

For a variety of small C-centered radicals heats of hydrogenation ∆_hyd_*H* obtained at ROMP2 and G3(MP2)-RAD level of theory have been collected in Table 2 together with experimentally available values. Hydrogenation energies have again been calculated relative to ethylene/ethane as the reference systems. For all systems considered here the hydrogenation energies are smaller for the radicals as compared to the respective closed shell systems: for the allyl radical **14R** already mentioned in the introduction the heat of hydrogenation amounts to ∆_hyd_*H*(**14R**) = −63.7 kJ/mol, while that of its closed-shell analog propene amounts to ∆_hyd_*H*(**14**) = −125.5 kJ/mol (G3(MP2)-RAD values). The difference of 61.8 kJ/mol reflects the difference in radical stabilization energy of the allyl radical with RSE(**14R**) = −72.0 kJ/mol relative to that of the 1-propyl radical with RSE(**33R**) = −12.2 kJ/mol (Table 3).

**Table 2:** Validation of theoretical methods with experimentally available data for the open-shell induced transfer hydrogenation ∆_trh_*H* at 298.15 K in the gas phase (in kJ/mol).

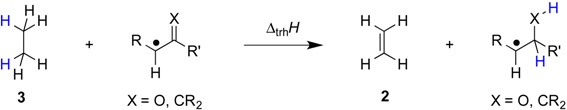							
Initial radicals	UB3LYP/ 6-31G(d)		ROMP2(FC)/ 6-311+G(3df,2p)		G3(MP2)-RAD		Exp. ^c^
	∆_trh_*H* ^a^	∆_hyd_*H* ^b^	∆_trh_*H* ^a^	∆_hyd_*H* ^b^	∆_trh_*H* ^a^	∆_hyd_*H* ^b^	∆_trh_*H* ^a^
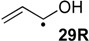	+79.5	−56.8	+62.5	−73.8	+64.9	−71.4	+55.4 ± 17.3
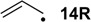	+82.8	−53.5	+71.5	−64.8	+72.7	−63.7	+65.4 ± 5.9
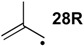	+88.4	−47.9	+72.9	−63.4	+72.8	−63.5	+64.6 ± 2.2
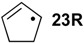	+88.3	−48.0	+79.9	−56.5	+75.9	−60.4	+82.7 ± 7.6
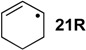	+96.3	−40.0	+85.8	−50.5	+81.6	−54.7	+74.9 ± 4.9
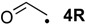	+137.3	+1.0	+90.1	−46.2	+91.4	−44.9	+94.9 ± 17.1
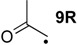	+149.0	+12.7	+98.3	−38.0	+99.3	−37.0	+74.1 ± 8.1
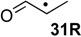	+154.6	+18.3	+104.8	−31.5	+104.6	−31.7	+91.9 ± 9.3
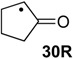	+161.6	+25.3	+105.7	−30.6	+106.6	−29.7	+106.6 ± 13.5

^a^ Defined as ∆_trh_*H* = ∆_f_*H*( C_2_H_4_) + ∆_f_*H* (•R_2_CH-OH) − ∆_f_*H* (•R_2_C=O) − ∆_f_*H* (C_2_H_6_) and ∆_trh_*H* = ∆_f_*H*( C_2_H_4_) + ∆_f_*H* (RCH_2_-CH_2_R) − ∆_f_*H* (RHC=CHR) − ∆_f_*H* (C_2_H_6_), respectively; ^b^ Addition of the reaction enthalpies ∆_trh_*H* to the experimentally determined hydrogenation enthalpy of ethylene ∆_hyd_*H* (C_2_H_4_, **2**) = −136.3 ± 0.2 kJ/mol [23] yields the hydrogenation enthalpy ∆_hyd_*H* of the respective double bond; ^c^ See Supporting Information for full validation and ∆_f_*H*^0^ of the respective radicals.

**Table 3:** Radical stabilization energies (RSE) obtained at G3(MP2)-RAD level of theory (in kJ/mol).

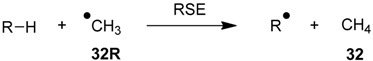							
	RSE ^a^	Exp. ^b^		RSE ^a^	Exp. ^b^	∆RSE ^c^	∆∆_trh_*H* ^d^
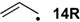	−72.0	−70.7	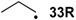	−12.2	−17.1	−59.8	−61.8
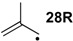	−66.1	−66.5	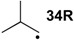	−10.6	−20.1	−55.5	−55.4
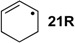	−85.4	−96.6	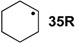	−20.7	−23.0	−64.7	−64.6
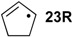	−84.7	−94.6	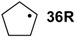	−33.4	−39.3	−51.3	−51.3
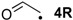	−36.7	−44.7	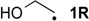	−10.3	−15.5	−26.4	−26.3
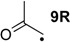	−32.4	−38.1	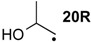	−8.4	−44.9	−24.0	−22.8

^a^: Defined as RSE = ∆*H* = ∆_f_*H* (CH_4_) + ∆_f_*H* (R•) − ∆_f_*H* (R-H) − ∆_f_*H* (•CH_3_); from ref. [8]; ^b^: Using following heats of formation:∆_f_*H*^0^ (•CH_3_, **32R**) = +146.7 kJ/mol [11] and ∆_f_*H*^0^ (CH_4_, **32**) = +74.6 kJ/mol [5]. ∆_f_*H*^0^ of radicals from ref. [11] ∆_f_*H*^0^ of closed-shell compounds from ref. [5]; ^c^ Defined as ∆RSE = RSE (RHC•-CR=X) − RSE (RHC•-CHR-XH); ^d^ Defined as ∆∆_trh_*H* = ∆_trh_*H* (RH_2_C-CR=X) − ∆_trh_*H* (RHC•-CR=X).

The effects are somewhat smaller (in an absolute as well as relative sense) in the hydrogenation of C–O double bonds, a typical example being the hydrogenation of acetaldehyde radical **4R** as compared to its closed-shell analog acetaldehyde (**4**): while the former is exothermic by ∆_hyd_*H*(**4R**) = −44.9 kJ/mol, the latter amounts to ∆_hyd_*H*(**4**) = −71.2 kJ/mol (Table 2). The “radical” effect as the difference in RSE values of reactant radical **4R** and product radical **1R** amounts to only 26.4 kJ/mol in this case (Table 3). Even smaller hydrogenation energies are found for radicals carrying one carbonyl and one alkyl substituent as is the case for 2-oxocyclopentan-1-yl radical **30R**. The small hydrogenation energy of ∆_hyd_*H*(**30R**) = −29.7 kJ/mol may be understood as a consequence of the stability of substrate radical **30R** arising from the combined action of a strong (carbonyl) acceptor with a weak (alkyl) donor substituent.

**Scheme 4:**
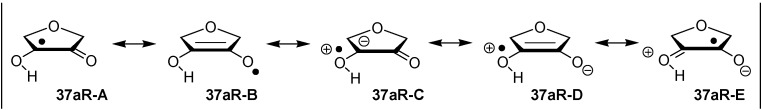
Resonance stabilization of donor/acceptor substituted radical **37aR**.

Hydrogenation energies for pentose-derived aldehydes, ketones and alkenes are collected in Table 4 together with results for smaller model systems based on the tetrahydrofuran ring system. The smallest hydrogenation energies are found for donor/acceptor substituted radicals such as **37aR**, **12aR**, and **10bR** combining a hydroxy-group donor with a carbonyl acceptor substituent. Hydrogenation energies are quite similar for all three systems, which implies that the hydroxymethyl substituent present in **12aR** (with ∆_hyd_*H*(**12aR**) = −14.4 kJ/mol), but not in radical **37aR** (with ∆_hyd_*H*(**37aR**) = −11.6 kJ/mol) is only of minor relevance. This conclusion is also supported by the almost negligible difference in hydrogenation energies of radicals **12aR** and **10bR**, in which the substituent and radical positions are interchanged (Table 4). Systematically larger hydrogenation energies are calculated for ribose model radicals carrying a carbonyl acceptor and the ring oxygen atom as alkoxy donor substituent, a typical example being **12bR** with ∆_hyd_*H*(**12bR**) = −33.1 kJ/mol. This group also includes radicals **40R** and **11R**, in which the oxidized C5' position acts as acceptor substituent to the radical center. Given the almost identical hydrogenation energies for these two systems (∆_hyd_*H*(**40R**) = −26.7 kJ/mol *vs.* (∆_hyd_*H*(**11R**) = −27.5 kJ/mol) the influence of the C2' hydroxy substituent present in **11R**, but not in **40R**, appears to be negligible. The small hydrogenation energies for all push/pull-substituted radicals described above reflect the efficient interaction of the alkoxy/hydroxy-donor and carbonyl-acceptor substituents [8,30]. As shown in Scheme 4 for the example of radical **37aR**, these can be rationalized with the admixture of charge-transfer configurations such as **37aR-D** and **37aR-E** to the canonical Lewis structures **37aR-A** and **37aR-B**. The relevance of the charge-transfer configurations **37aR-D** and **37aR-E** also imply that the carbonyl oxygen atom may be a better hydrogen-bond acceptor at the radical stage as compared to the closed-shell parent. Similarly, the hydroxy-substituent present in **37aR** may be a better hydrogen-bond donor as compared to closed shell analogs (and also significantly more acidic) [17,31,32,33]. This may, in part, also be responsible for the somewhat smaller hydrogenation energies in radicals carrying α-hydroxy- as compared to α-alkoxy substituents.

**Table 4:** Calculated Boltzmann-averaged heats of hydrogenation <∆_hyd_*H*> at 298.15 K in the gas phase for a variety of sugar radicals shown in Figure 3 (in kJ/mol). Only the reactant radicals are shown.

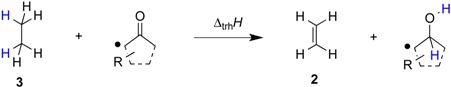				
	ROMP2(FC)/ 6-311+G(3df,2p)		G3(MP2)-RAD	
	<∆_trh_*H*> ^a^	<∆_hyd_*H*> ^b^	<∆_trh_*H*> ^a^	<∆_hyd_*H*> ^b^
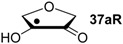	+192.0	−7.28	+124.7	−11.6
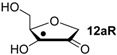	+128.2	−8.09	+121.9	−14.4
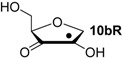	+125.4	−11.0	+121.3	−15.0
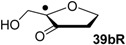	+118.8	−17.5	+114.8	−21.5
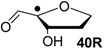	+115.7	−20.7	+109.6	−26.7
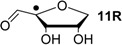	+115.5	−20.8	+108.8	−27.5
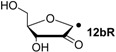	+105.3	−31.0	+103.2	−33.1
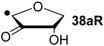	+104.6	−31.7	+101.4	−34.9
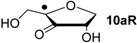	+104.0	−32.3	+100.1	−36.2
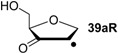	+93.3	−43.0	+94.1	−42.3
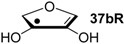	+76.5	−59.8	+73.8	−62.5
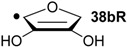	+65.9	−70.4	+65.2	−71.1
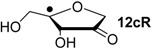	+39.5	−96.8	+41.8	−94.5
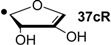	+13.8	−122.5	+18.1	−118.2

^a^ Defined as ∆_trh_*H* = ∆_f_*H*( C_2_H_4_) + ∆_f_*H* (•R_2_CH-OH) − ∆_f_*H* (•R_2_C=O) − ∆_f_*H* (C_2_H_6_); ^b^ Addition of the reaction enthalpies ∆_trh_*H* to the experimentally determined hydrogenation enthalpy of ethylene ∆_hyd_*H* (C_2_H_4_) = −136.3 ± 0.2 kJ/mol [23] yields the hydrogenation enthalpy ∆_hyd_*H* of the respective double bond.

Hydrogenation energies for sugar models containing an allyl radical such as **37bR** and **38bR** are systematically larger as compared to those of the respective tautomeric form. This may be exemplified with radical **37bR**, whose hydrogenation energy of ∆_hyd_*H*(**37bR**) = −62.5 kJ/mol is 50.9 kJ/mol larger than that of α-keto radical **37aR**. The hydrogenation product obtained is identical for both species and the energy difference of 50.9 kJ/mol thus corresponds to the energy difference between the enol and keto forms of radical **37a/bR**. Finally, the largest hydrogenation energies are calculated for π-systems not coupled in a resonant fashion to the radical center as is the case in radical **12cR** and **37cR** (Table 4).

**Scheme 5:**
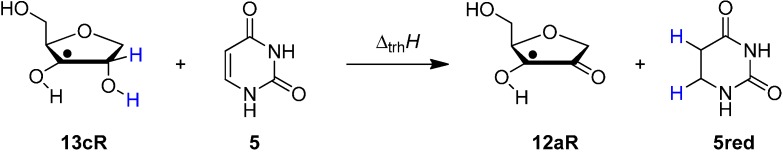
Transfer hydrogenation between ribose model radical **13cR** and uracil (**5**).

**Figure 3:**
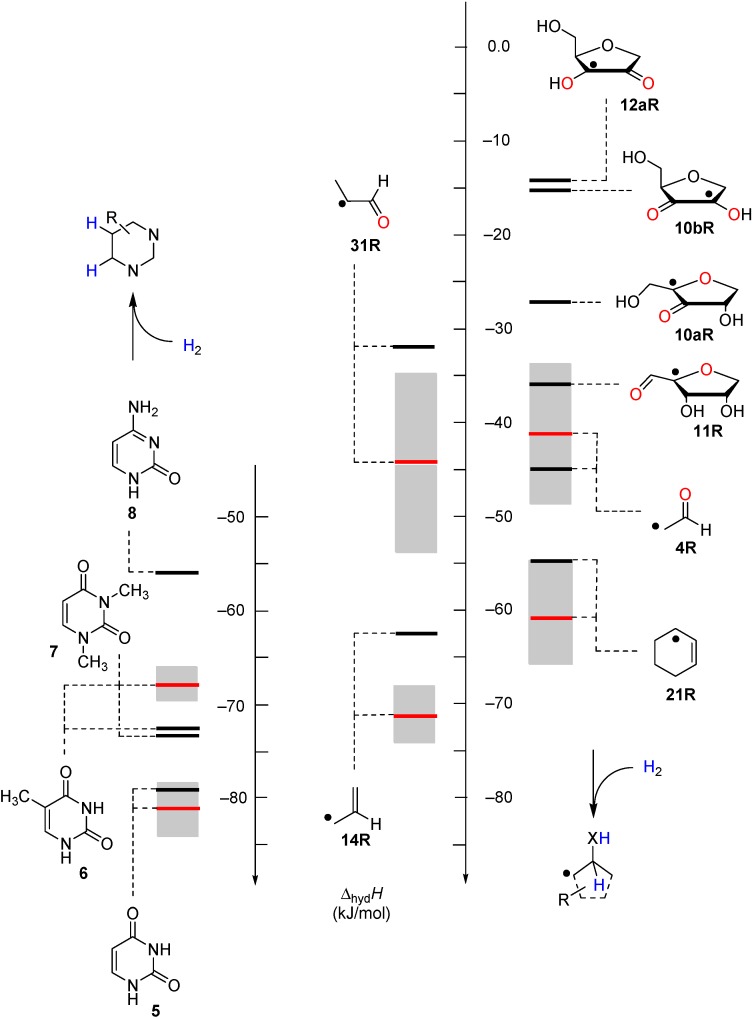
Hydrogenation enthalpies ∆_hyd_*H* at 298.15 K (G3(MP2)-RAD, in kJ/mol) of some selected open-shell systems (right side) in comparison to pyrimidine bases. Experimental enthalpies are shown as red lines together with their standard deviations as grey bars.

Reaction energies for dihydrogen transfer between ribose model radicals and the nucleotide bases can be calculated from the hydrogenation energies in Table 1 and Table 4 in a straightforward manner. For the reduction of uracil (**5**) with C3' ribosyl radical model **13cR** as an example (Scheme 5), the reaction enthalpy ∆_trh_*H*(9) is identical to the difference in hydrogenation energies for uracil (**5**) and oxidized sugar radical **12aR**, that is, ∆_trh_*H*(9) = −79.3 − (−14.4) = −64.9 kJ/mol. In pictorial terms, this difference equates to the vertical distance on the hydrogenation enthalpy scale shown in Figure 3. Closer inspection of this scale also shows that C3' ribosyl radical model **13cR** is sufficiently potent to reduce all three pyrimidine bases (as well as their N-methylated derivatives) in an exothermic manner.

**Figure 4:**
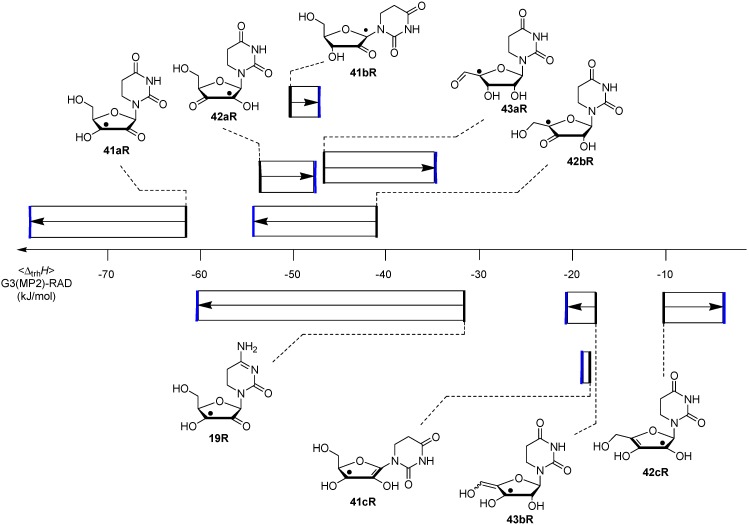
Boltzmann-averaged transfer hydrogenation enthalpy scale <∆_trh_*H>* for C-centered uridinyl and cytidinyl radicals (G3(MP2)-RAD, in kJ/mol, black bars gas phase, blue bars addition of ∆G_solv_ (IEF-PCM/UAHF/UHF/6-31G(d)) in water, only the product radicals are shown).

In order to assess the thermodynamics of such a process in complete nucleosides, intramolecular transfer hydrogenation reactions have been studied for different types of uridinyl radicals, where the unpaired spin is located at the C2', C3', or C4' position. The energies for intramolecular dihydrogen transfer between sugar and base fragments are depicted in Figure 4 in a pictorial manner such that the transfer hydrogenation product is shown with the C-C or C-O double bond in the ribose fragment indicating the origin of the dihydrogen unit. Energies have been calculated for the gas phase as well as the aqueous phase in order to identify the influence of a polar (hydrogen-bonding) medium on the reaction outcome. The two reaction energies are shown in Figure 4 through vertical lines connected by an arrow, where the base of the arrow corresponds to the gas phase and the tip of the arrow to the aqueous phase reaction energies.

**Figure 5:**
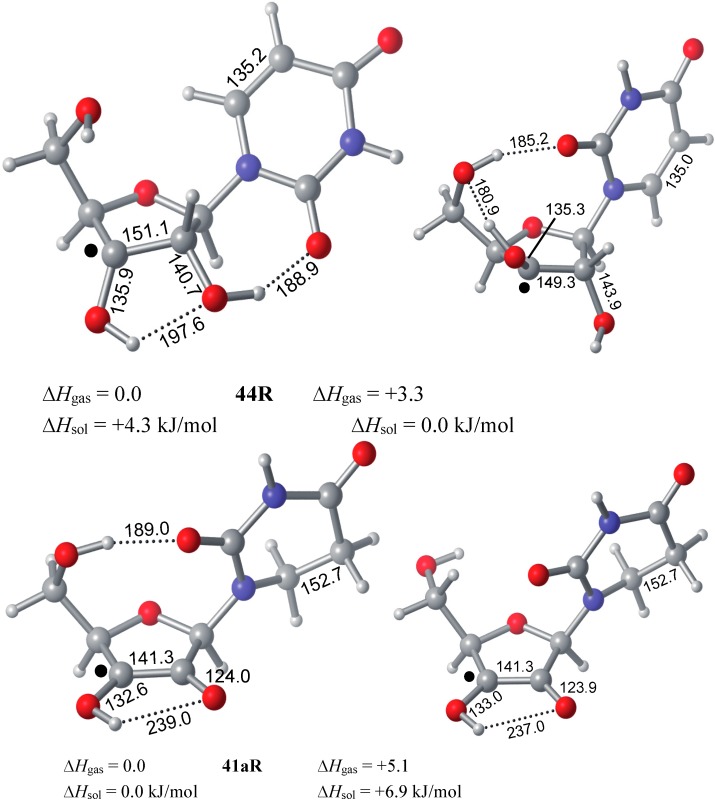
Graphical representation of the two most stable uridinyl radicals **44R** and **41aR** obtained at G3(MP2)-RAD level of theory in gas phase and with implicit solvation.

Comparison of the data of individual components in Figure 3 with those for the nucleosides in Figure 4 shows that sugar-to-base transfer hydrogenation becomes slightly more positive on covalent coupling both redox partners. The only exception is uridinyl radical **41bR** with ∆_trh_*H* (**41bR**) = −50.4 kJ/mol, where the hydrogenation energies of the respective fragments differ by ∆_hyd_*H* (**5**) − ∆_hyd_*H* (**12bR**) = −46.2 kJ/mol. This difference can be traced back to the presence of a second donor substituent in radical **41bR** not present in ribose model radical **12bR**. In more general terms, the most exothermic intramolecular transfer hydrogenation process is that of C3' uridinyl radical **44R** yielding the C2' oxidized product radical **41aR** with a reaction energy of ∆_trh_*H*(**41aR**) = −61.1 kJ/mol at G3(MP2)-RAD level in the gas phase. Transfer hydrogenation starting from the C2' radical to yield product radical **42aR** is somewhat less exothermic at ∆_trh_*H*(**42aR**) = −53.8 kJ/mol, closely followed by reaction of the uridin-C1'-yl radical to product radical **41bR** with ∆_trh_*H*(**41bR**) = −50.4 kJ/mol. Transfer hydrogenation reactions generating C-C (instead of C-O) double bonds in the ribose fragement are, in comparison, significantly less exothermic. Switching from uridine to cytidine leads to significantly smaller reaction energies, in line with the smaller hydrogenation energy of cytosine as compared to uracil (Table 1). The above results have been obtained from Boltzmann-averaged enthalpies for fully flexible nucleoside radicals and can potentially be modified through intermolecular interactions present in base-paired systems or polar solvents. In order to obtain an estimate for the magnitude of these effects, solvation energies in water were calculated using the continuum solvation model (IEF-PCM/UAHF/UHF/6-31G(d)//UB3LYP/6-31G(d)) and combined with the gas phase results obtained at G3(MP2)-RAD level. The resulting hydrogenation energies in polar solution show that uridinyl radical **41aR** (with an oxidized C2' position) and the respective cytidinyl radical **19R** benefit most from the solvation in that the reactions become more exothermic in polar solvents. The higher exothermicity results from the better solvation of the product radical due to the omission of the hydrogen bond upon oxidation of the C2' hydroxyl group (Figure 5).

**Scheme 6:**
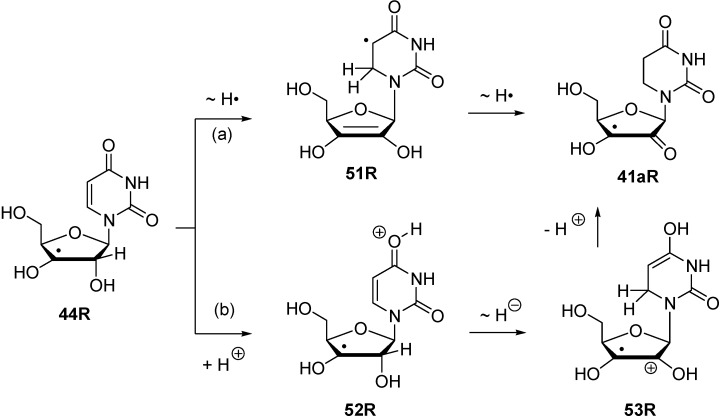
Possible pathways for stepwise transfer hydrogenation reactions using C3' radical **44R** as an example.

The reaction energies for inter- and intramolecular transferhydrogenation presented above permit no statement on the pathways along which such a process may occur. Using radical **44R** as an example, some speculation on possible pathways can nevertheless be made (Scheme 6).

While a concerted dihydrogen shift can most likely be ruled out in view of the relative orientation of the donor- and acceptor fragments in radical **44R**, two different reaction types for stepwise hydrogen transfer may be recognized: (a) Reactions involving open shell-intermediates at the nucleotide base. This may, for example, involve initial single hydrogen atom (or proton-coupled electron) transfer to the uracil C6 position. Radical **51R** formed in this process can then undergo a second hydrogen atom transfer to yield product radical **41aR**. That hydrogen atom transfer reactions between carbohydrate radicals and nucleotide bases can occur quite rapidly has recently been reported by Giese *et al*. in spectroscopic studies of C4' thymidine radicals, where the rearranged C5 thymyl radical could be detected as one of the main open-shell species by EPR spectroscopy [34]. (b) An alternative set of pathways exists in which the unpaired spin never leaves the ribose unit. This may, for example, involve initial protonation of the C4 carbonyl group in the uracil base, followed by hydride transfer between the ribose C2' and the uracil C6 positions. Deprotonation of the radical cation **53R** formed in such a step then leads, together with some tautomerization steps, to the rearranged radical **41aR** (Scheme 6). What both pathways have in common is the direct involvement of the uracil C6' position. As is also visible in the structures shown for **44R** in Figure 5, this is simply due to the spatial proximity of this center to the reacting C2' ribose carbon atom.

## 3. Experimental Section

Geometry optimizations of all systems have been performed at the (U)B3LYP/6-31G(d) level of theory. Thermochemical corrections to 298.15 K have been calculated at the same level of theory using the rigid rotor/harmonic oscillator model. A scaling factor of 0.9806 has been used for this latter part. Single point energies have then been calculated at the (RO)MP2(FC)/6-311+G(3df,2p) level. Combination of the (RO)MP2 total energies with thermochemical corrections obtained at B3LYP level yield the enthalpies termed as “ROMP2” in the text [20,21,22]. In conformationally flexible systems enthalpies and free energies have been calculated as Boltzmann-averaged values (w ≥ 1%) over all available conformers obtained by a conformational search using the MM3* force field implemented in *MacroModel 9.7* [35]. Improved relative energies have been obtained using the G3(MP2)-RAD scheme proposed by Radom *et al*. [6]. Solvation free energies have been calculated through single point calculations at the IEF-PCM/UAHF/UHF/6-31G(d) level [36,37]. The UCCSD(T) calculations required in the G3(MP2)-RAD compound scheme have been performed with *MOLPRO* [38] and all other calculations with *Gaussian 03*, Rev. D.01 [39].

## 4. Conclusions

The hydrogenation energies calculated for ribose model radicals fully support the strongly reductive nature of these species. This is particularly true for ribose model radicals whose oxidation generates captodatively stabilized product radicals. From all systems analyzed here the C3' ribosyl radical appears to be the most strongly reductive species. As revealed through comparison to hydrogenation energies for individual nucleotide bases and also seen in the reaction energies for intramolecular transfer hydrogenation in, for example, the uridin-C3'-yl radical, the strongly reductive nature of ribosyl radicals implies that pyrimidine bases can be reduced in an exothermic fashion. While these results clearly establish a significant driving force for the dihydrogen transfer processes, no statement can be made on the most preferred pathway along which such a process may proceed.

## Supplementary Materials

Supplementary materials can be accessed at: http://www.mdpi.com/1420-3049/19/12/21489/s1.
