# Supplementary material for: Transfer Hydrogenation in Open-Shell Nucleotides — A Theoretical Survey
Source: Molecules. 2014 Dec 22;19(12):21489–505. doi: 10.3390/molecules191221489 (PMC6271186; doi:10.3390/molecules191221489)
Supplement: Supplementary file 1 [file molecules-19-21489-s001.pdf]

Supporting Information

Contents

1. Theoretical Methods……………………………………………………………………S1
2. Energies and Enthalpies of Studied Compounds……………………………………….……S2
3. Energies and Enthalpies Related to Radical Stabilization Energies…………………………..S26
4. Experimental Thermochemical Data………………………………………….…………S28
5. Calculated Reaction Enthalpies…………………………………………………..…………S32
6. Influence of Solvation Methods…………………………………………………………S39
7. Structures of All Stationary Points……………………………………...…………S44
8. References…………………………………………………………………..…………..….S133

1. Theoretical Methods

Geometry optimizations of all systems have been performed at the (U)B3LYP/6-31G(d) level of theory. Thermochemical corrections to 298.15 K have been calculated at the same level of theory using the rigid rotor/harmonic oscillator model. A scaling factor of 0.9806 has been used for this latter part. Single point energies have then been calculated at the (RO)MP2(FC)/6-311+G(3df,2p) level. Combination of the MP2 total energies with thermochemical corrections obtained at B3LYP level yield the enthalpies termed as "ROMP2" in the text. This level of theory has recently been used to assess the stability of a wide variety of radicals and non-radicals [1–3]. In the conformationally flexible systems enthalpies and free energies have been calculated as Boltzmann-averaged values (w ≥ 1%) over all available conformers obtained by a preliminary conformational search using the MM3* force field implemented in *MacroModel 9.7* [4,5].

Improved relative energies are obtained using the G3(MP2)-RAD scheme proposed by *Radom et al.* [6,7]. These are based on the same geometries and thermal corrections as the MP2 level:

$$E_{tot}(\text{G3(MP2)-Rad}) = E(\text{(U)CCSD(T)/6-31G(d)//(U)B3LYP/6-31G(d)}) + DE(\text{G3MP2large}) + DE(\text{HLC})$$

$$DE(\text{G3MP2large}) = \text{(RO)MP2(FC)/G3MP2large//(U)B3LYP/6-31G(d)} - \text{(RO)MP2(FC)/6-31G(d)//(U)B3LYP/6-31G(d)}$$

$$DE(\text{HLC}) = -An(\text{beta}) - B(n(\text{alpha}) - n(\text{beta})) \text{ with } A = 9.413 \times 10^{-3} \text{ au}, B = 3.969 \times 10^{-3} \text{ au}.$$

n(alpha) = number of α valence electrons and n(beta) = number of β valence electrons

Additional consideration of solvation was included by calculating free energies of implicit H_2O using the polarizable continuum solvation model in its IEF-PCM [8], C-PCM [9] or SMD [10] variant. In case for the IEF- and C-PCM calculations the United Atom Hartree Fock (UAHF) radii in combination with UHF/6-31G(d) theory have been used [11].

$$H_{sol} = H_{298} + \Delta G_{solv}$$

(U)CCSD(T) calculations have been performed with *MOLPRO* [12], the geometry optimizations, frequencies and PCM calculations with *Gaussian03 Rev. D.01* [13]. The SMD model is implemented in *Gaussian09 Rev. C.01* [14].

2. Energies and Enthalpies of Studied Compounds

Table S1. Energies and enthalpies of studied compounds (closed- and open-shell) at various levels of theory in the gas phase (298.15 K, 1 atm, in Hartree).

	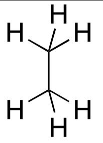 **(3)**		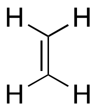 **(2)**		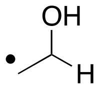 **(1R)**	
	E_{tot}	H_{298}	E_{tot}	H_{298}	E_{tot}	H_{298}
UB3LYP	−79.834175	−79.752210	−78.5874583	−78.533222	−154.3613832	−154.291870
ROMP2	−79.6200629	−79.5380979	−78.3932565	−78.3390202	−154.066633	−153.9971198
G3(MP2)−RAD	−79.7268957	−79.6449307	−78.4841764	−78.4299401	−154.1927918	−154.1232786

	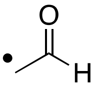 **(4R)**		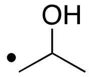 **(20R)**		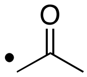 **(9R)**	
	E_{tot}	H_{298}	E_{tot}	H_{298}	E_{tot}	H_{298}
UB3LYP	−153.1715366	−153.125159	−193.6803856	−193.581855	−192.4950213	−192.419618
ROMP2	−152.8787261	−152.8323485	−193.2916261	−193.1930955	−192.1068777	−192.0314744
G3(MP2)−RAD	−152.989464	−152.9430864	−193.4603518	−193.3618212	−192.2600568	−192.1846535

	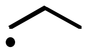 **(33R)**		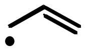 **(14R)**		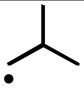 **(34R)**	
	E_{tot}	H_{298}	E_{tot}	H_{298}	E_{tot}	H_{298}
UB3LYP	−118.4711107	−118.377919	−117.2603540	−117.190479	−157.7856471	−157.663659
ROMP2	−118.1690627	−118.0758710	−116.9739045	−116.9040295	−157.3910101	−157.2690220
G3(MP2)−RAD	−118.3090897	−118.2158980	−117.0984521	−117.0285771	−157.5736006	−157.4516125

	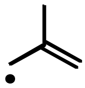 **(28R)**		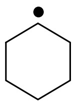 **(35R)**		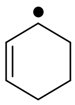 **(21R)**	
	E_{tot}	H_{298}	E_{tot}	H_{298}	E_{tot}	H_{298}
UB3LYP	−156.5772554	−156.478343	−235.2139163	−235.053530	−234.0081729	−233.871204
ROMP2	−156.1966271	−156.0977147	−234.6443077	−234.4839214	−233.4544966	−233.3175277
G3(MP2)−RAD	−156.3632755	−156.2643631	−234.8956477	−234.7352614	−233.6883126	−233.5513437

	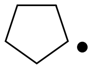 **(36R)**		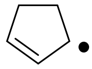 **(23R)**		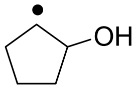 **(45R)**	
	E_{tot}	H_{298}	E_{tot}	H_{298}	E_{tot}	H_{298}
UB3LYP	−195.8948865	−195.764645	−194.6860804	−194.579304	−271.1017498	−270.966094
ROMP2	−195.4190525	−195.2888110	−194.2269215	−194.1201451	−270.5398569	−270.4042011
G3(MP2)-RAD	−195.6277641	−195.4975226	−194.4182277	−194.3114513	−270−7772463	−270.6415905

Table S2. Energies and enthalpies of some selected radicals at various levels of theory in the gas phase (298.15 K, 1 atm, in Hartree).

	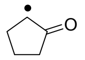<br>**(30R)**		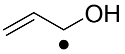<br>**(29R)**		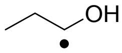<br>**(46aR)**	
	E_{tot}	H_{298}	E_{tot}	H_{298}	E_{tot}	H_{298}
UB3LYP	−269.9214771	−269.808652	−192.4759940	−192.400134	−193.6887929<br>−193.6887181	−193.588901<br>−193.588740<br><H><br>−193.5888273
ROMP2	−269.3582119	−269.2453868	−192.0973633	−192.0215033	−193.2966386<br>−193.2968177	−193.1967467<br>−193.1968396<br><H><br>−193.1967924
G3(MP2)−RAD	−269.5800170	−269.4671919	−192.2511757	−192.1753157	−193.4654625<br>−193.4655549	−193.3655706<br>−193.3655768<br><H><br>−193.3655780
	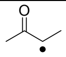<br>**(47R)**		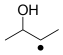<br>**(48R)**		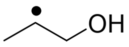<br>**(46bR)**	
	E_{tot}	H_{298}	E_{tot}	H_{298}	E_{tot}	H_{298}
UB3LYP	−231.8198849<br>−231.8171823	−231.714775<br>−231.712050<br><H><br>−231.7146372	−232.9988018<br>−232.9985058	−232.870922<br>−232.870630<br><H><br>−232.8707976	−193.6793493<br>−193.6795750	−193.580597<br>−193.580636<br><H><br>−193.5806200
ROMP2	−231.3339692<br>−231.3316005	−231.2288593<br>−231.2264682<br><H><br>−231.2286779	−232.5132100<br>−232.5129169	−232.3853302<br>−232.3850411<br><H><br>−232.3852139	−193.2877564<br>−193.2880859	−193.1890041<br>−193.1891469<br><H><br>−193.1890758
G3(MP2)−RAD	−231.5298190<br>−231.5276468	−231.4247091<br>−231.4225145<br><H><br>−231.4245051	−232.7251136<br>−232.7247112	−232.5972338<br>−232.5968354<br><H><br>−232.5970736	−193.4569905<br>−193.4572485	−193.3582382<br>−193.3583095<br><H><br>−193.3582743
	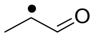<br>**(31R)**		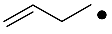<br>**(49R)**		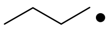<br>**(48R)**	
	E_{tot}	H_{298}	E_{tot}	H_{298}	E_{tot}	H_{298}
UB3LYP	−192.4957378<br>−192.4968916	−192.419812<br>−192.420785<br><H><br>−192.4205240	−156.5479930<br>−156.5471148	−156.449473<br>−156.448608<br><H><br>−156.4492262	−157.7852871<br>−157.7848264	−157.662946<br>−157.662442<br><H><br>−157.6627586
ROMP2	−192.1049337<br>−192.1062969	−192.0290079<br>−192.0301903<br><H><br>−192.0299310	−156.1656335<br>−156.1647294	−156.0671135<br>−156.0662226<br><H><br>−156.0668653	−157.3884319<br>−157.3882067	−157.2660908<br>−157.2658223<br><H><br>−157.2659792
G3(MP2)−RAD	−192.2582774<br>−192.2594781	−192.1823516<br>−192.1833715<br><H><br>−192.1831180	−156.3357210<br>−156.3351567	−156.2372010<br>−156.2366499<br><H><br>−156.2370043	−157.5714929<br>−157.5711403	−157.4491518<br>−157.4487559<br><H><br>−157.4489953

Table S3. Energies and enthalpies of studied pyrimidine bases and their dihydro derivatives at various levels of theory in the gas phase (298.15 K, 1 atm, in Hartree).

	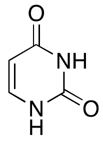 **(5)**		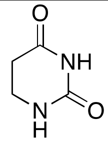 **(5red)**		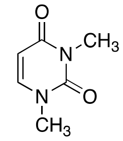 **(7)**	
	**E_tot**	**H_298**	**E_tot**	**H_298**	**E_tot**	**H_298**
B3LYP	−414.8159434	−414.723082	−416.0289298	−415.9123140	−493.4392316	−493.2880060
MP2	−414.0942780	−414.0014156	−415.2957353	−415.1791195	−492.5231659	−492.3719403
G3(MP2)−RAD	−414.3433253	−414.2504637	−415.5603634	−415.4437476	−492.8575198	−492.7062942

	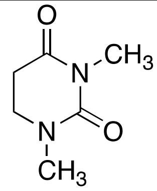 **(7red)**		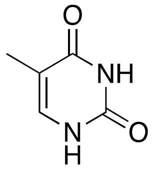 **(6)**		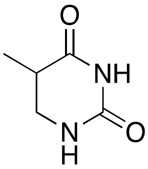 **(6red)**	
	**E_tot**	**H_298**	**E_tot**	**H_298**	**E_tot**	**H_298**
B3LYP	−494.6502952	−494.4754020	−454.1363070	−454.0143320	−455.3449323	−455.1991760
MP2	−493.7221289	−493.5472357	−453.3203336	−453.1983586	−454.5187530	−454.3729967
G3(MP2)−RAD	−494.0722892	−493.8973960	−453.6113003	−453.4893302	−454.8257664	−454.6800101

	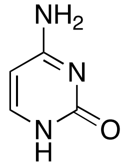 **(8)**		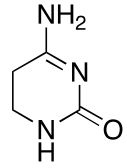 **(8red)**		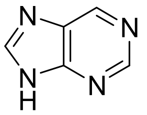 **(27)**	
	**E_tot**	**H_298**	**E_tot**	**H_298**	**E_tot**	**H_298**
B3LYP	−394.9280116	−394.823487	−396.1318383	−396.003514	−411.9463521	−411.846036
MP2	−394.2120332	−394.1075086	−395.4035169	−395.2751926	−411.1656980	−411.0653819
G3(MP2)−RAD	−394.1075086	−394.3643732	−395.6771488	−395.5488245	−411.4345202	−411.3342041

	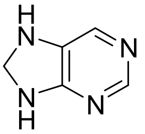 **(27red_a)**		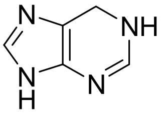 **(27red_b)**		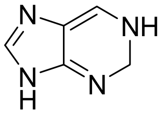 **(27red_c)**	
	**E_tot**	**H_298**	**E_tot**	**H_298**	**E_tot**	**H_298**
B3LYP	−412.1301989	−413.006589	−413.1324971	−413.008528	−413.1109252	−412.987804
MP2	−412.3345434	−413.2109335	−412.3432303	−412.2192612	−412.3176429	−412.1945217
G3(MP2)−RAD	−412.6233834	−412.4997735	−412.6294486	−412.5054795	−412.6078629	−412.4847417

	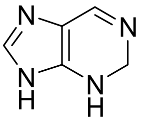 **(27red_d)**		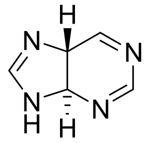 **(27red_e)**		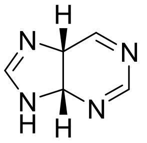 **(27red_f)**	
	**E_tot**	**H_298**	**E_tot**	**H_298**	**E_tot**	**H_298**
B3LYP	−413.1136403	−412.990115	−413.0741241	−412.9516110	−4120932267	−412.9703650
MP2	−412.3252181	−412.2016928	−412.2813681	−412.1588550	−412.2962836	−412.1734219
G3(MP2)−RAD	−412.6125301	−412.4890048	−412.5767815	−412.4542684	−412.5923974	−412.4695357

Table S4. Energies and enthalpies of studied purine bases and their dihydro derivatives at various levels of theory in the gas phase (298.15 K, 1 atm, in Hartree).

	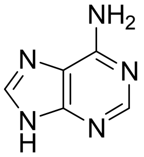 (**25**)		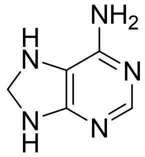 (**25red_a**)		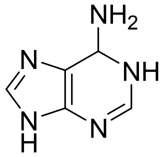 (**25red_b**)	
	**E_{tot}**	**H_{298}**	**E_{tot}**	**H_{298}**	**E_{tot}**	**H_{298}**
B3LYP	−467.3181723	−467.199675	−468.4966380	−468.354402	−468.4878746	−468.345603
MP2	−466.4494932	−466.3309998	−467.6141961	−467.4719598	−467.6134757	−467.4712041
-G3(MP2)-RAD	−466.75333230	−466.6348350	−467.9391229	−467.7968866	−467.9346456	−467.7923740

	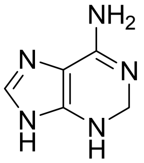 (**25red_c**)		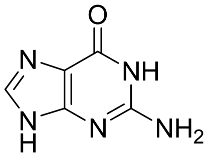 (**26**)		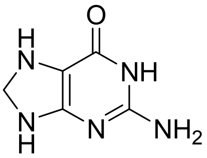 (**26red_a**)	
	**E_{tot}**	**H_{298}**	**E_{tot}**	**H_{298}**	**E_{tot}**	**H_{298}**
B3LYP	−468.4799493	−468.338478	−542.5500873	−542.425985	−543.7270992	−543.579564
MP2	−467.6037901	−467.4623188	−541.5875930	−541.4634907	−542.7510040	−542.6034689
G3(MP2)-RAD	−467.9254881	−467.7840168	−541.9189154	−541.7948131	−543.1036030	−542.9560079

	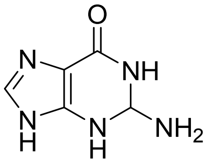 (**26red_b**)	
	**E_{tot}**	**H_{298}**
B3LYP	−543.7145259	−543.566937
MP2	−542.7486877	−542.6010988
G3(MP2)-RAD	−543.0976341	−542.9500452

Table S5. Energies and enthalpies of studied ribose models at various levels of theory in the gas phase (298.15 K, 1 atm, in Hartree).

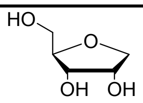

(**13**)

	B3LYP		MP2		G3(MP2)-RAD	
	E_{tot}	H_{298}	E_{tot}	H_{298}	E_{tot}	H_{298}
		<H>		<H>		<H>
				−496.3926203		−496.7225787
002	−497.3972201	−497.230477	−496.5596005	−496.3928574	−496.8895632	−496.7228201
011	−497.3966623	−497.230071	−496.5592698	−496.3926785	−496.8891756	−496.7225843
016	−497.396408	−497.229533	−496.5582756	−496.3914006	−496.8881060	−496.7212310

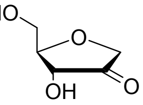

(**12**)

	B3LYP		MP2		G3(MP2)-RAD	
	E_{tot}	H_{298}	E_{tot}	H_{298}	E_{tot}	H_{298}
		<H>		<H>		<H>
		−496.0503862		−495.2157489		−495.5299653
003	−496.1930950	−496.050648	−495.3584727	−495.2160257	−495.672666	−495.5302185
027	−496.1914556	−496.049076	−495.3567252	−495.2143456	−495.670863	−495.5284832
005	−496.1867202	−496.044244	−495.3538885	−495.2114123	−495.668127	−495.5256512

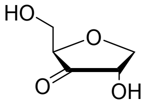

(**10**)

	B3LYP		MP2		G3(MP2)-RAD	
	E_{tot}	H_{298}	E_{tot}	H_{298}	E_{tot}	H_{298}
		<H>		<H>		<H>
		−496.0452432		−495.2104755		−495.5246764
005	−496.1883279	−496.045392	−495.3537861	−495.2108502	−495.6679202	−495.5249843
002	−496.1867424	−496.044049	−495.353377	−495.2106836	−495.6676771	−495.5249837
008	−496.1879531	−496.045105	−495.3530798	−495.2102317	−495.667255	−495.5244069
006	−496.1864131	−496.043627	−495.3527991	−495.210013	−495.6668444	−495.5240583
004	−496.1885387	−496.045615	−495.352261	−495.2093373	−495.6666437	−495.5237200

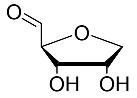

(**11**)

	B3LYP		MP2		G3(MP2)-RAD	
	E_{tot}	H_{298}	E_{tot}	H_{298}	E_{tot}	H_{298}
		<H>		<H>		<H>
		−496.0463035		−495.2126926		−495.5267166
001	−496.1889342	−496.046459	−495.3553908	−495.2129156	−495.6693859	−495.5269107
007	−496.1852568	−496.042536	−495.3520419	−495.2093211	−495.6655164	−495.5227956
009	−496.1849254	−496.042696	−495.3518052	−495.2095758	−495.6659202	−495.5236908
013	−496.1840697	−496.041779	−495.3510202	−495.2087295	−495.6652322	−495.5229415

Table S6. Energies and enthalpies of studied desoxyribose models at various levels of theory in the gas phase (298.15 K, 1 atm, in Hartree).

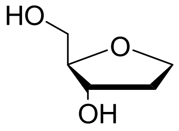

(**51**)

	B3LYP		MP2		G3(MP2)-RAD	
	E_{tot}	H_{298}	E_{tot}	H_{298}	E_{tot}	H_{298}
		<H>		<H>		<H>
		−422.0220199		−421.2723924		−421.5738135
009	−422.1838555	−422.022484	−421.4342941	−421.2729226	−421.7357118	−421.5743403
006	−422.1831374	−422.021856	−421.4328604	−421.271579	−421.7343532	−421.5730718
003	−422.1830676	−422.021595	−421.4318483	−421.2703757	−421.7333124	−421.5718398
015	−422.1825881	−422.021314	−421.4325334	−421.2712593	−421.7342896	−421.5730155

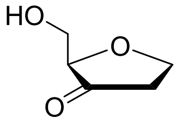

(**39**)

	B3LYP		MP2		G3(MP2)-RAD	
	E_{tot}	H_{298}	E_{tot}	H_{298}	E_{tot}	H_{298}
		<H>		<H>		<H>
		−420.8477979		−420.1003700		−420.3854679
004	−420.9859207	−420.848397	−420.2375691	−420.1000454	−420.5228435	−420.3853198
006	−420.9846209	−420.847150	−420.2377431	−420.1002722	−420.5228589	−420.385388
005	−420.9838296	−420.846388	−420.2377331	−420.1002915	−420.5226863	−420.3852447
007	−420.9848834	−420.847446	−420.237967	−420.1005296	−420.5230925	−420.3856551
002	−420.9827371	−420.845551	−420.2377247	−420.1005386	−420.5227934	−420.3856073

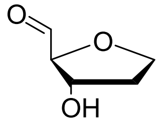

(**40**)

	B3LYP		MP2		G3(MP2)-RAD	
	E_{tot}	H_{298}	E_{tot}	H_{298}	E_{tot}	H_{298}
		<H>		<H>		<H>
		−420.8389221		−420.0934623		−420.3788172
001	−420.9763079	−420.839282	−420.2305171	−420.0934915	−420.5159564	−420.3789305
006	−720.6743994	−420.837599	−420.2305048	−420.0937044	−420.5158041	−420.3790037
002	−720.9734584	−420.836562	−420.2295738	−420.0926774	−420.5146234	−420.377727

Table S7. Energies and enthalpies of studied open-shell ribose models at various levels of theory in the gas phase (298.15 K, 1 atm, in Hartree).

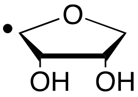

(**41aR**)

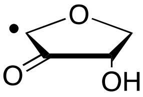

(**33aR**)

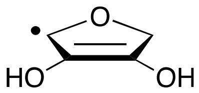

(**38bR**)

	(41aR)		(33aR)		(38bR)	
	**E_{tot}**	**H_{298}**	**E_{tot}**	**H_{298}**	**E_{tot}**	**H_{298}**
UB3LYP	−382.2095753	−382.091592	−381.0266100	−380.931548	−381.0020204	−380.908302
ROMP2	−381.5534556	−381.4354723	−380.3713030	−380.2762410	−380.3552209	−380.2615025
G3(MP2)-RAD	−381.8064076	−381.6884243	−380.6071139	−380.5120519	−380.591977	−380.4982586

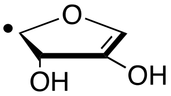

(**37bR**)

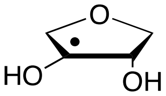

(**41bR**)

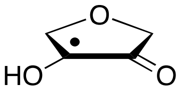

(**37aR**)

	(37bR)		(41bR)		(37aR)	
	**E_{tot}**	**H_{298}**	**E_{tot}**	**H_{298}**	**E_{tot}**	**H_{298}**
UB3LYP	−380.9821186	−380.888269	−382.2110494	−382.092602	−381.0374618	−380.942058
ROMP2	−380.3354956	−380.2416460	−381.5518970	−381.4334496	−380.3789169	−380.2835131
G3(MP2)-RAD	−380.5741869	−380.4803373	−381.8047854	−381.6863380	−380.6142424	−380.5188386

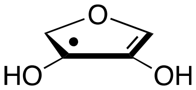

(**37bR**)

	**E_{tot}**	**H_{298}**
UB3LYP	−381.0026090	−380.908927
ROMP2	−380.3571748	−380.2634928
G3(MP2)-RAD	−380.5931358	−380.4994538

Table S8. Energies and enthalpies of studied open-shell ribose models at various levels of theory in the gas phase (298.15 K, 1 atm, in Hartree).

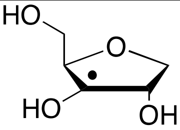

(**13cR**)

	**UB3LYP**		**ROMP2**		**G3(MP2)-RAD**	
	**E_{tot}**	**H_{298}**	**E_{tot}**	**H_{298}**	**E_{tot}**	**H_{298}**
		<H>		<H>		<H>
		−496.5847643		−495.7439638		−496.0685244
001	−496.7389357	−496.5851850	−495.8984312	−495.7446805	−496.2230396	−496.0692889
022	−496.7389600	−496.5851920	−495.8977324	−495.7439644	−496.2221522	−496.0683842
010	−496.7380026	−496.5844620	−495.8968379	−495.7432973	−496.2214063	−496.0678657
004	−496.7378733	−496.5843880	−495.8966704	−495.7431851	−496.2211722	−496.0676869
009	−496.7378587	−496.5844010	−495.8960390	−495.7425813	−496.2205709	−496.0671132
026	−496.7364926	−496.5829100	−495.8954764	−495.7418938	−496.2200524	−496.0664698
007	−496.7359818	−496.5825730	−495.8948468	−495.7414380	−496.2195662	−496.0661574
028	−496.7353216	−496.5819840	−495.8946429	−495.7413053	−496.2191180	−496.0657804

Table S9. Energies and enthalpies of studied open-shell ribose models at various levels of theory in the gas phase (298.15 K, 1 atm, in Hartree).

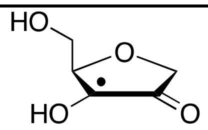

(**12aR**)

	UB3LYP		ROMP2		G3(MP2)-RAD	
	**E**$_{tot}$	**H**$_{298}$	**E**$_{tot}$	**H**$_{298}$	**E**$_{tot}$	**H**$_{298}$
		<H>		<H>		<H>
		−495.4336549		−494.5937203		−494.8999539
001	−495.5645486	−495.4339430	−494.7248278	−494.5942222	−495.0311105	−494.9005049
003	−495.5643379	−495.4337100	−494.7235412	−494.5929133	−495.0300378	−494.8994099
002	−495.5603865	−495.4300660	−494.7221730	−494.5918525	−495.0287338	−494.8984133
008	−495.5625623	−495.4320130	−494.7216880	−494.5911387	−495.0284445	−494.8978952
006	−495.5587599	−495.4284250	−494.7191816	−494.5888467	−495.0257035	−494.8953686
007	−495.5595650	−495.4291350	−494.7190198	−494.5885898	−495.0255795	−494.8951495
010	−495.5585514	−495.4283130	−494.7183161	−494.5880777	−495.0250338	−494.8947954
011	−495.5592788	−495.4287020	−494.7186330	−494.5880562	−495.0252295	−494.8946527

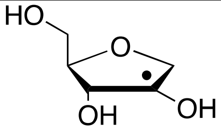

(**13bR**)

	UB3LYP		ROMP2		G3(MP2)-RAD	
	**E**$_{tot}$	**H**$_{298}$	**E**$_{tot}$	**H**$_{298}$	**E**$_{tot}$	**H**$_{298}$
		<H>		<H>		<H>
		−496.5862126		−495.7447389		−496.0689632
002	−496.7402859	−496.5867000	−495.8989489	−495.7453630	−496.2231852	−496.0695993
001	−496.7382715	−496.5845790	−495.8981496	−495.7444571	−496.2222990	−496.0686065
037	−496.7396709	−496.5861030	−495.8976813	−495.7441134	−496.2220197	−496.0684518
003	−496.7375551	−496.5841250	−495.8964631	−495.7430330	−496.2209669	−496.0675368
030	−496.7372751	−496.5836560	−495.8962149	−495.7425958	−496.2204282	−496.0668091
004	−496.7346958	−496.5813720	−495.8953572	−495.7420334	−496.2198706	−496.0665468

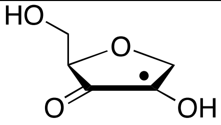

(**10bR**)

	UB3LYP		ROMP2		G3(MP2)-RAD	
	**E**$_{tot}$	**H**$_{298}$	**E**$_{tot}$	**H**$_{298}$	**E**$_{tot}$	**H**$_{298}$
		<H>		<H>		<H>
		−495.4343713		−494.5934030		−494.9001834
005	−495.5609850	−495.4306390	−494.7230698	−494.5927238	−494.9005816	−495.0309276
007	−495.5643016	−495.4337210	−494.7245314	−494.5939508	−494.9003491	−495.0309297
008	−495.5656280	−495.4348800	−494.7240830	−494.5933350	−494.9000286	−495.0307766
009	−495.5639624	−495.4333780	−494.7235357	−494.5929513	−494.8993588	−495.0299432
004	−495.5622536	−495.4317040	−494.7231050	−494.5925554	−494.8988698	−495.0294194

Table S10. Energies and enthalpies of studied open-shell ribose models at various levels of theory in the gas phase (298.15 K, 1 atm, in Hartree)

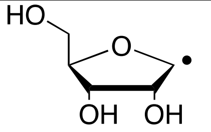

(**13aR**)

	UB3LYP		ROMP2		G3(MP2)-RAD	
	E_{tot}	H_{298}	E_{tot}	H_{298}	E_{tot}	H_{298}
		<H>		<H>		<H>
		−496.5890988		−495.7496618		−496.0738610
026	−496.7429036	−496.5896520	−495.9034825	−495.7502309	−496.2276791	−496.0744275
006	−496.7419682	−496.5887780	−495.9024489	−495.7492587	−496.2268552	−496.0736650
007	−496.7417069	−496.5883290	−495.9018220	−495.7484441	−496.2261835	−496.0728056
009	−496.7400111	−496.5866270	−495.9009916	−495.7476075	−496.2254693	−496.0720852
048	−496.7391764	−496.5860820	−495.8998893	−495.7467949	−496.2245148	−496.0714204
010	−496.7391360	−496.5858180	−495.8990543	−495.7457363	−496.2234430	−496.0701250

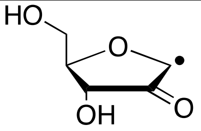

(**12bR**)

	UB3LYP		ROMP2		G3(MP2)-RAD	
	E_{tot}	H_{298}	E_{tot}	H_{298}	E_{tot}	H_{298}
		<H>		<H>		<H>
		−495.4286786		−494.5907064		−494.8981706
001	−495.5593527	−495.4292100	−494.7214832	−494.5913405	−495.0289152	−494.8987725
003	−495.5585438	−495.4284300	−494.7203944	−494.5902806	−495.0277781	−494.8976643
002	−495.5563707	−495.4263570	−494.7194472	−494.5894335	−495.0268017	−494.8967880
008	−495.5565169	−495.4264250	−494.7182927	−494.5882008	−495.0257388	−494.8956469
005	−495.5550092	−495.4251510	−494.7181693	−494.5883111	−495.0253449	−494.8954867
007	−495.5556553	−495.4255100	−494.7177302	−494.5875849	−495.0252485	−494.8951032
010	−495.5553354	−495.4253530	−494.7177918	−494.5878094	−495.0250310	−494.8950486

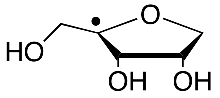

(**13dR**)

	UB3LYP		ROMP2		G3(MP2)-RAD	
	E_{tot}	H_{298}	E_{tot}	H_{298}	E_{tot}	H_{298}
		<H>		<H>		<H>
		−496.5944650		−495.7513101		−496.0757287
011	−496.7484927	−496.5941420	−495.9057420	−495.7513913	−496.2303854	−496.0760347
003	−496.7495057	−496.5949050	−495.9063667	−495.7517660	−496.2306135	−496.0760128
041	−496.7461084	−496.5921500	−495.9039720	−495.7500136	−496.2286865	−496.0747281
034	−496.7440079	−496.5900810	−495.9031911	−495.7492642	−496.2273662	−496.0734393
001	−496.7457353	−496.5917800	−495.9026737	−495.7487184	−496.2269933	−496.0730380
040	−496.7457153	−496.5911340	−495.9024036	−495.7478223	−496.2265507	−496.0719694

Table S10. *Cont.*

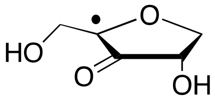

(10aR)

	UB3LYP		ROMP2		G3(MP2)−RAD	
	E_{tot}	H_{298}	E_{tot}	H_{298}	E_{tot}	H_{298}
		<H>		<H>		<H>
		−495.4330721		−494.5918567		−494.8988566
004	−495.5640058	−495.4333660	−494.7228944	−494.5922546	−495.0299643	−494.8993245
001	−495.5635219	−495.4328940	−494.7225180	−494.5918901	−495.0294867	−494.8988588
002	−495.5607439	−495.4301630	−494.7208498	−494.5902689	−495.0282087	−494.8976278
007	−495.5606439	−495.4300360	−494.7207735	−494.5901656	−495.0281588	−494.8975509
006	−495.5579128	−495.4274690	−494.7187897	−494.5883459	−495.0261139	−494.8956701

Table S11. Energies and enthalpies of studied open-shell ribose models at various levels of theory in the gas phase (298.15 K, 1 atm, in Hartree)

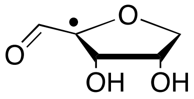

(11R)

	UB3LYP		ROMP2		G3(MP2)-RAD	
	E_{tot}	H_{298}	E_{tot}	H_{298}	E_{tot}	H_{298}
		<H>		<H>		<H>
		−495.4360673		−494.5962383		−494.9021763
001	−495.5667105	−495.4361000	−494.7268751	−494.5962646	−495.0328193	−494.9022088
007	−495.5608047	−495.4302910	−494.7217550	−494.5912413	−495.0286335	−494.8981198

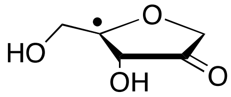

(12cR)

	UB3LYP		ROMP2		G3(MP2)-RAD	
	E_{tot}	H_{298}	E_{tot}	H_{298}	E_{tot}	H_{298}
		<H>		<H>		<H>
		−495.4062032		−494.5672676		−494.8766632
008	−495.5363043	−495.4067260	−494.6972479	−494.5676696	−495.0067052	−494.8771269
001	−495.5344524	−495.4050500	−494.6968455	−494.5674431	−495.0061551	−494.8767527
011	−495.5344614	−495.4048050	−494.6962923	−494.5666359	−495.0054501	−494.8757937
013	−495.5354128	−495.4060260	−494.6949241	−494.5655373	−495.0044191	−494.8750323
003	−495.5316145	−495.4025650	−494.6940222	−494.5649727	−495.0033158	−494.8742663

Table S12. Energies and enthalpies of studied open-shell desoxyribose models at various levels of theory in the gas phase (298.15 K, 1 atm, in Hartree).

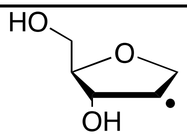

(49bR)

	UB3LYP		ROMP2		G3(MP2)-RAD	
	**E_{tot}**	**H_{298}**	**E_{tot}**	**H_{298}**	**E_{tot}**	**H_{298}**
		<H>		<H>		<H>
		−421.3724607		−420.6222104		−420.9180383
009	−421.5200951	−421.3731570	−420.7696730	−420.6227349	−421.0654868	−420.9185487
003	−421.5184042	−421.3715890	−420.7690937	−420.6222785	−421.0649586	−420.9181434
010	−421.5184392	−421.3716780	−420.7688639	−420.6221027	−421.0646903	−420.9179291
011	−421.5183745	−421.3715130	−420.7683399	−420.6214784	−421.0643432	−420.9174817
007	−421.5164382	−421.3697830	−420.7676499	−420.6209947	−421.0635209	−420.9168657
008	−421.5174683	−421.3705550	−420.7668700	−420.6199567	−421.0627337	−420.9158204
013	−421.5114896	−421.3651000	−420.7637524	−420.6173628	−421.0597762	−420.9133866

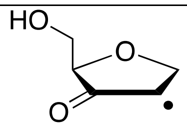

(39aR)

	UB3LYP		ROMP2		G3(MP2)−RAD	
	**E_{tot}**	**H_{298}**	**E_{tot}**	**H_{298}**	**E_{tot}**	**H_{298}**
		<H>		<H>		<H>
		−420.2099567		−419.4586582		−419.7388688
008	−420.3327589	−420.2090650	−419.5829335	−419.4592396	−419.8630729	−419.7393790
009	−420.3343007	−420.2104560	−419.5823908	−419.4585461	−419.8628199	−419.7389752
002	−420.3322767	−420.2085770	−419.5816695	−419.4579698	−419.8619609	−419.7382612
005	−420.3295910	−420.2061030	−419.5815823	−419.4580943	−419.8616669	−419.7381789
004	−420.3306986	−420.2070770	−419.5812724	−419.4576508	−419.8614524	−419.7378308

Table S13. Energies and enthalpies of studied open-shell desoxyribose models at various levels of theory in the gas phase (298.15 K, 1 atm, in Hartree).

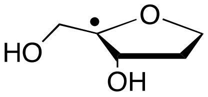

(49dR)

	UB3LYP		ROMP2		G3(MP2)-RAD	
	**E_{tot}**	**H_{298}**	**E_{tot}**	**H_{298}**	**E_{tot}**	**H_{298}**
		<H>		<H>		<H>
		−421.3854345		−420.6315212		−420.9273656
003	−421.5346756	−421.3857810	−420.7808311	−420.6319365	−421.0767406	−420.9278460
001	−421.5322712	−421.3837910	−420.7785680	−420.6300878	−421.0745078	−420.9260276
012	−421.5303108	−421.3819380	−420.7775152	−420.6291424	−421.0737101	−420.9253373
013	−421.5298020	−421.3813270	−420.7770852	−420.6286102	−421.0732112	−420.9247362
006	−421.5300162	−421.3816650	−420.7751596	−420.6268084	−421.0713262	−420.9229750

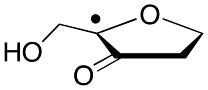

(38bR)

	UB3LYP		ROMP2		G3(MP2)-RAD	
	**E_{tot}**	**H_{298}**	**E_{tot}**	**H_{298}**	**E_{tot}**	**H_{298}**
		<H>		<H>		<H>
		−420.2314156		−419.4776997		−419.7560982
001	−420.3567811	−420.2315070	−419.6032351	−419.4779610	−419.8816649	−419.7563908
002	−420.3530989	−420.2278850	−419.6011761	−419.4759622	−419.8798062	−419.7545923
005	−420.3502802	−420.2252490	−419.5991268	−419.4740956	−419.8776532	−419.7526220

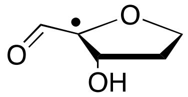

(40R)

	UB3LYP		ROMP2		G3(MP2)-RAD	
	**E_{tot}**	**H_{298}**	**E_{tot}**	**H_{298}**	**E_{tot}**	**H_{298}**
		<H>		<H>		<H>
		−420.2278730		−419.4764909		−419.7541275
004	−420.3532614	−420.2280760	−419.6018749	−419.4766895	−419.8794258	−419.7542404
005	−420.3512756	−420.2260690	−419.6013255	−419.4761189	−419.8792097	−419.7540031

Table S14. Energies and enthalpies of studied uridinyl radicals at various levels of theory in the gas phase (298.15 K, 1 atm, in Hartree)

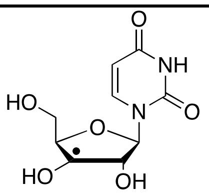

(44R)

	**UB3LYP**		**ROMP2**		**G3(MP2)-RAD**	
	**E_{tot}**	**H_{298}**	**E_{tot}**	**H_{298}**	**E_{tot}**	**H_{298}**
		<H>		<H>		<H>
		−910.1392473		−908.5948328		−909.1504578
007	−910.3664589	−910.140049	−908.8218201	−908.5954102	−909.3774355	−909.1510256
022	−910.3653081	−910.139071	−908.8202557	−908.5940186	−909.3760215	−909.1497844
029	−910.3645489	−910.138495	−908.8197226	−908.5936687	−909.3756425	−909.1495886
001	−910.3647645	−910.138453	−908.8215667	−908.5952552	−909.3772423	−909.1509308
016	−910.3646612	−910.138337	−908.8191918	−908.5928676	−909.3750343	−909.1487101
006	−910.3640039	−910.137808	−908.8195240	−908.5933281	−909.3753207	−909.1491248
011	−910.3637063	−910.137285	−908.8192463	−908.5928250	−909.3749907	−909.1485694
002	−910.3624828	−910.136200	−908.8191042	−908.5928214	−909.3747354	−909.1484526
	**ΔG_{solv} IEF-PCM/UAHF/ UHF/6-31G(d)/**	**H_{sol}**		**H_{sol}**		**H_{sol}**
		<H_{sol}>		<H_{sol}>		<H_{sol}>
		−910.1728247		−908.6279782		−909.1836984
007	−0.0317446	−910.1717936		−908.6271548		−909.1827702
022	−0.0346130	−910.1736840		−908.6286316		−909.1843974
029	−0.0334816	−910.1719766		−908.6271503		−909.1830702
001	−0.0326848	−910.1711378		−908.6279400		−909.1836156
016	−0.0338003	−910.1721373		−908.6266679		−909.1825104
006	−0.0316489	−910.1694569		−908.6249770		−909.1807737
011	−0.0349158	−910.1722008		−908.6277408		−909.1834852
002	−0.0351549	−910.1713549		−908.6279763		−909.1836075

Table S15. Energies and enthalpies of studied uridinyl radicals at various levels of theory in the gas phase (298.15 K, 1 atm, in Hartree).

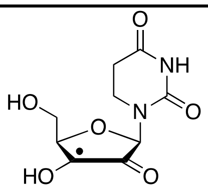

(41aR)

	**UB3LYP**		**ROMP2**		**G3(MP2)-RAD**	
	**E_{tot}**	**H_{298}**	**E_{tot}**	**H_{298}**	**E_{tot}**	**H_{298}**
		<H>		<H>		<H>
		−910.1725864		−908.6209887		−909.1737467
018	−910.4000892	−910.173117	−908.8484744	−908.6215022	−909.4013094	−909.1743372
006	−910.3980589	−910.170862	−908.8461554	−908.6189585	−909.3986315	−909.1714346
034	−910.397375	−910.170378	−908.8460842	−908.6190872	−909.399403	−909.1724060
040	−910.3967976	−910.169766	−908.8447738	−908.6177422	−909.3976715	−909.1706399
031	−910.3964794	−910.169468	−908.8448253	−908.6178139	−909.3983395	−909.1713281
025	−910.3961569	−910.169164	−908.8441937	−908.6172008	−909.397007	−909.1700141
022	−910.3957370	−910.168869	−908.8451302	−908.6182622	−909.3979019	−909.1709090
	**ΔG_{solv} IEF-PCM/UAHF/ UHF/6-31G(d)/**	**H_{sol}**		**H_{sol}**		**H_{sol}**
		<H_{sol}>		<H_{sol}>		<H_{sol}>
		−910.2126416		−908.6609990		−909.2137393
018	−0.0396488	−910.2127658		−908.6611510		−909.2139860
006	−0.0354736	−910.2063356		−908.6544321		−909.2069082
034	−0.0389636	−910.2093416		−908.6580508		−909.2113696
040	−0.0372106	−910.2069766		−908.6549528		−909.2078505
031	−0.0378321	−910.2073001		−908.6556460		−909.2091602
025	−0.0373700	−910.2065340		−908.6545708		−909.2073841
022	−0.0385971	−910.2074661		−908.6568593		−909.2095061

Table S16. Energies and enthalpies of studied uridinyl radicals at various levels of theory in the gas phase (298.15 K, 1 atm, in Hartree).

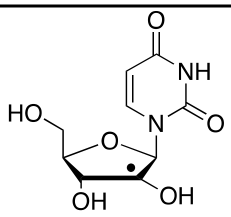

(44bR)

	**UB3LYP**		**ROMP2**		**G3(MP2)-RAD**	
	**E_{tot}**	**H_{298}**	**E_{tot}**	**H_{298}**	**E_{tot}**	**H_{298}**
		<H>		<H>		<H>
		−910.1404794		−908.5973043		−909.1530543
025	−910.3674962	−910.141315	−908.8238780	−908.5976968	−909.3796653	−909.1534841
006	−910.3663915	−910.140426	−908.8234801	−908.5975146	−909.3794525	−909.1534870
017	−910.3661894	−910.140144	−908.8237596	−908.5977142	−909.3793673	−909.1533219
014	−910.3660818	−910.140032	−908.8218777	−908.5958273	−909.3778423	−909.1517925
010	−910.3661606	−910.139888	−908.8220833	−908.5958107	−909.3779337	−909.1516611
005	−910.3655244	−910.139435	−908.8218906	−908.5958012	−909.3778962	−909.1518068
020	−910.3644661	−910.139360	−908.8216169	−908.5954687	−909.3775941	−909.1524880
018	−910.3644661	−910.138423	−908.8216348	−908.5955917	−909.3775161	−909.151473
004	−910.3641611	−910.138064	−908.8197640	−908.5936669	−909.3758322	−909.1497351
	**ΔG_{solv} IEF-PCM/UAHF/ UHF/6-31G(d)/**	**H_{sol}**		**H_{sol}**		**H_{sol}**
		<H_{sol}>		<H_{sol}>		<H_{sol}>
		−910.1774647		−908.6343478		−909.1899521
025	−0.0365254	−910.1778404		−908.6342222		−909.1900095
006	−0.0351708	−910.1755968		−908.6326854		−909.1886578
017	−0.0372425	−910.1773865		−908.6349567		−909.1905644
014	−0.0377524	−910.1777844		−908.6335803		−909.1895449
010	−0.0336728	−910.1735608		−908.6294835		−909.1853339
005	−0.0317446	−910.1711796		−908.6275458		−909.1835514
020	−0.0365413	−910.1759013		−908.6320100		−909.1890293
018	−0.0364776	−910.1749006		−908.6320693		−909.1879506
004	−0.0361907	−910.1742547		−908.6298576		−909.1859258

Table S17. Energies and enthalpies of studied uridinyl radicals at various levels of theory in the gas phase (298.15 K, 1 atm, in Hartree).

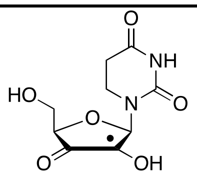

(42aR)

	UB3LYP		ROMP2		G3(MP2)-RAD	
	**E_{tot}**	**H_{298}**	**E_{tot}**	**H_{298}**	**E_{tot}**	**H_{298}**
		<H>		<H>		<H>
		−910.1737546		−908.6204229		−909.1735282
004	−910.4012167	−910.174298	−908.8480150	−908.6210963	−909.4009499	−909.1740312
008	−910.4009077	−910.173946	−908.8474979	−908.6205362	−909.4003928	−909.1734311
007	−910.4006944	−910.173603	−908.8445872	−908.6174959	−909.3978214	−909.1707300
003	−910.4003671	−910.173463	−908.8443338	−908.6174297	−909.3975667	−909.1706626
014	−910.1721520	−910.172152	−908.8469492	−908.6197707	−909.3998964	−909.1738269
019	−910.3982215	−910.171548	−908.8457340	−908.6190605	−909.3989084	−909.1722349
017	−910.3982919	−910.171204	−908.8460953	−908.6190074	−909.3990516	−909.1719637
034	−910.3980878	−910.171018	−908.8454791	−908.6184093	−909.3992915	−909.1722217

	**ΔG_{solv} IEF-PCM/UAHF/ UHF/6-31G(d)/**	**H_{sol}**		**H_{sol}**		**H_{sol}**
		<H_{sol}>		<H_{sol}>		<H_{sol}>
		−910.2078702		−908.6549679		−909.2085338
004	−0.0335294	−910.2078274		−908.6546257		−909.2075606
008	−0.0336888	−910.2076348		−908.6542250		−909.2071199
007	−0.0345334	−910.2081364		−908.6520292		−909.2052634
003	−0.0347405	−910.2082035		−908.6521702		−909.2054031
014	−0.0345493	−910.2067013		−908.6543200		−909.2083762
019	−0.0362704	−910.2078184		−908.6553309		−909.2085053
017	−0.0359198	−910.2071238		−908.6549272		−909.2078835
034	−0.0369716	−910.2079896		−908.6553809		−909.2091933

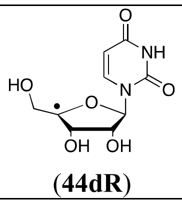

(44dR)

	UB3LYP		ROMP2		G3(MP2)−RAD	
	**E_{tot}**	**H_{298}**	**E_{tot}**	**H_{298}**	**E_{tot}**	**H_{298}**
		<H>		<H>		<H>
		−910.1414372		−908.5964747		−909.1527074
025	−910.3684648	−910.141883	−908.8236613	−908.5970795	−909.3799215	−909.1533397
008	−910.3661481	−910.139772	−908.8207562	−908.5943801	−909.3769973	−909.1506212
036	−910.3657102	−910.138977	−908.8220360	−908.5953028	−909.3780845	−909.1513513
048	−910.3655868	−910.138791	−908.8218973	−908.5951015	−909.3780882	−909.1512924
015	−910.3644762	−910.137891	−908.8222872	−908.5957020	−909.3788239	−909.1522387

Table S17. *Cont.*

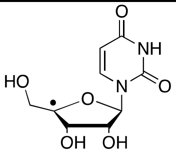

(**44dR**)

	UB3LYP		ROMP2	G3(MP2)-RAD
	**ΔGsolv IEF-PCM/UAHF/ UHF/6-31G(d)/**	**Hsol**	**Hsol**	**Hsol**
		<Hsol>	<Hsol>	<Hsol>
		−910.1758315	−908.6330825	−909.1896124
025	−0.0331788	−910.1750618	−908.6302583	−909.1865185
008	−0.0319358	−910.1717078	−908.6263159	−909.1825570
036	−0.0373222	−910.1762992	−908.6326250	−909.1886735
048	−0.0362067	−910.1749977	−908.6313082	−909.1874991
015	−0.0378321	−910.1757231	−908.6335341	−909.1900708

Table S18. Energies and enthalpies of studied uridinyl radicals at various levels of theory in the gas phase (298.15 K, 1 atm, in Hartree).

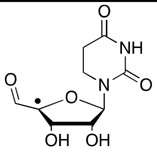

(**43aR**)

	UB3LYP		ROMP2		G3(MP2)-RAD	
	**Etot**	**H298**	**Etot**	**H298**	**Etot**	**H298**
		<H>		<H>		<H>
		−910.1692663		−908.6171885		−909.1709251
023	−910.3965350	−910.169704	−908.8443837	−908.6175527	−909.3981146	−909.1712836
025	−910.3947267	−910.167927	−908.8421603	−908.6153606	−909.3959421	−909.1691424
008	−910.3940192	−910.167364	−908.8415184	−908.6148632	−909.3946787	−909.1680235
007	−910.3930112	−910.166435	−908.8406424	−908.6140662	−909.3937429	−909.1671667
	**ΔGsolv IEF-PCM/UAHF/ UHF/6-31G(d)/**	**Hsol**		**Hsol**		**Hsol**
		<Hsol>		<Hsol>		<Hsol>
		−910.2018660		−908.6494336		−909.2028630
023	−0.0319039	−910.2016079		−908.6494566		−909.2031875
025	−0.0334178	−910.2013448		−908.6487784		−909.2025602
008	−0.0349796	−910.2023436		−908.6498428		−909.2030031
007	−0.0348999	−910.2013349		−908.6489661		−909.2020666

Table S19. Energies and enthalpies of studied uridinyl radicals at various levels of theory in the gas phase (298.15 K, 1 atm, in Hartree).

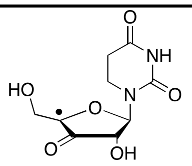

(42bR)

	UB3LYP		ROMP2		G3(MP2)-RAD	
	**E_{tot}**	**H_{298}**	**E_{tot}**	**H_{298}**	**E_{tot}**	**H_{298}**
		<H>		<H>		<H>
		−910.1667808		−908.6140645		−909.1686461
017	−910.3939700	−910.167430	−908.8404581	−908.6139181	−909.3942829	−909.1677429
011	−910.3939989	−910.167312	−908.8417578	−908.6150709	−909.3963689	−909.1696820
014	−910.3933674	−910.166754	−908.8398747	−908.6132613	−909.3937218	−909.1671084
026	−910.3930580	−910.166500	−908.8395743	−908.6130163	−909.3933345	−909.1667765
024	−910.3924285	−910.165864	−908.8388548	−908.6122903	−909.3926964	−909.1661319
016	−910.3920458	−910.165378	−908.8392185	−908.6125507	−909.3938731	−909.1672053
047	−910.3916412	−910.165033	−908.8386118	−908.6120036	−909.3932097	−909.1666015
031	−910.3907606	−910.164173	−908.8384723	−908.6118847	−909.3925804	−909.1659928
004	−910.3907381	−910.164108	−908.8360205	−908.6093904	−909.3909259	−909.1642958
009	−910.3905193	−910.163887	−908.8393586	−908.6127263	−909.3942491	−909.1676168
003	−910.3903952	−910.163778	−908.8356604	−908.6090432	−909.3906115	−909.1639943
029	−910.3900716	−910.163519	−908.8379142	−908.6113616	−909.3920216	−909.1654690
061	−910.3899909	−910.163352	−908.8355369	−908.6088980	−909.3902496	−909.1636107
057	−910.3895568	−910.162941	−908.8382875	−908.6116717	−909.3931822	−909.1665664
005	−910.3895803	−910.162906	−908.8379664	−908.6112921	−909.3932337	−909.1665594
007	−910.3894949	−910.162903	−908.8348492	−908.6082573	−909.3895906	−909.1629987
018	−910.3888004	−910.162372	−908.8375249	−908.6110965	−909.3916349	−909.1652065
010	−910.3888184	−910.162210	−908.8370766	−908.6104682	−909.3919609	−909.1653525

	**ΔG_{solv} IEF-PCM/UAHF/ UHF/6-31G(d)/**	**H_{sol}**	**H_{sol}**	**H_{sol}**
		<H_{sol}>	<H_{sol}>	<H_{sol}>
		−910.2052304	−908.6528871	−909.2075782
017	−0.0381349	−910.2055649	−908.6520530	−909.2058778
011	−0.0379756	−910.2052876	−908.6530465	−909.2076576
014	−0.0389636	−910.2057176	−908.6522249	−909.2060720
026	−0.0391708	−910.2056708	−908.6521871	−909.2059473
024	−0.0390592	−910.2049232	−908.6513495	−909.2051911
016	−0.0385652	−910.2039432	−908.6511159	−909.2057705
047	−0.0386130	−910.2036460	−908.6506166	−909.2052145
031	−0.0414496	−910.2056226	−908.6533343	−909.2074424
004	−0.0376728	−910.2017808	−908.6470632	−909.2019686
009	−0.0408600	−910.2047470	−908.6535863	−909.2084768

Table S19. *Cont.*

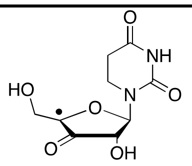

(42bR)

	UB3LYP		ROMP2	G3(MP2)-RAD
	**ΔG_{solv} IEF-PCM/UAHF/ UHF/6-31G(d)/**	**H_{sol}**	**H_{sol}**	**H_{sol}**
003	−0.0379118	−910.2016898	−908.6469550	−909.2019061
029	−0.0412424	−910.2047614	−908.6526040	−909.2067114
061	−0.0374975	−910.2008495	−908.6463955	−909.2011082
057	−0.0413699	−910.2043109	−908.6530416	−909.2079363
005	−0.0382783	−910.2011843	−908.6495704	−909.2048377
007	−0.0381030	−910.2010060	−908.6463603	−909.2011017
018	−0.0416568	−910.2040288	−908.6527533	−909.2068633
010	−0.0408918	−910.2031018	−908.6513600	−909.2062443

Table S20. Energies and enthalpies of studied uridinyl radicals at various levels of theory in the gas phase (298.15 K, 1 atm, in Hartree).

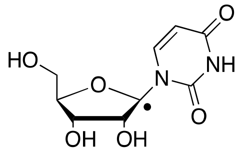

(44aR)

	UB3LYP		ROMP2		G3(MP2)-RAD	
	**E_{tot}**	**H_{298}**	**E_{tot}**	**H_{298}**	**E_{tot}**	**H_{298}**
		<H>		<H>		<H>
		−910.1440298		−908.5985539		−909.1548322
022	−910.3709609	−910.144709	−908.8244957	−908.5982438	−909.3812269	−909.1549750
009	−910.3707755	−910.144399	−908.8237197	−908.5973432	−909.3805048	−909.1541283
073	−910.3705720	−910.144115	−908.8231906	−908.5967336	−909.3801665	−909.1537095
010	−910.3700834	−910.143879	−908.8237039	−908.5974995	−909.3805722	−909.1543678
014	−910.3693185	−910.143173	−908.8241676	−908.5980221	−909.3808797	−909.1547342
003	−910.3695661	−910.143131	−908.8259405	−908.5995054	−909.3821488	−909.1557137
005	−910.3691660	−910.142914	−908.8231324	−908.5968803	−909.3798925	−909.1536405
015	−910.3689906	−910.142449	−908.8246616	−908.5981200	−909.3808999	−909.1543583
023	−910.3682453	−910.142178	−908.8222318	−908.5961645	−909.3790728	−909.1530055
017	−910.3677804	−910.141771	−908.8223483	−908.5963389	−909.3791571	−909.1531477
026	−910.3679035	−910.141711	−908.8214930	−908.5953005	−909.3780489	−909.1518564

Table S20. *Cont.*

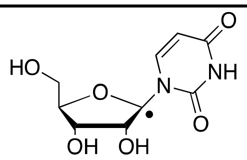

(**44aR**)

	UB3LYP		ROMP2	G3(MP2)-RAD
	**ΔG_{solv} IEF-PCM/UAHF/ UHF/6-31G(d)/**	**H_{sol}**	**H_{sol}**	**H_{sol}**
		<H_{sol}>	<H_{sol}>	<H_{sol}>
		−910.1771427	−908.6336516	−909.1897439
022	−0.0312984	−910.1760074	−908.6295422	−909.1862734
009	−0.0296569	−910.1740559	−908.6270001	−909.1837852
073	−0.0291948	−910.1733098	−908.6259284	−909.1829043
010	−0.0328920	−910.1767710	−908.6303915	−909.1872598
014	−0.0318880	−910.1750610	−908.6299101	−909.1866222
003	−0.0345971	−910.1777281	−908.6341025	−909.1903108
005	−0.0304219	−910.1733359	−908.6273022	−909.1840624
015	−0.0352346	−910.1776836	−908.6333546	−909.1895929
023	−0.0345812	−910.1767592	−908.6307457	−909.1875867
017	−0.0343740	−910.1761450	−908.6307129	−909.1875217
026	−0.0340872	−910.1757982	−908.6293877	−909.1859436

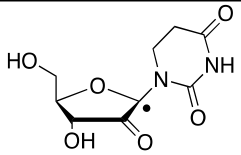

(**41bR**)

	UB3LYP		ROMP2		G3(MP2)-RAD	
	**E_{tot}**	**H_{298}**	**E_{tot}**	**H_{298}**	**E_{tot}**	**H_{298}**
		<H>		<H>		<H>
		−910.1724027		−908.6207030		−909.1740419
003	−910.3998174	−910.172796	−908.8481619	−908.6211405	−909.4014105	−909.1743891
004	−910.3986426	−910.171605	−908.8463553	−908.6193177	−909.3997694	−909.1727318
012	−910.3966897	−910.169768	−908.8459416	−908.6190199	−909.3978891	−909.1709674
006	−910.3959926	−910.168975	−908.8442775	−908.6172599	−909.3964746	−909.1694570

	**ΔG_{solv} IEF-PCM/UAHF/ UHF/6-31G(d)/**	**H_{sol}**	**H_{sol}**	**H_{sol}**
		<H_{sol}>	<H_{sol}>	<H_{sol}>
		−910.2065923	−908.6549800	−909.2082502
003	−0.0342306	−910.2070266	−908.6553711	−909.2086197
004	−0.0329398	−910.2045448	−908.6522575	−909.2056716
012	−0.0356011	−910.2053691	−908.6546210	−909.2065685
006	−0.0348362	−910.2038112	−908.6520961	−909.2042932

Table S21. Energies and enthalpies of studied uridinyl radicals at various levels of theory in the gas phase (298.15 K, 1 atm, in Hartree).

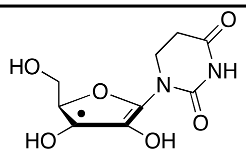

(41cR)

	**UB3LYP**		**ROMP2**		**G3(MP2)-RAD**	
	**E_{tot}**	**H_{298}**	**E_{tot}**	**H_{298}**	**E_{tot}**	**H_{298}**
		<H>		<H>		<H>
		−910.1501197		−908.6047301		−909.1570670
013	−910.3767445	−910.150730	−908.8307220	−908.6047075	−909.3831237	−909.1571092
015	−910.3763869	−910.150556	−908.8313908	−908.6055599	−909.3835725	−909.1577416
050	−910.3760145	−910.150190	−908.8306331	−908.6048086	−909.3832335	−909.1574090
009	−910.3757337	−910.149737	−908.8293879	−908.6033912	−909.3816343	−909.1556376
077	−910.3756005	−910.149604	−908.8293250	−908.6033285	−909.3815428	−909.1555463
010	−910.3753370	−910.149541	−908.8300332	−908.6042372	−909.3820802	−909.1562742
029	−910.3749247	−910.149144	−908.8292525	−908.6034718	−909.3817863	−909.1560056
052	−910.3746681	−910.148951	−908.8296750	−908.6039579	−909.3826392	−909.1569221
064	−910.3733917	−910.147801	−908.8291477	−908.6035570	−909.3810726	−909.1554819
032	−910.3734192	−910.147785	−908.8280928	−908.6024586	−909.3809674	−909.1553332
	**ΔG_{solv} IEF-PCM/UAHF/ UHF/6-31G(d)/**	**H_{sol}**		**H_{sol}**		**H_{sol}**
		<H_{sol}>		<H_{sol}>		<H_{sol}>
		−910.1832491		−908.6384169		−909.1905213
013	−0.0311071	−910.1818371		−908.6358146		−909.1882163
015	−0.0331788	−910.1837348		−908.6387387		−909.1909204
050	−0.0316171	−910.1818071		−908.6364257		−909.1890261
009	−0.0338322	−910.1835692		−908.6372234		−909.1894698
077	−0.0315533	−910.1811573		−908.6348818		−909.1870996
010	−0.0338322	−910.1833732		−908.6380694		−909.1901064
029	−0.0340234	−910.1831674		−908.6374952		−909.1900290
052	−0.0337206	−910.1826716		−908.6376785		−909.1906427
064	−0.0356170	−910.1834180		−908.6391740		−909.1910989
032	−0.0346927	−910.1824777		−908.6371513		−909.1900259

Table S22. Energies and enthalpies of studied uridinyl radicals at various levels of theory in the gas phase (298.15 K, 1 atm, in Hartree).

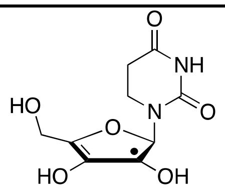

(42cR)

	**UB3LYP**		**ROMP2**		**G3(MP2)-RAD**	
	**E_{tot}**	**H_{298}**	**E_{tot}**	**H_{298}**	**E_{tot}**	**H_{298}**
		<H>		<H>		<H>
		−910.1479311		−908.6035572		−909.1569155
004	−910.3748849	−910.148801	−908.8304122	−908.6043283	−909.3832439	−909.1571600
002	−910.3747171	−910.148707	−908.8297656	−908.6037555	−909.3832922	−909.1572821
003	−910.3730306	−910.147143	−908.8291285	−908.6032409	−909.3824266	−909.1565390
001	−910.3728604	−910.147141	−908.8291756	−908.6034562	−909.3817016	−909.1559822
009	−910.3730747	−910.147067	−908.8298582	−908.6038505	−909.3832426	−909.1572349
014	−910.3728936	−910.147045	−908.8295389	−908.6036903	−909.3834026	−909.1575540
013	−910.3725547	−910.146817	−908.8286920	−908.6029543	−909.3822069	−909.1564692
026	−910.3728370	−910.146695	−908.8284112	−908.6022692	−909.3817346	−909.1555926
011	−910.3726297	−910.146691	−908.8284132	−908.6024745	−909.3823165	−909.1563778
005	−910.3724864	−910.146595	−908.8286255	−908.6027341	−909.3816298	−909.1557384
032	−910.3729310	−910.146524	−908.8266122	−908.6002052	−909.3798103	−909.1534033
008	−910.3721682	−910.146428	−908.8277617	−908.6020215	−909.3810829	−909.1553427
034	−910.3723201	−910.146184	−908.8280655	−908.6019294	−909.3813644	−909.1552283
020	−910.3722397	−910.146048	−908.8264528	−908.6002611	−909.3777566	−909.1515649
	**ΔG_{solv} IEF-PCM/UAHF/ UHF/6-31G(d)/**	**H_{sol}**		**H_{sol}**		**H_{sol}**
		<H_{sol}>		<H_{sol}>		<H_{sol}>
		−910.1829662		−908.6394185		−909.1917546
004	−0.0333063	−910.1821073		−908.6403756		−909.1904663
002	−0.0331469	−910.1818539		−908.6369024		−909.1904290
003	−0.0355533	−910.1826963		−908.6387942		−909.1920923
001	−0.0360473	−910.1831883		−908.6367625		−909.1920295
009	−0.0335613	−910.1806283		−908.6377942		−909.1907962
014	−0.0335135	−910.1805585		−908.6394507		−909.1910675
013	−0.0334497	−910.1802667		−908.6365156		−909.1899189
026	−0.0367007	−910.1833957		−908.6370894		−909.1922933
011	−0.0348202	−910.1815112		−908.6359242		−909.1911980
005	−0.0339437	−910.1805387		−908.6362476		−909.1896821
032	−0.0366210	−910.1831450		−908.6375114		−909.1900243
008	−0.0357604	−910.1821884		−908.6387222		−909.1911031
034	−0.0370991	−910.1832831		−908.6385504		−909.1923274
020	−0.0373062	−910.1833542		−908.6373602		−909.1888711

Table S23. Energies and enthalpies of studied uridinyl radicals at various levels of theory in the gas phase (298.15 K, 1 atm, in Hartree).

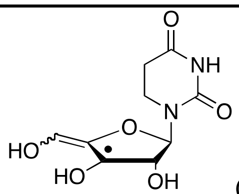

(**43bR**)

	UB3LYP		ROMP2		G3(MP2)-RAD	
	**E_{tot}**	**H_{298}**	**E_{tot}**	**H_{298}**	**E_{tot}**	**H_{298}**
		<H>		<H>		<H>
		−910.1475300		−908.6034406		−909.1569809
016	−910.3738691	−910.147993	−908.8298603	−908.6039842	−909.3831515	−909.1572754
007	−910.3732249	−910.147421	−908.8287356	−908.8287356	−909.3820922	−909.1568603
012	−910.3722436	−910.146427	−908.8285703	−908.8285703	−909.3827738	−909.1569572
011	−910.3717313	−910.145989	−908.8280772	−908.8280772	−909.3821750	−909.1564327
	**ΔG_{solv} IEF-PCM/UAHF/ UHF/6-31G(d)/**	**H_{sol}**		**H_{sol}**		**H_{sol}**
		<H_{sol}>		<H_{sol}>		<H_{sol}>
		−910.1809926		−908.6372946		−909.1914728
016	−0.0326370	−910.1806300		−908.6366212		−909.1899124
007	−0.0331788	−910.1805998		−908.6361105		−909.1900391
012	−0.0348840	−910.1813110		−908.6376377		−909.1918412
011	−0.0351071	−910.1810961		−908.6374420		−909.1915398

Table S24. Energies and enthalpies of studied cytidinyl radicals **16R** at various levels of theory in the gas phase (298.15 K, 1 atm, in Hartree).

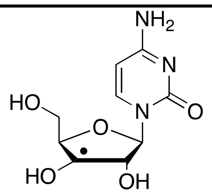

(**16R**)

	UB3LYP		ROMP2		G3(MP2)-RAD	
	**E_{tot}**	**H_{298}**	**E_{tot}**	**H_{298}**	**E_{tot}**	**H_{298}**
		<H>		<H>		<H>
		−890.2460349		−888.7066289		−889.2696342
007	−890.4849277	−890.246767	−888.9455584	−888.7073977	−889.5085576	−889.2703969
016	−890.4832920	−890.245216	−888.9432443	−888.7051683	−889.5063917	−889.2683157
029	−890.4831391	−890.245185	−888.9434718	−888.7055177	−889.5066110	−889.2686569
006	−890.4821766	−890.244315	−888.9430036	−888.7051420	−889.5060853	−889.2682237
022	−890.4821561	−890.244292	−888.9425999	−888.7047358	−889.5056856	−889.2678215
001	−890.4821270	−890.244060	−888.9442329	−888.7061659	−889.5073369	−889.2692699
011	−890.4819000	−890.243714	−888.9427660	−888.7045800	−889.5059140	−889.2677280

Table S24. *Cont.*

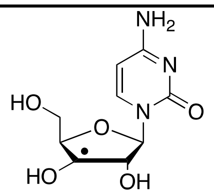

(**16R**)

	UB3LYP		ROMP2	G3(MP2)-RAD
	ΔG_{solv} IEF-PCM/UAHF/ UHF/6-31G(d)/	H_{sol}	H_{sol}	H_{sol}
		<H_{sol}>	<H_{sol}>	<H_{sol}>
		−890.2854490	−888.7459704	−889.3090066
007	−0.03920262	−890.2859696	−888.7466003	−889.3095995
016	−0.04036595	−890.2855820	−888.7455343	−889.3086817
029	−0.03984007	−890.2850251	−888.7453578	−889.3084970
006	−0.03901139	−890.2833264	−888.7441534	−889.3072351
022	−0.04124244	−890.2855344	−888.7459782	−889.3090639
001	−0.03953728	−890.2835973	−888.7457032	−889.3088072
011	−0.04100340	−890.2847174	−888.7455834	−889.3087314

Table S25. Energies and enthalpies of studied cytidinyl radicals **19R** at various levels of theory in the gas phase (298.15 K, 1 atm, in Hartree).

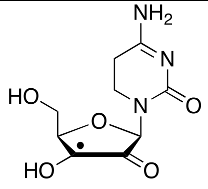

(**19R**)

	UB3LYP		ROMP2		G3(MP2)-RAD	
	E_{tot}	H_{298}	E_{tot}	H_{298}	E_{tot}	H_{298}
		<H>		<H>		<H>
		−890.2675709		−888.7201525		−889.2818173
018	−890.5064534	−890.267775	−888.9590225	−888.7203441	−889.5207963	−889.2821179
034	−890.5032064	−890.264483	−888.9565121	−888.7177887	−889.5188906	−889.2801672
006	−890.5031211	−890.264261	−888.9555468	−888.7166867	−889.5169264	−889.2780663
	ΔG_{solv} IEF-PCM/UAHF/ UHF/6-31G(d)/	H_{sol}		H_{sol}		H_{sol}
		<H_{sol}>		<H_{sol}>		<H_{sol}>
		−890.3177047		−888.7702194		−889.3319488
018	−0.04995944	−890.3177344		−888.7703035		−889.3320773
034	−0.04868456	−890.3131676		−888.7664733		−889.3288518
006	−0.04554516	−890.3098062		−888.7622319		−889.3236115

3. Energies and Enthalpies Related to Radical Stabilization Energies

The radical stabilisation energy (RSE) is defined as reaction enthalpy of the hydrogen atom transfer to the methyl radical.

$$\text{R–H} + {}^{\bullet}\text{CH}_3 \xrightarrow[= \Delta H]{\text{RSE}} \text{R}^{\bullet} + \text{CH}_4$$

Table S26. Energies and enthalpies of studied compounds (closed- and open-shell) at various levels of theory in the gas phase (298.15 K, 1 atm, in Hartree).

	$^{\bullet}CH_3$ **(32R)**		CH_4 **(32)**		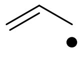 **(14R)**	
	**E_{tot}**	**H₂₉₈**	**E_{tot}**	**H₂₉₈**	**E_{tot}**	**H₂₉₈**
UB3LYP	−39.8382922	−39.804975	−40.5183890	−40,470240	−117.2603540	−117.190479
ROMP2	−39.7316815	−39.6983643	−40.4055437	−40,3573947	−116.9739045	−116.9040295
G3(MP2)-RAD	−39.7851922	−39.7518750	−40.4651605	−40.4170115	−117.0984522	−117.0285772

	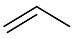 **(14)**		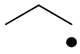 **(33R)**		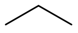 **(33)**	
	**E_{tot}**	**H₂₉₈**	**E_{tot}**	**H₂₉₈**	**E_{tot}**	**H₂₉₈**
UB3LYP	−117.9075622	−117.823957	−118.4711107	−118.377919	−119.1442483	−119.036640
ROMP2	−117.6166619	−117.5330709	−118.1690627	−118.0758710	−118.8387689	−118.7311606
G3(MP2)-RAD	−117.7498900	−117.6662848	−118.3090897	−118.2158980	−118.9847884	−118.8771801

	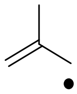 **(28R)**		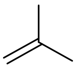 **(28)**		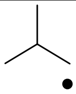 **(34R)**	
	**E_{tot}**	**H₂₉₈**	**E_{tot}**	**H₂₉₈**	**E_{tot}**	**H₂₉₈**
UB3LYP	−156.5772554	−156.478343	−157.2272881	−157.114574	−157.7856471	−157.663659
ROMP2	−156.1966271	−156.0977147	−156.8420116	−156.7292975	−157.3910101	−157.2690220
G3(MP2)-RAD	−156.3632755	−156.2643631	−157.0170527	−156.9043386	−157.5736006	−157.4516125

	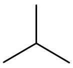 **(34)**		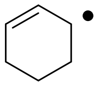 **(21R)**		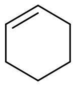 **(21)**	
	**E_{tot}**	**H₂₉₈**	**E_{tot}**	**H₂₉₈**	**E_{tot}**	**H₂₉₈**
UB3LYP	−158.4588061	−158.322279	−234.0081729	−233.871204	−234.6482883	−234.497595
ROMP2	−158.0609392	−157.9244121	−233.4544966	−233.3175277	−234.0908015	−233.9401082
G3(MP2)-RAD	−158.2492314	−158.1127043	−233.6883122	−233.5513433	−234.3346577	−234.1839644

	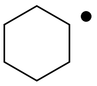 **(35R)**		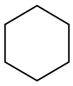 **(35)**		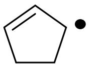 **(23R)**	
	**E_{tot}**	**H₂₉₈**	**E_{tot}**	**H₂₉₈**	**E_{tot}**	**H₂₉₈**
UB3LYP	−235.2139163	−235.053530	−235.8804309	−235.705840	−194.6860804	−194.579304
ROMP2	−234.6443077	−234.4839214	−235.3102004	−235.1356095	−194.2269215	−194.1201451
G3(MP2)-RAD	−234.8956477	−234.7352614	−235.5671031	−235.3925122	−194.4182277	−194.3114513

Table S27. Energies and enthalpies of studied compounds (closed- and open-shell) at various levels of theory in the gas phase (298.15 K, 1 atm, in Hartree).

	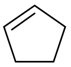 **(23)**		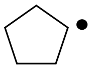 **(36R)**		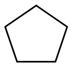 **(36)**	
	**E_{tot}**	**H_{298}**	**E_{tot}**	**H_{298}**	**E_{tot}**	**H_{298}**
UB3LYP	−195.3271387	−195.206463	−195.8948865	−195.764645	−196.5570819	−196.412193
ROMP2	−194.8640871	−194.7434114	−195.4190525	−195.2888110	−196.0804928	−195.9356039
G3(MP2)-RAD	−195.0650164	−194.9443407	−195.6277641	−195.4975226	−196.2948337	−196.1499448
	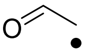 **(4R)**		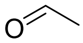 **(4)**		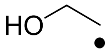 **(1R)**	
	**E_{tot}**	**H_{298}**	**E_{tot}**	**H_{298}**	**E_{tot}**	**H_{298}**
UB3LYP	−153.1715366	−153.125159	−153.8301215	−153.770515	−154.3613832	−154.291870
ROMP2	−152.8787261	−152.8323485	−153.5380653	−153.4784588	−154.0666330	−153.9971198
G3(MP2)-RAD	−152.9894640	−152.9430864	−153.6538781	−153.5942716	−154.1927918	−154.1232786
	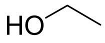 **(1)**		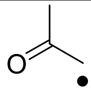 **(9R)**		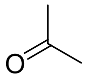 **(9)**	
	**E_{tot}**	**H_{298}**	**E_{tot}**	**H_{298}**	**E_{tot}**	**H_{298}**
UB3LYP	−155.0342871	−154.950239	−192.4950213	−192.419618	−193.1556942	−193.066834
ROMP2	−154.7365194	−154.6524713	−192.1068777	−192.0314744	−192.7680234	−192.6791632
G3(MP2)−RAD	−154.8685341	−154.7844860	−192.2600568	−192.1846535	−192.9263022	−192.8374420
	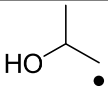 **(20R)**		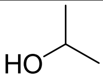 **(20)**			
	**E_{tot}**	**H_{298}**	**E_{tot}**	**H_{298}**		
UB3LYP	−193.6803856	−193.581855	−194.3534513	−194.240614		
ROMP2	−193.2916261	−193.1930955	−193.9614458	−193.848685		
G3(MP2)-RAD	−193.4603518	−193.3618212	−194.1361518	−194.0233145		

Table S28. Radical stabilization energies at various levels of theory in the gas phase (298.15 K, 1 atm, in kJ/mol).

$$R{-}H + {}^{\bullet}CH_3 \xrightarrow{RSE} R^{\bullet} + CH_4$$

**R•**	**RSE**			**RSE (exp.)** [a]
	**UB3LYP**	**ROMP2**	**G3(MP2)-RAD**	
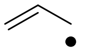 **(14R)**	−83.44	−78.74	−71.97	**−70.3**<br>−70.7 [b]
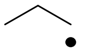 **(33R)**	−17.18	−9.82	−10.12	**−17.5**<br>−17.1 [b]
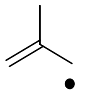 **(31R)**	−76.23	−72.06	−66.06	**−66.5**

[a]: Using $\Delta_f H^0$ of radicals from ref. [15]; $\Delta_f H^0$ of closed-shell compounds from ref. [16]; [b]: from ref. [17].

Table S29. Radical stabilization energies at various levels of theory in the gas phase (298.15 K, 1 atm, in kJ/mol).

$$R{-}H + {}^{\bullet}CH_3 \xrightarrow{RSE} R^{\bullet} + CH_4$$

R$^{\bullet}$	RSE			RSE (exp.) [a]
	UB3LYP	ROMP2	G3(MP2)-RAD	
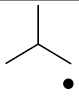 (34R)	−17.45	−9.56	−10.62	**−21.0**<br>−20.1 [b]
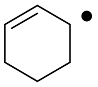 (21R)	−102.06	−95.70	−85.37	**−96.6**
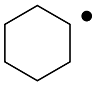 (35R)	−34.01	−19.28	−20.70	**−39.7**<br>−23.0 [b]
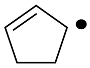 (23R)	−100.05	−93.90	−84.66	**−94.6**
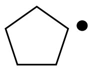 (36R)	−46.52	−32.13	−33.38	**−37.9**<br>−39.3 [b]
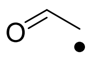 (4R)	−52.27	−33.92	−36.63	**−44.6**<br>−44.7 [b]
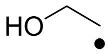 (1R)	−18.11	−9.66	−10.32	**−17.5**<br>−15.5 [b]
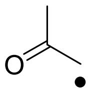 (9R)	−47.39	29.78	−32.42	**−38.1**<br>−38.1 [b]
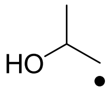 (20R)	−17.08	−9.03	−9.57	**−44.9**

[a]: Using $\Delta_f H^0$ of radicals from ref. [15]; $\Delta_f H^0$ of closed-shell compounds from ref. [16]; [b]: from ref. [17].

4. Experimental Thermochemical Data

Table S30. Benson increment system (BGVA) for the assignment of heats of formation.

System	Benson Notation	BGVA [kJ·mol^{-1}]	Δ_fH(BGVA) [kJ·mol^{-1}]	Δ_fH(exp.) [kJ·mol^{-1}]
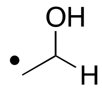 (1R)	C• – (C)(H$_2$)<br>C – (C•)(O)(H$_2$)<br>O – (C)(H)	+160.7 [a]<br>−39.6 [b]<br>−158.6 [b]	−37.5	−31.0 ± 7.0 [a]
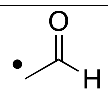 (4R)	C• – (CO)(H$_2$)<br>CO – (C•)(H)	+155.4 [b]<br>−145.0 [b]	+10.4	+10.5 ± 9.20 [d]
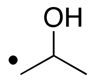 (20R)	C• – (C)(H$_2$)<br>C – (C•)(C)(O)(H)<br>C – (C)(H$_3$)<br>O – (C)(H)	+160.7 [a]<br>−51.5 [b]<br>−41.8 [c]<br>−158.6 [b]	−91.2	−96.2 ± 4.2 [a]
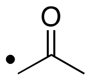 (9R)	C• – (CO)(H$_2$)<br>CO – (C•)(C)<br>C – (CO)(H$_3$)	+155.4 [b]<br>−146.9 [b]<br>−41.8 [c]	−33.3	−33.9 ± 3.0 [a]

Table S30. *Cont.*

System	Benson Notation	BGVA [kJ·mol^{-1}]	Δ_fH(BGVA) [kJ·mol^{-1}]	Δ_fH(exp.) [kJ·mol^{-1}]
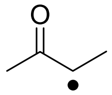 **(47R)**	C – (CO)(H_3)<br>CO – (C)(C•)<br>C• – (CO)(C)(H)<br>C – (C•)(H_3)	−41.8 [c]<br>−132.6 [b]<br>+138.9 [b]<br>−41.8 [a]	−77.3	−70.3 ± 7.1 [a]
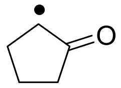 **(30R)**	C• – (CO)(C)(H)<br>C – (C•)(C)(H_2)<br>C – (C_2)(H_2)<br>C – (CO)(C)(H_2)<br>CO – (C•)(C)<br>Cyclopentanone	+138.9 [b]<br>−20.9 [a]<br>−20.9 [c]<br>−21.8 [c]<br>−132.6 [b]<br>+25.9 [c]	−31.4	−41.8 ± 12.6 [a]
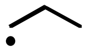 **(33R)**	C• – (C)(H_2)<br>C – (C•)(C)(H_2)<br>C – (C)(H_3)	+160.7 [a]<br>−20.9 [a]<br>−41.8 [c]	+98.0	+100.0 ± 2.0 [a]
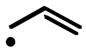 **(14R)**	C• – (C_D)(H_2)<br>C – (C•)(C)(H_2)<br>C – (C)(H_3)	+108.4 [a]<br>+36.0 [a]<br>+26.4[c]	+170.8	+171.0 ± 3.0 [e]
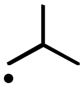 **(34R)**	C• – (C)(H_2)<br>C – (C•)(C_2)(H)<br>2 × C – (C)(H_3)	+160.7 [a]<br>−10.0[a]<br>2× (−41.8) [a]	+67.1	+66.11 ± 1.3 [f]
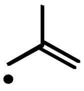 **(28R)**	C• – (C_D)(H_2)<br>C_D – (C•)(C)<br>C – (C_D)(H_3)<br>C_D – (H_2)	+108.4 [a]<br>+42.7 [a]<br>−41.8 [c]<br>+26.4 [c]	+135.7	+137.9 [a]
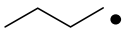 **(50R)**	C• – (C)(H_2)<br>C – (C•)(C)(H_2)<br>C – (C_2)(H_2)<br>C – (C)(H_3)	+160.7 [a]<br>−20.9 [a]<br>−20.9 [c]<br>−41.8 [c]	+77.1	+77.8 ± 2.1 [a]
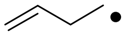 **(49R)**	C• – (C)(H_2)<br>C – (C•)(C)(H_2)<br>C_D – (C)(C_D)<br>C_D – (H_2)	+160.7 [a]<br>−20.9 [a]<br>+36.8 [c]<br>+26.4 [c]	+203.0	+192.5 [a]
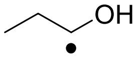 **(46aR)**	C – (C)(H_3)<br>C – (C)(C•)(H_2)<br>C• – (C)(O)(H)<br>O – (C•)(H)	−41.8 [c]<br>−17.4 [a]<br>+117.4 [b]<br>−146.3 [b]	−88.1	−81.0 ± 8.0 [a]
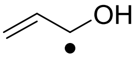 **(29R)**	C_D – (C_D)(H_2)<br>C_D – (C_D)(H)(C•)<br>C• – (C_D)(O)(H)<br>O – (C•)(H)	+26.4 [c]<br>+36.0 [a]<br>+84.08 [b]<br>−146.25 [b]	+0.23	0.0 ± 8.4 [a]

[a]: from ref. [15]; [b]: from ref. [18]; [c]: from ref. [19]; [d]: from ref. [20]; [e]: from ref. [21]; [f]: from ref. [22].

Table S31. Benson increment system (BGVA) for the assignment of heats of formation.

System	Benson Notation	BGVA [kJ·mol^{-1}]	Δ_fH(BGVA) [kJ·mol^{-1}]	Δ_fH(exp.) [kJ·mol^{-1}]
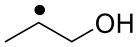 (**46bR**)	C – (C•)(H_3)	−41.8 [a]	−68.5	−78.7 ± 8.4 [a]
	C• – (C_2)(H)	+171.5 [a]		
	C – (C•)(O)(H_2)	−39.6 [b]		
	O – (C)(H)	−158.6 [c]		
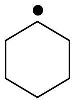 (**35R**)	C• – (C_2)(H)	+171.5 [a]	64.1	+58.2 ± 4.0 [b]
	2× C – (C•)(C)(H_2)	2× (−20.9) [a]		
	3× C – (C_2)(H_2)	3× (−20.9) [c]		
	Cyclohexane	+2.9 [c]		
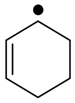 (**21R**)	C• – (C_D)(C)(H)	+109.6 [a]	+121.8	+119.7 [a]
	C – (C)(C•)(H_2)	−20.9 [a]		
	C – (C_2)(H_2)	−20.9 [c]		
	C – (C)(C_D)(H_2)	−20.1 [c]		
	C_D – (C)(H)	+36.0 [c]		
	C_D – (C•)(H)	+36.0 [a]		
	Cyclohexene	+2.1 [c]		
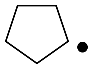 (**36R**)	C• – (C_2)(H)	+171.5 [a]	+117.6	+107.0 ± 2.5 [b]
	2× C – (C•)(C)(H_2)	2× (−20.9) [a]		
	2× C – (C_2)(H_2)	2× (−20.9) [c]		
	Cyclopentane	+29.7 [c]		
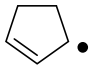 (**23R**)	C• – (C_D)(C)(H)	+109.6 [a]	+165.3	+160.7 ± 4.2 [a]
	C – (C•)(C)(H_2)	−20.9 [a]		
	C – (C)(C_D)(H_2)	−20.1 [c]		
	C_D – (C)(H)	+36.0 [c]		
	C_D – (C•)(H)	+36.0 [a]		
	Cyclopentene	+24.7 [c]		

[a]: from ref. [15]; [b]: from ref. [18]; [c]: from ref. [19].

Table S32. Calculated heats of formation using Benson's increment system (in kJ·mol^{-1}).

System	Benson Notation	BGVA	Δ_fH(BGVA)	Δ_fH(corr.) [a]
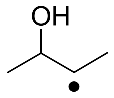 (48R)	C – (C•)(H$_3$)	−41.8 [b]	−122.3	−121.5
	C• – (C$_2$)(H)	+171.5 [b]		
	C – (C•)(C)(O)(H)	−51.5 [c]		
	O – (C)(H)	−158.6 [d]		
	C – (C)(H$_3$)	−41.9 [d]		
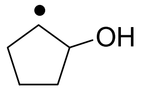 (45R)	C• – (C$_2$)(H)	+171.5 [b]	−71.6	−71.6
	C – (C•)(C)(H2)	−20.9 [b]		
	2x C – (C2)(H2)	2× (−20.9) [d]		
	C – (C•)(C)(O)(H)	−51.5 [c]		
	O – (C)(H)	−158.6 [d]		
	Cyclopentane	+29.7 [d]		
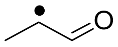 (31R)	C – (C•)(H$_3$)	−41.8 [b]	−33.6	−34.2
	C• – (CO)(C)(H)	+153.2 [c]		
	CO – (C•)(H)	−145.0 [c]		

[a]: Using Δ_fH(corr.) = 0.9838 Δ_fH (BGVA) – 1.1779; [b]: from ref. [15]. [c]: from ref. [18]; [d]: from ref. [19].

Figure S1. Correlation of experimental available heats of formation $\Delta_f H^0$(exp.) *vs.* calculated $\Delta_f H^0$(BGVA) using Benson's group additivity values.

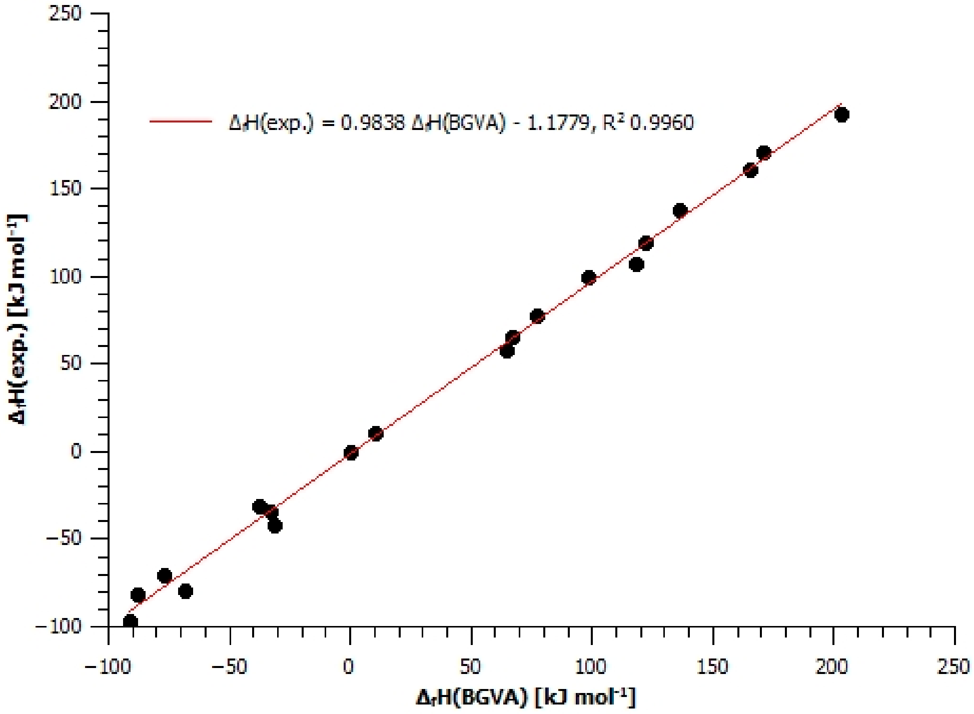

5. Calculated Reaction Enthalpies

Table S33. Transfer hydrogenation enthalpies $\Delta_{trh}H$ applying experimentally available heats of formation Δ_fH (298.15 K, 1 atm, in kJ·mol^{-1}).

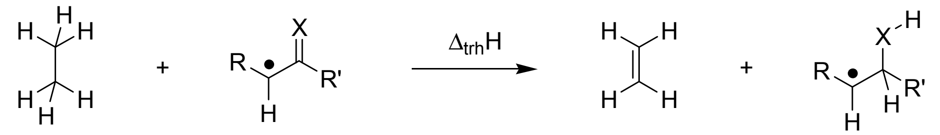

	Δ_fH^0 (exp.)		Δ_fH^0(exp.)	$\Delta_{trh}H^0$ (exp.)
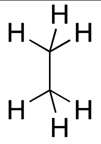 (3)	−84.0 ± 0.4 [a]	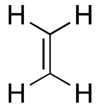 (2)	+52.4 ± 0.5 [a]	
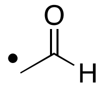 (4R)	+10.5 ± 9.2 [e]	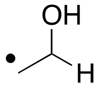 (1R)	−31.0 ± 7.0 [c]	**+94.9 ± 17.1**
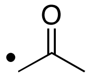 (9R)	−33.9 ± 3.0 [c]	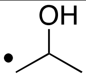 (20R)	−96.2 ± 4.2 [c]	**+74.1 ± 8.1**
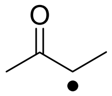 (47R)	−70.3 ± 7.1 [c]	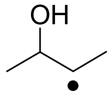 (48R)	−121.5 [f]	**+85.2 ± 8.0**
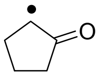 (30R)	−41.8 ± 12.6 [c]	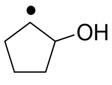 (45R)	−71.6 [f]	**+106.6 ± 13.5**
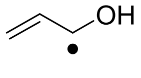 (29R)	0.0 ± 8.4 [c]	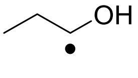 (46aR)	−81.0 ± 8.0 [c]	**+55.4 ± 17.3**
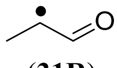 (31R)	−34.2 [f]	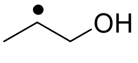 (46bR)	−78.7 ± 8.4 [c]	**+91.9 ± 9.3**
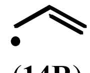 (14R)	+171.0 ± 3.0 [d]	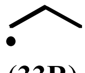 (33R)	+100.0 ± 2.0 [c]	**+65.4 ± 5.9**
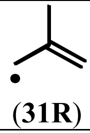 (31R)	+137.9 [c]	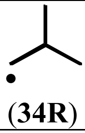 (34R)	+66.1 ± 1.3 [b]	**+64.6 ± 2.2**
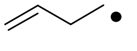 (49R)	+192.5 [c]	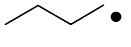 (50R)	+77.8 ± 2.1 [c]	**+21.7 ± 3.0**
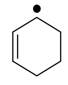 (21R)	+119.7 [c]	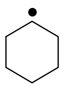 (35R)	+58.2 ± 4.0 [g]	**+74.9 ± 4.9**
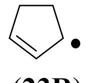 (23R)	+160.7 ± 4.2 [c]	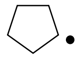 (36R)	+107.0 ± 2.5 [g]	**+82.7 ± 7.6**

[a]: from ref. [23]; [b]: from ref. [22]; [c]: from ref. [15]; [d]: from ref. [21]; [e]: from ref. [20]; [f]: from Table S32; [g]: from ref. [18].

Table S34. Calculated reaction enthalpies ΔH for transfer hydrogenation at various levels of theory in the gas phase at 298.15 K (in kJ·mol^{-1}).

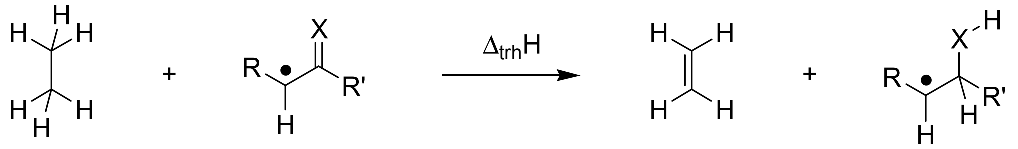

	UB3LYP		ROMP2		G3(MP2)-RAD		Δ_{trh}H(exp.)
	Δ_{trh}H	Δ_{hyd}H [a]	Δ_{trh}H	Δ_{hyd}H [a]	Δ_{trh}H	Δ_{hyd}H [a]	
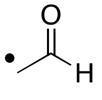 **(4R)**	+137.25	+0.95	+90.07	−46.23	+91.36	−44.94	+94.9 ± 17.1
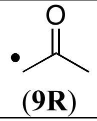 **(9R)**	+149.00	+12.7	+98.34	−37.96	+99.30	−37.00	+74.1 ± 8.1
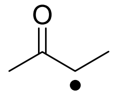 **(47R)**	+164.95	+28.65	+111.69	−24.61	+111.38	−24.92	+85.2 ± 8.0
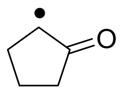 **(30R)**	+161.59	+25.29	+105.71	−30.59	+106.57	−29.73	+106.6 ± 13.5
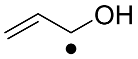 **(29R)**	+79.54	−56.76	+62.46	−73.84	+64.92	−71.38	+55.4 ± 17.3
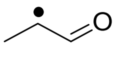 **(31R)**	+154.62	+18.32	+104.84	−31.46	+104.58	−31.72	+91.9 ± 9.3
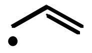 **(14R)**	+82.83	−53.47	+71.51	−64.79	+72.65	−63.65	+65.4 ± 5.9
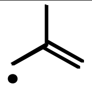 **(28R)**	+88.41	−47.89	+72.91	−63.39	+72.83	−63.47	+64.6 ± 2.2
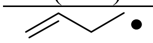 **(49R)**	+14.32	−121.98	−0.10	−136.4	+7.88	−128.42	+21.7 ± 3.0
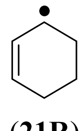 **(21R)**	+96.26	−40.04	+85.81	−50.49	+81.58	−54.72	+74.9 ± 4.9
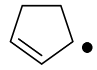 **(23R)**	+88.34	−47.96	+79.85	−56.45	+75.93	−60.37	+82.7 ± 7.6

[a]: Addition of the reaction enthalpies Δ_{trh}H to the experimentally hydrogenation enthalpy of ethylene of Δ_{hyd}H (C_2H_4) = −136.3 ± 0.2 kJ·mol^{-1} yields the hydrogenation enthalpies Δ_{hyd}H of the respective double bond.

Figure S2. Correlation of experimental available heats of transfer hydrogenation $\Delta_{trh}H$ (exp.) *vs*. calculated $\Delta_{trh}H$(calc.) at various levels of theory in the gas phase (298.15 K, 1 atm).

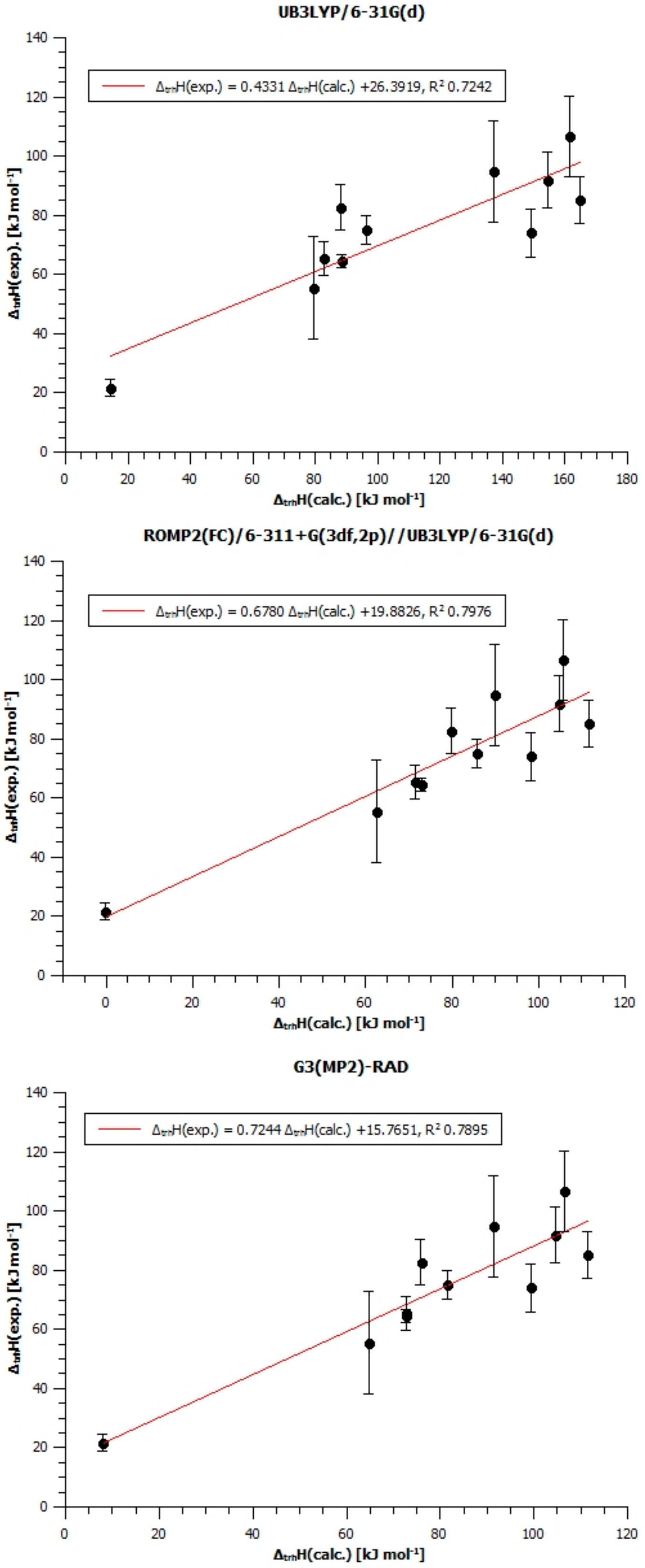

The large discrepancy of calculated *vs.* experimental reaction enthalpies for the reaction of *iso*-propanol derived radical **20R** and the acetonyl radical **9R** motivated us to perform additional investigations:

1. We assume that the deviation from the experiment derives from the experimental $\Delta_f H$ value of isopropyl radical **20R** ($\Delta_f H$ (exp.) = −96.2 ± 4.2 kJ·mol^{-1}, taken from [15,24], since more expensive calculations (see Table S35) supported previous findings.

Table S35. Calculated reaction enthalpies ΔH for transfer hydrogenation at various levels of theory in the gas phase at 298.15 K (in kJ·mol^{-1}).

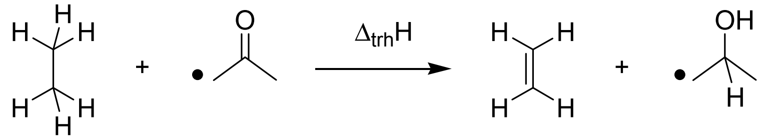

	Δ_{trh}H(calc.)	Δ_{trh}H(exp.)	Δ_{trh}H(exp.) [BGAV]
UB3LYP/6-31G(d)	+149.00		
ROMP2(FC)/6-311 + G(3df,2p)// UB3LYP/6-31G(d)	+98.34	+74.1 ± 8.1	+78.5
G3(MP2)-RAD	+99.30		
G3(MP2)(+)-RAD(p)[a]	+101.90		
G3B3[b]	+104.51		

[a]: from ref. [25]; [b]: frome ref. [26].

Table S36. Literature known $\Delta_f H$ for **20R** and consequential $\Delta_{trh}H$ (exp.) (in kJ·mol^{-1}).

	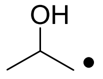 **20R**			Literature
$\Delta_f H^0$	**−62.55**	$\Delta_{trh}H^a$ (exp.)	**+107.75**	CBSQ//B3LYP/6-31G(d,p) Isodesmic reactions analysis [27]
	**−64.02**		**+106.28**	CBS-QB3 Isodesmic reaction analysis [28]

[a]: Using heats of formation $\Delta_f H$ from Table S33 except for **20R**.

2. The calculated heats of formation of **20R** at CBS-QB3 level have been validated using the O-/C-radical isomerization of **20R**. Unfortunately, the isomerization enthalpy is not known in the literature, we therefore applied calculated enthalpy values (see Table S37).

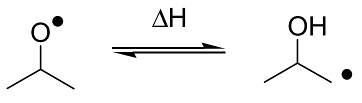

20_OR **20R**

$\Delta_{rxn}H$ (calc.) = BDE (•$CH_2CH(OH)CH_3$) – BDE (($CH_3)_2CHO$•)

BDE (•$CH_2CH(OH)CH_3$) = BDE (CH_4) + RSE(•$CH_2CH(OH)CH_3$) =
BDE (CH_4) + $\Delta_f H$ (CH_4) + $\Delta_f H$ (•$CH_2CH(OH)CH_3$) – $\Delta_f H$ (•CH_3) – $\Delta_f H$ ($CH_3CH(OH)CH_3$)

Table S37. Calculated reaction enthalpies for the isomerization of 20R at various levels of theory (in kJ·mol^{-1}).

	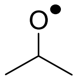 **20_OR**		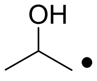 **20R**		
	$\mathbf{E_{tot}}$	$\mathbf{H_{298}}$	$\mathbf{E_{tot}}$	$\mathbf{H_{298}}$	$\mathbf{\Delta_{rxn}H}$ **(calc.)**
UB3LYP	−193.6887947	−193.589865	−193.6803856	−193.581855	**+21.03**
ROMP2	−193.2713835	−193.1724538	−193.2916261	−193.1930955	**−54.19**
UG3B3	−193.6088951	−193.5118434	−193.6151693	−193.5184887	**−17.45**
CBS-QB3 [a]	−193.329811	−193.328867	−193.335952	−193.335008	**−16.12**

[a]: from ref. [29].

Δ_fH of radical **20R** can then be calculated by Equation (1)

$$\Delta_fH\ (\bullet CH_2CH(OH)CH_3) = \Delta_{rxn}H\ (calc.) - BDE\ (CH_4) - \Delta_fH\ (CH_4) + \Delta_fH\ (\bullet CH_3) + \\ + \Delta_fH\ (CH_3CH(OH)CH_3) + BDE\ ((CH_3)_2CHO\bullet) \quad (1)$$

Table S38. Calculated heat of formation of **20R** at various levels of theory (in kJ·mol^{-1}).

	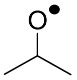	CH_4	$\bullet CH_3$	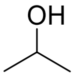
BDE [a]	+442.3 ± 2.8	+439.3 ± 0.4		
Δ_fH [b]		−74.6	146.7 ± 0.3	−272.6
		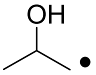		
		UB3LYP/6-31G(d)		
Δ_fH		**−27.27**		
		ROMP2		
Δ_fH		**−102.49**		
		G3B3		
Δ_fH		**−65.75**		
		CBS-QB3		
Δ_fH		**−64.42**		

[a]: BDEs and $\Delta_fH(\bullet CH_3)$ from ref. [15] [b]: from ref. [16].

Table S39. Boltzmann-averaged reaction enthalpies <ΔH> for open-shell sugar models at various levels of theory in the gas phase (298.15 K, 1 atm, in kJ·mol^{-1}).

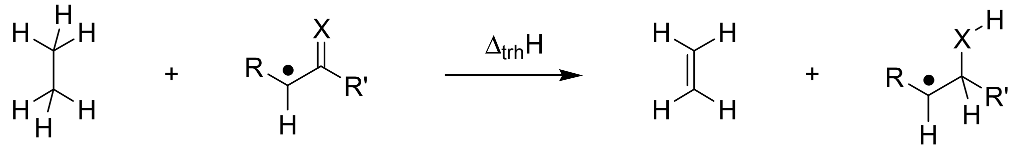

	UB3LYP		ROMP2		G3(MP2)-RAD	
	<Δ_{trh}H>	<Δ_{hyd}H> [a]	<Δ_{trh}H>	<Δ_{hyd}H> [a]	<Δ_{trh}H>	<Δ_{hyd}H> [a]
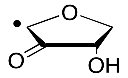 **(38aR)**	+154.76	+18.46	+104.61	−31.69	+101.39	−34.91
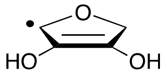 **(38bR)**	+93.73	−42.57	+65.92	−70.38	+65.18	−71.12
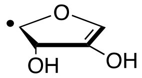 **(37cR)**	+41.13	−95.17	+13.79	−122.51	+18.13	−118.17
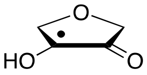 **(37aR)**	+179.70	+43.40	+129.02	−7.28	+124.69	−11.61
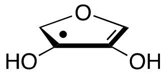 **(37bR)**	+92.71	−43.59	+76.46	−59.84	+73.79	−62.51
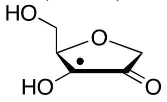 **(12aR)**	+178.22	+41.92	+128.21	−8.09	+121.88	−14.42
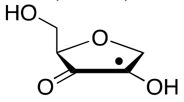 **(10bR)**	+176.29	+39.99	+125.35	−10.95	+121.33	−14.97
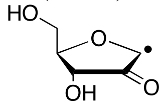 **(12bR)**	+153.77	+17.47	+105.34	−30.96	+103.18	−33.12
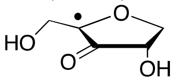 **(10aR)**	+151.22	+14.92	+104.03	−32.27	+100.08	−36.22
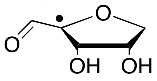 **(11R)**	+159.08	+22.78	+115.54	−20.76	+108.80	−27.50
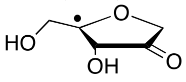 **(12cR)**	+80.67	−55.63	+39.47	−96.83	+41.81	−94.49

Table S39. *Cont.*

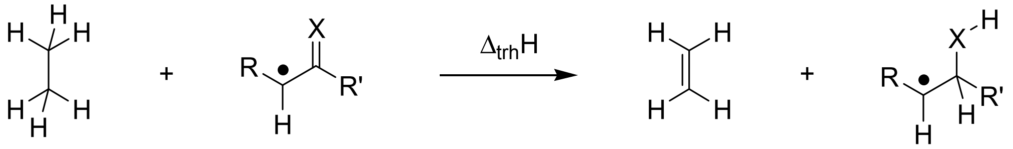

	UB3LYP		ROMP2		G3(MP2)-RAD	
	**<Δ_{trh}H>**	**<Δ_{hyd}H>** [a]	**<Δ_{trh}H>**	**<Δ_{hyd}H>** [a]	**<Δ_{trh}H>**	**<Δ_{hyd}H>** [a]
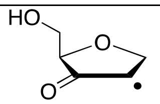 **(39aR)**	+148.30	+12.00	+93.27	−43.03	+94.05	−42.25
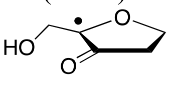 **(39bR)**	+170.58	+34.28	+118.82	−17.48	+114.80	−21.50
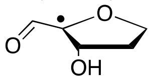 **(40R)**	+161.28	+24.98	+115.65	−20.65	+109.62	−26.68

[a] Addition of the reaction enthalpies Δ_{trh}H to the experimentally hydrogenation enthalpy of ethylene of Δ_{hyd}H (C_2H_4) = −136.3 ± 0.2 kJ·mol^{-1} yields the hydrogenation enthalpies Δ_{hyd}H of the respective double bond.

Table S40. Boltzmann-averaged reaction enthalpies <ΔH> for intramolecular redox reaction of open-shell nucleosides at various levels of theory in the gas phase (298.15 K, 1 atm, in kJ·mol^{-1}, only product radical is shown).

	UB3LYP		ROMP2		G3(MP2)-RAD	
	**<Δ_{trh}H>**	**<Δ_{trh}H + Δ_{solv}G>**	**<Δ_{trh}H>**	**<Δ_{trh}H + Δ_{solv}G>**	**<Δ_{trh}H>**	**<Δ_{trh}H + Δ_{solv}G>**
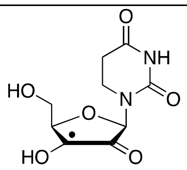 **(41aR)**	−87.53	−104.54	−68.67	−86.70	−61.14	−78.87
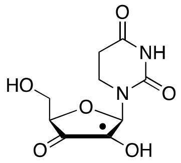 **(42aR)**	−87.36	−79.83	−60.70	−54.14	−53.75	−48.79
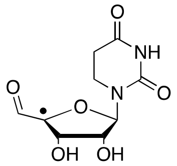 **(43aR)**	−73.07	−68.35	−54.38	−42.93	−47.83	−34.79
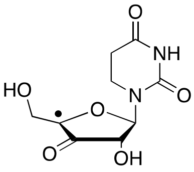 **(42bR)**	−66.54	−77.19	−46.18	−52.00	−41.85	−54.26
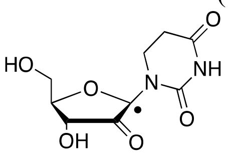 **(41bR)**	−74.49	−77.32	−58.15	−56.00	−50.44	−48.59

Table S40. *Cont.*

	UB3LYP		ROMP2		G3(MP2)-RAD	
	$\langle\Delta_{trh}H\rangle$	$\langle\Delta_{trh}H + \Delta_{solv}G\rangle$	$\langle\Delta_{trh}H\rangle$	$\langle\Delta_{trh}H + \Delta_{solv}G\rangle$	$\langle\Delta_{trh}H\rangle$	$\langle\Delta_{trh}H + \Delta_{solv}G\rangle$
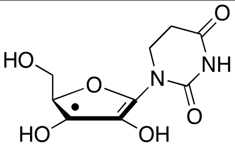 (**41cR**)	−28.55	−27.37	−25.99	−27.41	−17.35	−17.91
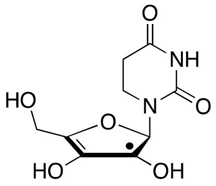 (**42cR**)	−19.56	−14.44	−16.42	−13.31	−10.14	−4.73
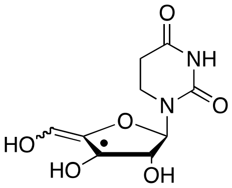 (**43bR**)	−21.75	−21.44	−22.60	−24.46	−17.13	−20.41
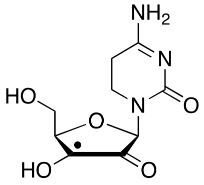 (**19R**)	−56.54	−84.69	−35.51	−63.67	−31.99	−60.23

6. Influence of Solvation Methods

In order to clarify the importance of hydrogen bonding in the covalently coupled ribonucleotides the free solvation energies of the most exothermic substrate pair **44R** and **41aR** have been calculated for all considered conformers using different PCM methods and theory.

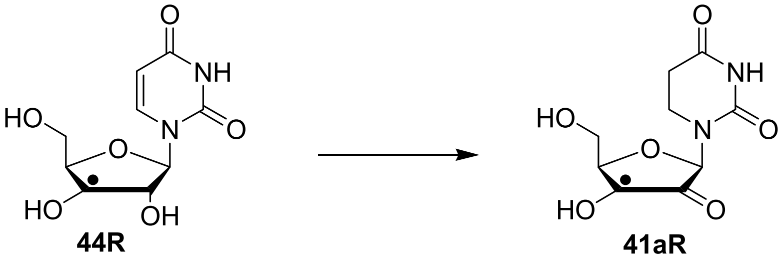

Table S41. Calculated free solvation energies ΔG_{solv} of **44R** and **41aR** in water (at 298.15 K and 1 atm, in Hartree).

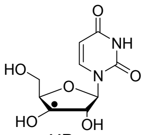 44R	**G3(MP2)-RAD**	**IEF-PCM/UAHF/ UHF/6-31G(d)**	**C-PCM/UAHF/ UHF/6-31G(d)**	**SMD/ UHF/6-31G(d)**	**SMD/ UB3LYP/ 6-31G(d)**
	**H_{298}**	**ΔG_{solv}**	**ΔG_{solv}**	**ΔG_{solv}**	**ΔG_{solv}**
007 (**A**)	−909.1510256	−0.0317446	−0.0321111	−0.0375293	−0.0269478
022 (**B**)	−909.1497844	−0.0346130	−0.0349796	−0.0407962	−0.0293860
029 (**C**)	−909.1495886	−0.0334816	−0.0338481	−0.0379756	−0.0274259
001 (**D**)	−909.1509308	−0.0326848	−0.0330194	−0.0362704	−0.0268203
016 (**E**)	−909.1487101	−0.0338003	−0.0341987	−0.0378321	−0.0269159
006 (**F**)	−909.1491248	−0.0316489	−0.0319995	−0.0392345	−0.0287008
011 (**G**)	−909.1485694	−0.0349158	−0.0353302	−0.0392345	−0.0287486
002 (**H**)	−909.1484526	−0.0351549	−0.0355692	−0.0397285	−0.0290514
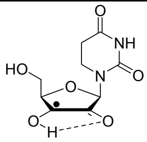 41aR	H_{298}	ΔG_{solv}	ΔG_{solv}	ΔG_{solv}	ΔG_{solv}
018 (**A**)	−909.1743372	−0.0396488	−0.0400154	−0.0443181	−0.0342465
006 (**B**)	−909.1714346	−0.0354736	−0.0358242	−0.0439994	−0.0343103
034 (**C**)	−909.1724060	−0.0389636	−0.0392982	−0.0434894	−0.0340075
040 (**D**)	−909.1706399	−0.0372106	−0.0375453	−0.0437125	−0.0349477
031 (**E**)	−909.1713281	−0.0378321	−0.0381508	−0.0429954	−0.0340234
025 (**F**)	−909.1700141	−0.0373700	−0.0377046	−0.0442384	−0.0341987
022 (**G**)	−909.1709090	−0.0385971	−0.0388998	−0.0436806	−0.0347883

Figure S3. Relative stability of **44R** in water (H + ΔG_{solv}) and gas phase (G3(MP2)-RAD, at 298.15 K and 1 atm) and their graphical representations (conformer denotation in parenthesis).

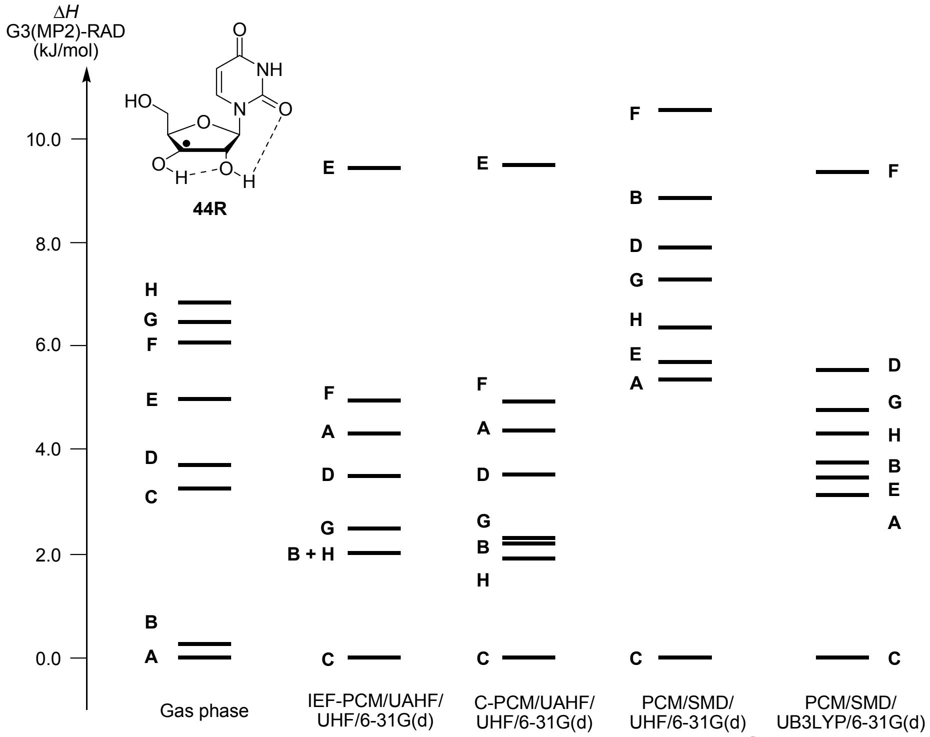

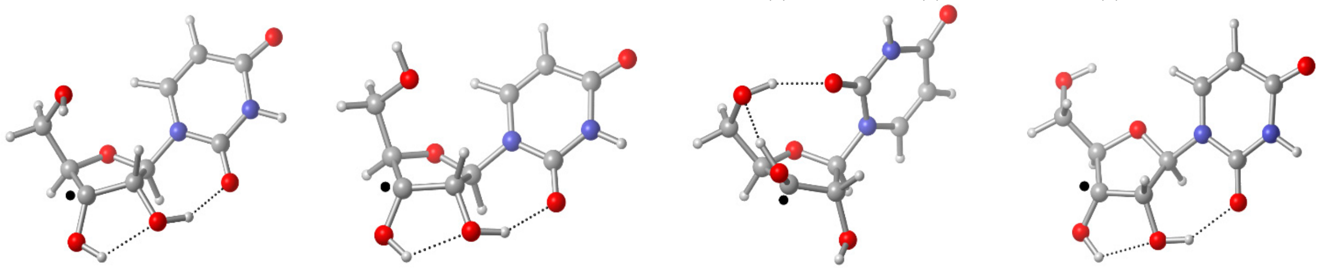

A (007) **B** (001) **C** (022) **D** (029)

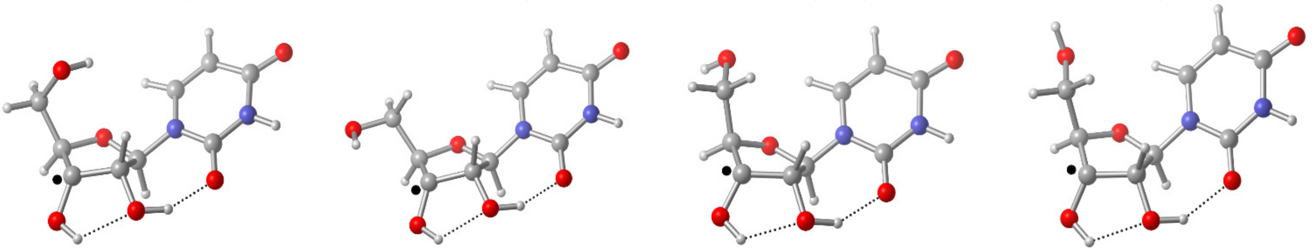

E (006) **F** (016) **G** (011) **H** (002)

Figure S4. Relative stability of **41aR** in water (H + ΔG$_{solv}$) and gas phase (G3(MP2)-RAD, at 298.15 K and 1 atm) and their graphical representations (conformer denotation in parenthesis).

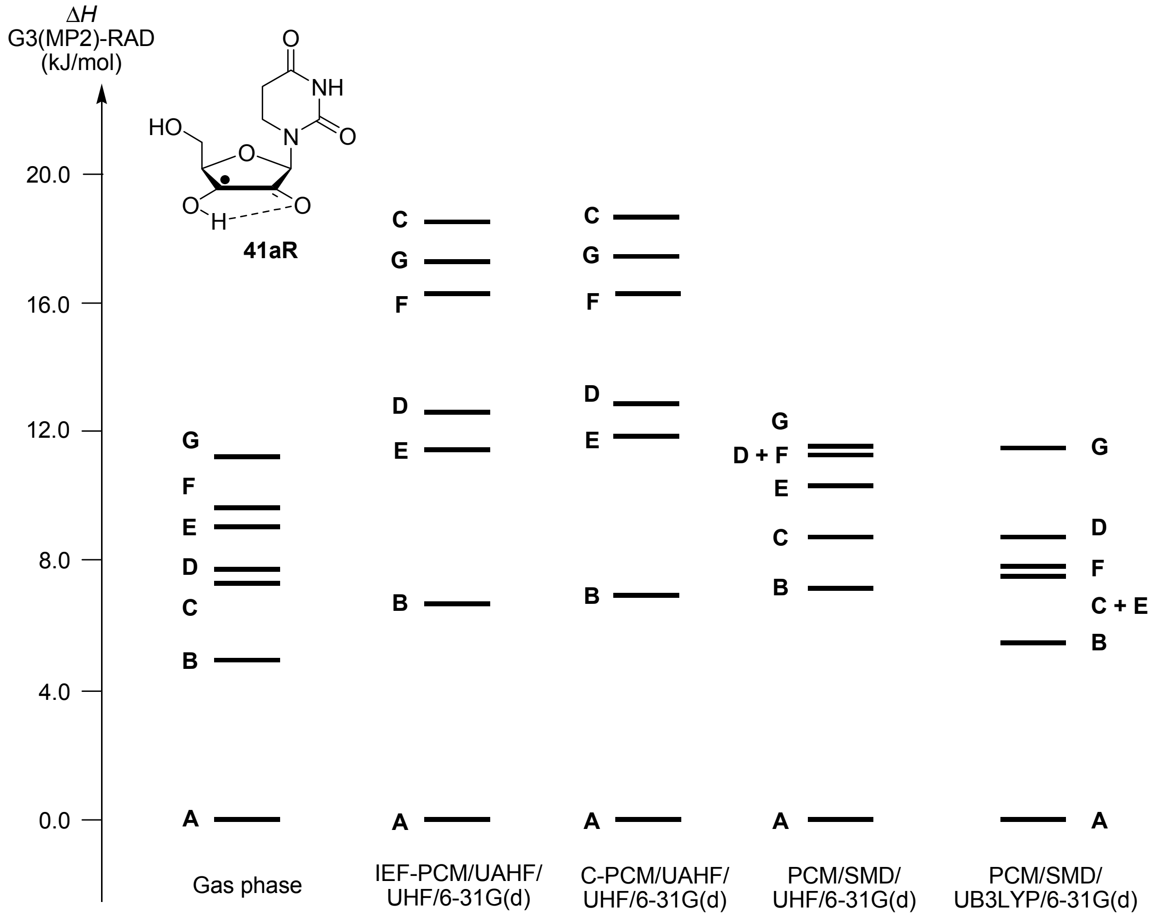

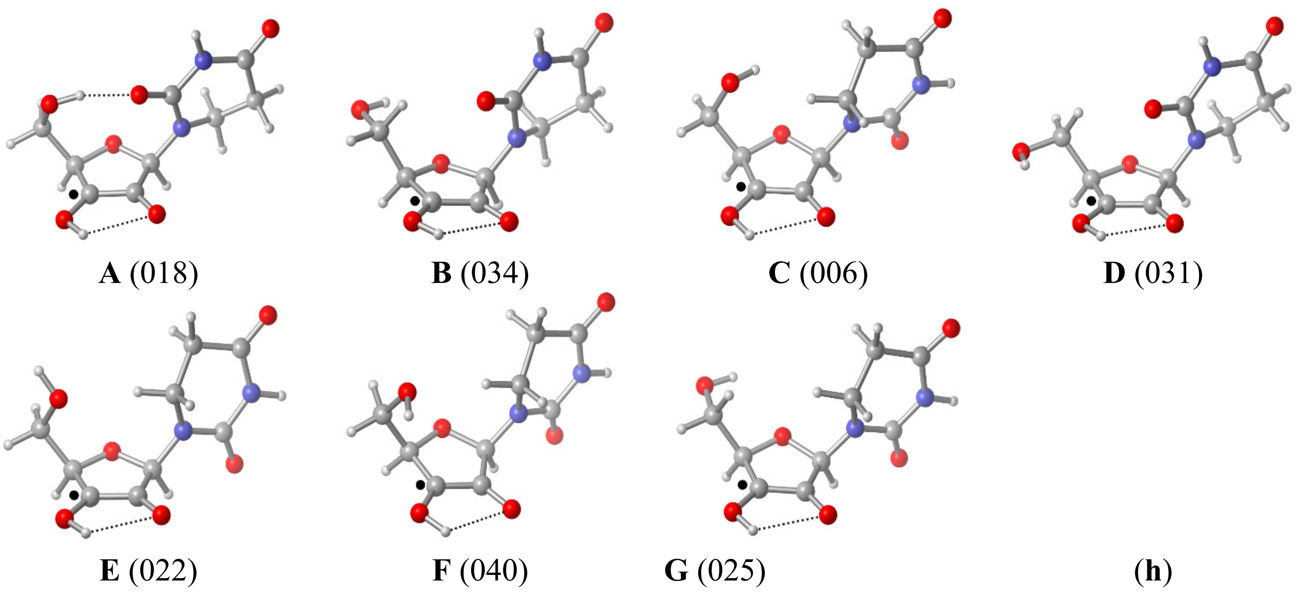

Table S42. Boltzmann-averaged enthalpies ΔH_{sol} of 44R and 41aR including implicit solvation $<\Delta H_{298} + \Delta G_{solv}>$ at G3(MP2)-RAD level of theory (298.15 K, 1 atmin Hartree).

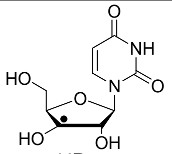 44R	Gas Phase	IEF-PCM/UAHF/ UHF/6-31G(d)	C-PCM/UAHF/ UHF/6-31G(d)	SMD/ UHF/6-31G(d)	SMD/ UB3LYP/ 6-31G(d)
**<H>**	**−909.1504578**	**−909.1836984**	**−909.1840667**	**−909.1898126**	**−909.1783504**
007 (**A**)	−909.1510256	−909.1827702	−909.1831367	−909.1885549	−909.1779734
022 (**B**)	−909.1497844	−909.1843974	−909.1847640	−909.1905806	−909.1791704
029 (**C**)	−909.1495886	−909.1830702	−909.1834367	−909.1875642	−909.1770145
001 (**D**)	−909.1509308	−909.1836156	−909.1839502	−909.1872012	−909.1777511
016 (**E**)	−909.1487101	−909.1825104	−909.1829088	−909.1865422	−909.1756260
006 (**F**)	−909.1491248	−909.1807737	−909.1811243	−909.1883593	−909.1778256
011 (**G**)	−909.1485694	−909.1834852	−909.1838996	−909.1878039	−909.1773180
002 (**H**)	−909.1484526	−909.1836075	−909.1840218	−909.1881811	−909.1775040
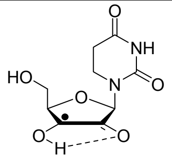 41aR	**Gas phase**	**IEF-PCM/UAHF/ UHF/6-31G(d)**	**C-PCM/UAHF/ UHF/6-31G(d)**	**SMD/ UHF/6-31G(d)**	**SMD/ UB3LYP/ 6-31G(d)**
**<H>**	**−909.1737467**	**−909.2137393**	**−909.2141520**	**−909.2182765**	**−909.2080023**
018 (**A**)	−909.1743372	−909.2139860	−909.2143526	−909.2186553	−909.2085837
006 (**B**)	−909.1714346	−909.2069082	−909.2072588	−909.2154340	−909.2057449
034 (**C**)	−909.1724060	−909.2113696	−909.2117042	−909.2158954	−909.2064135
040 (**D**)	−909.1706399	−909.2078505	−909.2081852	−909.2143524	−909.2055876
031 (**E**)	−909.1713281	−909.2091602	−909.2094789	−909.2143235	−909.2053515
025 (**F**)	−909.1700141	−909.2073841	−909.2077187	−909.2142525	−909.2042128
022 (**G**)	−909.1709090	−909.2095061	−909.2098088	−909.2145896	−909.2056973

Table S43. Boltzmann-averaged $<\Delta_{trh}H>$ at G3(MP2)-RAD level with and without implicit solvation (in kJ·mol^{-1}).

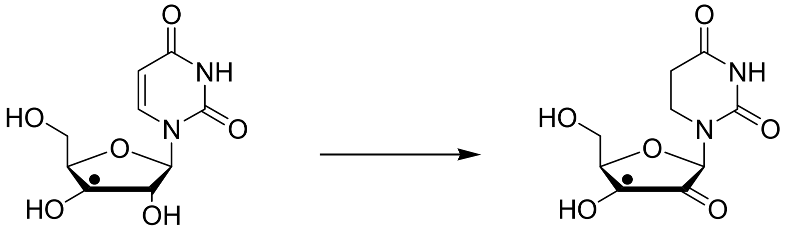

44R → 41aR

**Method**	**$<\Delta_{trh}H>$**
Gas phase	−61.14
IEF-PCM/UAHF/ UHF/6-31G(d)	−78.87
C-PCM/UAHF/ UHF/6-31G(d)	−78.99
SMD/ UHF/6-31G(d)	−74.73
SMD/ UB3LYP/6-31G(d)	−77.85

7. Structures of all Stationary Points (Optimized at UB3LYP/6-31G(d) Level of Theory)

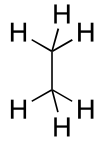

1\1\GINC-GOLEM\SP\RMP2-FC\6-311+G(3df,2p)\C2H6\ZIPSE\27-Jun-2008\0\\#p MP2(FC)/6-311+G(3df,2p) scf = tight geom = check guess = read\\etha_2b etha ne opt + freq\\0,1\C,0,0.,0.,-0.0015888318\C,0,0.,0.,1.5289641618\H,0, 1.0209278182,0.,-0.4006534375\H,0,-0.5104639091,-0.884149426,-0.400653 4375\H,0,-0.5104639091,0.884149426,-0.4006534375\H,0,-1.0209278182,0.,1.9280287675\H,0,0.5104639091,-0.884149426,1.9280287675\H,0,0.51046390 91,0.884149426,1.9280287675\\Version=AM64L-G03RevD.01\State=1-A1G\HF=-79.2572663\MP2=79.6200629\ RMSD=1.021e-09\Thermal=0.\PG=D03D [C3(C1.C1),3SGD(H2)]\\@

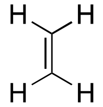

1\1\GINC-GOLEM\SP\RMP2-FC\6-311+G(3df,2p)\C2H4\ZIPSE\27-Jun-2008\0\\#p MP2(FC)/6-311+G(3df,2p) scf = tight geom = check guess = read\\ethl_2b ethy lene opt + freq\\0,1\C,0,0.6654676409,0.,1.\C,0,-0.6654676409,0.,1.\H, 0,1.2395972555,-0.923582982,1.\ H,0,1.2395972555,0.923582982,1.\H,0,-1. 2395972555,0.923582982,1.\H,0,-1.2395972555,-0.923582982,1.\\Version=A M64L-G03RevD.01\State=1-AG\HF=-78.0619904\MP2=-78.3932565\ RMSD=3.743e-09\Thermal=0.\PG=D02H [C2"(C1.C1),SG(H4)]\\@

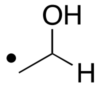

1\1\GINC-IBLIS\SP\ROMP2-FC\GTMP2large\C2H5O1(2)\FLORIAN\28-Jan-2013\0\ \#p ROMP2(FC)/ GTMP2large geom = check guess = read scf = tight\\RadEtOH\\0,2 \C,0,-2.563248188, -0.3135055054,1.2059933284\H,0,-2.8702776064,-0.9360 82036,2.0639897991\C,0,-1.0791148694, -0.2470170963,1.1222383597\H,0,-0.6148569062,0.5176508866,0.5064327538\H,0,-0.455690429,-1.0410989576,1 .5209790557\O,0,-3.1765116764,0.9746473746,1.2510078165\H,0,-2.7665730 963,1.4531466104,1.9889840025\H,0,-2.9931743982,-0.7918846764,0.310973 7443\\Version = AM64L-G03RevD.01\State = 2-A\HF = -153.5001952\MP2 = -154.0598 412\RMSD = 8.143e-09\Thermal=0.\PG=C01 [X(C2H5O1)]\\@

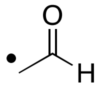

1\1\GINC-IBLIS\SP\ROMP2-FC\GTMP2large\C2H3O1(2)\FLORIAN\28-Jan-2013\0\ \#p ROMP2(FC)/ GTMP2large geom = check guess = read scf = tight\\RadEthanal\\ 0,2\C,0,-0.205669768,-0.6381018947, 2.4865123122\H,0,-0.5624965695,-1.6 627856515,2.4446457123\H,0,-0.0502256039,-0.1699498311, 3.4535627823\C, 0,0.0632570761,0.1046263822,1.2995822685\O,0,0.4717419386,1.2741060372, 1.313107886\H,0,-0.1111938733,-0.4161291022,0.3373031486\\Version = AM6 4L-G03RevD.01\State = 2-A\HF = -152.2969665\MP2=-152.8124586\RMSD=5.102e-0 9\Thermal=0.\PG=C01 [X(C2H3O1)]\\@

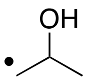

1\1\GINC-IBLIS\SP\ROMP2-FC\GTMP2large\C3H7O1(2)\FLORIAN\28-Jan-2013\0\ \#p ROMP2(FC)/ GTMP2large geom = check guess = read scf = tight\\RadIsoPro\\0,2\C,0,-3.0767671824,-0.9909101483, -0.0680920063\H,0,-2.7133837128,-2. 0197773598,-0.1555778412\H,0,-2.7676956159,-0.4300396524, -0.9560012026\H,0,-4.1715494129,-1.0035303365,-0.0281235119\C,0,-2.5214557007,-0.32 20423711, 1.1964828053\H,0,-2.8403236371,-0.9204250315,2.0677231778\C,0 ,-1.0327025399,-0.2184743414, 1.195393198\H,0,-0.5553847079,0.528180247 ,0.56641303\H,0,-0.4114641393,-0.9794715497, 1.657436988\O,0,-3.1302073 32,0.9760415401,1.2478911448\H,0,-2.7219365492,1.4501852036, 1.98964772 79\\Version=AM64L-G03RevD.01\State=2-A\HF=-192.5514408\MP2=-193.283241 8\RMSD= 3.737e-09\Thermal=0.\PG=C01 [X(C3H7O1)]\\@

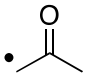

1\1\GINC-IBLIS\SP\ROMP2-FC\GTMP2large\C3H5O1(2)\FLORIAN\28-Jan-2013\0\\#p ROMP2(FC)/ GTMP2large geom=check guess=read scf=tight\\RadAceton\\0,2\C,0,-0.4708362557,-0.6509334584, -0.0102072299\H,0,0.0056173178,-1.6 329236832,-0.1194209972\H,0,-0.2148220639,-0.0283225062, -0.8696131933\H,0,-1.5551239291,-0.8173077379,0.004702506\C,0,-0.2373208041,-0.62819 79144, 2.5236454108\H,0,-0.7210815585,-1.5986951354,2.5875432253\H,0,0.0866594313,-0.1414295953, 3.4373973253\C,0,-0.0113002218,0.0377815302,1.2674730862\O,0,0.543878584,1.1451217706, 1.2385025168\\Version = AM64L-G 03RevD.01\State=2-A\HF=-191.3963355\MP2=-192.0980503\RMSD= 9.354e-09\Thermal=0.\PG=C01 [X(C3H5O1)]\\@

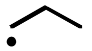

1\1\GINC-IBLIS\SP\ROMP2-FC\6-311+G(3df,2p)\C3H7(2)\FLORIAN\29-Apr-2013\0\\#p ROMP2(FC)/ 6-311+G(3df,2p) geom = check guess = read scf = tight\\RadE tOH\\0,2\C,0,-1.6521729288,0.3597831102, -0.1020612052\H,0,-1.297095855 2,-0.6735459778,-0.1864396019\H,0,-1.2396593413,0.931247481, -0.9427774 706\H,0,-2.7433766994,0.3469094439,-0.2024456966\C,0,-1.2270820247,0.9 834565185, 1.2493039846\H,0,-1.5681655672,2.0278452837,1.2724267101\H,0 ,-0.1292443732,1.0125826258, 1.2883513551\C,0,-1.7594274413,0.247554883 1,2.434237919\H,0,-1.2446514214,-0.6257908589, 2.8245823274\H,0,-2.7604 57678,0.4437197546,2.8078067899\\Version = AM64L-G03RevD.01\State = 2- A'\H F = -117.6670955\MP2 = -118.1690627\RMSD = 3.679e-09\Thermal = 0.\PG = CS [SG(C3 H1),X(H6)]\\@

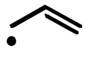

1\1\GINC-IBLIS\SP\ROMP2-FC\6-311+G(3df,2p)\C3H5(2)\FLORIAN\29-Apr-2013 \0\\#p ROMP2(FC)/ 6-311+G(3df,2p) geom = check guess = read scf = tight\\RadE tOH\\0,2\C,0,-0.7108880263, 0.4364532337,0.3305525944\H,0,-0.289078329, 1.0012366356,-0.494071225\H,0,-0.4279279368, -0.6099483758,0.4119072587 \C,0,-1.5747034582,1.0183892836,1.2452814771\H,0,-1.817883065, 2.072504 3349,1.1064356208\C,0,-2.1533000448,0.3656746369,2.3221033106\H,0,-1.9 524467484,- 0.684684449,2.5167502454\H,0,-2.8233404566,0.8777165213,3.0 046071289\\Version = AM64L- G03RevD.01\State = 2-A\HF=-116.4846099\MP2=-116 .9739045\RMSD=3.769e-09\Thermal=0.\PG=C01 [X(C3H5)]\\@

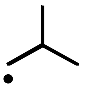

1\1\GINC-IBLIS\SP\ROMP2-FC\6-311+G(3df,2p)\C4H9(2)\FLORIAN\02-May-2013 \0\\#p ROMP2(FC)/ 6-311 + G(3df,2p) geom = check guess = read scf = tight\\Isob utyl\\0,2\C,0,1.1862501653,0.171465072, -0.4031473011\H,0,2.1023558961, -0.3698302561,-0.622468346\H,0,1.1581243425,1.2302075738, -0.6452760436 \C,0,0.096092473,-0.460290234,0.4029969337\C,0,-1.2649404213,0.2093897 297, 0.1408954403\H,0,-1.2194287399,1.2880518442,0.3318363882\H,0,-1.57 37735421,0.0660715725, -0.9016305413\C,0,0.0266198484,-1.9805332924,0.1 715365814\H,0,-0.7278701913,-2.4433134487, 0.8183762411\H,0,-0.23646571 48,-2.2030936782,-0.8696416354\H,0,0.9904017744,-2.4583930871, 0.383250 671\H,0,0.3266215237,-0.30932043,1.476593811\H,0,-2.043142194,-0.21422 78058, 0.7864942306\\Version = AM64L-G03RevD.01\State = 2-A\HF = -156.7146276 \MP2 = -157.3910101\ RMSD=8.755e-09\Thermal=0.\PG=C01 [X(C4H9)]\\@

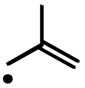

1\1\GINC-IBLIS\SP\ROMP2-FC\6-311+G(3df,2p)\C4H7(2)\FLORIAN\02-May-2013 \0\\#p ROMP2(FC)/ 6-311+G(3df,2p) geom = check guess = read scf = tight\\MeAl lyl\\0,2\C,0,1.2124199208,0.2124839094, -0.0034814793\H,0,2.1616877892, -0.314107824,-0.0191752082\H,0,1.2473203213,1.2978564518, 0.0183595433\C,0,-0.0001209682,-0.4674610541,-0.0097921493\C,0,-1.2124117098,0.2126 702493, -0.003573494\H,0,-1.2470965796,1.2980566443,0.0182328536\H,0,-2 .1617907588,-0.3137070923, -0.0193133267\C,0,-0.0000960358,-1.986682819 3,-0.0017661646\H,0,0.0053865563,-2.3717837019, 1.0257981029\H,0,-0.888 6495322,-2.3902465161,-0.4979968607\H,0,0.8833011467,-2.3901717972, -0.507243327\\Version = AM64L-G03RevD.01\State = 2-A\HF = -155.5327637\MP2 = -156.1966271\ RMSD=8.106e-09\Thermal=0.\PG=C01 [X(C4H7)]\\@

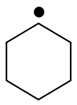

1\1\GINC-IBLIS\SP\ROMP2-FC\6-311+G(3df,2p)\C6H11(2)\FLORIAN\02-May-201 3\0\\#p ROMP2(FC)/ 6-311+G(3df,2p) geom = check guess = read scf = tight\\Cyc lohexyl\\0,2\C,0,-1.8009205888, -2.3857371903,-0.0341517207\C,0,-0.2658 278741,-2.4329812855,-0.0037876434\C,0,0.3408029601, -1.0138334743,0.04 09213384\C,0,-0.300323249,-0.1635528396,1.0932248977\C,0,-1.7903845654 , -0.1816296112,1.2372797107\C,0,-2.3597133162,-1.6153439741,1.17162993 57\H,0,1.426976124, -1.0658490357,0.1889934571\H,0,0.0577436056,-2.9891 492849,0.887109338\H,0,0.1190067863, -2.9774170685,-0.8755013319\H,0,-2 .1323929133,-1.8982286549,-0.9637533277\H,0,-2.2096063255, -3.404306934 5,-0.0536516617\H,0,-2.0988168005,0.3109312931,2.1682235792\H,0,-2.254 1412552, 0.4070301597,0.4201222327\H,0,-2.0915217242,-2.1498875075,2.09 361424\H,0,-3.4558965312, -1.5814586978,1.1312953501\H,0,0.1971654936,- 0.550175744,-0.9559370328\ H,0,0.2720728338,0.6368418202,1.5561222887\\Version = AM64L-G03RevD.01\State = 2-A\HF = -233.6463017\MP2=-234.6443077\RM SD=3.776e-09\Thermal=0.\PG=C01 [X(C6H11)]\\@

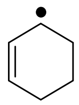

1\1\GINC-IBLIS\SP\ROMP2-FC\6-311+G(3df,2p)\C6H9(2)\FLORIAN\02-May-2013\0\\#p ROMP2(FC)/ 6-311+G(3df,2p) geom = check guess = read scf = tight\\CyAl lyl\\0,2\C,0,-1.7447219217,-2.4682143987, -0.1332616975\C,0,-0.22864856 97,-2.4573382795,0.1344352445\C,0,0.3632062833,-1.0419503232, 0.0029712 975\C,0,-0.4362033874,-0.035895306,0.7851945975\C,0,-1.776023221,-0.26 04582705, 1.0783767056\C,0,-2.45086216,-1.3992028015,0.6553249474\H,0,1 .4109555969,-1.0390205398, 0.3322896764\H,0,-0.0509697335,-2.8175774968 ,1.1559961122\H,0,0.2837670274,-3.1497995631, -0.5440452777\H,0,-1.9295 41353,-2.3185898344,-1.2115692466\H,0,-2.162315039,-3.4567302296, 0.100 7831418\H,0,-2.3215177882,0.4916238973,1.6464641881\H,0,-3.5096012056, -1.5179491711, 0.8713458442\H,0,0.3885797717,-0.7506185869,-1.061778045 4\H,0,0.0446612297,0.8858152336, 1.1027491621\\Version = AM64L-G03RevD.01 \State = 2-A\HF = -232.4679565\MP2 = -233.4544966\ RMSD = 4.523e-09\Thermal = 0\PG=C01 [X(C6H9)]\\@

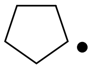

1\1\GINC-NODE3\SP\ROMP2-FC\6-311+G(3df,2p)\C5H9(2)\ZIP06\30-Jun-2014\0\\#p ROMP2(FC)/6-311+G(3df,2p) scf=tight geom=check guess=read\\b3lyp/ 6-31G(d)\\0,2\C,0,-1.3560973771,-0.2515697767,-0.1084782509\C,0,0.1802 586402,-0.2298308421,-0.2969719837\C,0,0.6173623528,1.1092891539,0.329 7846117\C,0,-0.5254956537,2.0878363453,-0.034513689\C,0,-1.7315227526, 1.2005196057,-0.1233818153\ H,0,-1.8707441137,-0.8432113878,-0.87950663 41\H,0,-1.6194031512,-0.7256683842,0.8549607695\ H,0,0.4176772797,-0.23 13240018,-1.3687114795\H,0,0.6808219036,-1.095431988,0.1501147652\H,0, 0.6761448843,1.0050021394,1.4209602252\H,0,1.5993837002,1.4454256194,- 0.0200179543\H,0, -0.6362917958,2.9028378317,0.6954744062\H,0,-0.316571 0799,2.5813557161,-1.0015210931\H,0, -2.7517533831,1.5648671653,-0.1903 272716\\Version = AM64L-G03RevD.01\State = 2-B\HF = -194.593948\MP2=-195.419 0525\RMSD=5.613e-09\Thermal=0.\PG=C02 [C2(C1H1),X(C4H8)]\\@

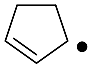

1\1\GINC-NODE8\SP\ROMP2-FC\6-311+G(3df,2p)\C5H7(2)\ZIP06\30-Jun-2014\0 \\#p ROMP2(FC)/ 6-311+G(3df,2p) scf = tight geom = check guess = read\\b3lyp/6-31G(d)\\0,2\C,0,-1.2779074295, -0.1902315324,-0.3472975027\C,0,0.0479 639511,-0.2829748015,0.0587488547\C,0,0.605419119, 1.0895892433,0.34548 47273\C,0,-0.5880804761,2.0524087189,0.052299128\C,0,-1.7059036234,1.1 317946209,-0.3717610828\H,0,-1.8994890673,-1.0412294607,-0.61148777\H, 0,0.6179255426,- 1.1999214969,0.1613211308\H,0,0.9563333685,1.178594502 4,1.3838825286\H,0,1.4774988449, 1.3162017265,-0.2849205576\H,0,-0.8611 180225,2.6458443669,0.9369127467\H,0,-0.3407362677, 2.7822665255,-0.732 2379058\H,0,-2.6963560395,1.473461197,-0.6518565172\\Version = AM64L-G03 RevD.01\State = 2-A\HF = -193.4137872\MP2 = -194.2269215\RMSD=3.098e-09\Ther mal = 0.\PG = C01 [X(C5H7)]\\@

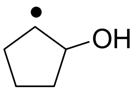

1\1\GINC-NODE21\SP\ROMP2-FC\6-311+G(3df,2p)\C5H9O1(2)\ZIP06\04-Jul-201 4\0\\#p ROMP2(FC)/ 6-311+G(3df,2p) scf = tight geom = check guess = read\\b3l yp/6-31G(d)\\0,2\C,0,-0.9487826698,-0.8191538408, -0.7941513369\C,0,0.5 065653878,-0.7400077266,-0.4522042861\C,0,1.0229858587,0.6646358491, -0.5222417937\C,0,-0.146359169,1.4484064507,-1.1671310857\C,0,-1.3963662 271,0.6587640754, -0.7344899509\H,0,-1.5132852852,-1.4503267727,-0.0884 91799\H,0,1.1233365899,-1.6168276907, -0.285026158\H,0,1.2471905684,1.0 588755732,0.4856216001\H,0,1.9599957381,0.7496041621, -1.0900521918\H,0,-0.1775555998,2.5009102463,-0.8675427632\H,0,-0.0523406141,1.41103810 49, -2.2580523579\H,0,-1.6648224449,0.9131121857,0.3003059491\H,0,-2.27 44092089,0.8523886604,-1.3616068419\O,0,-1.0716823291,-1.37602717,-2.1 211513769\H,0,-2.021116945,-1.4900713568,-2.2926907273\\Version = AM64L- G03RevD.01\State=2-A\HF=-269.478688\MP2=-270.5398569\ RMSD=7.162e-09\Thermal=0.\PG=C01 [X(C5H9O1)]\\@

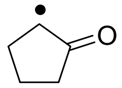

1\1\GINC-NODE21\SP\ROMP2-FC\6-311+G(3df,2p)\C5H7O1(2)\ZIP06\04-Jul-201 4\0\\#p ROMP2(FC)/ 6-311+G(3df,2p) scf = tight geom = check guess = read\\b3l yp/6-31G(d)\\0,2\C,0,-0.9038799588,-0.6689565128, -1.3558402545\C,0,0.4 777530153,-0.730995197,-0.9445029525\C,0,0.9674955355,0.5908265869,-0. 4604356723\C,0,-0.0999533199,1.5997141909,-0.9756463278\C,0,-1.3914963 92,0.7730535308,-1.1390389103\H,0,1.052357726,-1.6516357497,-0.9505655 779\H,0,1.0176409759,0.5960355254, 0.6427325325\H,0,1.9835946745,0.8255 892884,-0.8034938192\H,0,-0.2177511632,2.4571929004,-0.3070140491\H,0, 0.2162726565,1.9879656784,-1.9504364587\H,0,-2.0040721106,0.7945451702 , -0.2267408907\H,0,-2.0325865636,1.0997252025,-1.9629700526\O,0,-1.583 7528556,-1.6065253443, -1.7794523568\\Version=AM64L-G03RevD.01\State = 2- A\HF = -268.3273564\MP2 = -269.3582119\ RMSD=3.389e-09\Thermal=0.\PG=C01 [X (C5H7O1)]\\@

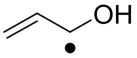

1\1\GINC-NODE7\SP\ROMP2-FC\6-311+G(3df,2p)\C3H5O1(2)\ZIP06\30-Jun-2014 \0\\#p ROMP2(FC)/ 6-311+G(3df,2p) scf = tight geom = check guess = read\\b3ly p/6-31G(d)\\0,2\C,0,-3.452788162, -0.2238563773,-1.6209436833\H,0,-3.20 29999358,-1.273838642,-1.4942069546\H,0,-4.4662560282, 0.0762562349,-1. 3808211218\C,0,-2.5212331824,0.6933516832,-2.0697299953\H,0,-2.8161136 501, 1.7363766943,-2.1816478145\C,0,-1.2085403231,0.3975118801,-2.39977 80886\H,0,-0.7980495773, -0.6073374579,-2.3299192061\O,0,-0.3744839453, 1.3909827145,-2.8353027711\H,0,0.4971059942, 1.0139188502,-3.0206180246 \\Version = AM64L-G03RevD.01\State = 2-A\HF -191.3721985\MP2 = -192.0973633\RMSD = 8.225e-09\Thermal = 0.\PG=C01 [X(C3H5O1)]\\@

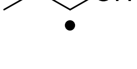

001

1\1\GINC-NODE4\SP\ROMP2-FC\6-311+G(3df,2p)\C3H7O1(2)\ZIP06\01-Jul-2014 \0\\#p ROMP2(FC)/ 6-311+G(3df,2p) scf = tight geom = check guess = read\\b3ly p/6-31G(d)\\0,2\C,0,

-1.9369620205,0.161547466,-0.0695330093\H,0,-2.033 4787126,0.8261191923,-0.9352328341\H,0, -2.746674952,-0.5745548693,-0. 12861503\C,0,-0.5672284028,-0.5278063099,-0.0533774689\H,0, -0.44297801 94,-1.1439802901,-0.9551387472\C,0,0.5582412837,0.4477864668,0.0161332 154\ H,0,0.4997989585,1.2989568583,0.6990418932\O,0,1.8057071796,-0.108 4322392,-0.1655947388\ H,0,2.4745181393,0.5831165629,-0.0514312851\H,0, -2.0872354543,0.7665109358,0.8331719847\H,0, -0.5228722496,-1.236094523 6,0.795299533\\Version = AM64L-G03RevD.01\State = 2-A\HF = - 192.5581661\MP2 = -193.2966386\RMSD = 4.003e-09\Thermal = 0.\PG = C01 [X(C3H7O1)]\\@

002

1\1\GINC-NODE8\SP\ROMP2-FC\6-311+G(3df,2p)\C3H7O1(2)\ZIP06\01-Jul-2014 \0\\#p ROMP2(FC)/ 6-311 + G(3df,2p) scf = tight geom = check guess = read\\b3ly p/6-31G(d)\\0,2\C,0,-1.5722971973, -0.5546333993,-0.0646953005\H,0,-1.1 722716344,-1.3635754692,0.5542233091\H,0,-2.6082833213, -0.3705046189,0 .24135273\C,0,-0.7219879379,0.7180964873,0.0877926935\H,0,-0.802635767 7,1.0724985331,1.1325232511\C,0,0.7099588045,0.5281819513,-0.285714516 7\H,0,1.3160536425, 1.3818247893,-0.5947714377\O,0,1.3607800245,-0.4529 504064,0.4321557934\H,0,2.2837850108,- 0.4916812128,0.1409002354\H,0,-1 .1309800433,1.5209243503,-0.5386684712\H,0,-1.5767790605,- 0.8988096147 ,-1.1049365765\\Version=AM64L-G03RevD.01\State=2-A\HF=-192.5577533\MP2 =- 193.2968177\RMSD=6.821e-09\Thermal=0.\PG=C01 [X(C3H7O1)]\\@

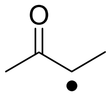

001

1\1\GINC-NODE4\SP\ROMP2-FC\6-311+G(3df,2p)\C4H7O1(2)\ZIP06\01-Jul-2014 \0\\#p ROMP2(FC)/ 6-311+G(3df,2p) scf = tight geom = check guess = read\\b3ly p/6-31G(d)\\0,2\C,0,-2.0079182799, -0.0015138415,0.4929020864\H,0,-2.69 5613479,-0.5741197004,-0.1471783209\H,0,-2.4380570263,- 0.0315866116,1.5047595948\C,0,-0.6442615938,-0.5912155473,0.4730280875\H,0,-0.5091195 838, -1.6203087489,0.8023148165\C,0,0.5115106946,0.1500300409,0.0250203 891\H,0,-1.9775427553, 1.0339468588,0.1466391527\O,0,0.4097993407,1.324 9288152,-0.3586777059\C,0,1.8593190843, -0.5592045407,0.0171750766\H,0, 2.6569603609,0.1803906007,-0.0793390324\H,0,2.0146530056, -1.1525221129,0.9254175322\H,0,1.9194146219,-1.2468510222,-0.8365887268\\Version = AM 64L- G03RevD.01\State = 2-A\HF = -230.4513834\MP2 = -231.3339692\RMSD = 4.472e- 09\Thermal = 0.\PG = C01 [X(C4H7O1)]\\@

002

1\1\GINC-NODE4\SP\ROMP2-FC\6-311+G(3df,2p)\C4H7O1(2)\ZIP06\01-Jul-2014 \0\\#p ROMP2(FC)/ 6-311 + G(3df,2p) scf = tight geom = check guess = read\\b3ly p/6-31G(d)\\0,2\C,0,-0.9838525546, -0.9147823513,1.5873387255\H,0,-2.03 73806155,-0.7373328108,1.850887432\H,0,-0.8711529533, -2.0074341098,1.5 220778706\C,0,-0.6472099134,-0.2692618398,0.2878681314\H,0,-1.21242534 7, -0.5603026813,-0.5950846217\C,0,0.3679531587,0.7252984822,0.04994343 18\H,0,-0.3635039734,- 0.5591673122,2.4140601001\O,0,0.5304376447,1.171 4223306,-1.0974994945\C,0,1.2333393378,

1.222775931,1.2015160185\H,0,1.9329566604,1.9661781749,0.815067296\H,0,0.6257102552,1.6808798704,1.99 16037237\H,0,1.8002253405,0.4035723563,1.6607167567\\Version=AM64L-G03 RevD.01\State = 2-A\HF = -230.4482656\MP2 = -231.3316005\RMSD = 6.186e-09\Ther mal = 0.\PG = C01 [X(C4H7O1)]\\@

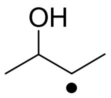

001

1\1\GINC-NODE4\SP\ROMP2-FC\6-311+G(3df,2p)\C4H9O1(2)\ZIP06\01-Jul-2014 \0\\#p ROMP2(FC)/ 6-311+G(3df,2p) scf = tight geom = check guess = read\\b3ly p/6-31G(d)\\0,2\C,0,-2.0289268185, -0.1239613959,0.0230741661\H,0,-2.79 32281775,-0.7929612488,-0.3890403859\H,0,-2.3069286711, 0.086945209,1.0 688148099\C,0,-0.6644536497,-0.7167901036,-0.0708495724\H,0,-0.5461130 073, -1.7970436967,-0.0305779082\C,0,0.5546603508,0.1436589477,-0.00080 3948\H,0,-2.0872734851, 0.8363939448,-0.5067373383\O,0,0.4607618518,0.9 402971621,1.2002429093\C,0,1.8546422037, -0.6625169114,-0.0214829456\H, 0,2.7284335455,-0.0006461583,0.0154658736\H,0,1.8961685942, -1.33074827 27,0.8454154459\H,0,1.9279971886,-1.2659672075,-0.9340016562\H,0,0.547 7450038, 0.8315733752,-0.869149151\H,0,1.1475679308,1.6243871162,1.1437 547608\\Version=AM64L-G03RevD.01\State=2-A\HF=-231.6019154\MP2=-232.51 321\RMSD=6.960e-09\Thermal=0.\PG=C01 [X(C4H9O1)]\\@

002

1\1\GINC-NODE7\SP\ROMP2-FC\6-311+G(3df,2p)\C4H9O1(2)\ZIP06\01-Jul-2014\0\\#p ROMP2(FC)/ 6-311+G(3df,2p) scf = tight geom = check guess = read\\b3ly p/6-31G(d)\\0,2\C,0,0.9716007415, -1.4158526213,-1.7481978026\H,0,2.021 4766571,-1.3362832864,-1.411526402\H,0,0.9049040631, -2.3401844507,-2.3 342896526\C,0,0.0024138812,-1.4170877939,-0.6151798412\H,0,-0.20684821 26,-2.3392799812,-0.0790008299\C,0,-0.4764691989,-0.1517513544,0.01749 46641\H,0,0.7988372872,-0.571079696,-2.4286812137\O,0,-1.6584803626,-0.469446826,0.7578154912\C,0,0.582640587, 0.4894499739,0.937800818\H,0,0 .2050861613,1.425150261,1.3736708575\H,0,0.8352749774,-0.1962384609,1. 7534119401\H,0,1.4971199391,0.7285217614,0.3833279865\H,0,-0.712877218 3,0.5771239621,-0.7813863906\H,0,-1.9243185424,0.3303817725,1.23769769 52\\Version = AM64L-G03RevD.01\State = 2-A\HF = -231.6018579\MP2 = -232.512916 9\RMSD = 3.814e-09\Thermal = 0.\PG = C01 [X(C4H9O1)]\\@

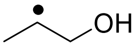

001

1\1\GINC-ANGIE\SP\ROMP2-FC\6-311+G(3df,2p)\C3H7O1(2)\FLORIAN\04-Jul-20 14\0\\#P ROMP2(FC)/ 6-311+G(3df,2p) geom = check guess = read scf = tight\\Ub 3LYP\\0,2\C,0,-1.9090751884,0.1610420216, -0.0728273397\H,0,-2.25778160 92,0.667945836,-0.9914959542\H,0,-2.6252459518,-0.6431958301, 0.1303567 964\C,0,-0.5194624223,-0.363718206,-0.2021528841\H,0,-0.3461521046,-1. 3449470909, -0.6363938329\C,0,0.6620784742,0.5319623128,-0.0822880446\H,0,0.5196126225,1.2297256917, 0.7646953336\O,0,1.8324381784,-0.2633044732,0.0872424717\H,0,2.5993901095,0.3259154862,

0.0281157928\H,0,-1.9904 562038,0.8994394439,0.7367465716\H,0,0.7586961356,1.1714412179,-0.9866 777307\\Version = AM64L-G03RevD.01\State = 2-A\HF = -192.5512766\MP2 = -193.28 77564\RMSD = 4.659e-09\Thermal = 0.\PG = C01 [X(C3H7O1)]\\@

002

1\1\GINC-ANGIE\SP\ROMP2-FC\6-311+G(3df,2p)\C3H7O1(2)\FLORIAN\04-Jul-20 14\0\\#P ROMP2(FC)/6-311+G(3df,2p) geom = check guess = read scf = tight\\Ub 3LYP\\0,2\C,0,-1.5282283695, -0.5431559984,-0.1438716273\H,0,-1.0257321 03,-1.4312286692,0.2525728043\H,0,-2.4687320231,-0.3977486575,0.399325 7744\C,0,-0.647309998,0.656259875,-0.0451791341\C,0,0.8103799633,0.580 6326613,-0.3461318616\H,0,1.3170424042,1.4858461135,0.0290955791\O,0,1 .3628052014,-0.6011459593, 0.2374465399\H,0,2.2487572593,-0.7243237077, -0.135096817\H,0,-1.0952829067,1.6465521505,-0.0138327728\H,0,-1.79786 16938,-0.771223483,-1.1921339971\H,0,0.9738276359,0.5654447648,-1.4453 011676\\Version = AM64L-G03RevD.01\State = 2-A\HF = -192.550861\MP2 = -193.288 0859\RMSD = 4.994e-09\Thermal = 0.\PG = C01 [X(C3H7O1)]\\@

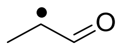

001

1\1\GINC-NODE3\SP\ROMP2-FC\6-311+G(3df,2p)\C3H5O1(2)\ZIP06\04-Jul-2014\0\\#p ROMP2(FC)/6-311+G(3df,2p) scf = tight geom = check guess = read\\b3ly p/6-31G(d)\\0,2\C,0,1.8938999541, 0.0858307555,0.052592071\H,0,2.515680 8587,-0.0779424919,0.9448676243\H,0,2.4330903784,-0.384144323,-0.78296 82956\C,0,0.5428999382,-0.5124344158,0.2226288237\H,0,0.4491826179,-1. 5793772011,0.415932102\C,0,-0.6728004362,0.2312090618,0.1496273877\O,0 ,-1.7947812927,-0.2743908772,0.2927271271\H,0,1.8425023353,1.163447391 2,-0.1362463911\H,0,-0.5678823536, 1.3190051007,-0.0481684492\\Version = AM64L-G03RevD.01\State = 2-A\HF = -191.3927699\MP2 = -192.1049337\RMSD = 7.046 e-09\Thermal = 0.\PG = C01 [X(C3H5O1)]\\@

002

1\1\GINC-NODE3\SP\ROMP2-FC\6-311+G(3df,2p)\C3H5O1(2)\ZIP06\04-Jul-2014\0\\#p ROMP2(FC)/ 6-311+G(3df,2p) scf = tight geom = check guess = read\\b3ly p/6-31G(d)\\0,2\C,0,1.5780111372, -0.4718064054,0.0960583099\H,0,1.0344 203802,-1.4191967178,0.0995874724\H,0,2.3284475368, -0.4904320222,-0.70 76304885\C,0,0.6380000577,0.6655971985,-0.0803220404\C,0,-0.7739657715 , 0.4837795925,-0.2221286711\H,0,-1.3754335531,1.4056242903,-0.34775163 36\O,0,-1.3283570132,-0.6248313258,-0.210099179\H,0,1.0268945782,1.682 3605298,-0.1043804996\H,0,2.1402416477,-0.3756111399,1.0362607298\\Ver sion = AM64L-G03RevD.01\State = 2-A\HF = -191.3939538\MP2 = -192.1062969\RMSD = 3.036e-09\Thermal = 0.\PG = C01 [X(C3H5O1)]\\@

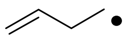

001

1\1\GINC-NODE3\SP\ROMP2-FC\6-311+G(3df,2p)\C4H7(2)\ZIP06\04-Jul-2014\0\\#p ROMP2(FC)/6-311+G(3df,2p) scf = tight geom = check guess = read\\b3lyp/ 6-31G(d)\\0,2\C,0,1.8407488429,

0.0076440476,0.0848031314\H,0,2.8579744 759,-0.1782223493,-0.2447966779\C,0,0.6923695976, -0.7806403738,-0.4630 623379\H,0,0.5109973282,-1.6814527677,0.1516211346\C,0,-0.5890388135, 0.0150227561,-0.5405167309\H,0,1.6909555895,0.6837904988,0.920789708\C, 0,-1.7188290202, -0.2892629889,0.0985688705\H,0,-2.6110914162,0.3247586 189,0.0091880752\H,0,-1.7962677204, -1.1685743141,0.73550484\H,0,0.9547 572319,-1.1547730969,-1.4642749489\H,0,-0.5500570955, 0.9093386794,-1.1 635502342\\Version = AM64L-G03RevD.01\State = 2-A\HF = -155.5227436\MP2 = -156 .1656335\RMSD = 7.125e-09\Thermal = 0.\PG = C01 [X(C4H7)]\\@

002

1\1\GINC-NODE3\SP\ROMP2-FC\6-311+G(3df,2p)\C4H7(2)\ZIP06\04-Jul-2014\0 \\#p ROMP2(FC)/6-311+G(3df,2p) scf = tight geom = check guess = read\\b3lyp/ 6-31G(d)\\0,2\C,0,1.8689070856, 0.2582860262,0.1555750242\H,0,2.6923341 404,0.0375294841,0.8262393935\C,0,0.7393736653, -0.7004290488,-0.011113 3434\H,0,0.6605015391,-1.3481088533,0.8761331534\C,0,-0.6171397077,-0. 0907154631,-0.2916312398\H,0,1.978588395,1.1003133019,-0.5206975208\C, 0,-0.9403209192, 1.1984693506,-0.1868078062\H,0,-1.9453272426,1.5491092 304,-0.4052928686\H,0,-0.2193290008, 1.9485194714,0.1277500844\H,0,-1.3 830438286,-0.8033297372,-0.6007970854\H,0,0.9628916935,- 1.4040959023,- 0.8381093614\\Version = AM64L-G03RevD.01\State = 2-A\HF = -155.521402\MP2 = -1 56.1647294\RMSD = 8.144e-09\Thermal = 0.\PG = C01 [X(C4H7)]\\@

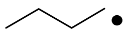

001

1\1\GINC-NODE3\SP\ROMP2-FC\6-311+G(3df,2p)\C4H9(2)\ZIP06\04-Jul-2014\0 \\#p ROMP2(FC)/ 6-311+G(3df,2p) scf = tight geom = check guess = read\\b3lyp/ 6-31G(d)\\0,2\C,0,1.9720409979, 0.0011619292,-0.20730015\H,0,2.86271713 96,-0.4957793096,-0.5790595852\C,0,0.6733835087, -0.7236348296,-0.09320 2365\H,0,0.6474206892,-1.3149240723,0.843508194\C,0,-0.5571270976,0.19 96819459,-0.1120600069\H,0,2.0940205316,0.9893708897,0.2279281782\C,0, -1.8790015403,-0.5574569555, 0.044020749\H,0,-2.7351942821,0.1264706078 ,0.0305988281\H,0,-2.0196751158,-1.28220105, -0.7674663392\H,0,-1.90981 91792,-1.1116371778,0.9904292908\H,0,-0.4593705859,0.9405841464,0.6939 591429\H,0,-0.5630807826,0.7687723095,-1.0510469638\H,0,0.5877200863,- 1.4684913737,-0.8987564029\\ Version = AM64L-G03RevD.01\State = 2-A\HF = -156 .7142138\MP2 = -157.3884319\RMSD = 6.698e-09\Thermal = 0.\PG = C01 [X(C4H9)]\\@

002

1\1\GINC-NODE3\SP\ROMP2-FC\6-311+G(3df,2p)\C4H9(2)\ZIP06\04-Jul-2014\0 \\#p ROMP2(FC)/ 6-311+G(3df,2p) scf = tight geom = check guess = read\\b3lyp/6-31G(d)\\0,2\C,0,1.8443280319, 0.1088851326,0.6261333872\H,0,2.8502443 253,0.1532808515,0.2206759948\C,0,0.7605046987, -0.6507619681,-0.063288 3105\H,0,0.7872770696,-1.7115407739,0.2524642891\C,0,-0.6564434877,-0. 1069423404,0.2059646623\H,0,1.6936160562,0.4894861168,1.6332058965\C,0 ,-0.8902255675, 1.2938256796,-0.3681835695\H,0,-1.9036891474,1.65009831 19,-0.1506742381\H,0,-0.1804940874, 2.0162707262,0.0507629421\H,0,-0.76 12864568,1.2987260633,-1.4578392047\H,0,-1.3919035582,

-0.8048230024,-0 .215756183\H,0,-0.8323406308,-0.0949409145,1.2910473317\H,0,0.94713775 41, -0.6693282625,-1.1470904178\\Version = AM64L-G03RevD.01\State = 2-A\HF = -156.7133328\MP2 = -157.3882067\RMSD = 3.914e-09\Thermal = 0.\PG = C01 [X(C4H9)]\\@

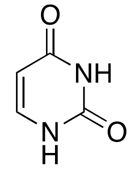

1\1\GINC-Z7\SP\RMP2-FC\6-311+G(3df,2p)\C4H4N2O2\ZIPSE\11-Oct-2007\0\\# p MP2(FC)/ 6-311+G(3df,2p) scf = tight geom = check guess = read\\ur_1 MP2(FC)/6-311+G(3df,2p) sp\\0,1\C,0, 0.0049427144,0.0003758988,0.0012319678\N ,0,-0.0036272722,0.0003630256,1.3968633969\ C,0,1.1359719924,0.00012327 79,2.1675393905\C,0,2.3690879744,-0.0002361342,1.6174131413\ C,0,2.5192 86828,-0.000349624,0.1650906523\N,0,1.2856535992,-0.0000800562,-0.5257 504815\ H,0,0.9700236259,0.0002190131,3.2395684286\H,0,-0.9228822746,0. 0007713476,1.8166667067\O,0,- 1.014199282,0.0006975846,-0.6632680587\O, 0,3.5711695688,-0.0006444836,-0.4515389654\ H,0,1.3416407416,-0.0000984 824,-1.538176708\H,0,3.2654166747,-0.0004425771,2.2223544156\\ Version = IA32L-G03RevD.01\State = 1-A\HF = -412.6149163\MP2 = -414.094278\RMSD = 2.357e -09\Thermal = 0.\PG = C01 [X(C4H4N2O2)]\\@

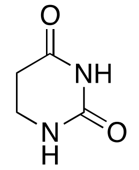

1\1\GINC-TICHY\SP\RMP2-FC\6-311+G(3df,2p)\C4H6N2O2\ZIPSE\11-Oct-2007\0 \\#p MP2(FC)/6- 311+G(3df,2p) scf=tight geom=check guess=read\\dhur_1 M P2(FC)/6-311+G(3df,2p) sp\\0,1\C,0,- 0.0650980891,0.1042590637,0.049228 1943\N,0,-0.0289535826,-0.2476642366,1.3759868317\ C,0,1.1425096537,0.0 19197509,2.2059029197\C,0,2.3951507741,-0.450540879,1.4655639854\ C,0,2.4502808209,0.0846489268,0.0425125149\N,0,1.203279771,0.3250051773,-0. 5212617906\ H,0,1.0296435286,-0.5250956494,3.1475018971\H,0,-0.94345165 33,-0.2432038745,1.8081531152\O,0,- 1.0832577048,0.2062991228,-0.611913 7832\O,0,3.4783631935,0.2654096136,-0.5803836248\ H,0,1.1885261424,0.59 9136637,-1.4973487812\H,0,2.4027122645,-1.5467359515,1.4032191231\H,0, 3.3079662477,-0.139670679,1.9790323426\H,0,1.2269885552,1.0898983977,2.4488035195\\Version = IA32L-G03RevD.01\State = 1-A\HF = -413.7901097\MP2 = -4 15.2957353\RMSD = 8.573e-09\Thermal = 0.\PG = C01 [X(C4H6N2O2)]\\@

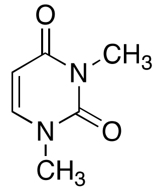

1\1\GINC-NODE-12\SP\RMP2-FC\6-311+G(3df,2p)\C6H8N2O2\ZIP06\28-Oct-2009 \0\\#P MP2(FC)/6- 311+G(3df,2p) scf=tight\\MP2(FC)/6-311+G(3df,2p) Dime thylUracil sp\\0,1\C,0,2.821841,-0.16459,- 0.000056\H,0,3.122405,0.4013 64,-0.885316\N,0,1.37424,-0.37612,-0.000047\C,0,0.816627,-1.630559,0.0 00083\H,0,1.525969,-2.451597,0.00018\C,0,-0.518345,-1.830879,0.00011\H ,0,-0.944376,-2.824934,

0.000184\C,0,-1.435078,-0.703247,-0.000029\O,0, -2.654591,-0.794809,-0.000199\N,0,-0.793303, 0.565571,0.000091\C,0,0.57 9448,0.776102,-0.000011\O,0,1.08217,1.890325,-0.000051\H,0,3.315062, -1.138215,-0.000325\H,0,3.122526,0.400948,0.88543\C,0,-1.623286,1.773687 ,0.000101\H,0,-2.661738, 1.448853,-0.000456\H,0,-1.407559,2.374825,0.88 6856\H,0,-1.406722,2.375385,-0.886052\\Version= IA32L-G03RevD.01\State= 1-A\HF=-490.6923947\MP2=-492.5231659\RMSD=8.372e-09\Thermal=0\ PG=C01 [X(C6H8N2O2)]\\@

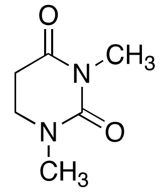

1\1\GINC-NODE-13\SP\RMP2-FC\6-311+G(3df,2p)\C6H10N2O2\ZIP06\28-Oct-200 9\0\\#P MP2(FC)/ 6-311+G(3df,2p) scf=tight\\MP2(FC)/6-311+G(3df,2p) Dim ethyldihydroUracil sp\\0,1\C,0,-2.827958,- 0.071536,-0.002248\H,0,-3.05 4664,0.911813,-0.411553\N,0,-1.388742,-0.285038,-0.073535\C,0,-0.87271 1,-1.578059,0.356774\H,0,-1.582884,-2.349366,0.042809\C,0,0.489157,-1. 821764,-0.278422\ H,0,0.375909,-1.955642,-1.362585\C,0,1.434731,-0.6562 18,-0.0562\O,0,2.644732,-0.79398,-0.005374\ N,0,0.8177,0.589213,0.04910 8\C,0,-0.576388,0.825,-0.066833\O,0,-0.993138,1.971127,-0.158381\H,0,- 3.193839,-0.114816,1.033993\H,0,-3.339554,-0.843039,-0.587345\C,0,1.65 7253,1.788201,0.154965\ H,0,2.680697,1.454019,0.312937\H,0,1.589998,2.3 81432,-0.760643\H,0,1.318435,2.403636,0.990304\ H,0,-0.802244,-1.62856, 1.456029\H,0,0.96818,-2.71961,0.118857\\Version=IA32L-G03RevD.01\State =1- A\HF=-491.8655838\MP2=-493.7221289\RMSD=4.117e-09\Thermal=0.\PG=C01 [X(C6H10N2O2)]\\@

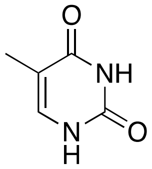

1\1\GINC-LX64I64\SP\RMP2-FC\6-311+G(3df,2p)\C5H6N2O2\UI271AB\14-Sep-20 07\0\\#P MP2(FC)/6- 311+G(3df,2p) scf=tight geom=check guess=read\\thy_ 1 MP2(FC)/6-311+G(3df,2p)//B3LYP/6-31G(d) sp\\0,1\C,0,-0.143842004,-0. 001933543,0.0755335496\N,0,0.025945792,0.0728821245,1.3024995744\C,0, 1.2559802389,0.0754546615,1.92833794\C,0,2.4219425863,0.004137564,1.24 72916766\C,0,2.3654939878, -0.0786253924,-0.2174637978\N,0,1.0632211003 ,-0.0743763482,-0.7533192171\H,0,1.2192928455, 0.1388354919,3.011208725 3\H,0,-0.8304430144,0.1286729027,1.8357825107\O,0,-1.2358197537,-0.002 3743981,-0.6139712185\O,0,3.3373088023,-0.1473815096,0.9546155654\C,0,3.775726934,0.0043552185, 1.8956229568\H,0,4.3306710432,0.905582453,1.6405854325\H,0,4.3789480234,0.8469527712,1.5390001 92\H,0,3.6949085247 ,0.0698361521,2.9851646726\H,0,0.9968103778,-0.1306797822,-1.763462777 6\\Version=IA64L-G03RevD.01\State=1-A\HF=-451.6653897\MP2=-453.3203336 \RMSD=8.000e- 09\Thermal=0.\PG=C01 [X(C5H6N2O2)]\\@

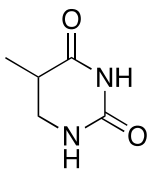

1\1\GINC-LX64I42\SP\RMP2-FC\6-311+G(3df,2p)\C5H8N2O2\UI271AB\14-Sep-20 07\0\\#P MP2(FC)/ 6-311+G(3df,2p) scf=tight geom=check guess=read\\dhth y_1 MP2(FC)/6-311+G(3df,2p)//B3LYP/6-31G(d) sp\\0,1\C,0,-0.0273144647, -0.0517707883,-0.0152162324\N,0,0.114718625,-0.1655631909,1.3444295402 \C,0,1.4254526522,-0.1005109221,1.9823023315\C,0,2.2023081137,1.111053 3387,1.4533788002\ C,0,2.1967515086,1.1222582504,-0.0762679869\N,0,1.08 28749696,0.5297651161,-0.6564477409\ H,0,1.2787683302,-0.0063170865,3.0 621678303\H,0,-0.611485412,-0.7244413335,1.7726422538\O,0,- 1.014740531 5,-0.3937733558,-0.6416623841\O,0,3.0680194007,1.6301359019,-0.7573404 376\C,0, 3.6283335899,1.1862892766,2.0017739203\H,0,4.192913975,0.27986 11054,1.7556991839\ H,0,4.1566534028,2.0350575973,1.5627472583\H,0,3.6175878066,1.3000549782,3.0910422487\H,0,1.002 698785,0.6027217641,-1.664 6182194\H,0,1.6492543778,2.0143467878,1.7533835688\H,0,2.0067315629, -1.0201725882,1.8058434224\\Version=IA64L-G03RevD.01\State=1-A\HF=-452.8381248\MP2=-454.518753\ RMSD=8.718e-09\Thermal=0.\PG=C01 [X(C5H8N2O2)]\\@

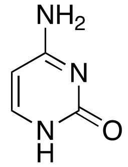

1\1\GINC-HAENSEL\SP\RMP2-FC\6-31G(d)\C4H5N3O1\WALED\07-Jul-2007\0\\#P MP2/6-31G(D) SCF=TIGHT\\single point\\0,1\C,0,-1.2496475018,-0.2395580 849,-0.2717188748\N,0,-0.9466938197,- 0.6345633883,1.0689296409\C,0,0.2 805118077,-0.5012677365,1.628938362\C,0,1.3007141918, 0.0324750081,0.90 64577638\C,0,0.9825374444,0.4246824062,-0.4431736669\N,0,-0.2086148254, 0.2907858955,-0.9935899051\H,0,0.3902563332,0.8350812672,2.6560415179\H,0,2.2886757783, 0.1643046998,1.3298654272\O,0,-2.3878439001,-0.4033 352667,0.6779491311\N,0,1.9551545889, 1.0041396412,-1.2068639621\H,0,2 .9261042221,0.8865626459,0.959956258\H,0,1.7379942207, 1.1189860997,-2.1876842771\H,0,-1.7238966119,-1.0286146403,1.582974718\\Version=x86-L inux- G03RevB.03\State=1-A\HF=-392.6112164\MP2=-393.7649579\RMSD=4.360e -09\PG=C01 [X(C4H5N3O1)]\\@

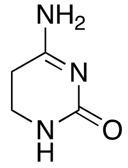

1\1\GINC-MAX\SP\RMP2-FC\6-31G(d)\C4H7N3O1\WALED\13-Jul-2007\0\\#P MP2/ 6-31G(D) SCF=TIGHT\\single point\\0,1\C,0,-1.1637496128,-0.4946183861, -0.4305749737\N,0,-1.0206804856,- 0.7338088508,0.9348317048\C,0,0.27963 34172,-0.6926381613,1.5797444349\ C,0,0.9873064063,0.5756038241,1.10368 869\C,0,0.8753250895,0.642688007,-0.4073796258\N,0,- 0.1069627083,0.155 7087496,-1.0941633654\H,0,0.8967814876,-1.5740431957,1.3361993042\H,0, 2.0351188435,0.5849304324,1.4228669789\O,0,-2.183707024,-0.8194582705, -1.0154618569\ N,0,1.8996177521,1.2500366972,-1.0660464619\H,0,1.765439 2368,1.4355365114,-2.0514227306\ H,0,2.5725458785,1.8123353142,-0.56823 4805\H,0,-1.7144591086,-1.3779809786,1.2919187976\

H,0,0.4940322473,1.4 587387015,1.5312615521\H,0,0.1452838978,-0.6736184955,2.6658814638\\Version=x86-Linux-G03RevB.03\State=1-A\HF=-393.7763666\MP2=-394.9390721\RMSD=9.088e-09\PG=C01 [X(C4H7N3O1)]\\@

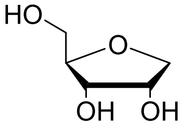

002

1\1\GINC-IBLIS\SP\RMP2-FC\6-311+G(3df,2p)\C5H10O4\FLORIAN\23-Nov-2011\0\\#P MP2(FC)/ 6-311+G(3df,2p) scf=tight\\n2\\0,1\C,0,-1.616185,0.25859 8,0.398034\C,0,-1.629233,-1.267119,0.220007\O,0,-0.284321,-1.677924,-0 .106786\C,0,0.49906,-0.514933,-0.330272\C,0,-0.118638,0.541254,0.597362\C,0,1.958991,-0.810047,-0.032162\O,0,2.642631,0.440409,-0.193763\O,0 ,0.181314,1.880451, 0.252552\O,0,-2.065398,0.877493,-0.796333\H,0,0.403 5,-0.160996,-1.36892\H,0,-2.317059,-1.540961,-0.58787\H,0,-1.925542,-1.790734,1.136346\H,0,0.168537,0.322111,1.63859\H,0,1.137117,1.896545,0.063234\H,0,2.051912,-1.189444,0.995861\H,0,2.351931,-1.57148,-0.71992 \H,0,3.565844,0.328473,0.075182\H,0,-2.223886,0.580892,1.256451\H,0,-1 .570125,1.715648,-0.852121\\Version=AM64L-G03RevD.01\State=1-A\HF=-494 .7630135\MP2=-496.5596005\RMSD=7.308e-09\Thermal=0.\PG=C01 [X(C5H10O4)]\\@

011

1\1\GINC-BORIX\SP\RB3LYP\6-31G(d)\C5H10O4\FLORIAN\23-Nov-2011\0\\#P B3 LYP/6-31G(d) scf=tight\\n_11\\0,1\C,0,-1.493856,-0.266892,0.534194\C,0 ,-0.850279,-1.631428,0.261655\O,0,0.440016,-1.387074,-0.334333\C,0,0.5 66437,0.008266,-0.631038\C,0,-0.289088,0.683505,0.451462\C,0,2.052814, 0.34108,-0.598914\O,0,2.634499,0.002793,0.649366\O,0,-0.7506,1.986866, 0.120917\O,0,-2.417535, 0.019614,-0.504841\H,0,0.141525,0.244335,-1.618 546\H,0,-1.480255,-2.209978,-0.423105\H,0,-0.694254, -2.20916,1.179879\ H,0,0.264952,0.681017,1.398971\H,0,-0.044029,2.623478,0.301453\H,0,2.5 59244,-0.174239,-1.429777\H,0,2.196381,1.41883,-0.742327\H,0,2.371367, -0.921145,0.803299\H,0,-1.988232,-0.227207,1.516039\H,0,-2.493908,0.98 9292,-0.538923\\Version=AM64L-G03RevD.01\State=1-A\HF=-497.3966623\RMS D=7.448e-09\Thermal=0.\Dipole=0.0398611,0.4479262,0.3169659\PG=C01 [X(C5H10O4)]\\@

016

1\1\GINC-TOFU\SP\RB3LYP\6-31G(d)\C5H10O4\FLORIAN\23-Nov-2011\0\\#P B3L YP/6-31G(d) scf=tight\\n_16\\0,1\C,0,-1.514704,-0.414311,0.53159\C,0,- 0.494235,-1.540664,0.542248\O,0,0.454556,-1.128547,-0.452196\C,0,0.637 753,0.309258,-0.307637\C,0,-0.598584,0.836588,0.476865\C,0,1.976495,0.557466,0.378057\O,0,3.040663,-0.048003,-0.330156\O,0,-1.233882,1.94708 5,-0.109495\O,0,-2.321157,-0.451075,-0.648807\H,0,0.660642,0.729616,-1 .318129\H,0,-0.905447,-2.507812,0.240468\H,0,-0.025034,-1.644255,1.532 773\H,0,-0.301823,1.129255,1.492392\H,0,-1.906553,1.56464,-0.705313\H, 0,2.180401, 1.633164,0.416082\H,0,1.926533,0.191686,1.419729\H,0,2.7551 45,-0.963036,-0.493322\H,0,-2.202515,-0.412423,1.38012\H,0,-1.743142,- 0.766538,-1.366307\\Version = AM64L-G03RevD.01\State = 1-A\ HF = -497.396408\RMSD = 3.671e-09\Thermal = 0.\Dipole = -0.5012526,-0.8263658,0.3724238\PG = C0 1[X(C5H10O4)]\\@

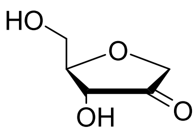

003

1\1\GINC-IBLIS\SP\RB3LYP\6-31G(d)\C5H8O4\FLORIAN\25-Nov-2011\0\\#P B3L YP/6-31G(d) scf=tight\\mm3\\0,1\C,0,0.668762,0.13256,-0.253362\O,0,0.5 5422,-1.263625,0.113233\C,0,-0.804297,-1.660163,-0.032273\C,0,-0.59698 6,0.75173,0.350374\H,0,0.64798,0.241035,-1.348572\H,0,-1.001343,-2.116 813,-1.015112\H,0,-1.059677,-2.388991,0.744603\C,0,-1.602837,-0.36772, 0.089963\O,0,-2.788195,-0.192923,-0.083943\H,0,-0.458346,0.832331,1.44 5719\O,0,-0.994584,1.971368,-0.213559\H,0,-1.966754, 1.932241,-0.288007 \C,0,1.996837,0.626872,0.292239\H,0,2.133994,1.678223,0.021463\H,0,1.9 75927, 0.555196,1.392723\O,0,3.085517,-0.090217,-0.258559\H,0,2.90369,- 1.029719,-0.091851\\Version= AM64L-G03RevD.01\State=1-A\HF=-496.193095\ RMSD=5.056e-09\Thermal=0.\Dipole=-0.3510017,-0.5917434,0.372556\PG=C01 [X(C5H8O4)]\\@

027

1\1\GINC-IBLIS\SP\RB3LYP\6-31G(d)\C5H8O4\FLORIAN\25-Nov-2011\0\\#P B3L YP/6-31G(d) scf=tight\\mm27\\0,1\C,0,0.61624,-0.497657,-0.285168\O,0,- 0.128563,-1.681701,0.048425\C,0,-1.515,-1.385661,-0.096676\C,0,-0.2066 76,0.612581,0.385068\H,0,0.628409,-0.330942,-1.374192\H,0,-1.89344,-1. 658612,-1.094563\H,0,-2.088938,-1.939929,0.653701\C,0,-1.617307,0.1258 54,0.082974\O,0,-2.576042, 0.849261,-0.07088\H,0,-0.053806,0.536529,1.4 78572\O,0,0.049752,1.912065,-0.080048\H,0,-0.812368, 2.365702,-0.138515\C,0,2.048571,-0.617837,0.217654\H,0,2.038835,-0.727444,1.31497\H,0,2. 516112,-1.513301,-0.202592\O,0,2.825578,0.488137,-0.207238\H,0,2.34442 ,1.30223,0.017429\\Version=AM64L-G03RevD.01\State=1-A\HF=-496.1914556\RMSD=4.196e-09\Thermal=0.\Dipole=-0.6141105,-0.2333055, 0.3333369\PG=C0 1 [X(C5H8O4)]\\@

005

1\1\GINC-IBLIS\FOpt\RB3LYP\6-31G(d)\C5H8O4\FLORIAN\24-Nov-2011\0\\#P B 3LYP/6-31G(d) opt\\mm5\\0,1\C,-0.7246161616,0.7079304043,0.5488782901\O,-0.4488828908,-0.367322065, 1.4695360754\C,0.4088558247,-1.3225826047,0.865276313\C,0.4809912559,0.8015786651,-0.3885364075\ H,-0.8154793724 ,1.6208062814,1.1461243002\H,1.1169038034,-1.7011541412,1.610850895\H, -0.1496493129,-2.1710132909,0.4432561785\C,1.1305180051,-0.5881425241, -0.2561445183\ O,2.0919599484,-0.9654459543,-0.8870855023\H,0.200815740 1,0.9972964812,-1.4313931455\ O,1.395809785,1.7756782829,0.1052193435\H,2.230491699,1.640632752,-0.3755752818\C,-2.0332130027, 0.4612839665,-0.1910436464\H,-2.8166270447,0.2386902105,0.5471840894\H,-2.3186344807, 1.3766458799,-0.7353203114\O,-1.8317368563,-0.6315991499,-1.0840841474\H,-2.6775099395,-0.851454194,-1.5013105246\\Version = AM64L-G03RevD.01\State = 1-A\HF = -496.1867202\RMSD = 5.982e-09\RMSF = 1.424e-05\Thermal = 0.\Dip ole = -1.0981337,0.1759081,-0.4006576\PG = C01 [X(C5H8O4)]\\@

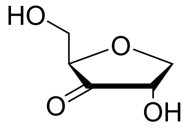

005

1\1\GINC-NODE10\SP\RMP2-FC\6-311+G(3df,2p)\C5H8O4\ZIP06\28-Nov-2011\0\ \#p MP2(FC)/ 6-311+G(3df,2p) scf=tight\\rnak_5\\0,1\C,0,0.38856,0.87653 6,-0.053018\C,0,1.404598,-0.150158,-0.576923\C,0,0.670587,-1.462875,-0 .367945\O,0,-0.287105,-1.260272,0.693615\C,0,-0.614641, 0.122835,0.8190 05\O,0,2.541492,-0.102034,0.283881\C,0,-2.065434,0.37162,0.38686\O,0,- 2.320299,-0.217086,-0.877379\O,0,0.426263,2.072448,-0.231925\H,0,-0.47 7936,0.433383,1.864906\H,0,1.346746,-2.265683,-0.063726\H,0,0.149862,- 1.753295,-1.290399\H,0,-2.749181,-0.016381,1.156301\H,0,-2.231783, 1.44 8413,0.284855\H,0,-2.200905,-1.172309,-0.74862\H,0,1.673059,0.035982,- 1.624662\H,0,2.905317, 0.797698,0.227939\\Version=AM64L-G03RevD.01\Stat e=1-A\HF=-493.5918816\MP2=-495.3537861\ RMSD=4.296e-09\Thermal=0.\PG=C0 1 [X(C5H8O4)]\\@

002

1\1\GINC-NODE17\SP\RMP4SDTQ-FC\6-31+G(d)\C5H8O4\ZIP06\25-Nov-2011\0\\# p MP4(FC)/6-31+G(d) scf=tight\\rnak_2\\0,1\C,0,-0.36144,0.838163,-0.17 616\C,0,-1.18077,-0.216734,0.579079\C,0,-0.595139,-1.537025,0.073972\O ,0,0.342099,-1.23051,-0.984072\C,0,0.777553,0.116654,-0.897382\O,0,-2. 555053,-0.090463,0.254099\C,0,2.098475,0.274203,-0.145904\O,0,1.853355 ,-0.044578,1.221264\O,0,-0.651612,2.011307,-0.241671\H,0,0.895203,0.51 2985,-1.912872\H,0,-1.379054,-2.170429,-0.349363\H,0,-0.074756,-2.0759 98,0.872309\H,0,2.840143,-0.403031,-0.593164\H,0,2.448847,1.311532,-0. 263348\ H,0,2.686303,0.028489,1.710168\H,0,-1.007855,-0.084392,1.657973 \H,0,-2.751222,0.863221,0.279709\\ Version=AM64L-G03RevD.01\State=1-A\H F=-493.4187827\MP2=-494.7900801\MP3=-494.8156732\ MP4D=-494.8472258\MP4 DQ=-494.8248191\MP4SDQ=-494.8430305\MP4SDTQ=-494.8930259\ RMSD=7.054e-0 9\Thermal=0.\PG=C01 [X(C5H8O4)]\\@

008

1\1\GINC-NODE21\SP\RMP2-FC\6-311+G(3df,2p)\C5H8O4\ZIP06\28-Nov-2011\0\\#p MP2(FC)/6-311+G(3df,2p) scf=tight\\rnak_8\\0,1\C,0,-0.589838,0.888 088,0.054388\C,0,-1.566785,-0.219942, 0.495006\C,0,-0.667802,-1.446427, 0.519621\O,0,0.394904,-1.199244,-0.420699\C,0,0.660675,0.204223,-0.484 205\O,0,-2.54468,-0.369068,-0.52979\C,0,1.920073,0.539861,0.330177\O,0 ,3.020083,-0.213751,-0.133704\O,0,-0.808888,2.078483,0.08882\H,0,0.832 948,0.469501,-1.533702\H,0,-1.191296,-2.350392, 0.200776\H,0,-0.260933, -1.600958,1.529747\H,0,2.164557,1.599184,0.208159\H,0,1.728744,0.35828 9,1.401732\H,0,2.711076,-1.135188,-0.17411\H,0,-2.023546,0.004276,1.46 834\H,0,-2.990845,0.489115,-0.627881\\Version=AM64L-G03RevD.01\State=1 -A\HF=-493.5914641\MP2=-495.3530798\RMSD= 4.873e-09\Thermal=0.\PG=C01 [X(C5H8O4)]\\@

006

1\1\GINC-NODE10\SP\RMP4SDTQ-FC\6-31+G(d)\C5H8O4\ZIP06\25-Nov-2011\0\\# p MP4(FC)/ 6-31+G(d) scf=tight\\rnak_6\\0,1\C,0,-0.250444,0.844755,-0.1 05813\C,0,-1.362039,0.054008,

0.601613\C,0,-0.803028,-1.359052,0.560183 \O,0,0.019022,-1.43531,-0.616792\C,0,0.599512,-0.166021, -0.881496\O,0, -2.529671,0.12792,-0.214758\C,0,2.067003,-0.076934,-0.436348\O,0,2.204 306,- 0.029394,0.981521\O,0,-0.083035,2.044752,-0.063915\H,0,0.536186,0 .027871,-1.961377\H,0,-1.590001,- 2.111391,0.469037\H,0,-0.201996,-1.55 4562,1.457917\H,0,2.582489,-0.980984,-0.772313\H,0,2.536917, 0.79311,-0 .916366\H,0,2.077236,0.892731,1.253894\H,0,-1.544997,0.425228,1.618395 \H,0,-2.786836, 1.063721,-0.266468\\Version=AM64L-G03RevD.01\State=1-A\ HF=-493.4171797\MP2=-494.7891933\ MP3=-494.8146459\MP4D=-494.8461854\MP 4DQ=-494.8236301\MP4SDQ=-494.8419679\MP4SDTQ=- 494.8922012\RMSD=7.295e- 09\Thermal=0.\PG=C01 [X(C5H8O4)]\\@

004

1\1\GINC-NODE19\SP\RMP4SDTQ-FC\6-31+G(d)\C5H8O4\ZIP06\25-Nov-2011\0\\# p MP4(FC)/6- 31+G(d) scf=tight\\rnak_4\\0,1\C,0,-0.127178,0.743299,0.10 9568\C,0,-1.557495,0.30924,0.461064\C,0,- 1.400254,-1.201281,0.540003\O ,0,-0.316569,-1.559011,-0.335164\C,0,0.57048,-0.461607,-0.504098\O,0,- 2.38747,0.634084,-0.652352\C,0,1.940621,-0.690339,0.171332\O,0,2.83431 4,0.357021,-0.138401\ O,0,0.359499,1.843214,0.291449\H,0,0.743341,-0.29 1537,-1.575177\H,0,-2.297898,-1.723553,0.199752\ H,0,-1.174773,-1.50651 4,1.572613\H,0,1.800005,-0.816185,1.258087\H,0,2.371482,-1.617919,-0.2 15701\ H,0,2.395615,1.18954,0.113886\H,0,-1.914666,0.765766,1.39266\H,0 ,-2.398339,1.602073,-0.737585\\ Version=AM64L-G03RevD.01\State=1-A\HF=-493.4168227\MP2=-494.7885391\MP3=-494.8141706\ MP4D=-494.8455992\MP4DQ= -494.8228344\MP4SDQ=-494.841177\MP4SDTQ=-494.8915703\RMSD= 5.676e-09\Th ermal=0.\PG=C01 [X(C5H8O4)]\\@

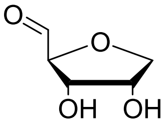

001

1\1\GINC-NODE10\SP\RMP4SDTQ-FC\6-31+G(d)\C5H8O4\ZIP06\25-Nov-2011\0\\# p MP4(FC)/6- 31+G(d) scf=tight\\rnac_1\\0,1\C,0,-0.052832,0.569038,0.57 8101\C,0,-1.541225,0.232106,0.414693\C,0,- 1.506857,-1.297723,0.282483\ O,0,-0.166407,-1.668553,-0.116224\C,0,0.564466,-0.484896,-0.365529\O, 0,-2.030159,0.788735,-0.793508\C,0,2.039476,-0.663786,-0.112387\O,0,2.7 86286,0.297074,-0.083784\ O,0,0.213329,1.902644,0.207657\H,0,0.430288,- 0.128007,-1.401228\H,0,-2.226713,-1.623391,-0.475536\ H,0,-1.727185,-1. 801509,1.230302\H,0,0.26729,0.365594,1.611389\H,0,1.176078,1.962855,0. 068458\ H,0,2.404545,-1.696715,0.058767\H,0,-2.14263,0.561901,1.27418\H ,0,-1.62424,1.671645,-0.863617\\Version= AM64L-G03RevD.01\State=1-A\HF= -493.4186915\MP2=-494.7921944\MP3=-494.8178761\MP4D= -494.8490527\MP4DQ =-494.8264347\MP4SDQ=-494.8443984\MP4SDTQ=-494.8943777\RMSD= 9.136e-09\Thermal=0.\PG=C01 [X(C5H8O4)]\\@

007

1\1\GINC-NODE16\SP\RMP4SDTQ-FC\6-31+G(d)\C5H8O4\ZIP06\26-Nov-2011\0\\# p MP4(FC)/6- 31+G(d) scf=tight\\rnac_7\\0,1\C,0,-0.426537,0.84075,0.442 37\C,0,-1.220496,-0.478101,0.670227\C,0,- 0.176917,-1.538014,0.352932\O ,0,0.437316,-1.023003,-0.837658\C,0,0.654174,0.368425,-0.611364\O, 0,-2 .304854,-0.52516,-0.2599\C,0,2.05245,0.646506,-0.087695\O,0,2.789978,- 0.194049,0.373449\O,0,

-1.241822,1.894955,0.00581\H,0,0.505025,0.91079, -1.552021\H,0,-0.606668,-2.514949,0.117599\ H,0,0.560438,-1.645989,1.15 9511\H,0,0.058357,1.159717,1.372546\H,0,-2.022218,1.458799,-0.390509\H, 0,2.34194,1.722119,-0.117775\H,0,-1.657564,-0.545647,1.668734\H,0,-1. 920294,-0.804182,-1.110503\\ Version=AM64L-G03RevD.01\State=1-A\HF=-493 .4107833\MP2=-494.7881457\MP3=-494.812873\ MP4D=-494.8444095\MP4DQ=-494 .8213647\MP4SDQ=-494.8396834\MP4SDTQ=-494.8905659\ RMSD=9.717e-09\Therm al=0.\PG=C01 [X(C5H8O4)]\\@

009

1\1\GINC-NODE27\SP\RMP4SDTQ-FC\6-31+G(d)\C5H8O4\ZIP06\26-Nov-2011\0\\# p MP4(FC)/ 6-31+G(d) scf=tight\\rnac_9\\0,1\C,0,-0.138992,-0.758319,-0. 054055\C,0,-1.192624,0.183608,-0.683898\ C,0,-1.07566,1.419785,0.224107 \O,0,0.325253,1.544674,0.47574\C,0,0.89567,0.235073,0.52675\O,0,- 2.474 192,-0.382045,-0.769395\C,0,2.2134,0.25942,-0.235056\O,0,2.791085,-0.7 43028,-0.59283\O,0,- 0.820357,-1.489924,0.968308\H,0,1.112286,-0.053145 ,1.568491\H,0,-1.63835,1.256217,1.15572\H,0,- 1.417326,2.346437,-0.2431 53\H,0,0.319549,-1.440306,-0.775928\H,0,-0.355727,-2.326723,1.11378\H, 0,2.609117,1.277588,-0.434749\H,0,-0.875636,0.451873,-1.698363\H,0,-2.538978,-0.986753,-0.007474\\ Version=AM64L-G03RevD.01\State=1-A\HF=-493 .416155\MP2=-494.7887372\MP3=-494.8146533\ MP4D=-494.8459478\MP4DQ=-494 .8233586\MP4SDQ=-494.8413287\MP4SDTQ=-494.8911952\ RMSD=7.838e-09\Thermal=0.\PG=C01 [X(C5H8O4)]\\@

013

1\1\GINC-NODE16\SP\RMP4SDTQ-FC\6-31+G(d)\C5H8O4\ZIP06\26-Nov-2011\0\\# p MP4(FC)/ 6-31+G(d) scf=tight\\rnac_13\\0,1\C,0,-0.143633,-0.800854,0. 107016\C,0,-1.077939,0.114556,-0.718773\ C,0,-0.998579,1.423558,0.06981 3\O,0,0.35816,1.515032,0.497642\C,0,0.918476,0.194061,0.593132\O,0,- 2. 419714,-0.361509,-0.748127\C,0,2.204401,0.185257,-0.225817\O,0,2.70222 4,-0.809844,-0.70269\O,0,- 0.850629,-1.318502,1.219701\H,0,1.171277,-0. 035584,1.638991\H,0,-1.686741,1.373915,0.925218\H,0,- 1.230227,2.310507 ,-0.525913\H,0,0.313555,-1.606127,-0.483208\H,0,-1.777733,-1.39653,0.9 26348\H,0, 2.659592,1.191205,-0.351683\H,0,-0.682409,0.253392,-1.733725\H,0,-2.503995,-0.991671,-1.480456\\ Version=AM64L-G03RevD.01\State=1-A\HF=-493.4143607\MP2=-494.7876023\MP3=-494.8134689\ MP4D=-494.8447923\MP4DQ=-494.8221857\MP4SDQ=-494.8401135\MP4SDTQ=-494.8899726\ RMSD=7.627e -09\Thermal=0.\PG=C01 [X(C5H8O4)]\\@

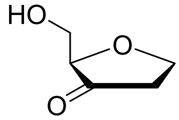

004

1\1\GINC-NODE19\SP\RMP4SDTQ-FC\6-31+G(d)\C5H8O3\ZIP06\28-Nov-2011\0\\# p MP4(FC)/ 6-31+G(d) scf=tight\\dnak_4\\0,1\C,0,-0.248613,0.922374,-0.1 56911\C,0,-1.744335,0.829363,0.107877\ C,0,-1.913299,-0.667703,0.386185\O,0,-0.907298,-1.325507,-0.403491\C,0,0.228873,-0.479154,-0.534157\ C, 0,1.41356,-0.902955,0.361206\O,0,2.5543,-0.119222,0.084253\O,0,0.46894 2,1.895244,-0.027555\ H,0,0.571583,-0.496146,-1.577084\H,0,-2.881259,-1 .070699,0.079469\H,0,-1.768966,-0.886823,1.454629\

H,0,1.115425,-0.8495 95,1.422049\H,0,1.665761,-1.944287,0.141004\H,0,2.281896,0.813316,0.16 4041\
H,0,-2.05617,1.496895,0.914596\H,0,-2.272952,1.121668,-0.809564\\ Version=AM64L-G03RevD.01\
State=1-A\HF=-418.5696028\MP2=-419.7584311\MP 3=-419.7870076\MP4D=-419.8149009\MP4DQ=-
419.7946245\MP4SDQ=-419.810424 6\MP4SDTQ=-419.8548069\RMSD=4.147e-09\Thermal=0.\PG=C01
[X(C5H8O3)]\\@

006

1\1\GINC-NODE14\SP\RMP4SDTQ-FC\6-31+G(d)\C5H8O3\ZIP06\28-Nov-2011\0\\# p MP4(FC)/
6-31+G(d) scf=tight\\dnak_6\\0,1\C,0,0.75349,0.879666,0.0575 65\C,0,1.781423,-0.159912,-0.375986\
C,0,0.993498,-1.46443,-0.264814\O, 0,-0.000933,-1.250857,0.761465\C,0,-0.340302,0.13453,0.833811\
C,0,-1.7 43431,0.387715,0.267575\O,0,-1.902469,-0.236142,-0.996033\O,0,0.771732 ,2.070465,-0.147837\
H,0,-0.306203,0.454559,1.885591\H,0,1.594248,-2.32323,0.044606\H,0,0.504063,-1.70185,-1.218793\
H,0,-2.499239,0.035108,0.985189\H,0,-1.880485,1.463248,0.119566\H,0,-1.817096,-1.188975,-0.82975\
H,0,2.173251,0.058221,-1.372393\H,0,2.616763,-0.130217,0.336313\\Version=AM64L-G03RevD.01\
State=1-A\HF=-418.5698108\MP2=-419.7591604\MP3=- 419.7874907\MP4D=-419.8155894\MP4DQ=-
419.7954971\MP4SDQ=-419.8113017\M P4SDTQ=-419.8555353\RMSD=8.005e-09\Thermal=0.\PG=C01
[X(C5H8O3)]\\@

005

1\1\GINC-NODE10\SP\RMP4SDTQ-FC\6-31+G(d)\C5H8O3\ZIP06\28-Nov-2011\0\\# p MP4(FC)/
6-31+G(d) scf=tight\\dnak_5\\0,1\C,0,-0.386935,0.976261,-0.2 26054\C,0,-1.676441,0.473241,0.409284\
C,0,-1.409704,-1.032459,0.500589\O,0,-0.582001,-1.335148,-0.636048\C,0,0.286721,-0.237313,-0.887214\
C, 0,1.700881,-0.438267,-0.321596\O,0,1.748723,-0.324407,1.097281\O,0,0.0 80136,2.093362,-0.179058\
H,0,0.353226,-0.093591,-1.974948\H,0,-2.30840 7,-1.6483,0.418915\H,0,-0.884872,-1.282634,
1.431378\H,0,2.027937,-1.45 3017,-0.565204\H,0,2.383945,0.2745,-0.805412\H,0,1.749397,0.622815,1.3
07574\H,0,-1.877495,0.974963,1.359085\H,0,-2.505735,0.686023,-0.278845 \\Version=AM64L-G03RevD.01\
State=1-A\HF=-418.5695568\MP2=-419.758704\MP3=-419.7870766\MP4D=-419.8150801\MP4DQ=-
419.7950029\MP4SDQ=-419.81080 86\MP4SDTQ=-419.8550479\RMSD=8.028e-09\Thermal=0.\PG=C01
[X(C5H8O3)]\\@

007

1\1\GINC-AZAZEL\SP\RMP4SDTQ-FC\6-31+G(d)\C5H8O3\FLORIAN\28-Nov-2011\0\\#P MP4(FC)/
6-31+G(d) scf=tight\\dnak_7\\0,1\C,0,-1.009135,0.76969,-0. 112017\C,0,-1.908722,-0.43062,0.183004\C,0,
-0.896885,-1.569372,0.32790 2\O,0,0.205098,-1.213575,-0.52904\C,0,0.35449,0.210301,-0.524253\C,0,
1.482374,0.624732,0.430753\O,0,2.692354,-0.009572,0.069517\O,0,-1.28931 9,1.942634,-0.019091\
H,0,0.616901,0.523271,-1.541702\H,0,-1.263312,-2. 541715,-0.008595\H,0,-0.559798,-1.666913,1.370445\
H,0,1.637991,1.70544 4,0.365767\H,0,1.19696,0.387584,1.469926\H,0,2.4749,-0.952049,-0.03240 9\H,0,-
2.538899,-0.252397,1.058043\H,0,-2.562542,-0.587501,-0.684898\\Version=AM64L-G03RevD.01\State=
1-A\HF=-418.5699841\MP2=-419.759191\MP3=-419.7874961\MP4D=-419.8156021\MP4DQ=-419.795428\
MP4SDQ=-419.8112125\MP4SDTQ=-419.8554712\RMSD=8.207e-09\Thermal=0.\PG=C01 [X(C5H8O3)]\\@

002

1\1\GINC-AZAZEL\SP\RMP4SDTQ-FC\6-31+G(d)\C5H8O3\FLORIAN\28-Nov-2011\0\\#P MP4(FC)/ 6-31+G(d) scf=tight\\dnak_2\\0,1\C,0,-0.588457,0.944187,-0.105612\C,0,-1.67903,0.082257,0.523982\C,0, -1.133926,-1.337419,0.342695\O,0,-0.290452,-1.282207,-0.824789\C,0,0.332655,-0.008709,-0.890948\ C ,0,1.74933,0.005175,-0.323633\O,0,1.666328,-0.277834,1.069725\O,0,-0.4 54699,2.143184,-0.031716\ H,0,0.378582,0.300919,-1.943432\H,0,-1.906245 ,-2.083937,0.141111\H,0,-0.54793,-1.65045,1.214421\ H,0,2.353176,-0.747434,-0.851925\H,0,2.180571,1.000564,-0.510457\H,0,2.555716,-0.213265,1.447795\ H,0,-1.860283,0.378261,1.560594\H,0,-2.606428,0.237259,-0.0427 65\\Version=AM64L-G03RevD.01\ State=1-A\HF=-418.5702614\MP2=-419.7588839\MP3=-419.7871113\MP4D=-419.8152142\MP4DQ=- 419.7953126\MP4SDQ=-419.81 10284\MP4SDTQ=-419.8550794\RMSD=6.301e-09\Thermal=0.\PG=C01 [X(C5H8O3)]\\@

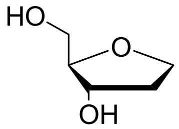

009

1\1\GINC-NODE23\SP\RMP4SDTQ-FC\6-31+G(d)\C5H10O3\ZIP06\28-Nov-2011\0\\#p MP4(FC)/ 6-31+G(d) scf=tight\\prc_9\\0,1\C,0,-1.754937,0.799192,0.03 9645\C,0,-2.086453,-0.702248,0.087162\O,0, -0.853064,-1.414997,-0.142688\C,0,0.192498,-0.478904,-0.373925\C,0,-0.252642,0.816726,0.324691\C, 0,1.498067,-1.045546,0.161909\O,0,2.480037,-0.018285,-0.018068\O,0,0.36 8319,1.982123,-0.173872\ H,0,0.305732,-0.263873,-1.449897\H,0,-2.819335 ,-0.994334,-0.674788\H,0,-2.47604,-1.0092,1.066215\ H,0,-0.077592,0.707 641,1.409867\H,0,1.324629,1.80687,-0.122054\H,0,1.369073,-1.29749,1.22 4987\ H,0,1.774289,-1.962124,-0.378254\H,0,3.313912,-0.316263,0.373271\H,0,-2.339315,1.388543,0.752069\ H,0,-1.91688,1.214171,-0.961277\\Version=AM64L-G03RevD.01\State=1-A\HF=-419.7274458\MP2=- 420.9300699\MP3=-42 0.969141\MP4D=-420.9959076\MP4DQ=-420.9768066\MP4SDQ=-420.9896588\ MP4S DTQ=-421.029432\RMSD=3.832e-09\Thermal=0.\PG=C01 [X(C5H10O3)]\\@

006

1\1\GINC-NODE26\SP\RMP4SDTQ-FC\6-31+G(d)\C5H10O3\ZIP06\28-Nov-2011\0\\#p MP4(FC)/6- 31+G(d) scf=tight\\prc_6\\0,1\C,0,-1.89721,-0.509468,0.02 7249\C,0,-0.957806,-1.70592,0.164784\ O,0,0.318841,-1.25621,-0.318337\C ,0,0.375225,0.180698,-0.306233\C,0,-0.950775,0.668551,0.313943\ C,0,1.6 34958,0.603587,0.437826\O,0,2.796359,0.050322,-0.154534\O,0,-1.376715, 1.937834,-0.143184\ H,0,0.446204,0.531153,-1.349173\H,0,-1.260319,-2.57 4627,-0.428518\H,0,-0.869475,-2.023839,1.214655\ H,0,-0.830652,0.788906,1.397481\H,0,-1.495568,1.880502,-1.105449\H,0,1.740655,1.693176,0.402 511\ H,0,1.541553,0.304299,1.496184\H,0,2.622004,-0.903449,-0.224314\H, 0,-2.757083,-0.545885,0.702188\ H,0,-2.271551,-0.430493,-1.002534\\Vers ion=AM64L-G03RevD.01\State=1-A\HF=-419.7250715\MP2=- 420.9278862\MP3=-420.966904\MP4D=-420.9937121\MP4DQ=-420.9744916\MP4SDQ=-420.9876098\ MP4 SDTQ=-421.0276284\RMSD=5.229e-09\Thermal=0.\PG=C01 [X(C5H10O3)]\\@

003

1\1\GINC-NODE40\SP\RMP4SDTQ-FC\6-31+G(d)\C5H10O3\ZIP06\28-Nov-2011\0\\#p MP4(FC)/ 6-31+G(d) scf=tight\\prc_3\\0,1\C,0,-1.758766,0.804056,-0.0 0314\C,0,-2.098183,-0.700164,0.009976\O,0,-0.849534,-1.418051,-0.07207 1\C,0,0.204838,-0.500602,-0.321593\C,0,-0.264308,0.801394,0.347286\C, 0,1.528757,-1.031522,0.219694\O,0,2.590253,-0.147975,-0.107981\O,0,0.48 4608,1.955904,-0.015408\ H,0,0.325471,-0.318919,-1.407502\H,0,-2.742331 ,-0.987432,-0.830574\H,0,-2.59613,-1.004114,0.937941\ H,0,-0.132261,0.711998,1.431858\H,0,0.423852,2.057851,-0.980229\H,0,1.436007,-1.187592, 1.307448\ H,0,1.764832,-1.998416,-0.235056\H,0,2.302996,0.748787,0.1397 54\H,0,-2.365373,1.389866,0.692811\ H,0,-1.893703,1.229977,-1.006118\\Version=AM64L-G03RevD.01\State=1-A\HF=-419.7233469\MP2= -420.9269779\MP3 =-420.9660063\MP4D=-420.9928289\MP4DQ=-420.9734891\MP4SDQ=-420.9866084 \MP4SDTQ=-421.0267909\RMSD=4.626e-09\Thermal=0.\PG=C01 [X(C5H10O3)]\\@

015

1\1\GINC-NODE13\SP\RMP4SDTQ-FC\6-31+G(d)\C5H10O3\ZIP06\28-Nov-2011\0\\#p MP4(FC)/6-31+G(d) scf=tight\\prc_15\\0,1\C,0,0.965621,-0.650108,0.302696\C,0,1.871115,0.56796,0.052541\C, 0,0.879157,1.722595,0.180073\O,0 ,-0.346012,1.231097,-0.377213\C,0,-0.370112,-0.209157,-0.319818\C,0, -1.613121,-0.632671,0.454157\O,0,-2.787672,-0.097588,-0.127301\O,0,1.381 848,-1.857977,-0.315811\ H,0,1.171651,2.617974,-0.377212\H,0,0.732204,2 .008959,1.233445\H,0,2.158454,-2.186605,0.162028\ H,0,-1.710156,-1.723684,0.437088\H,0,-1.507052,-0.316382,1.507152\H,0,-2.608375,0.851465,-0.239565\ H,0,2.712941,0.641861,0.749601\H,0,0.83729,-0.799458,1.387849\H,0,2.262187,0.502757,-0.969045\H,0, -0.430423,-0.592854,-1.346641\\Version=AM64L-G03RevD.01\State=1-A\HF=-419.7252302\MP2=-420.9281156\MP3=- 420.9670549\MP4D=-420.9938591\MP4DQ=-420.9747062\MP4SDQ=-420.987775\MP 4SDTQ=-421.0277083\RMSD=4.953e-09\Thermal=0.\PG=C01 [X(C5H10O3)]\\@

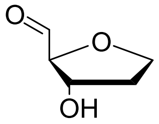

001

1\1\GINC-NODE19\SP\RMP4SDTQ-FC\6-31+G(d)\C5H8O3\ZIP06\28-Nov-2011\0\\#p MP4(FC)/6-31+G(d) scf=tight\\dnac_01\\0,1\C,0,-0.179922,0.822029,0.3 11968\C,0,-1.687147,0.775216,0.067286\C,0,-1.990741,-0.731749,0.136594\O,0,-0.759622,-1.426218,-0.169775\C,0,0.25972,-0.474283,-0.408875\C,0,1.59952,-0.964663,0.087595\O,0,2.55918,-0.220742,0.154487\O,0,0.423362,1.982322,-0.208209\H,0,0.3592 72,-0.240141,-1.484111\H,0,-2.760227,-1.039549,-0.580035\H,0,-2.313542,-1.042028,1.138148\H,0,0.028 719,0.716321,1.38925\H,0,1.383511,1.853376,-0.108422\H,0,1.656178,-2.026845,0.40375\H,0,-2.260017, 1.35813,0.793771\H,0,-1.885839,1.178536,-0.931789\\Version=AM64L-G03RevD.01\State=1-A\HF=-418.5625988\MP2=-419.7515263\MP3=-419.7804094\MP4D=-419.8082897\MP4DQ=-419.7882035\ MP4SDQ=-419.8036404\MP4SDTQ=-419.8474974\RMSD=3.929e-09\Thermal=0.\PG=C01 [X(C5H8O3)]\\@

006

1\1\GINC-NODE12\SP\RMP4SDTQ-FC\6-31+G(d)\C5H8O3\ZIP06\28-Nov-2011\0\\#p MP4(FC)/6-31+G (d) scf=tight\\dnac_06\\0,1\C,0,-0.330017,0.875963,0.244704\C,0,-1.260479,-0.036927,1.060886\C,0,-1.520073,-1.173229,0.070104\O,0,-0.262549,-1.379659,-0.584305\C,0,0.462171,-0.146298,-0.591172\C,0,1.859933,-0.430219,-0.052344\O,0,2.583695,0.405969,0.439907\O,0,-1.050609,1.69827,-0.672437\H,0,0.559001,0.238903,-1.618006\H,0,-2.285872,-0.882948,-0.662621\H,0,-1.811846,-2.118677,0.536103\H,0,0.347733,1.476477,0.862579\H,0,-1.505977,2.382822,-0.157893\H,0,2.168963,-1.492619,-0.153738\H,0,-0.735585,-0.413855,1.947258\H,0,-2.169932,0.477516,1.387934\\Version=AM64L-G03RevD.01\State=1-A\HF=-418.5612728\MP2=-419.7514737\MP3=-419.7799292\MP4D=-419.807997\MP4DQ=-419.7879661\MP4SDQ=-419.8034755\MP4SDTQ=-419.847395\RMSD=8.447e-09\Thermal=0.\PG=C01 [X(C5H8O3)]\\@

002

1\1\GINC-NODE20\SP\RMP4SDTQ-FC\6-31+G(d)\C5H8O3\ZIP06\28-Nov-2011\0\\#p MP4(FC)/6-31+G(d) scf=tight\\dnac_02\\0,1\C,0,0.422114,-0.909371,0.257673\C,0,0.979876,0.181348, 1.192007\C,0,1.108222,1.413895,0.269664\O,0,0.348275,1.121555,-0.925851\C,0,-0.422455,-0.059093,-0.702478\C,0,-1.80857,0.291208,-0.162621\O,0,-2.505727,-0.474389,0.464437 \O,0,1.437377,-1.622497,-0.433209\H,0,-0.554376,-0.565067,-1.668119\H,0,2.141064,1.607676,-0.040625 \H,0,0.72005,2.326857,0.736993\H,0,-0.17906,-1.655325,0.779573\H,0,1.893889,-0.989604,-1.011874 \H,0,-2.137302,1.327077,-0.403631\H,0,0.271203,0.372226,2.005535\H,0,1.930002,-0.129108,1.633657 \\Version=AM64L-G03RevD.01\State=1-A\HF=-418.5588154\MP2=-419.7507119\MP3=-419.7788996\MP4D=-419.8071188\MP4DQ=-419.7868917\MP4SDQ=-419.8025386\MP4SDTQ=-419.846831\RMSD=9.200e-09\Thermal=0.\PG=C01 [X(C5H8O3)]\\@

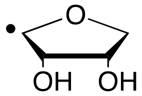

1\1\GINC-NODE16\SP\ROMP2-FC\6-311+G(3df,2p)\C4H7O3(2)\ZIP06\12-Apr-2013\0\\#p ROMP2(FC)/6-311+G(3df,2p) scf=tight geom=check guess=read\\ub3lyp/6-31G(d)\\0,2\C,0,-0.3330582194,-1.2029690854,-0.5173290516\O,0,-1.530939753,-0.5355181437,-0.572143549\C,0,0.6144637518,-0.5736553268,0.4591007208\O,0,1.9828094114,-0.6560980691,0.1235461126\C,0,0.0612970661,0.8789553453, 0.4900281089\O,0,0.7051027694,1.5595585224,-0.5850242019\C,0,-1.4305689111,0.6571324946,0.2295192977\H,0,0.5323958014,2.5085971919,-0.5009061578\H,0,0.5401969431,-1.01305158,1.4669962489\H,0,2.0906419541,-0.0830530278,-0.6558173129\H,0,0.2516221735,1.382445446,1.4460580024\H,0,-1.8986288114,1.4816580346,-0.3168097161\H,0,-1.9770112122,0.4942907479,1.1688970327\H,0,-0.3809580937,-2.2497309299,-0.7931103446\\Version=AM64L-G03RevD.01\State=2-A\HF=-380.1962285\MP2=-381.5534556\RMSD=4.896e-09\Thermal=0.\PG=C01 [X(C4H7O3)]\\@

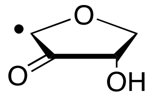

1\1\GINC-NODE22\SP\ROMP2-FC\6-311+G(3df,2p)\C4H5O3(2)\ZIP06\12-Apr-201 3\0\\#p ROMP2(FC)/6-311+G(3df,2p) scf=tight geom=check guess=read\\ub3lyp/6-31G(d)\\0,2\C,0,-0.3825654347,-1.2898443749,-0.3943260177\O,0,-1.5690116414,-0.6723437114,-0.4032748955\C,0,0.6945863322,-0.49

65467742,0.0822938368\O,0,1.8775793278,-0.8136861125,0.2343282123\C,0,0.0938819074,0.908042565,0.348762197\O,0,0.6040271627,1.790506328,-0.6432856749\C,0,-1.4138266005,0.6501719119,0.2126565226\H,0,0.574375329,2.6925179166,-0.2908227294\H,0,0.3621496538,1.2447490944,1.357259292\H,0,-1.9048699382,1.369033595,-0.4456159519\H,0,-1.9335447016,0.6028336777,1.1744883222\H,0,-0.3650855263,-2.3062634957,-0.7655619236\\Version=AM64L-G03RevD.01\State=2-A\HF=-379.0360508\MP2=-380.371303\RMSD=4.848e-09\Th ermal=0.\PG=C01 [X(C4H5O3)]\\@

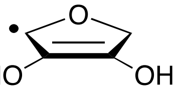

1\1\GINC-NODE25\SP\ROMP2-FC\6-311+G(3df,2p)\C4H5O3(2)\ZIP06\12-Apr-2013\0\\#p ROMP2(FC)/6-311+G(3df,2p) scf=tight geom=check guess=read\\ub3lyp/6-31G(d)\\0,2\C,0,-0.4349752929,-1.3972179778,-0.2239079557\O,0,-1.6527011056,-0.7854058251,-0.0157066808\C,0,0.5962945232,-0.4634158553,-0.2661088749\O,0,1.9091791915,-0.7596238227,-0.4638250163\C,0,0.0487986818,0.7920975365,-0.0758066245\O,0,0.7456800303,1.9668726498,-0.2544660851\C,0,-1.4418204024,0.6420844098,0.0149681053\H,0,0.6016868785,2.5490245303,0.5104196473\H,0,2.3835171749,0.0881835663,-0.5021043973\H,0,-1.9765441518,1.1019662602,-0.8342268112\H,0,-1.8960207285,1.0331147717,0.940180272\H,0,-0.4341809291,-2.4742686237,-0.2780543886\\Version=AM64L-G03RevD.01\State=2-A\HF=-379.0120932\MP2=-380.3552209\RMSD=3.394e-09\Thermal=0.\PG=C01 [X(C4H5O3)]\\@

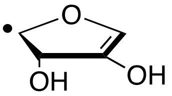

1\1\GINC-NODE20\SP\ROMP2-FC\6-311+G(3df,2p)\C4H5O3(2)\ZIP06\12-Apr-2013\0\\#p ROMP2(FC)/6-311+G(3df,2p) scf=tight geom=check guess=read\\ub3lyp/6-31G(d)\\0,2\C,0,-0.4713510908,-1.3708933834,0.0704476132\O,0,-1.5308599775,-0.6612466643,-0.4258014631\C,0,0.7139863515,-0.5042307443,0.3462859435\O,0,1.8898272232,-0.8003112481,-0.433212113\C,0,0.0951883948,0.8468419045,0.0579437681\O,0,0.8342922011,1.97030061,0.2565004085\C,0,-1.1567387608,0.6824020829,-0.3950612338\H,0,0.3047765907,2.7484068,0.0199998179\H,0,1.0859978644,-0.5744336656,1.3779890716\H,0,1.6185699776,-0.7761003958,-1.3668059499\H,0,-1.915881275,1.378034336,-0.7235365577\H,0,-0.5436246192,-2.4467470019,0.0138018747\\Version=AM64L-G03RevD.01\State=2-A\HF=-379.0029169\MP2=-380.3354956\RMSD=3.719e-09\Th rmal=0.\PG=C01 [X(C4H5O3)]\\@

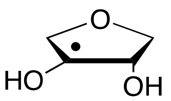

1\1\GINC-NODE14\SP\ROMP2-FC\6-311+G(3df,2p)\C4H7O3(2)\ZIP06\12-Apr-2013\0\\#p ROMP2(FC)/6-311+G(3df,2p) scf=tight geom=check guess=read\\ub3lyp/6-31G(d)\\0,2\C,0,-0.4598311141,-1.4352735938,-0.3051217679\O,0,-1.6401415585,-0.7659122456,0.1551649124\C,0,0.6615047198,-0.5090353545,0.0777876818\O,0,1.8283338487,-0.527403509,-0.6214579797\C,0,0.0773647774,0.8257699898,0.4770476716\O,0,0.7636441144,1.8544873136,-0.252110854\C,0,-1.3980542976,0.635015496,0.0662229059\H,0,0.8056569799,2.6504425876,0.2972830931\H,0,-0.4804668281,-1.5898614682,-1.4005628854\H,0,2.1253539213,0.4046628211,-0.6673602469\H,0,0.1703417822,1.0215491772,1.5572960042\H,0,-1.5131005799,1.0076172616,-0.9648458004\H,0,-0.437849461,-2.4203875505,0.1755961816\H,0,-2.1244703048,1.1356710746,0.7132880838\\Version=AM64L-G03RevD.01\State=2-A\HF=-380.1960275\MP2=-381.551897\RMSD=1.990e-09\Thermal=0.\PG=C01 [X(C4H7O3)]\\@

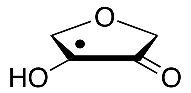

1\1\GINC-NODE18\SP\ROMP2-FC\6-311+G(3df,2p)\C4H5O3(2)\ZIP06\12-Apr-2013\0\\#p ROMP2(FC)/6-311+G(3df,2p) scf=tight geom=check guess=read\\ub3lyp/6-31G(d)\\0,2\C,0,-0.4652995743,-1.4053420613,-0.5165896919\O,0,-1.6751414608,-0.6807049797,-0.2569768591\C,0,0.6180290597,-0.4293370205,-0.2404207043\O,0,1.9220482879,-0.6677962987,-0.3496696435\C,0,0.0965393895,0.8254829797,0.1652593572\O,0,0.7954643983,1.8118625029,0.4547031435\C,0,-1.4163054471,0.6687502092,0.1563310239\H,0,-0.4570929139,-1.7626339466,-1.5587861606\H,0,2.3575095898,0.1779131709,-0.0970284119\H,0,-1.8886600608,1.3713364265,-0.5438536435\H,0,-0.4114597726,-2.2925922,0.1343147957\H,0,-1.8439459558,0.8428015276,1.1532330347\\Version=AM64L-G03RevD.01\State=2-A\HF=-379.0471936\MP2=-380.3789169\RMSD=6.493e-09\Thermal=0.\PG=C01 [X(C4H5O3)]\\@

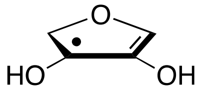

1\1\GINC-NODE23\SP\ROMP2-FC\6-311+G(3df,2p)\C4H5O3(2)\ZIP06\12-Apr-2013\0\\#p ROMP2(FC)/6-311+G(3df,2p) scf=tight geom=check guess=read\\ub3lyp/6-31G(d)\\0,2\C,0,-0.4485388982,-1.4287821004,-0.440313916\O,0,-1.6509392504,-0.6315175067,-0.3533428447\C,0,0.6606050441,-0.4316835042,-0.3408236272\O,0,1.9703655039,-0.8045642886,-0.3306133573\C,0,0.1038196614,0.8277593294,-0.2360441714\O,0,0.8735427792,1.9630457924,-0.1532188425\C,0,-1.2822377972,0.6957697107,-0.2514018465\H,0,0.3467061536,2.6747479151,0.2398538399\H,0,-0.4654190171,-1.9887183036,-1.3901799066\H,0,2.5006877865,0.0025164024,-0.2176562453\H,0,-2.0831804431,1.4193924497,-0.2402386202\H,0,-0.4503075226,-2.167162896,0.3777525376\\Version=AM64L-G03RevD.01\State=2-A\HF=-379.0128885\MP2=-380.3571748\RMSD=3.589e-09\Thermal=0.\PG=C01 [X(C4H5O3)]\\@

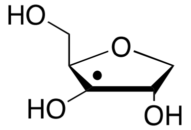

001

1\1\GINC-NODE3\SP\ROMP2-FC\6-311+G(3df,2p)\C5H9O4(2)\ZIP06\20-Feb-2013\0\\#p ROMP2(FC)/6-311+G(3df,2p) scf=tight\\Rad3Ribo_001\\0,2\O,0,2.677944,0.436727,-0.164106\C,0,1.962261,-0.791017,0.057928\C,0,0.534992,-0.511626,-0.411952\O,0,-0.296365,-1.644556,-0.201511\C,0,-0.127107,0.594262,0.357009\O,0,0.303035,1.884813,0.278719\C,0,-1.581057,0.291132,0.447821\O,0,-2.316066,0.773937,-0.696976\C,0,-1.549791,-1.2404,0.390597\H,0,-2.085951,1.712458,-0.79463\H,0,1.94846,-1.059573,1.122501\H,0,2.408708,-1.615822,-0.513745\H,0,0.577822,-0.269919,-1.492242\H,0,1.265209,1.832667,0.1031\H,0,-2.051969,0.662279,1.369804\H,0,-2.373266,-1.618782,-0.223514\H,0,-1.621059,-1.676124,1.395366\H,0,3.547868,0.371344,0.255941\\Version=AM64L-G03RevD.01\State=2-A\HF=-494.1334404\MP2=-495.8984312\RMSD=2.222e-09\Thermal=0.\PG=C01 [X(C5H9O4)]\\@

022

1\1\GINC-NODE6\SP\ROMP2-FC\6-311+G(3df,2p)\C5H9O4(2)\ZIP06\20-Feb-2013\0\\#p ROMP2(FC)/6-311+G(3df,2p) scf=tight\\Rad3Ribo_022\\0,2\O,0,3.07889,-0.049447,-0.175579\C,0,1.965068,0.543415,

0.464996\C,0,0.691714,0.350307,-0.3667\O,0,0.498582,-1.072429,-0.592336\C,0,-0.591135,0.792508,
0.28282\O,0,-1.162701,1.96847,-0.101394\C,0,-1.460438,-0.40345,0.557659\O,0,-2.585906,-0.435959,-
0.341344\C,0,-0.453604,-1.545586,0.371614\H,0,-2.224708,-0.510346,-1.241896\H,0,1.808143,0.131333,
1.476371\H,0,2.179153,1.611932,0.572456\H,0,0.837773,0.816055,-1.34978\H,0,-2.129725,1.840194,-
0.070937\H,0,-1.919412,-0.414521,1.552408\H,0,-0.918391,-2.452893,-0.023482\H,0,0.037605,-1.784667,
1.326766\H,0,2.789007,-0.945323,-0.419005\\Version=AM64L-G03RevD.01\State=2-A\HF=-494.1308571\
MP2=-495.8977324\RMSD=1.340e-09\Thermal=0.\PG=C01 [X(C5H9O4)]\\@

010

1\1\GINC-NODE8\SP\ROMP2-FC\6-311+G(3df,2p)\C5H9O4(2)\ZIP06\20-Feb-2013\0\\#p ROMP2(FC)/6-
311+G(3df,2p) scf=tight\\Rad3Ribo_010\\0,2\O,0,3.182134,-0.054746,-0.119094\C,0,2.048883,0.622085,
0.395818\C,0,0.769134,0.187404,-0.315512\O,0,0.643548,-1.235454,-0.102919\C,0,-0.521896,0.737656,
0.221337\O,0,-1.009535,1.915647,-0.248718\C,0,-1.484781,-0.39385,0.48835\O,0,-2.737783,-0.076236,
-0.134643\C,0,-0.738787,-1.576657,-0.164158\H,0,-3.45538,-0.439447,0.404207\H,0,1.924784,0.450871,
1.477062\H,0,2.213783,1.692279,0.235865\H,0,0.870267,0.401595,-1.395105\H,0,-1.983513,1.813597,
-0.268058\H,0,-1.646961,-0.566434,1.56431\H,0,-1.08962,-1.667991,-1.203931\H,0,-0.864903,-2.536632,
0.345307\H,0,2.969311,-1.001345,-0.071681\\Version=AM64L-G03RevD.01\State=2-A\HF=-494.1318614\
MP2=-495.8968379\RMSD=4.211e-09\Thermal=0.\PG=C01[X(C5H9O4)] \\@

004

1\1\GINC-NODE11\SP\ROMP2-FC\6-311+G(3df,2p)\C5H9O4(2)\ZIP06\20-Feb-2013\0\\#p ROMP2(FC)/
6-311+G(3df,2p) scf=tight\\Rad3Ribo_004\\0,2\O,0,2.439175,0.029277,1.069799\C,0,2.204267,0.466254,-
0.254282\C,0,0.78281,0.128899,-0.713952\O,0,0.619374,-1.293806,-0.517801\C,0,-0.342163,0.724178,0.0
83625\O,0,-0.848055,1.938005,-0.259513\C,0,-1.281058,-0.357734,0.545174\O,0,-2.623028,0.030144,0.2
14552\C,0,-0.760377,-1.562248,-0.266386\H,0,-3.21967,-0.294158,0.904614\H,0,2.344681,1.551635,-0.27
3233\H,0,2.920226,0.015789,-0.96171\H,0,0.676094,0.388859,-1.784013\H,0,-1.806943,1.891524,-0.06694
9\H,0,-1.21271,-0.543652,1.629274\H,0,-1.335699,-1.615729,-1.203074\H,0,-0.819418,-2.525925,0.248609
\H,0,2.132842,-0.893383,1.085107\\Version=AM64L-G03RevD.01\State=2-A\HF=-494.1307779\MP2=-49
5.8966704\RMSD=3.645e-09\Thermal=0.\PG=C01 [X(C5H9O4)]\\@

009

1\1\GINC-NODE26\SP\ROMP2-FC\6-311+G(3df,2p)\C5H9O4(2)\ZIP06\20-Feb-2013\0\\#p ROMP2(FC)/
6-311+G(3df,2p) scf=tight\\Rad3Ribo_009\\0,2\O,0,2.475628,0.060449,1.003259\C,0,2.192297,0.420575,-
0.332854\C,0,0.750633,0.073733,-0.718994\O,0,0.578548,-1.333765,-0.446203\C,0,-0.334168,0.710324,0.
094779\O,0,-0.749584,1.975246,-0.192206\C,0,-1.325053,-0.316651,0.566415\O,0,-2.675526,0.040486,0.2
41267\C,0,-0.791098,-1.584786,-0.136264\H,0,-2.754077,0.029457,-0.728444\H,0,2.340083,1.501576,-0.4
21733\H,0,2.87449,-0.077937,-1.041427\H,0,0.613815,0.278753,-1.799514\H,0,-1.664863,2.046588,0.141
932\H,0,-1.346225,-0.433264,1.65783\H,0,-1.376009,-1.755834,-1.057509\H,0,-0.835414,-2.492871,0.470
996\H,0,2.159998,-0.854971,1.090438\\Version=AM64L-G03RevD.01\State=2-A\HF=-494.1299283\MP2=
-495.896039\RMSD=2.248e-09\Thermal=0.\PG=C01 [X(C5H9O4)]\\@

026

1\1\GINC-NODE27\SP\ROMP2-FC\6-311+G(3df,2p)\C5H9O4(2)\ZIP06\20-Feb-2013\0\\#p ROMP2(FC)/
6-311+G(3df,2p) scf=tight\\Rad3Ribo_026\\0,2\O,0,-2.452368,-0.201044,-0.856456\C,0,-2.094889,
0.61267,0.247356\C,0,-0.652695,0.345652,0.712338\O,0,-0.464971,-1.079547,0.870436\C,0,0.43
927,0.76115,-0.232421\O,0,0.976237,2.008428,-0.103627\C,0,1.296716,-0.409399,-0.592752\O,0,2.572983,
-0.361084,0.080263\C,0,0.382919,-1.569565,-0.183258\H,0,2.390738,-0.364315,1.035729\H,0,-2.191067,
1.65457,-0.074773\H,0,-2.779617,0.453273,1.095539\H,0,-0.512527,0.822132,1.696758\H,0,1.914627,1.9
49984,-0.359916\H,0,1.568665,-0.461867,-1.653046\H,0,0.946954,-2.42491,0.200502\H,0,-0.220927,-1.89
1885,-1.042779\H,0,-2.39982,-1.114061,-0.530518\\Version=AM64L-G03RevD.01\State=2-A\HF=-494.1
292303\MP2=-495.8954764\RMSD=5.344e-09\Thermal=0.\PG=C01 [X(C5H9O4)]\\@

007

1\1\GINC-NODE10\SP\ROMP2-FC\6-311+G(3df,2p)\C5H9O4(2)\ZIP06\20-Feb-2013\0\\#p ROMP2(FC)/
6-311+G(3df,2p) scf=tight\\Rad3Ribo_007\\0,2\O,0,-2.877507,-0.669737,-0.007608\C,0,
-2.140533,0.508688,0.272322\C,0,-0.7523,0.456583,-0.374565\O,0,-0.071171,1.692103,-0.115965\C,0,0.17
5304,-0.578357,0.203116\O,0,0.116266,-1.872144,-0.216945\C,0,1.515383,0.041755,0.494205\O,0,2.5255
89,-0.768235,-0.125805\C,0,1.331396,1.436962,-0.142426\H,0,3.341098,-0.69984,0.391961\H,0,-2.022367,
0.681693,1.353154\H,0,-2.713193,1.341786,-0.146089 \H,0,-0.880001,0.291956,-1.460794\H,0,1.0387,-
2.201558,-0.216974\H,0,1.723971,0.116232,1.573768\H,0,1.71694,1.39212,-1.173201\H,0,1.824752,2.2
53581,0.392842\H,0,-2.350809,-1.425649,0.300005\\Version=AM64L-G03RevD.01\State=2-A\HF=-494.
1301448\MP2=-495.8948468\RMSD=4.059e-09\Thermal=0.\PG=C01 [X(C5H9O4)]\\@

028

1\1\GINC-NODE20\SP\ROMP2-FC\6-311+G(3df,2p)\C5H9O4(2)\ZIP06\20-Feb-2013\0\\#p ROMP2(FC)/
6-311+G(3df,2p) scf=tight\\Rad3Ribo_028\\0,2\O,0,-2.446268,-0.226551,1.078895\C,0,-2.2393,-0.202558,
-0.320476\C,0,-0.794067,0.109994,-0.713687\O,0,-0.489957,1.466648,-0.359902\C,0,0.256646,-0.692105,
0.020005\O,0,0.667488,-1.884664,-0.485689\C,0,1.301719,0.250231,0.575338\O,0,2.595675,-0.253169,0.
219377\C,0,0.910926,1.563899,-0.133393\H,0,3.221392,-0.037247,0.925492\H,0,-2.518131,-1.159741,-0.7
92388\H,0,-2.896961,0.575397,-0.720143\H,0,-0.684488,-0.041215,-1.805249\H,0,1.623997,-1.946729,-0.2
80354\H,0,1.232283,0.354066,1.668848\H,0,1.477809,1.621329,-1.07684\H,0,1.089644,2.470927,0.45138
4\H,0,-1.776585,-0.831675,1.441088\\Version=AM64L-G03RevD.01\State=2-A\HF=-494.1287585\MP2=-
495.8946429\RMSD=1.745e-09\Thermal=0.\PG=C01 [X(C5H9O4)]\\@

001

1\1\GINC-NODE18\SP\ROMP2-FC\6-311+G(3df,2p)\C5H7O4(2)\ZIP06\20-Feb-2013\0\\#p ROMP2(FC)/
6-311+G(3df,2p) scf=tight\\R3Ket2Ribo_001\\0,2\O,0,2.078942,0.114259, 1.226893\C,0,2.092648,
0.351389,-0.163793\C,0,0.756056,-0.033889,-0.812317\O,0,0.486075,-1.415028,-0. 481993\C,0,-0.440661,

0.681745,-0.29511\O,0,-0.592467,2.001925,-0.29519\C,0,-1.441255,-0.213235,0.158789\O,0,-2.547394, 0.132242,0.606738\C,0,-0.874413,-1.609425,-0.057765\H,0,2.895324,-0.209201,-0.668341\H,0,2.275746, 1.420327,-0.312169\H,0,0.846141,0.080751,-1.907228\H,0,-1.480539,2.162295,0.096016\H,0,-1.44259,-2.152336,-0.827055\H,0,-0.882246,-2.207806,0.860438\H,0,1.832658,-0.82073,1.327917\\Version= AM64L-G03RevD.01\State=2-A\HF=-492.9825188\MP2=-494.7248278\RMSD=5.688e-09\Thermal= 0.\PG=C01 [X(C5H7O4)]\\@

003

1\1\GINC-NODE17\SP\ROMP2-FC\6-311+G(3df,2p)\C5H7O4(2)\ZIP06\20-Feb-2013\0\\#p ROMP2(FC)/ 6-311+G(3df,2p) scf=tight\\R3Ket2Ribo_003\\0,2\O,0,3.025424,-0.026227,0.189696\C,0,1.815056, 0.645685,0.464409\C,0,0.71528,0.183649,-0.506399\O,0,0.585399,-1.252818,-0.386617\C,0,-0.648161,0.686281,-0.210963\O,0,-1.010225,1.966624,-0.215005\C,0,-1.544351,-0.364548,0.113774\O,0,-2.737603,-0.198588,0.416633\C,0,-0.738491,-1.650686,0.006423\H,0,1.999253,1.717157,0.336886\H,0,1.473112,0.473434,1.497744\H,0,1.041204,0.417563,-1.531425\H,0,-1.962193,1.966232,0.03448\H,0,-1.159552,-2.327458, -0.748579\H,0,-0.70393,-2.186319,0.964418\H,0,2.812142,-0.974821,0.205353\\Version=AM64L-G03RevD.01\State=2-A\HF=-492.9817658\MP2=-494.7235412\RMSD=8.359e-09\Thermal=0.\PG=C01 [X(C5H7O4)] \\@

002

1\1\GINC-NODE19\SP\ROMP2-FC\6-311+G(3df,2p)\C5H7O4(2)\ZIP06\20-Feb-2013\0\\#p ROMP2(FC)/ 6-311+G(3df,2p) scf=tight\\R3Ket2Ribo_002\\0,2\O,0,1.726428,0.150395,1.320771\C,0,2.037552,0.332562,-0.051262\C,0,0.790143,-0.014962,-0.868944\O,0,0.469423,-1.410959,-0.755468\C,0,-0.448554,0.662914,-0.395406\O,0,-0.668612,1.974786,-0.426867\C,0,-1.359594,-0.258794,0.179926\O,0,-2.448893,0.058027,0.686346\C,0,-0.695206,-1.621881,0.057463\H,0,2.851354,-0.330657,-0.379293\H,0,2.325082,1.37338,-0.273064\H,0,1.009178,0.20232,-1.925771\H,0,-1.545144,2.09514,0.004076\H,0,-1.343002, -2.35605,-0.435316\H,0,-0.412804,-2.006738,1.047003\H,0,2.542518,0.245591,1.83346\\Version=AM64L-G03RevD.01\State=2-A\HF=-492.9810175\MP2=-494.722173\RMSD=2.541e-09\Thermal=0.\PG=C01 [X (C5H7O4)]\\@

008

1\1\GINC-NODE12\SP\ROMP2-FC\6-311+G(3df,2p)\C5H7O4(2)\ZIP06\20-Feb-2013\0\\#p ROMP2(FC)/ 6-311+G(3df,2p) scf=tight\\R3Ket2Ribo_008\\0,2\O,0,2.801298,0.464833,0.117697\C,0,1.896588,-0.569194,0.452528\C,0,0.661362,-0.558625,-0.472842\O,0,-0.163971,-1.705001,-0.202766\C,0,-0.247745, 0.591553,-0.240916\O,0,0.116184,1.875897,-0.275564\C,0,-1.561735,0.17506,0.090368\O,0,-2.497213,0.950811,0.34924\C,0,-1.530785,-1.34569,0.051969\H,0,1.562541,-0.519358,1.500031\H,0,2.426006,-1.515468,0.31142\H,0,1.02347,-0.591079,-1.513177\H,0,-0.696324,2.381688,-0.044584\H,0,-2.18148,-1.737564,-0.742757\H,0,-1.854078,-1.783392,1.004612\H,0,2.363368,1.31423,0.288938\\Version=AM64L-G03RevD.01\State=2-A\HF=-492.9804872\MP2=-494.721688\RMSD=6.276e-09\Thermal=0.\PG=C01 [X(C5H7O4)] \\@

006

1\1\GINC-NODE27\SP\ROMP2-FC\6-311+G(3df,2p)\C5H7O4(2)\ZIP06\20-Feb-2013\0\\#p ROMP2(FC)/ 6-311+G(3df,2p) scf=tight\\R3Ket2Ribo_006\\0,2\O,0,2.775692,0.531468,0.035886\C,0,1.911905,-0.511569,0.452997\C,0,0.680917,-0.474827,-0.460821\O,0,-0.084834,-1.67247,-0.234536\C,0,-0.289085, 0.622484,-0.197241\O,0,-0.03453,1.924824,-0.25772\C,0,-1.580325,0.118227,0.105945\O,0,-2.568476,0.831262,0.353058\C,0,-1.466108,-1.395721,0.038153\H,0,1.590631,-0.398045,1.499343\H,0,2.372484,-1.505097,0.343571\H,0,1.041802,-0.449323,-1.502182\H,0,-0.893362,2.358129,-0.047001\H,0,-2.1006,-1.806211,-0.760218\H,0,-1.756796,-1.872457,0.983025\H,0,3.4992,0.600769,0.675756\\Version=AM64L-G03RevD.01\State=2-A\HF=-492.9783472\MP2=-494.7191816\RMSD=3.905e-09\Thermal=0.\PG=C01 [X(C5H7O4)]\\@

007

1\1\GINC-NODE20\SP\ROMP2-FC\6-311+G(3df,2p)\C5H7O4(2)\ZIP06\20-Feb-2013\0\\#p ROMP2(FC)/ 6-311+G(3df,2p) scf=tight\\R3Ket2Ribo_007\\0,2\O,0,2.772661,0.565983,0.280714\C,0,1.898568,-0.530637,0.461038\C,0,0.680707,-0.479311,-0.486615\O,0,-0.096989,-1.67552,-0.300772\C,0,-0.28248,0.621109,-0.223932\O,0,-0.022845,1.920983,-0.313563\C,0,-1.566304,0.123064,0.118591\O,0,-2.546729,0.839576,0.383543\C,0,-1.455931,-1.39221,0.067985\H,0,1.551197,-0.486388,1.497103\H,0,2.39653,-1.50037, 0.31256\H,0,1.051608,-0.447164,-1.526609\H,0,-0.869791,2.365404,-0.080309\H,0,-2.140954,-1.818093,-0.677594\H,0,-1.681985,-1.850545,1.039857\H,0,3.197257,0.47689,-0.586789\\Version=AM64L-G03RevD.01\State=2-A\HF=-492.9777706\MP2=-494.7190198\RMSD=4.742e-09\Thermal=0.\PG=C01 [X(C5H7O4)]\\@

010

1\1\GINC-NODE3\SP\ROMP2-FC\6-311+G(3df,2p)\C5H7O4(2)\ZIP06\20-Feb-2013\0\\#p ROMP2(FC)/6-311+G(3df,2p) scf=tight\\R3Ket2Ribo_010\\0,2\O,0,3.082815,-0.099055,0.216575\C,0,1.839515, 0.516055,0.482663\C,0,0.719709,0.045231,-0.465278\O,0,0.466877,-1.359644,-0.308102\C,0,-0.598103, 0.678331,-0.199356\O,0,-0.844019,1.986764,-0.2254\C,0,-1.595241,-0.284247,0.102401\O,0,-2.776856,-0.005785,0.367171\C,0,-0.903524,-1.635443,0.016259\H,0,1.898797,1.615594,0.429192\H,0,1.574249,0.237459,1.50648\H,0,1.051046,0.23269,-1.503557\H,0,-1.798195,2.071879,0.001179\H,0,-1.354325,-2.264937,-0.763435\H,0,-0.959284,-2.180923,0.967499\H,0,3.383051,0.190428,-0.659438\\Version=AM64L-G03RevD.01\State=2-A\HF=-492.9775378\MP2=-494.7183161\RMSD=3.764e-09\Thermal=0.\PG=C01 [X(C5H7O4)]\\@

011

1\1\GINC-NODE6\SP\ROMP2-FC\6-311+G(3df,2p)\C5H7O4(2)\ZIP06\20-Feb-2013\0\\#p ROMP2(FC)/6-311+G(3df,2p) scf=tight\\R3Ket2Ribo_011\\0,2\O,0,2.6488,0.430963,0.001565\C,0,1.849933,-0.690994,0.413971\C,0,0.575716,-0.612453,-0.431\O,0,-0.293926,-1.689444,-0.097923\C,0,-0.217407,0.632433,-0.172217\O,0,0.307831,1.857372,-0.18268\C,0,-1.586562,0.311712,0.07167\O,0,-2.538677,1.066392,0.29266\C,0,-1.649289,-1.219113,-0.003294\H,0,1.587627,-0.629634,1.478069\H,0,2.370859,-1.638923,0.228722\H,0,0.877973,-0.670211,-1.493626\H,0,1.284936,1.747959,-0.223829\H,0,-2.229659,-1.543594,-0.

879594\H,0,-2.111323,-1.647752,0.892676\H,0,3.393011,0.530379,0.613832\\Version=AM64L-G03RevD.01\State=2-A\HF=-492.9767641\MP2=-494.718633\RMSD=7.561e-09\Thermal=0.\PG=C01 [X(C5H7O4)]\\@

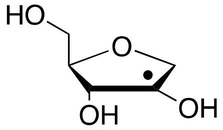

002

1\1\GINC-NODE19\SP\ROMP2-FC\6-311+G(3df,2p)\C5H9O4(2)\ZIP06\20-Feb-2013\0\\#p ROMP2(FC)/6-311+G(3df,2p) scf=tight\\Rad2Ribo_002\\0,2\O,0,-2.541594,0.10976,-0.916011\C,0,-2.148277,0.399246,0.415053\C,0,-0.690494,0.029545,0.652863\O,0,-0.565095,-1.369254,0.35849\C,0,0.335077,0.719045,-0.273039\O,0,0.965009,1.863973,0.320282\C,0,1.367217,-0.368263,-0.453233\O,0,2.678106,-0.060007,-0.259345\C,0,0.818844,-1.655339,0.097014\H,0,2.702577,0.909878,-0.122774\H,0,-2.78085,-0.128923,1.1455\H,0,-2.287596,1.475528,0.566957\H,0,-0.40218,0.240513,1.695778\H,0,-0.151499,1.005239,-1.216763\H,0,0.60899,2.661393,-0.09701\H,0,1.347558,-1.942689,1.023352\H,0,0.847187,-2.509638,-0.589859\H,0,-2.269806,-0.812473,-1.064462\\Version=AM64L-G03RevD.01\State=2-A\HF=-494.1328567\MP2=-495.8989489\RMSD=4.917e-09\Thermal=0.\PG=C01 [X(C5H9O4)]\\@

001

1\1\GINC-NODE21\SP\ROMP2-FC\6-311+G(3df,2p)\C5H9O4(2)\ZIP06\20-Feb-2013\0\\#p ROMP2(FC)/6-311+G(3df,2p) scf=tight\\Rad2Ribo_001\\0,2\O,0,2.620761,0.573888,-0.179938\C,0,2.020024,-0.713957,0.0126\C,0,0.551964,-0.521852,-0.319451\O,0,-0.169761,-1.716494,-0.044609\C,0,-0.148543,0.54958,0.541446\O,0,0.014953,1.880798,0.090286\C,0,-1.590267,0.11933,0.379829\O,0,-2.377514,0.90298,-0.415688\C,0,-1.5705,-1.360413,0.082111\H,0,-1.930765,1.77188,-0.465172\H,0,2.115152,-1.050856,1.055026\H,0,2.476489,-1.470671,-0.640407\H,0,0.450866,-0.222284,-1.376821\H,0,0.187027,0.441039,1.586974\H,0,0.968951,1.997018,-0.064258\H,0,-2.11724,-1.565641,-0.850484\H,0,-1.980483,-1.995693,0.875233\H,0,3.546419,0.529707,0.100294\\Version=AM64L-G03RevD.01\State=2-A\HF=-494.1328171\MP2=-495.8981496\RMSD=3.754e-09\Thermal=0.\PG=C01 [X(C5H9O4)]\\@

037

1\1\GINC-NODE19\SP\ROMP2-FC\6-311+G(3df,2p)\C5H9O4(2)\ZIP06\20-Feb-2013\0\\#p ROMP2(FC)/6-311+G(3df,2p) scf=tight\\Rad2Ribo_037\\0,2\O,0,-2.497543,0.05874,-0.998052\C,0,-2.169471,0.439282,0.325529\C,0,-0.72373,0.088789,0.655708\O,0,-0.5823,-1.325159,0.46218\C,0,0.343534,0.745487,-0.250989\O,0,0.970104,1.912624,0.296845\C,0,1.322317,-0.38485,-0.447602\O,0,2.660114,-0.12105,-0.424229\C,0,0.797773,-1.626419,0.208903\H,0,2.745022,0.850392,-0.490799\H,0,-2.309,1.523042,0.396044\H,0,-2.834849,-0.040799,1.060436\H,0,-0.504879,0.354919,1.707286\H,0,-0.111702,1.093888,-1.18542\H,0,1.253752,1.694591,1.201495\H,0,1.334883,-1.848577,1.150667\H,0,0.826179,-2.530431,-0.411432\H,0,-2.224949,-0.872007,-1.071527\\Version=AM64L-G03RevD.01\State=2-A\HF=-494.1315266\MP2=-495.8976813\RMSD=4.074e-09\Thermal=0.\PG=C01 [X(C5H9O4)]\\@

003

1\1\GINC-NODE17\SP\ROMP2-FC\6-311+G(3df,2p)\C5H9O4(2)\ZIP06\20-Feb-2013\0\\#p ROMP2(FC)/6-311+G(3df,2p) scf=tight\\Rad2Ribo_003\\0,2\O,0,3.128656,-0.041671,-0.267115\C,0,2.034804,0.632741,0.325678\C,0,0.707031,0.147585,-0.233566\O,0,0.634941,-1.259418,0.037526\C,0,-0.569908,0.741146,0.398799\O,0,-1.15531,1.809834,-0.358269\C,0,-1.506529,-0.442786,0.355222\O,0,-2.77588,-0.255274,-0.094537\C,0,-0.736648,-1.665152,-0.060569\H,0,-2.864226,0.707518,-0.250735\H,0,2.025853,0.504252,1.421786\H,0,2.164227,1.699505,0.10988\H,0,0.664305,0.331142,-1.319065\H,0,-0.375144,1.087162,1.428126\H,0,-0.912698,2.65276,0.051436\H,0,-0.997345,-1.961513,-1.093166\H,0,-0.863663,-2.5407,0.58701\H,0,2.926943,-0.989093,-0.189505\\Version=AM64L-G03RevD.01\State=2-A\HF=-494.1316183\MP2=-495.8964631\RMSD=5.874e-09\Thermal=0.\PG=C01 [X(C5H9O4)]\\@

030

1\1\GINC-NODE21\SP\ROMP2-FC\6-311+G(3df,2p)\C5H9O4(2)\ZIP06\20-Feb-2013\0\\#p ROMP2(FC)/6-311+G(3df,2p) scf=tight\\Rad2Ribo_030\\0,2\O,0,-3.040546,-0.071176,-0.114499\C,0,-1.867117,0.441781,-0.712749\C,0,-0.733074,0.519911,0.313187\O,0,-0.583509,-0.770436,0.948953\C,0,0.639099,0.853146,-0.294422\O,0,1.493714,1.660862,0.538294\C,0,1.227612,-0.516342,-0.497751\O,0,2.582751,-0.662637,-0.514194\C,0,0.384918,-1.542316,0.206431\H,0,2.9628,0.235118,-0.454363\H,0,-1.548233,-0.172637,-1.573372\H,0,-2.101407,1.443006,-1.091249\H,0,-1.00529,1.22817,1.102469\H,0,0.547091,1.442139,-1.215074\H,0,1.521362,1.235477,1.412953\H,0,0.969501,-2.141687,0.915377\H,0,-0.131679,-2.234202,-0.477114\H,0,-2.742056,-0.825371,0.42375\\Version=AM64L-G03RevD.01\State=2-A\HF=-494.1287821\MP2=-495.8962149\RMSD=2.961e-09\Thermal=0.\PG=C01 [X(C5H9O4)]\\@

004

1\1\GINC-NODE11\SP\ROMP2-FC\6-311+G(3df,2p)\C5H9O4(2)\ZIP06\20-Feb-2013\0\\#p ROMP2(FC)/6-311+G(3df,2p) scf=tight\\Rad2Ribo_004\\0,2\O,0,2.379562,-0.094622,1.141049\C,0,2.196352,0.422399,-0.16592\C,0,0.778956,0.178397,-0.664255\O,0,0.5778,-1.25324,-0.640608\C,0,-0.342242,0.803594,0.196102\O,0,-0.949375,1.85553,-0.566444\C,0,-1.250981,-0.366876,0.423391\O,0,-2.561362,-0.077395,0.701438\C,0,-0.808309,-1.511745,-0.442786\H,0,-3.070353,-0.899311,0.778134\H,0,2.390257,1.498916,-0.119884\H,0,2.909888,-0.022671,-0.87757\H,0,0.658125,0.557516,-1.689603\H,0,0.057,1.206638,1.13878\H,0,-1.759091,2.110054,-0.09527\H,0,-1.349639,-1.532793,-1.410706\H,0,-0.905707,-2.50527,0.015966\H,0,2.053856,-1.009883,1.097479\\Version=AM64L-G03RevD.01\State=2-A\HF=-494.1316741\MP2=-495.8953572\RMSD=2.325e-09\Thermal=0.\PG=C01 [X(C5H9O4)]\\@

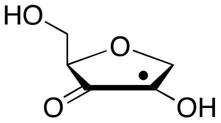

005

1\1\GINC-NODE11\SP\ROMP2-FC\6-311+G(3df,2p)\C5H7O4(2)\ZIP06\20-Feb-2013\0\\#p ROMP2(FC)/6-311+G(3df,2p) scf=tight\\R2Ket3Ribo_005\\0,2\O,0,1.710499,0.116752,1.340732\C,0,2.048676,0.350775,-0.020225\C,0,0.83167,0.000358,-0.869401\O,0,0.497348,-1.392881,-0.758553\C,0,-0.421869,0.

747824,-0.418304\O,0,-0.666108,1.966086,-0.452141\C,0,-1.290369,-0.236763,0.111971\O,0,-2.467766, 0.052679,0.659824\C,0,-0.67665,-1.587859,0.042714\H,0,2.896882,-0.273281,-0.3391\H,0,2.300265,1.40 7211,-0.204473\H,0,1.063524,0.213702,-1.921244\H,0,-1.320143,-2.333225,-0.44478\H,0,-0.408614,-1.96 6721,1.041754\H,0,2.480201,0.33572,1.886092\H,0,-2.552646,1.029501,0.582334\\Version=AM64L-G03 RevD.01\State=2-A\HF=-492.9813416\MP2=-494.7230698\RMSD=3.299e-09\Thermal=0.\PG=C01 [X(C5 H7O4)]\\@

007

1\1\GINC-NODE21\SP\ROMP2-FC\6-311+G(3df,2p)\C5H7O4(2)\ZIP06\20-Feb-2013\0\\#p ROMP2(FC)/ 6-311+G(3df,2p) scf=tight\\R2Ket3Ribo_007\\0,2\O,0,2.071063,0.106871,1.24788\C,0,2.105784,0.380985, -0.13875\C,0,0.794373,-0.016772,-0.816279\O,0,0.512123,-1.397151,-0.490093\C,0,-0.418141,0.762963,- 0.322382\O,0,-0.602954,1.991177,-0.306648\C,0,-1.362539,-0.199053,0.112999\O,0,-2.56467,0.117881, 0.585342\C,0,-0.850044,-1.580909,-0.069369\H,0,2.942818,-0.135655,-0.634225\H,0,2.247649,1.460018,- 0.255278\H,0,0.89228,0.094899,-1.907682\H,0,-1.41641,-2.139602,-0.833029\H,0,-0.866653,-2.175546, 0.854717\H,0,1.856772,-0.838442,1.317995\H,0,-2.597554,1.100824,0.568332\\Version=AM64L-G03 RevD.01\State=2-A\HF=-492.9822208\MP2=-494.7245314\RMSD=5.635e-09\Thermal=0.\PG=C01 [X(C5 H7O4)]\\@

008

1\1\GINC-NODE17\SP\ROMP2-FC\6-311+G(3df,2p)\C5H7O4(2)\ZIP06\20-Feb-2013\0\\#p ROMP2(FC)/ 6-311+G(3df,2p) scf=tight\\R2Ket3Ribo_008\\0,2\O,0,-2.773843,-0.556068,0.061717\C,0,- 1.948049,0.521375,0.453062\C,0,-0.699266,0.578615,-0.440336\O,0,0.124381,1.711267,-0.115448\C,0,0.1 85873,-0.635971,-0.22193\O,0,-0.13748,-1.842216,-0.214019\C,0,1.487206,-0.142577,0.040136\O,0,2.538 578,-0.918708,0.282807\C,0,1.503954,1.344188,0.018563\H,0,-1.631426,0.452874,1.50672\H,0,-2.528742, 1.44128,0.336739\H,0,-1.025161,0.634971,-1.489921\H,0,2.096207,1.741753,-0.822889\H,0,1.910013,1.77 9728,0.94248\H,0,-2.216823,-1.35663,0.067771\H,0,2.204541,-1.841944,0.241674\\Version=AM64L-G03 RevD.01\State=2-A\HF=-492.982506\MP2=-494.724083\RMSD=3.076e-09\Thermal=0.\PG=C01 [X(C5H 7O4)]\\@

009

1\1\GINC-NODE19\SP\ROMP2-FC\6-311+G(3df,2p)\C5H7O4(2)\ZIP06\20-Feb-2013\0\\#p ROMP2(FC)/ 6-311+G(3df,2p) scf=tight\\R2Ket3Ribo_009\\0,2\O,0,-3.059874,-0.049793,-0.193862\C,0,-1.847411, 0.634668,-0.441748\C,0,-0.744729,0.164229,0.507264\O,0,-0.570767,-1.263093,0.335873\C,0,0.617201, 0.774305,0.220366\O,0,0.960918,1.969872,0.213068\C,0,1.48459,-0.306492,-0.078862\O,0,2.766884,-0.14 2041,-0.38878\C,0,0.78145,-1.611502,0.002684\H,0,-2.033417,1.700997,-0.281484\H,0,-1.507893,0.49870 2,-1.481751\H,0,-1.058707,0.352881,1.543086\H,0,1.202357,-2.269448,0.779444\H,0,0.804304,-2.167405, -0.947064\H,0,-2.846868,-0.996678,-0.243541\H,0,2.916325,0.830138,-0.357316\\Version=AM64L-G03 RevD.01\State=2-A\HF=-492.9817433\MP2=-494.7235357\RMSD=3.751e-09\Thermal=0.\PG=C01 [X(C5 H7O4)]\\@

004

1\1\GINC-NODE17\SP\ROMP2-FC\6-311+G(3df,2p)\C5H7O4(2)\ZIP06\20-Feb-2013\0\\#p ROMP2(FC)/ 6-311+G(3df,2p) scf=tight\\R2Ket3Ribo_004\\0,2\O,0,-1.920045,-0.408787,1.268875\C,0,-2.114338,-0.058097,-0.092396\C,0,-0.810023,0.317371,-0.805275\O,0,-0.329308,1.603334,-0.374584\C,0,0.311369,-0.659933,-0.480351\O,0,0.328847,-1.900045,-0.602861\C,0,1.35641,0.103779,0.092341\O,0,2.491594,-0.431171,0.530865\C,0,0.977468,1.533934,0.206288\H,0,-2.780039,0.809831,-0.102213\H,0,-2.596764,-0.870096,-0.656937\H,0,-0.995719,0.357381,-1.889792\H,0,1.664988,2.201678,-0.336088\H,0,0.945381, 1.870191,1.254403\H,0,-1.544631,-1.303592,1.281012\H,0,2.412766,-1.394362,0.347613\\Version=AM64 L-G03RevD.01\State=2-A\HF=-492.9814161\MP2=-494.723105\RMSD=6.966e-09\Thermal=0.\PG=C01 [X(C5H7O4)]\\@

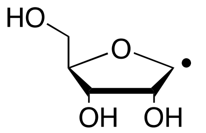

026

1\1\GINC-NODE11\SP\ROMP2-FC\6-311+G(3df,2p)\C5H9O4(2)\ZIP06\21-Feb-2013\0\\#p ROMP2(FC)/ 6-311+G(3df,2p) scf=tight\\Rad1Ribo_026\\0,2\O,0,2.627135,0.043901,0.720953\C,0,2.080832,0.407516,-0.535291\C,0,0.604263,0.05615,-0.638897\O,0,0.474251,-1.375051,-0.411636\C,0,-0.314307,0.69558, 0.423609\O,0,-0.797321,1.962691,0.05983\C,0,-1.436421,-0.366469,0.583718\O,0,-2.557448,0.003197,-0.267031\C,0,-0.745592,-1.611112,0.162927\H,0,-2.480182,-0.526243,-1.078504\H,0,2.188991,1.49259,-0.629475\H,0,2.631844,-0.064098,-1.363921\H,0,0.225806,0.290088,-1.644274\H,0,0.249011,0.787739, 1.358257\H,0,-1.657108,1.78143,-0.370066\H,0,-1.84731,-0.397205,1.597112\H,0,-0.894853,-2.620702, 0.523609\H,0,2.478214,-0.911497,0.813932\\Version=AM64L-G03RevD.01\State=2-A\HF=-494.1319144\ MP2=-495.9034825\RMSD=6.797e-09\Thermal=0.\PG=C01 [X(C5H9O4)]\\@

006

1\1\GINC-NODE11\SP\ROMP2-FC\6-311+G(3df,2p)\C5H9O4(2)\ZIP06\20-Feb-2013\0\\#p ROMP2(FC)/ 6-311+G(3df,2p) scf=tight\\Rad1Ribo_006\\0,2\O,0,3.072966,-0.049699,-0.449917\C,0,2.021478, 0.577032,0.263506\C,0,0.653314,0.120187,-0.206194\O,0,0.55295,-1.310097,0.040769\C,0,-0.558357, 0.720023,0.536852\O,0,-1.000753,1.943122,0.011944\C,0,-1.615024,-0.416476,0.423817\O,0,-2.476366,-0.13909,-0.713556\C,0,-0.760823,-1.619151,0.258749\H,0,-2.14012,-0.66929,-1.45579\H,0,2.105597,0.401 5,1.348861\H,0,2.113469,1.653353,0.087171 \H,0,0.554316,0.29015,-1.286862\H,0,-0.297343,0.870521, 1.592609\H,0,-1.69798,1.695137,-0.628188 \H,0,-2.286874,-0.451971,1.28649\H,0,-0.944479,-2.627 081,0.607594\H,0,2.959508,-1.005903,-0.326183 \\Version=AM64L-G03RevD.01\State=2-A\HF=-494.1317701\MP2=-495.9024489\RMSD=8.115e-09\Thermal=0.\PG=C01 [X(C5H9O4)]\\@

007

1\1\GINC-NODE21\SP\ROMP2-FC\6-311+G(3df,2p)\C5H9O4(2)\ZIP06\21-Feb-2013\0\\#p ROMP2(FC)/ 6-311+G(3df,2p) scf=tight\\Rad1Ribo_007\\0,2\O,0,-2.759066,-0.506678,-0.288391\C,0,-2.044959, 0.669185,0.055336\C,0,-0.573769,0.49187,-0.289115\O,0,0.172093,1.714035,-0.097142\C,0,0.170976,-0.5

20659,0.604245\O,0,-0.082195,-1.866972,0.276914\C,0,1.6431,-0.083368,0.418025\O,0,2.203093,-0.847318,-0.684248\C,0,1.476373,1.369387,0.140698\H,0,2.218692,-0.257287,-1.456229\H,0,-2.144755,0.92033,1.124756\H,0,-2.480223,1.491069,-0.52197\H,0,-0.488981,0.18415,-1.341446\H,0,-0.132053,-0.367652,1.647595\H,0,0.56905,-2.079446,-0.421624\H,0,2.27213,-0.309547,1.284122\H,0,2.116093,2.186462, 0.448246\H,0,-2.231668,-1.2711,0.004348\\Version=AM64L-G03RevD.01\State=2-A\HF=-494.1302736\MP2=-495.901822\RMSD=2.663e-09\Thermal=0.\PG=C01 [X(C5H9O4)]\\@

009

1\1\GINC-NODE17\SP\ROMP2-FC\6-311+G(3df,2p)\C5H9O4(2)\ZIP06\21-Feb-2013\0\\#p ROMP2(FC)/6-311+G(3df,2p) scf=tight\\Rad1Ribo_009\\0,2\O,0,-2.199203,-0.43696,-1.089772\C,0,-2.153405,0.52294,-0.043682\C,0,-0.786445,0.560763,0.630553\O,0,-0.565391,-0.756049,1.219412\C,0,0.385382,0.815733,-0.327774\O,0,1.308104,1.686613,0.299773\C,0,1.029512,-0.592404,-0.540795\O,0,2.462101,-0.519851,-0.584794\C,0,0.504749,-1.357412,0.624407\H,0,2.756192,-0.597099,0.339659\H,0,-2.361923,1.495575,-0.501343\H,0,-2.928841,0.330504,0.712558\H,0,-0.774796,1.294289,1.442938\H,0,0.033267,1.235418,-1.279802\H,0,2.163401,1.493857,-0.127819\H,0,0.732753,-1.004801,-1.513558\H,0,0.567418,-2.428632, 0.782617\H,0,-2.111119,-1.306856,-0.668442\\Version=AM64L-G03RevD.01\State=2-A\HF=-494.1305392\MP2=-495.9009916\RMSD=3.086e-09\Thermal=0.\PG=C01 [X(C5H9O4)]\\@

048

1\1\GINC-NODE21\SP\ROMP2-FC\6-311+G(3df,2p)\C5H9O4(2)\ZIP06\21-Feb-2013\0\\#p ROMP2(FC)/6-311+G(3df,2p) scf=tight\\Rad1Ribo_048\\0,2\O,0,3.083411,-0.043263,-0.415469\C,0,2.022578, 0.569348,0.295319\C,0,0.661244,0.139921,-0.222724\O,0,0.556837,-1.300499,-0.06344\C,0,-0.555989, 0.725688,0.524307\O,0,-1.029842,1.930941,-0.021654\C,0,-1.592055,-0.432365,0.442732\O,0,-2.396418,-0.237701,-0.759457\C,0,-0.710304,-1.612528,0.345526\H,0,-3.280086,0.047872,-0.477892\H,0,2.079571, 0.3523,1.37536\H,0,2.130587,1.650583,0.161633\H,0,0.582028,0.360142,-1.294917\H,0,-0.296373,0.91947,1.571731\H,0,-1.569215,1.649335,-0.786094\H,0,-2.261267,-0.460688,1.310666\H,0,-0.989696,-2.6539,0.257188\H,0,2.939704,-1.001315,-0.348457\\Version=AM64L-G03RevD.01\State=2-A\HF=-494.1306266\MP2=-495.8998893\RMSD=2.720e-09\Thermal=0.\PG=C01 [X(C5H9O4)]\\@

010

1\1\GINC-NODE19\SP\ROMP2-FC\6-311+G(3df,2p)\C5H9O4(2)\ZIP06\21-Feb-2013\0\\#p ROMP2(FC)/6-311+G(3df,2p) scf=tight\\Rad1Ribo_010\\0,2\O,0,3.123701,-0.089736,0.01487\C,0,1.964191, 0.455531,0.611256\C,0,0.767114,0.404231,-0.33382\O,0,0.597681,-0.993833,-0.722714\C,0,-0.557313, 0.852028,0.291809\O,0,-1.290421,1.6087,-0.64875 \C,0,-1.305694,-0.48772,0.616674\O,0,-2.706038,-0.390616,0.312249\C,0,-0.574039,-1.475428,-0.221067\H,0,-2.791654,-0.657795,-0.619793\H,0,1.708808,-0.063092,1.552209\H,0,2.191628,1.49827,0.857085\H,0,0.979381,0.965019,-1.247514\H,0,-0.383015, 1.435193,1.209464\H,0,-2.225069,1.483639,-0.397243\H,0,-1.272195,-0.703439,1.693209\H,0,-0.644292,-2.555855,-0.171262\H,0,2.871466,-0.969934,-0.310496\\Version=AM64L-G03RevD.01\State=2-A\HF=-494.1290844\MP2=-495.8990543\RMSD=2.045e-09\Thermal=0.\PG=C01 [X(C5H9O4)]\\@

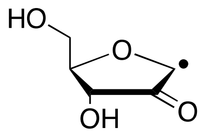

001

1\1\GINC-NODE19\SP\ROMP2-FC\6-311+G(3df,2p)\C5H7O4(2)\ZIP06\21-Feb-2013\0\\#p ROMP2(FC)/6-311+G(3df,2p) scf=tight\\R1Ket2Ribo_001\\0,2\O,0,2.350876,-0.025079,1.095332\C,0,2.123398,0.4163,-0.231\C,0,0.694202,0.167806,-0.686088\O,0,0.486311,-1.302769,-0.656846\C,0,-0.421271,0.746138,0.186541\O,0,-0.979954,1.912466,-0.380058\C,0,-1.441374,-0.403102,0.241125\O,0,-2.617842,-0.258432,0.587739\C,0,-0.754378,-1.562315,-0.21404\H,0,2.819176,-0.056529,-0.940682\H,0,2.310172,1.494775,-0.242569\H,0,0.548474,0.485975,-1.724408\H,0,-0.04153,0.934724,1.203554\H,0,-1.907811,1.924424,-0.080001\H,0,-1.082831,-2.592789,-0.259465 \H,0,2.235771,-0.989028,1.094991\\Version=AM64L-G03RevD.01\State=2-A\HF=-492.9783266\MP2=-494.7214832\RMSD=7.814e-09\Thermal=0.\PG=C01 [X(C5H7O4)]\\@

003

1\1\GINC-NODE11\SP\ROMP2-FC\6-311+G(3df,2p)\C5H7O4(2)\ZIP06\21-Feb-2013\0\\#p ROMP2(FC)/6-311+G(3df,2p) scf=tight\\R1Ket2Ribo_003\\0,2\O,0,3.099582,-0.05262,-0.08484\C,0,1.949505,0.532591,0.49238\C,0,0.686944,0.221239,-0.292114\O,0,0.538527,-1.259101,-0.292525\C,0,-0.626847,0.761271,0.274454\O,0,-1.111205,1.862182,-0.462021\C,0,-1.572703,-0.450305,0.16646\O,0,-2.801034,-0.36247,0.263479\C,0,-0.748892,-1.575952,-0.102442\H,0,2.106451,1.616178,0.484505\H,0,1.810324,0.220105,1.540628\H,0,0.797296,0.514255,-1.340277 \H,0,-0.511529,1.023443,1.341527\H,0,-2.081897,1.82417,-0.373274\H,0,-1.012267,-2.622477,-0.186211\H,0,2.956622,-1.012666,-0.092058\\Version=AM64L-G03RevD.01\State=2-A\HF=-492.9773747\MP2=-494.7203944\RMSD=2.416e-09\Thermal=0.\PG=C01 [X(C5H7O4)]\\@

002

1\1\GINC-NODE9\SP\ROMP2-FC\6-311+G(3df,2p)\C5H7O4(2)\ZIP06\21-Feb-2013\0\\#p ROMP2(FC)/6-311+G(3df,2p) scf=tight\\R1Ket2Ribo_002\\0,2\O,0,2.232076,-0.037358,1.121033\C,0,2.1328,0.307671,-0.250778\C,0,0.701588,0.099203,-0.700046\O,0,0.419515,-1.351836,-0.672715\C,0,-0.392422,0.751109,0.152064\O,0,-0.934726,1.894507,-0.481271\C,0,-1.439039,-0.368185,0.278013\O,0,-2.59855,-0.176166,0.662284\C,0,-0.806547,-1.55535,-0.177242\H,0,2.795245,-0.311454,-0.874724\H,0,2.384472,1.36518,-0.430403\H,0,0.587762,0.419339,-1.741435\H,0,-0.004541,0.995854,1.151845\H,0,-1.856836,1.944115,-0.167691\H,0,-1.17416,-2.573261,-0.192981\H,0,3.143259,0.120377,1.40866\\Version=AM64L-G03RevD.01\State=2-A\HF=-492.9763132\MP2=-494.7194472\RMSD=6.129e-09\Thermal=0.\PG=C01 [X(C5H7O4)]\\@

008

1\1\GINC-NODE10\SP\ROMP2-FC\6-311+G(3df,2p)\C5H7O4(2)\ZIP06\21-Feb-2013\0\\#p ROMP2(FC)/6-311+G(3df,2p) scf=tight\\R1Ket2Ribo_008\\0,2\O,0,2.841966,0.551366,0.011692\C,0,2.033924,-0.575402,0.299067\C,0,0.664448,-0.418281,-0.346097\O,0,-0.0847,-1.683931,-0.212702\C,0,-0.252641,

0.636776,0.277881\O,0,-0.177795,1.884774,-0.36962\C,0,-1.638774,-0.017583,0.146013\O,0,-2.696908,
0.616508,0.194817\C,0,-1.387284,-1.402964,-0.055011\H,0,1.915219,-0.742125,1.38224\H,0,2.549625,-
1.444381,-0.119483\H,0,0.772849,-0.219078,-1.417696\H,0,-0.027501,0.741787,1.355917\H,0,-1.075662,
2.263739,-0.309789\H,0,-2.074914,-2.237736,-0.094378\H,0,2.361834,1.352775,0.278567\\Version=AM
64L-G03RevD.01\State=2-A\HF=-492.9753733\MP2=-494.7182927\RMSD=5.087e-09\Thermal=0.\PG=
C01 [X(C5H7O4)]\\@

005

1\1\GINC-NODE26\SP\ROMP2-FC\6-311+G(3df,2p)\C5H7O4(2)\ZIP06\21-Feb-2013\0\\#p ROMP2(FC)/
6-311+G(3df,2p) scf=tight\\R1Ket2Ribo_005\\0,2\O,0,3.054053,-0.10929,-0.217632\C,0,1.957536,
0.440663,0.489674\C,0,0.688684,0.11654,-0.265903\O,0,0.458463,-1.344474,-0.213974\C,0,-0.591719,
0.744031,0.290588\O,0,-0.985703,1.89379,-0.422466\C,0,-1.613843,-0.39718,0.143471\O,0,-2.836583,-
0.220541,0.184942\C,0,-0.852591,-1.575348,-0.077385\H,0,2.021506,1.538903,0.562847\H,0,1.878863,
0.037415,1.512071\H,0,0.80128,0.374253,-1.32365\H,0,-0.474104,0.972202,1.365728\H,0,-1.961056,
1.893053,-0.386747\H,0,-1.176144,-2.605548,-0.152929\H,0,3.859416,0.061604,0.293044\\Version=
AM64L-G03RevD.01\State=2-A\HF=-492.9750719\MP2=-494.7181693\RMSD=6.088e-09\Thermal=
0.\PG=C01 [X(C5H7O4)]\\@

007

1\1\GINC-NODE12\SP\ROMP2-FC\6-311+G(3df,2p)\C5H7O4(2)\ZIP06\21-Feb-2013\0\\#p ROMP2(FC)/
6-311+G(3df,2p) scf=tight\\R1Ket2Ribo_007\\0,2\O,0,2.745435,-0.624486,-0.454997\C,0,1.998368,
0.570692,-0.323027\C,0,0.701941,0.333776,0.449624\O,0,-0.034427,1.614897,0.464662\C,0,-0.2455,-0.7
0749,-0.149022\O,0,-0.391189,-1.818287,0.719054\C,0,-1.574866,0.059799,-0.279633\O,0,-2.649113,-0.47
4963,-0.578168\C,0,-1.297951,1.407235,0.072458\H,0,1.758755,0.894393,-1.34091\H,0,2.573485,1.37629
2,0.157117\H,0,0.902971,0.083249,1.497342\H,0,0.103696,-1.031749,-1.141102\H,0,-1.24446,-2.224495,
0.482126\H,0,-1.956779,2.265945,0.088193\H,0,3.004739,-0.924999,0.430426\\Version=AM64L-G03Rev
D.01\State=2-A\HF=-492.9747464\MP2=-494.7177302\RMSD=2.722e-09\Thermal=0.\PG=C01 [X(C5H7
O4)]\\@

010

1\1\GINC-NODE17\SP\ROMP2-FC\6-311+G(3df,2p)\C5H7O4(2)\ZIP06\21-Feb-2013\0\\#p ROMP2(FC)/
6-311+G(3df,2p) scf=tight\\R1Ket2Ribo_010\\0,2\O,0,3.122152,-0.143927,-0.04748\C,0,1.959918,
0.444357,0.503183\C,0,0.688229,0.127779,-0.267271\O,0,0.464908,-1.332822,-0.241341\C,0,-0.597131,
0.745415,0.290691\O,0,-0.998296,1.890292,-0.428083\C,0,-1.611257,-0.403327,0.145246\O,0,-2.834915,-
0.237313,0.197921\C,0,-0.843563,-1.573895,-0.093995\H,0,2.040085,1.541247,0.575619\H,0,1.872169,
0.049871,1.520826\H,0,0.786954,0.403823,-1.324163\H,0,-0.479503,0.97911,1.364217\H,0,-1.972934,
1.893727,-0.378549\H,0,-1.160282,-2.605689,-0.175803\H,0,3.305555,0.286103,-0.8974\\Version=AM64L
-G03RevD.01\State=2-A\HF=-492.9745623\MP2=-494.7177918\RMSD=6.338e-09\Thermal=0.\PG=C01
[X(C5H7O4)]\\@

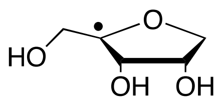

011

1\1\GINC-NODE40\SP\ROMP2-FC\6-311+G(3df,2p)\C5H9O4(2)\ZIP06\21-Feb-2013\0\\#p ROMP2(FC)/6-311+G(3df,2p) scf=tight\\Rad4Ribo_011\\0,2\O,0,-2.518489,-0.60391,-0.628323\C,0,-2.177401,0.22266,0.528182\C,0,-0.719893,0.491171,0.591126\O,0,-0.271114,1.5215,-0.206665\C,0,0.310049,-0.582673,0.708258\O,0,0.185542,-1.590971,-0.322622\C,0,1.615608,0.187735,0.41076\O,0,2.629878,-0.614081,-0.138611\C,0,1.115024,1.295927,-0.543251\H,0,2.158459,-1.277567,-0.677738\H,0,-2.50012,-0.36595,1.390499\H,0,-2.752849,1.156148,0.504746\H,0,0.317779,-1.075873,1.688164\H,0,-0.752396,-1.60197,-0.596606\H,0,2.006379,0.631129,1.332683\H,0,1.174638,0.949968,-1.582113\H,0,1.648509,2.244639,-0.448146\H,0,-2.367262,-0.049752,-1.412171\\Version=AM64L-G03RevD.01\State=2-A\HF=-494.135635\MP2=-495.905742\RMSD=8.460e-09\Thermal=0.\PG=C01 [X(C5H9O4)]\\@

003

1\1\GINC-NODE41\SP\ROMP2-FC\6-311+G(3df,2p)\C5H9O4(2)\ZIP06\21-Feb-2013\0\\#p ROMP2(FC)/6-311+G(3df,2p) scf=tight\\Rad4Ribo_003\\0,2\O,0,-2.160234,0.270603,0.904378\C,0,-1.959304,-0.63672,-0.22505\C,0,-0.526557,-0.70393,-0.606712\O,0,0.223247,-1.600146,0.119304\C,0,0.277826,0.52719,-0.959541\O,0,-0.346455,1.762769,-0.609321\C,0,1.592404,0.304584,-0.141728\O,0,1.461203,0.92348,1.122399\C,0,1.610302,-1.206506,0.044232 \H,0,0.992457,1.759743,0.933102\H,0,-2.346013,-1.632851,0.019566\H,0,-2.564673,-0.209486,-1.028026\H,0,0.502192,0.588198,-2.033092\H,0,-1.051582,1.54859,0.039124\H,0,2.484026,0.66602,-0.674605 \H,0,2.094747,-1.514404,0.972541\H,0,2.082912,-1.712254,-0.807681\H,0,-1.584184,-0.054915,1.617797\\Version=AM64L-G03RevD.01\State=2-A\HF=-494.1338523\MP2=-495.9063667\RMSD=4.616e-09\Thermal=0.\PG=C01 [X(C5H9O4)]\\@

041

1\1\GINC-NODE42\SP\ROMP2-FC\6-311+G(3df,2p)\C5H9O4(2)\ZIP06\21-Feb-2013\0\\#p ROMP2(FC)/6-311+G(3df,2p) scf=tight\\Rad4Ribo_041\\0,2\O,0,-2.482522,-0.59703,-0.608751\C,0,-2.17564,0.342008,0.464594\C,0,-0.709839,0.536858,0.509333\O,0,-0.196497,1.561255,-0.244887\C,0,0.278191,-0.566255,0.68686\O,0,0.162081,-1.600354,-0.317593 \C,0,1.618165,0.159794,0.422662\O,0,2.62005,-0.681894,-0.08779\C,0,1.18245,1.27202,-0.556724\H,0,2.138334,-1.335791,-0.630381\H,0,-2.680932,1.299623,0.286214\H,0,-2.527985,-0.071895,1.419966\H,0,0.235496,-1.034519,1.678616\H,0,-0.751951,-1.55857,-0.659648\H,0,1.995074,0.600534,1.351824 \H,0,1.240146,0.905791,-1.588573\H,0,1.753693,2.198977,-0.468275\H,0,-3.386727,-0.926517,-0.477941\\Version=AM64L-G03RevD.01\State=2-A\HF=-494.1339936\MP2=-495.903972\RMSD=2.419e-09\Thermal=0.\PG=C01 [X(C5H9O4)]\\@

034

1\1\GINC-NODE42\SP\ROMP2-FC\6-311+G(3df,2p)\C5H9O4(2)\ZIP06\21-Feb-2013\0\\#p ROMP2(FC)/6-311+G(3df,2p) scf=tight\\Rad4Ribo_034\\0,2\O,0,-1.807423,-0.152174,1.05821\C,0,-1.851166,-0.520147,-0.35176\C,0,-0.449429,-0.575365,-0.834096\O,0,0.2911,-1.610658,-0.302758\C,0,0.411905,0.67

3206,-0.938817\O,0,-0.27004,1.89301,-0.762091\C,0,1.453013,0.380609,0.179019\O,0,0.917884,0.771738, 1.435353\C,0,1.616555,-1.130403,0.031818 \H,0,0.058094,0.314187,1.558711\H,0,-2.402872,0.234741,-0.922481\H,0,-2.34149,-1.496072,-0.458696\H,0,0.91569,0.725451,-1.914215\H,0,-0.301084,2.024235, 0.204316\H,0,2.390305,0.928638,0.057034 \H,0,1.925964,-1.624767,0.95572\H,0,2.317644,-1.386454,-0.773832\H,0,-2.699684,0.097311,1.346752\\Version=AM64L-G03RevD.01\State=2-A\HF=-494.1326 508\MP2=-495.9031911\RMSD=2.117e-09\Thermal=0.\PG=C01 [X(C5H9O4)]\\@

001

1\1\GINC-NODE38\SP\ROMP2-FC\6-311+G(3df,2p)\C5H9O4(2)\ZIP06\21-Feb-2013\0\\#p ROMP2(FC)/ 6-311+G(3df,2p) scf=tight\\Rad4Ribo_001\\0,2\O,0,-2.311734,-0.348516,-0.789863\C,0,-2.005189, 0.659188,0.223992\C,0,-0.546367,0.680217,0.456433 \O,0,0.192181,1.580188,-0.25781\C,0,0.261622,-0.50936,0.891197\O,0,-0.258531,-1.768959,0.44193 \C,0,1.659807,-0.223536,0.239513\O,0,1.786878,-0.947949,-0.960603\C,0,1.589636,1.274407,-0.058761\H,0,1.190177,-1.715384,-0.84434\H,0,-2.530015, 0.399127,1.154474\H,0,-2.347628,1.645598,-0.111045\H,0,0.353469,-0.584114,1.983743\H,0,-1.002554,-1.563979,-0.163318\H,0,2.485431,-0.454257,0.929452\H,0,2.122522,1.541797,-0.972464\H,0,1.963413, 1.873338,0.78204 \H,0,-3.262224,-0.54573,-0.742022\\Version=AM64L-G03RevD.01\State=2-A\HF=-494.1301118\MP2=-495.9026737\RMSD=2.689e-09\Thermal=0.\PG=C01 [X(C5H9O4)]\\@

040

1\1\GINC-NODE38\SP\ROMP2-FC\6-311+G(3df,2p)\C5H9O4(2)\ZIP06\21-Feb-2013\0\\#p ROMP2(FC)/ 6-311+G(3df,2p) scf=tight\\Rad4Ribo_040\\0,2\O,0,-1.926189,-0.013787,1.018279\C,0,-1.798125,-0.824514,-0.183932\C,0,-0.405336,-0.767572,-0.710121\O,0,0.472063,-1.618186,-0.060627\C,0,0.283353, 0.556401,-1.002226\O,0,-0.546391,1.703398,-0.833907\C,0,1.432323,0.515754,0.043523\O,0,0.934577, 1.009953,1.282648\C,0,1.754976,-0.973246,0.076155 \H,0,0.138641,0.482081,1.513676\H,0,-2.044703,-1.846723,0.111976\H,0,-2.521698,-0.488731,-0.934856 \H,0,0.691026,0.586813,-2.022464\H,0,-0.241483,2.097562,0.007409 \H,0,2.290138,1.141026,-0.215609\H,0,2.19726,-1.297925,1.020761\ H,0,2.412131,-1.262281,-0.755483\H,0,-1.996938,0.896223,0.683057\\Version=AM64L-G03RevD.01\ State=2-A\HF=-494.1298487\MP2=-495.9024036\RMSD=5.562e-09\Thermal=0.\PG=C01 [X(C5H9O4)] \\@

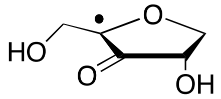

004

1\1\GINC-NODE8\SP\ROMP2-FC\6-311+G(3df,2p)\C5H7O4(2)\ZIP06\21-Feb-2013\0\\#p ROMP2(FC)/6-311+G(3df,2p) scf=tight\\R4Ket3Ribo_004\\0,2\O,0,2.793429,-0.538367,-0.149114\C,0,2.109201, 0.606719,0.307135\C,0,0.642717,0.536427,0.017271 \O,0,-0.085566,1.659327,0.014323\C,0,-0.133567,-0.631363,-0.174686\O,0,0.233312,-1.821741,-0.101111 \C,0,-1.583832,-0.187613,-0.404493\O,0,-2.464328,-0.819754,0.507216 \C,0,-1.51101,1.318621,-0.136608\H,0,2.542266,1.483773,-0.18996\ H,0,2.243706,0.766875,1.394467\H,0,-2.00996,1.569975,0.802287\H,0,-1.888516,1.945824,-0.944956\ H,0,2.214921,-1.309276,0.009304 \H,0,-1.880376,-0.399893,-1.443781\H,0,-2.177866,-1.749739,

0.550416\\Version=AM64L-G03RevD.01\State=2-A\HF=-492.9785734\MP2=-494.7228944\RMSD=5.796e-09\Thermal=0.\PG=C01 [X(C5H7O4)]\\@

001

1\1\GINC-NODE3\SP\ROMP2-FC\6-311+G(3df,2p)\C5H7O4(2)\ZIP06\21-Feb-2013\0\\#p ROMP2(FC)/6-311+G(3df,2p) scf=tight\\R4Ket3Ribo_001\\0,2\O,0,-2.711001,-0.572062,-0.418336\C,0,-2.127546,0.641213,0.003241\C,0,-0.635176,0.549343,0.078893\O,0,0.103722,1.658873,-0.029816\C,0,0.135979,-0.615756,0.306978\O,0,-0.2646,-1.793526,0.410266\C,0,1.610076,-0.193198,0.344989\O,0,2.364059,-0.858015,-0.655512\C,0,1.532045,1.306953,0.047007\H,0,-2.511833,0.961781,0.990928\H,0,-2.409892,1.425704,-0.70963\H,0,1.977626,1.536837,-0.923238\H,0,1.961037,1.945543,0.82046\H,0,-2.177004,-1.301328,-0.047026\H,0,2.030549,-0.387987,1.343618\H,0,2.099798,-1.794038,-0.614577\\Version=AM64L-G03RevD.01\State=2-A\HF=-492.9779287\MP2=-494.722518\RMSD=7.027e-09\Thermal=0.\PG=C01 [X(C5H7O4)]\\@

002

1\1\GINC-NODE6\SP\ROMP2-FC\6-311+G(3df,2p)\C5H7O4(2)\ZIP06\21-Feb-2013\0\\#p ROMP2(FC)/6-311+G(3df,2p) scf=tight\\R4Ket3Ribo_002\\0,2\O,0,-2.908874,-0.080281,0.742014\C,0,-2.133638,0.477485,-0.312184\C,0,-0.712258,0.027576,-0.282726\O,0,-0.483797,-1.299804,-0.293033\C,0,0.488616,0.786242,-0.224269\O,0,0.644083,2.006424,-0.088662\C,0,1.653617,-0.215922,-0.306366\O,0,2.636028,0.055148,0.669491\C,0,0.946973,-1.549288,-0.057643\H,0,-2.146713,1.562638,-0.183612\H,0,-2.576096,0.246423,-1.296322\H,0,1.067951,-1.854981,0.985488\H,0,1.228865,-2.362825,-0.726381\H,0,-2.871848,-1.045113,0.644707\H,0,2.087835,-0.181195,-1.319573\H,0,2.650622,1.026607,0.756331\\Version=AM64L-G03RevD.01\State=2-A\HF=-492.9786466\MP2=-494.7208498\RMSD=6.724e-09\Thermal=0.\PG=C01 [X(C5H7O4)]\\@

007

1\1\GINC-NODE3\SP\ROMP2-FC\6-311+G(3df,2p)\C5H7O4(2)\ZIP06\21-Feb-2013\0\\#p ROMP2(FC)/6-311+G(3df,2p) scf=tight\\R4Ket3Ribo_007\\0,2\O,0,3.048117,-0.081366,0.427144\C,0,2.086087,0.483881,-0.457185\C,0,0.697101,0.026679,-0.163185\O,0,0.467959,-1.299014,-0.176703\C,0,-0.467999,0.782553,0.139627\O,0,-0.631934,2.009399,0.124572\C,0,-1.591052,-0.223944,0.443997\O,0,-2.75596,0.063937,-0.303849\C,0,-0.966966,-1.555255,0.022903\H,0,2.334967,0.262605,-1.508693\H,0,2.121706,1.567629,-0.32293\H,0,-1.380123,-1.888862,-0.932766\H,0,-1.035446,-2.353562,0.762692\H,0,3.018454,-1.043655,0.305307\H,0,-1.806412,-0.207837,1.52471\H,0,-2.821625,1.036551,-0.314579\\Version=AM64L-G03RevD.01\State=2-A\HF=-492.9785919\MP2=-494.7207735\RMSD=8.672e-09\Thermal=0.\PG=C01 [X(C5H7O4)]\\@

006

1\1\GINC-NODE6\SP\ROMP2-FC\6-311+G(3df,2p)\C5H7O4(2)\ZIP06\21-Feb-2013\0\\#p ROMP2(FC)/6-311+G(3df,2p) scf=tight\\R4Ket3Ribo_006\\0,2\O,0,2.953613,-0.117341,0.527764\C,0,2.089505,0.349705,-0.507877\C,0,0.696299,-0.060199,-0.191814\O,0,0.392988,-1.366625,-0.188296\C,0,-0.421588,0.765516,

0.115384\O,0,-0.520825,1.998363,0.077114\C,0,-1.590072,-0.172883,0.457918\O,0,-2.760624,0.182289,-0.250531\C,0,-1.051602,-1.53606,0.021247\H,0,2.378443,-0.07242,-1.484252\H,0,2.090113,1.445871,-0.588659\H,0,-1.493573,-1.83487,-0.933418\H,0,-1.161718,-2.335979,0.753988\H,0,3.857672,0.134533,0.282846\H,0,-1.766922,-0.144148,1.545485\H,0,-2.760474,1.157055,-0.273543\\Version=AM64L-G03 RevD.01\State=2-A\HF=-492.9767303\MP2=-494.7187897\RMSD=5.552e-09\Thermal=0.\PG=C01 [X(C5H7O4)]\\@

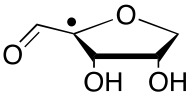

001

1\1\GINC-NODE27\SP\ROMP2-FC\6-311+G(3df,2p)\C5H7O4(2)\ZIP06\21-Feb-2013\0\\#p ROMP2(FC)/ 6-311+G(3df,2p) scf=tight\\R4Ket5Ribo_001\\0,2\O,0,2.7,0.180371,-0.141379\C,0,1.909133,-0.783891,-0.285916\C,0,0.555213,-0.663533,0.095534 \O,0,-0.34375,-1.633033,-0.110546\C,0,-0.063452, 0.56149,0.719072\O,0,0.458307,1.781945,0.241681\C,0,-1.539113,0.391186,0.287493\O,0,-1.716067, 0.913915,-1.008465\C,0,-1.670961,-1.131598,0.242568\H,0,2.242324,-1.740698,-0.720815\H,0,-2.362861,-1.471751,-0.528598\H,0,-1.943638,-1.560099,1.212984 \H,0,-2.242417,0.837098,1.005276\H,0,-1.058425,1.632551,-1.08687\H,0,0.015726,0.500205,1.818781\H,0,1.416456,1.61519,0.096406\\Version= AM64L-G03RevD.01\State=2-A\HF=-492.9781631\MP2=-494.7268751\RMSD=4.815e-09\Thermal= 0.\PG=C01 [X(C5H7O4)]\\@

007

1\1\GINC-NODE22\SP\ROMP2-FC\6-311+G(3df,2p)\C5H7O4(2)\ZIP06\21-Feb-2013\0\\#p ROMP2(FC)/ 6-311+G(3df,2p) scf=tight\\R4Ket5Ribo_007\\0,2\O,0,-2.813134,-0.70021,0.208822\C,0,-2.244193, 0.393247,0.024554\C,0,-0.831802,0.493183,0.095108 \O,0,-0.178377,1.626987,-0.2236\C,0,0.092375,-0.647525,0.397168\O,0,0.221119,-1.531026,-0.724242 \C,0,1.452278,0.073082,0.549799\O,0,2.556732,-0.701529,0.167735\C,0,1.243084,1.339834,-0.308978\H,0,-2.807418,1.313881,-0.213985\H,0,1.497069, 1.142799,-1.356027\H,0,1.777105,2.22385,0.042405 \H,0,1.591782,0.362455,1.597129\H,0,2.22563,-1.327203,-0.502865\H,0,-0.208632,-1.205857,1.289551\H,0,-0.636709,-1.974622,-0.831825\\Version= AM64L-G03RevD.01\State=2-A\HF=-492.9771366\MP2=-494.721755\RMSD=1.360e-09\Thermal= 0.\PG=C01 [X(C5H7O4)]\\@

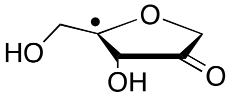

008

1\1\GINC-NODE17\SP\ROMP2-FC\6-311+G(3df,2p)\C5H7O4(2)\ZIP06\21-Feb-2013\0\\#p ROMP2(FC)/ 6-311+G(3df,2p) scf=tight\\R4Ket2Ribo_008\\0,2\O,0,2.622475,0.261992,-0.66346\C,0,2.100251,-0.292698,0.550163\C,0,0.619448,-0.439149,0.526238\O,0,0.112617,-1.536167,-0.134931\C,0,-0.337475, 0.69404,0.566377\O,0,0.060022,1.728572,-0.36932\C,0,-1.637336,0.037687,0.075789\O,0,-2.724063, 0.565176,0.01655\C,0,-1.283054,-1.37204,-0.385163\H,0,2.380409,0.325397,1.418019\H,0,2.57524,-

1.271433,0.665073\H,0,-0.464756,1.138855,1.564491\H,0,-0.597465,2.443885,-0.310308\H,0,-1.476126,-1.500165,-1.455967\H,0,-1.857805,-2.124323,0.168341\H,0,2.101096,1.064157,-0.840792\\Version=AM64L-G03RevD.01\State=2-A\HF=-492.9638212\MP2=-494.6972479\RMSD=4.755e-09\Thermal=0.\PG=C01 [X(C5H7O4)]\\@

001

1\1\GINC-NODE20\SP\ROMP2-FC\6-311+G(3df,2p)\C5H7O4(2)\ZIP06\21-Feb-2013\0\\#p ROMP2(FC)/6-311+G(3df,2p) scf=tight\\R4Ket2Ribo_001\\0,2\O,0,2.745789,0.078098,0.822418\C,0,2.123355,0.276563,-0.460567\C,0,0.671856,-0.038526,-0.445864\O,0,0.379933,-1.395389,-0.39154\C,0,-0.385709,0.755235,0.250114\O,0,-0.685379,1.977571,-0.3966\C,0,-1.565735,-0.219966,0.216271\O,0,-2.726808,0.084111,0.363839\C,0,-1.006716,-1.600302,-0.106269\H,0,2.628367,-0.318102,-1.235363\H,0,2.260354,1.334897,-0.69541\H,0,-0.112776,0.936323,1.312118 \H,0,-1.5621,2.248388,-0.07058\H,0,-1.51537,-2.035249,-0.974608\H,0,-1.107458,-2.288813,0.740932\H,0,2.678395,-0.870598,1.015865\\Version=AM64L-G03RevD.01\State=2-A\HF=-492.9669876\MP2=-494.6968455\RMSD=4.174e-09\Thermal=0.\PG=C01 [X(C5H7O4)]\\@

011

1\1\GINC-NODE26\SP\ROMP2-FC\6-311+G(3df,2p)\C5H7O4(2)\ZIP06\21-Feb-2013\0\\#p ROMP2(FC)/6-311+G(3df,2p) scf=tight\\R4Ket2Ribo_011\\0,2\O,0,-2.582352,-0.384521,-0.436888\C,0,-2.036872,0.468921, 0.605421\C,0,-0.561681,0.475076,0.484503\O,0,-0.016168,1.492371,-0.2666\C,0,0.289432,-0.747913,0.470861\O,0,-0.01401,-1.635072,-0.625858 \C,0,1.658051,-0.132446,0.141217\O,0,2.752524,-0.594874,0.348579\C,0,1.365225,1.213914,-0.526159\H,0,-2.325355,0.073495,1.590011\H,0,-2.424684,1.491391,0.509974\H,0,0.301228,-1.299566,1.419508\H,0,-0.968447,-1.521179,-0.801086\H,0,1.522204,1.157867,-1.609155\H,0,1.989839,2.01209,-0.113015 \H,0,-3.499668,-0.602639,-0.205163\\Version=AM64L-G03RevD.01\State=2-A\HF=-492.9613087\MP2=-494.6962923\RMSD=4.171e-09\Thermal=0.\PG=C01 [X(C5H7O4)]\\@

013

1\1\GINC-NODE9\SP\ROMP2-FC\6-311+G(3df,2p)\C5H7O4(2)\ZIP06\21-Feb-2013\0\\#p ROMP2(FC)/6-311+G(3df,2p) scf=tight\\R4Ket2Ribo_013\\0,2\O,0,2.708101,-0.586013,-0.18428\C,0,2.090511,0.689332,-0.141843\C,0,0.601598,0.575203,-0.233441\O,0,-0.142848,1.620474,0.262318\C,0,-0.160777,-0.681473,-0.360624\O,0,0.153662,-1.583698,0.74443\C,0,-1.603393,-0.183375,-0.199507\O,0,-2.611443,-0.831438,-0.365539\C,0,-1.528951,1.268581,0.265995\H,0,2.5117,1.257026,-0.98402\H,0,2.346397,1.256717,0.772218\H,0,-0.009483,-1.212211,-1.309905\H,0,-0.311986,-2.42157,0.572361\H,0,-1.927164,1.382462,1.280118\H,0,-2.08535,1.929043,-0.408704\H,0,2.222182,-1.155654,0.439019\\Version=AM64L-G03RevD.01\State=2-A\HF=-492.9605005\MP2=-494.6949241\RMSD=4.641e-09\Thermal=0.\PG=C01 [X(C5H7O4)]\\@

003

1\1\GINC-NODE12\SP\ROMP2-FC\6-311+G(3df,2p)\C5H7O4(2)\ZIP06\21-Feb-2013\0\\#p ROMP2(FC)/ 6-311+G(3df,2p) scf=tight\\R4Ket2Ribo_003\\0,2\O,0,3.040056,-0.097644,-0.464664\C,0,2.054628, 0.572253,0.307719\C,0,0.670931,0.092583,0.030831\O,0,0.525692,-1.286934,0.127406\C,0,-0.607812, 0.768187,0.406455\O,0,-0.849198,1.975052,-0.282808\C,0,-1.615051,-0.327268,0.040067\O,0,-2.781854,- 0.135931,-0.213015\C,0,-0.85204,-1.644367,-0.020223\H,0,2.116685,1.636011,0.059813\H,0,2.280037, 0.475553,1.39082\H,0,-0.678371,0.936139,1.506434\H,0,-1.817923,2.066613,-0.335609\H,0,-1.019448,- 2.148339,-0.979242\H,0,-1.140139,-2.322239,0.791102\H,0,2.877656,-1.048415,-0.35777\\Version= AM64L-G03RevD.01\State=2-A\HF=-492.9651263\MP2=-494.6940222\RMSD=6.174e-09\Thermal= 0.\PG=C01 [X(C5H7O4)]\\@

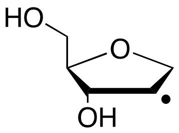

009

1\1\GINC-LX64I13\SP\ROMP2-FC\6-311+G(3df,2p)\C5H9O3(2)\UI271AB\14-Oct-2007\0\\#P ROMP2 (FC)/6-311+G(3df,2p) scf=tight geom=check guess=read\\ prh_009 ROMP2(FC)/6- 311+G(3df,2p)//B3LYP/6-31G(d) sp\\0,2\C,0,-0.0302746705,0.0073553955,-0.0713114161\ C,0,0.1289220994,0.3786610921,1.3657351937 \C,0,1.4834464623,-0.0218341104,1.8427879419\ O,0,2.1726702547,-0.4934957655,0.6754471345 \C,0,1.200920532,-0.9089108184,-0.2934674755\ C,0,1.8505432179,-0.7902430506,-1.6662814186\O,0,2.3245704844,0.5251491941,-1.8972663986\O,0,- 1.2692596845,-0.6044520391,-0.421195041 \H,0,1.4463966355,-0.8200920971,2.6085453\H,0, 2.0694004793,0.8032671787,2.2736778887 \H,0,-1.4437032568,-1.2944171609,0.2402982108\H,0, 2.6624671978,-1.5296154269,-1.7522492635 \H,0,1.1060386509,-1.0092908945,-2.4384385117\H,0, 2.855391373,0.743758898,-1.1121585948\ H,0,-0.5963673607,0.937795904,1.9454404451\H,0, 0.0449468859,0.8799740793,-0.7350158991\H,0,0.8968695889,-1.9552271434,-0.1109119295\\Version= IA64L-G03RevD.01\State=2-A\HF=-419.2419201\MP2=-420.769673\RMSD=3.504e-09\Thermal= 0.\PG=C01 [X(C5H9O3)]\\@

003

1\1\GINC-LX64I15\SP\ROMP2-FC\6-311+G(3df,2p)\C5H9O3(2)\UI271AB\14-Oct-2007\0\\#P ROMP2 (FC)/6-311+G(3df,2p) scf=tight geom=check guess=read\\prh_003 ROMP2(FC)/6- 311+G(3df,2p)//B3LYP/6-31G(d) sp\\0,2\C,0,-0.0277505902,0.0377262552,- 0.0472685627\C,0,0.1762788684,0.4712077233,1.3698049765\C,0,1.5087103399,0.00461522,1.84907882 16\O,0,2.1843401356,-0.4579343553,0.6722509748\C,0,1.1952400179,-0.8770173547,-0.2776908463\ C,0,1.8265015929,-0.7721345634,-1.6594688821\O,0,2.2971189348,0.5416261228,-1.9121407297\O,0,- 1.2100590945,-0.7358455602,-0.2882811679 \H,0,1.4268679095,-0.8167245669,2.5869127405\ H,0,2.1263637001,0.7889709059,2.3093196016\ H,0,-1.9457551549,-0.1196391308,-0.4214651927\ H,0,2.6382945965,-1.5113305453,-1.7458077826\H,0,1.0742797568,-1.0023555858,-2.4207855899\H,0, 2.8297481416,0.7708444665,-1.1310897911 \H,0,-0.5604888173,0.9785579837,1.9819781925\

H,0,0.0124972311,0.8887226784,-0.7465389528 \H,0,0.8781013531,-1.9131860983,-0.0782231644\\Version=IA64L-G03RevD.01\State=2-A\HF=-419.241994\MP2=-420.7690937\RMSD=3.679e-09\Thermal=0.\PG=C01 [X(C5H9O3)]\\@

010

1\1\GINC-LX64I35\SP\ROMP2-FC\6-311+G(3df,2p)\C5H9O3(2)\UI271AB\14-Oct-2007\0\\#P ROMP2(FC)/6-311+G(3df,2p) scf=tight geom=check guess=read\\prh_010 ROMP2(FC)/6-311+G(3df,2p)//B3LYP/6-31G(d) sp\\0,2\C,0,-0.0258529165,0.0328574083,-0.0553545598\C,0,0.1474220038,0.4088665523,1.3751149799\C,0,1.5103017623,0.0190740792,1.8358732379 \O,0,2.1937037642,-0.4245349776,0.6536060985\C,0,1.208122273,-0.8784734276,-0.2841565067 \C,0,1.8395854765,-0.7912254797,-1.667173965\O,0,2.2744510818,0.527576312,-1.953531907 \O,0,-1.2667911481,-0.6352697868,-0.2869208502 \H,0,1.4838969843,-0.7945731672,2.5854678177\H,0,2.0943623283,0.8405626639,2.2755743395 \H,0,-1.50182081,-0.5018932533,-1.217721542\H,0,2.6702872653,-1.5109843135,-1.7351943564 \H,0,1.1007251222,-1.0621168911,-2.4302024862\H,0,2.798005397,0.7919845499,-1.1773425306 \H,0,-0.6246598388,0.8484301502,1.9941214861\H,0,0.0537655735,0.9157058696,-0.7133679915\H,0,0.9051512381,-1.9148275489,-0.0630580229\\Version=IA64L-G03RevD.01\State=2-A\HF=-419.2415372\MP2=-420.7688639\RMSD=2.834e-09\Thermal=0.\PG=C01 [X(C5H9O3)]\\@

011

1\1\GINC-LX64I61\SP\ROMP2-FC\6-311+G(3df,2p)\C5H9O3(2)\UI271AB\15-Oct-2007\0\\#P ROMP2(FC)/6-311+G(3df,2p) scf=tight geom=check guess=read\\prh_011 ROMP2(FC)/6-311+G(3df,2p) // B3LYP/6-31G(d) sp\\0,2\C,0,0.9621822369,-0.7004598441,0.3526554111\C,0,1.8732874092,0.4790310508,0.2357207222 \C,0,1.0823276581,1.6954550212,-0.1089820712\O,0,-0.2882572267,1.2850080712,-0.0266826976\C,0,-0.3485726723,-0.1295106573,-0.2445826701\C,0,-1.6376459436,-0.6331723949,0.3815066494 \O,0,-2.7773989533,-0.0264091686,-0.2018465554\O,0,1.4109280337,-1.8971191445,-0.2767926316 \H,0,1.3104997889,2.074773541,-1.1239668124\H,0,1.2282127691,2.5361093271,0.5846522261 \H,0,1.6817258355,-1.6607431186,-1.1794505265\H,0,-1.7271643528,-1.7117008568,0.2169447602 \H,0,-1.5962268959,-0.4538100752,1.469055759\H,0,-2.6281719419,0.9315903945,-0.1420375922 \H,0,2.9364466152,0.4603668658,0.4455390646\H,0,0.8064833309,-0.9947216137,1.402359365\H,0,-0.3634521087,-0.3417615826,-1.328429415\\Version=IA64L-G03RevD.01\State=2-A\HF=-419.2418787\MP2=-420.7683399\RMSD=4.178e-09\Thermal=0.\PG=C01 [X(C5H9O3)]\\@

007

1\1\GINC-LX64I50\SP\ROMP2-FC\6-311+G(3df,2p)\C5H9O3(2)\UI271AB\14-Oct-2007\0\\#P ROMP2(FC)/6-311+G(3df,2p) scf=tight geom=check guess=read\\prh_007 ROMP2(FC)/6-311+G(3df,2p) // B3LYP/6-31G(d) sp\\0,2\C,0,-0.01378645,0.0322602828,-0.0600758672\C,0,0.1261987059,0.3558013554,1.3881927051 \C,0,1.5121463676,0.0248954732,1.8344166086\O,0,2.2139856205,-0.3672815929,0.6437199994 \C,0,1.2414386909,-0.8475860976,-0.2781063339\C,0,1.8037134869,-0.7744066823,-1.6866728103 \O,0,0.734014006,-1.163736992,-2.5539102066\O,0,-1.2298325304,-

0.6272070215,-0.3729371462 \H,0,1.5254124891,-0.7984384514,2.5742573004\H,0,2.0599530225, 0.8666409482,2.2827242637 \H,0,-1.1516486845,-0.8666694858,-1.3131069053\H,0,2.1325632103, 0.2559248762,-1.8886634006\H,0,2.6716750555,-1.4404052732,-1.7934546072\H,0,1.0315455953,- 1.0689894648,-3.4703389635\H,0,-0.6663567815,0.7351533657,2.0208112537\H,0,0.081150198, 0.9516982921,-0.6737100469\H,0,0.9546385818,-1.887233541,-0.0476498646\\Version=IA64L-G03Rev D.01\State=2-A\HF=-419.2414555\MP2=-420.7676499\RMSD=3.302e-09\Thermal=0.\PG=C01[X(C5H9O 3)]\\@

008

1\1\GINC-LX64I41\SP\ROMP2-FC\6-311+G(3df,2p)\C5H9O3(2)\UI271AB\14-Oct-2007\0\\#P ROMP2 (FC)/6-311+G(3df,2p) scf=tight geom=check guess=read\\prh_008 ROMP2(FC)/6-311+G(3df,2p) // B3LYP/6-31G(d) sp\\0,2\C,0,-0.0211454932,0.0567281301,-0.0388888795\C,0,0.1072816467, 0.3141190783,1.4251558741 \C,0,1.5062203412,-0.0060836891,1.8421586949\O,0,2.1942139062,- 0.3591614151,0.6317935025 \C,0,1.2203293473,-0.8357679043,-0.2885245381\C,0,1.7379719775,- 0.7834344572,-1.7201180145 \O,0,0.7956712774,-1.3680781646,-2.6061815239\O,0,-1.2465395104,- 0.5124522725,-0.5045330479 \H,0,1.5480836632,-0.8439073952,2.5640716119\H,0,2.0399666676, 0.8383248689,2.3012642357 \H,0,-1.4103032372,-1.3156125788,0.0182837721\H,0,1.9655048842, 0.2625266648,-1.9854484843 \H,0,2.6644676505,-1.3586615558,-1.811432151\H,0,-0.085138484,- 1.0337472424,-2.3614613086 \H,0,-0.6352328272,0.8062277695,2.0424867001\H,0, 0.0716021496,0.9882053613,-0.6206834318\H,0,0.9433894793,-1.8816976288,-0.0568731331\\Version= IA64L-G03RevD.01\State=2-A\HF=-419.239385\MP2=-420.76687\RMSD=3.706e-09\Thermal= 0.\PG=C01 [X(C5H9O3)]\\@

013

1\1\GINC-LX64I18\SP\ROMP2-FC\6-311+G(3df,2p)\C5H9O3(2)\UI271AB\15-Oct-2007\0\\#P ROMP2 (FC)/6-311+G(3df,2p) scf=tight geom=check guess=read\\prh_013 ROMP2(FC)/6-311+G(3df,2p) // B3LYP/6-31G(d) sp\\0,2\C,0,-0.531127115,0.8135608388,0.3214658565\C,0,-1.7146875959,- 0.0535155139,0.6059116124 \C,0,-1.4905756361,-1.4104274699,0.0297704534\O,0,-0.1152848292,- 1.429849542,-0.3580884004 \C,0,0.2913581466,-0.0905952638,-0.6264453368\C,0,1.7982530193, 0.0245890195,-0.4885521434 \O,0,2.1633629638,-0.1329120216,0.8769232874\O,0,-0.8169621892, 2.036668316,-0.3748813801 \H,0,-2.1431271355,-1.603746795,-0.8453079104\H,0,-1.655302555,- 2.2343405839,0.7391312675 \H,0,-1.1104999522,2.6862847572,0.2813537354\H,0,2.2662367211,- 0.7450473061,-1.1204183551 \H,0,2.0952553427,1.012982458,-0.8743190849\H,0,3.1298566991,- 0.1045576446,0.9298840279 \H,0,-2.6187474344,0.2671708912,1.1110572327\H,0,0.0487874853, 1.0317652808,1.2311798574\H,0,0.0192883524,0.196565259,-1.6570914778\\Version=IA64L-G03Rev D.01\State=2-A\HF=-419.2378506\MP2=-420.7637524\RMSD=3.847e-09\Thermal=0.\PG=C01 [X(C5H9O3)]\\@

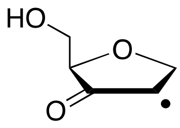

008

1\1\GINC-NODE40\SP\ROMP2-FC\6-311+G(3df,2p)\C5H7O3(2)\ZIP06\21-Feb-2013\0\\#p ROMP2 (FC)/6-311+G(3df,2p) scf=tight\\R2Ket3dRibo_008\\0,2\C,0,-1.681183,0.160792,0.491391\C,0,0.312456,-0.01999,-0.796039\C,0,-1.468844,-1.261489,0.133409 \H,0,-2.521594,0.55085,1.052732\H,0,-1.474414,-1.931709,1.006661\H,0,-2.251564,-1.631637,-0.553843\C,0,1.769367,0.071348,-0.341093\H,0,2.09189,1.114898,-0.408816\H,0,2.401151,-0.533763,-1.010094\O,0,1.906817,-0.328918,1.008591\H,0,1.518218,-1.218623,1.052521\O,0,-0.183203,-1.335495,-0.501476\C,0,-0.617321,0.952116,-0.054966\O,0,-0.460816,2.171597,0.010765\H,0,0.24708,0.175845,-1.878413\\Version=AM64L-G03RevD.01\State=2-A\HF=-418.0848229\MP2=-419.5829335\RMSD=8.708e-09\Thermal=0.\PG=C01 [X(C5H7O3)]\\@

009

1\1\GINC-NODE41\SP\ROMP2-FC\6-311+G(3df,2p)\C5H7O3(2)\ZIP06\21-Feb-2013\0\\#p ROMP2 (FC)/6-311+G(3df,2p) scf=tight\\R2Ket3dRibo_009\\0,2\C,0,-1.650765,0.880485,0.199041\C,0,0.174008,-0.500893,-0.437132\C,0,-2.116624,-0.522511,0.088878\H,0,-2.264835,1.734736,0.458081\H,0,-2.627159,-0.875634,0.99858\H,0,-2.835613,-0.647272,-0.742625\C,0,1.411022,-0.925934,0.372034\H,0,1.628775,-1.980236,0.17661\H,0,1.189196,-0.822212,1.447048\O,0,2.539302,-0.175908,-0.024238\H,0,2.289245,0.764229,0.046499\O,0,-0.952931,-1.324808,-0.135318 \C,0,-0.248353,0.928484,-0.093458\O,0,0.511223,1.904927,-0.047011\H,0,0.423909,-0.5651,-1.507829\\Version=AM64L-G03RevD.01\State=2-A\HF=-418.0850069\MP2=-419.5823908\RMSD=4.254e-09\Thermal=0.\PG=C01 [X(C5H7O3)]\\@

002

1\1\GINC-NODE41\SP\ROMP2-FC\6-311+G(3df,2p)\C5H7O3(2)\ZIP06\21-Feb-2013\0\\#p ROMP2 (FC)/6-311+G(3df,2p) scf=tight\\R2Ket3dRibo_002\\0,2\C,0,-1.883403,-0.277387,0.273024\C,0,0.333768,0.134439,-0.474966\C,0,-1.186895,-1.575908,0.125985\H,0,-2.910618,-0.146643,0.591929\H,0,-1.165694,-2.153242,1.065388\H,0,-1.672035,-2.222503,-0.626541\C,0,1.511907,0.60149,0.381371\H,0,1.256965,0.471637,1.446111\H,0,1.688777,1.66596,0.201622 \O,0,2.697278,-0.090834,0.041344\H,0,2.485547,-1.036391,0.114569\O,0,0.153605,-1.279138,-0.285821\C,0,-0.993833,0.79007,-0.08556\O,0,-1.217688,2.002372,-0.077923\H,0,0.562233,0.325747,-1.533012\\Version=AM64L-G03RevD.01\State=2-A\HF=-418.0844151\MP2=-419.5816695\RMSD=7.903e-09\Thermal=0.\PG=C01 [X(C5H7O3)]\\@

005

1\1\GINC-NODE41\SP\ROMP2-FC\6-311+G(3df,2p)\C5H7O3(2)\ZIP06\21-Feb-2013\0\\#p ROMP2 (FC)/6-311+G(3df,2p) scf=tight\\R2Ket3dRibo_005\\0,2\C,0,-1.56561,0.101844,0.59268\C,0,0.300305,-0.002083,-0.873023\C,0,-1.26866,-1.315166,0.27418\H,0,-2.327878,0.450366,1.27911\H,0,-0.872282,-1.849039,1.156013\H,0,-2.150757,-1.872755,-0.075535 \C,0,1.720116,0.013624,-0.317114\H,0,2.136489,1.020201,-0.478055\H,0,2.331514,-0.713946,-0.87157\ O,0,1.646441,-0.313978,1.065368 \H,0,2.540235,-0.269522,1.435435 \O,0,-0.293801,-1.296301,-0.775795\C,0,-0.623755,0.944345,-

0.083893\O,0,-0.523176,2.171298,-0.048536\H,0,0.332579,0.291158,-1.930684\\Version=AM64L-G03RevD.01\State=2-A\HF=-418.0842134\MP2=-419.5815823\RMSD=7.747e-09\Thermal=0.\PG=C01 [X(C5H7O3)]\\@

004

1\1\GINC-NODE40\SP\ROMP2-FC\6-311+G(3df,2p)\C5H7O3(2)\ZIP06\21-Feb-2013\0\\#p ROMP2 (FC)/6-311+G(3df,2p) scf=tight\\R2Ket3dRibo_004\\0,2\C,0,-1.546414,0.639206,0.457695\C,0,0.22769,-0.298885,-0.818508\C,0,-1.753098,-0.822106,0.349717\H,0,-2.182116,1.329199,0.999697\H,0,-1.757787,-1.309824,1.339996\H,0,-2.71582,-1.073958,-0.128516\C,0,1.6526,-0.581257,-0.327641\H,0,2.345967,0.065446,-0.884877\H,0,1.898505,-1.623515,-0.550418\O,0,1.796039,-0.418369,1.075317\H,0,1.846514,0.535317,1.247406\O,0,-0.675842,-1.337292,-0.43516\C,0,-0.327568,0.993845,-0.206003\O,0,0.230566,2.094526,-0.244849\H,0,0.239382,-0.2184,-1.917317\\Version=AM64L-G03RevD.01\State=2-A\HF=-418.0841091\MP2=-419.5812724\RMSD=7.968e-09\Thermal=0.\PG=C01 [X(C5H7O3)]\\@

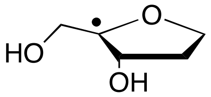

003

1\1\GINC-LX64I32\SP\ROMP2-FC\6-311+G(3df,2p)\C5H9O3(2)\UI271AB\19-Oct-2007\0\\#P ROMP2 (FC)/6-311+G(3df,2p) scf=tight geom=check guess=read\\prg_3 ROMP2(FC)/6-311+G(3df,2p)//B3LYP/6-31G(d) sp\\0,2\C,0,1.6637487602,0.6513380952,0.4110277597\C,0,1.9146200992,-0.8382272439,0.1815751265 \O,0,0.6016726847,-1.4002058168,-0.0429325181\C,0,-0.1790319052,-0.4332988439,-0.6419313983 \C,0,0.4422457348,0.9424251801,-0.5034773748\C,0,-1.6422660868,-0.6530689904,-0.5290462401 \O,0,-2.138729999,-0.2730263531,0.790045818\O,0,-0.4520425097,1.926212216,-0.0040677131 \H,0,2.3535630869,-1.3677903846,1.0303828074\H,0,2.5330376013,-1.0196763458,-0.7076391935 \H,0,0.7833776775,1.3333823894,-1.4721822747\H,0,-1.0666312909,1.4679124825,0.6015229858\H,0,-2.1808038945,-0.0051622809,-1.2237020018 \H,0,-1.9044186936,-1.6970507328,-0.7404636398 \H,0,-1.6971504306,-0.8640311929,1.4221428156\H,0,2.5328761009,1.2762942768,0.1881597082\H,0,1.3630488222,0.8372722376,1.448526861\\Version=IA64L-G03Rev D.01\State=2-A\HF=-419.2482543\MP2=-420.7808311\RMSD=5.478e-09\Thermal=0.\PG=C01 [X(C5H9O3)]\\@

001

1\1\GINC-LX64I15\SP\ROMP2-FC\6-311+G(3df,2p)\C5H9O3(2)\UI271AB\20-Oct-2007\0\\#P ROMP2 (FC)/6-311+G(3df,2p) scf=tight geom=check guess=read\\prg_1 ROMP2(FC)/6-311+G(3df,2p)//B3LYP/6-31G(d) sp\\0,2\C,0,0.1158003238,-0.0477140372,-0.048798309\C,0,0.0725173178,-0.2405872758,1.4663213971\O,0,1.4317745896,-0.0183017834,1.9052014652 \C,0,2.0236341524,0.858942824,1.0319214711\C,0,1.1982312674,1.0512282048,-0.221287978 \C,0,3.5013471264,0.9081541728,1.0423498185\O,0,4.0149832157,-0.137533502,0.1616072812 \O,0,1.9490674362,0.9705906456,-1.4232807254\H,0,-0.2129249084,-1.242641256,1.7943178948 \H,0,-0.5820051332,0.4932151883,1.956278988\H,0,0.7220214389,2.0424606754,-0.2488027045 \H,0,2.6758250301,0.3403882578,-

1.2516608365\H,0,3.8379584253,1.888126572,0.6778983726\H,0,3.8800651303,0.757837031,2.0611779065\H,0,4.9575900343,0.0418416283,0.0064318601 \H,0,-0.852190097,0.2233857747,-0.4792920913 \H,0,0.4741740471,-0.9578970468,-0.5423453981\\Version=IA64L-G03RevD.01 \State=2-A\HF=-419.2460879\MP2=-420.778568\RMSD=6.424e-09\Thermal=0.\PG=C01 [X(C5H9O3)]\\@

012

1\1\GINC-LX64I61\SP\ROMP2-FC\6-311+G(3df,2p)\C5H9O3(2)\UI271AB\20-Oct-2007\0\\#P ROMP2 (FC)/6-311+G(3df,2p) scf=tight geom=check guess=read\\prg_12 ROMP2(FC)/6-311+G(3df,2p)//B3LYP/6-31G(d) sp\\0,2\C,0,0.0887949969,-0.1325920979,0.0912648631\C,0,-0.1300136064,0.1287129362,1.5851443665\O,0,1.17367097,0.4445614379,2.1043785054\C,0,1.9020280026,1.0326932255,1.1002846927\C,0,1.2990265968,0.7697261278,-0.2490079628\C,0,3.352966626,1.2621198308,1.3385440478\O,0,4.1661732083,0.1050893287,1.0941015691\O,0,2.2728072241,0.1196104415,-1.0945088194\H,0,-0.5070316339,-0.7322880972,2.1416418797\H,0,-0.7989471627,0.983309941,1.7567751418\H,0,0.9839411478,1.7003954766,-0.7489277426 \H,0,2.0188226434,0.2586512016,-2.0204035447\H,0,3.6849847906,2.1059793137,0.7124070494\H,0,3.520858936,1.534384752,2.384664187\H,0,3.9380510566,-0.1791567312,0.1919982808\H,0,-0.8013526287,0.0698333087,-0.5121011736\H,0,0.398952934,-1.1692246345,-0.0753626583\\Version=IA64L-G03RevD.01\State=2-A\HF=-419.2460346\MP2=-420.7775152\RMSD=5.055e-09\Thermal=0.\PG=C01 [X(C5H9O3)]\\@

013

1\1\GINC-LX64I18\SP\ROMP2-FC\6-311+G(3df,2p)\C5H9O3(2)\UI271AB\20-Oct-2007\0\\#P ROMP2 (FC)/6-311+G(3df,2p) scf=tight geom=check guess=read\\prg_13 ROMP2(FC)/6-311+G(3df,2p) //B3LYP/6-31G(d) sp\\0,2\C,0,0.2005280971,-0.1552554199,0.0664827085\C,0,-0.0972502604,0.1334527147,1.5400850109\O,0,1.1723730547,0.4991495682,2.1104012199\C,0,1.9262710594,1.0934512367,1.1275426023\C,0,1.3785809527,0.7883454321,-0.2436659323\C,0,3.3786528618,1.2738665934,1.4029451018\O,0,4.1469197052,0.077631008,1.2072419896 \O,0,2.3409934382,0.1068920611,-1.0913994252\H,0,-0.4743951111,-0.7254834836,2.0995203022\H,0,-0.7980157384,0.9712995217,1.6598159895\H,0,1.0444839094,1.692892861,-0.7735067989 \H,0,2.7120479127,0.7562394216,-1.7073793098\H,0,3.7612212409,2.08924936,0.7649690156\H,0,3.5332321128,1.5705616227,2.4444884624\H,0,3.8599892865,-0.2651928432,0.3419122654\H,0,-0.6637925953,-0.0103856674,-0.5873518148 \H,0,0.5633218254,-1.1807593362,-0.0592826044\\Version=IA64L-G03RevD.01\State=2-A\HF=-419.2451591\MP2=-420.7770852\RMSD=4.040e-09\Thermal=0.\PG=C01 [X(C5H9O3)]\\@

006

1\1\GINC-LX64I61\SP\ROMP2-FC\6-311+G(3df,2p)\C5H9O3(2)\UI271AB\20-Oct-2007\0\\#P ROMP2 (FC)/6-311+G(3df,2p) scf=tight geom=check guess=read\\prg_6 ROMP2(FC)/6-311+G(3df,2p)//B3LYP/6-31G(d) sp\\0,2\C,0,-0.0314047839,-0.0276231485,0.0014661874\C,0,-0.0102514299,-0.0238115465,1.5335188934\O,0,1.37828188,-0.1845440119,1.9139999948 \C,0,2.169572919,0.0792829305,0.8256655893\C,0,1.3609137761,0.5420597161,-0.3453920558 \C,0,3.6022235554,0.4282716858,1.0655304099\O,0,4.4231409643,0.1045641036,-0.0544737387 \O,0,1.9096712682,0.1291555958,-1.603698331\H,0,-0.5793020511,-0.8352401772,1.9938674796 \H,0,-0.3647177356,0.930318737,

1.9445012858\H,0,1.3170032493,1.6404138362,-0.4341663132 \H,0,2.1370678321,-0.8125280781,-1.502554351\H,0,3.7099616847,1.5034274601,1.3190970754 \H,0,3.9826428806,-0.1364906866,1.923472705\H,0,3.9523910627,0.4156676779,-0.8469210271 \H,0,-0.8565696478,0.5537557585,-0.4180440544\H,0,-0.100924198,-1.0516218608,-0.3837123143\\Version=IA64L-G03RevD.01\State=2-A\HF=-419.2443323\MP2=-420.7751596\RMSD=1.496e-09\Thermal=0.\PG=C01 [X(C5H9O3)]\\@

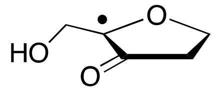

001

1\1\GINC-NODE10\SP\ROMP2-FC\6-311+G(3df,2p)\C5H7O3(2)\ZIP06\21-Feb-2013\0\\#p ROMP2 (FC)/6-311+G(3df,2p) scf=tight\\R4Ket3dRibo_001\\0,2\C,0,-1.774968,0.834646,-0.070853\C,0,0.196242,-0.481344,0.047051\C,0,-2.090354,-0.666729,-0.052886\H,0,-2.231328,1.376297,0.763671\H,0,-2.093547,1.324828,-0.997015\H,0,-2.64865,-0.988188,0.82975\H,0,-2.603001,-1.025846,-0.947735\C,0,1.608256,-0.963676,0.173436\H,0,1.736819,-1.876457,-0.422356\H,0,1.789805,-1.252439,1.227296\O,0,2.539935,-0.005637,-0.276174\H,0,2.18369,0.875118,-0.04315\O,0,-0.800495,-1.374865,-0.005451\C,0,-0.245087,0.86905,0.043785\O,0,0.473268,1.882378,0.124918\\Version=AM64L-G03RevD.01 \State=2-A\HF=-418.0958574\MP2=-419.6032351\RMSD=2.454e-09\Thermal=0.\PG=C01 [X(C5H7O3)]\\@

002

1\1\GINC-NODE19\SP\ROMP2-FC\6-311+G(3df,2p)\C5H7O3(2)\ZIP06\21-Feb-2013\0\\#p ROMP2 (FC)/6-311+G(3df,2p) scf=tight\\R4Ket3dRibo_002\\0,2\C,0,-2.019312,-0.045908,-0.119497\C,0,0.331438,0.025089,0.210378\C,0,-1.410159,-1.450366,-0.078284\H,0,-2.693595,0.148482,0.7219\H,0,-2.572834,0.153511,-1.041577\H,0,-1.826046,-2.10443,0.690618\H,0,-1.447397,-1.965456,-1.042295\C,0,1.763489,0.385256,0.410511\H,0,2.067384,0.154939,1.44595\H,0,1.861328,1.463122,0.261086\O,0,2.628297,-0.251217,-0.52627\H,0,2.514141,-1.208028,-0.411793\O,0,0.013439,-1.282505,0.249103\C,0,-0.789453,0.878034,-0.020463\O,0,-0.786611,2.109376,-0.102803\\Version=AM64L-G03RevD.01\State=2-A\HF=-418.0958187\MP2=-419.6011761\RMSD=7.948e-09\Thermal=0.\PG=C01 [X(C5H7O3)]\\@

005

1\1\GINC-NODE21\SP\ROMP2-FC\6-311+G(3df,2p)\C5H7O3(2)\ZIP06\21-Feb-2013\0\\#p ROMP2 (FC)/6-311+G(3df,2p) scf=tight\\R4Ket3dRibo_005\\0,2\C,0,2.017868,0.080395,-0.154499\C,0,-0.32327,-0.049071,0.224627\C,0,1.5286,-1.369517,-0.087072\H,0,2.516833,0.320524,-1.097965\H,0,2.705636,0.336252,0.658964\H,0,1.604352,-1.897923,-1.041267\H,0,2.004135,-1.971693,0.690096\C,0,-1.763019,0.228136,0.459688\H,0,-1.873368,1.320945,0.4915\H,0,-2.059093,-0.181332,1.440161\O,0,-2.530161,-0.364035,-0.589665\H,0,-3.463151,-0.176365,-0.402521\O,0,0.099346,-1.321826,0.245409\C,0,0.720461,0.897928,-0.012637\O,0,0.615917,2.126157,-0.070695\\Version=AM64L-G03RevD.01\State=2-A\HF=-418.0938533\MP2=-419.5991268\RMSD=2.111e-09\Thermal=0.\PG=C01 [X(C5H7O3)]\\@

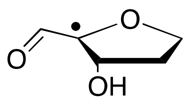

004

1\1\GINC-NODE8\SP\ROMP2-FC\6-311+G(3df,2p)\C5H7O3(2)\ZIP06\21-Feb-2013\0\\#p ROMP2(FC)/6-311+G(3df,2p) scf=tight\\R4Ket5dRibo_004\\0,2\C,0,-1.64331,0.831496,-0.124957\C,0,-0.19165, 0.844855,0.382688\C,0,0.235945,-0.566976,0.058938\C,0,-2.043051,-0.635861,0.04905\H,0,-1.632146, 1.112347,-1.182952\H,0,-2.301208,1.515632,0.416165 \H,0,-0.179503,0.975383,1.48163\H,0,-2.42324,-0.855659,1.053169\H,0,-2.751798,-1.010798,-0.691271\H,0,1.747747,-2.066845,-0.303465\C,0,1.573622,-1.001987,-0.071124\O,0,2.526126,-0.199397,0.070271\O,0,0.584677,1.836759,-0.245811\H,0,1.512414, 1.542612,-0.136265\O,0,-0.806004,-1.392591,-0.125033\\Version=AM64L-G03RevD.01\State=2-A\HF=-418.091951\MP2=-419.6018749\RMSD=2.047e-09\Thermal=0.\PG=C01 [X(C5H7O3)]\\@

005

1\1\GINC-NODE6\SP\ROMP2-FC\6-311+G(3df,2p)\C5H7O3(2)\ZIP06\21-Feb-2013\0\\#p ROMP2(FC)/6-311+G(3df,2p) scf=tight\\R4Ket5dRibo_005\\0,2\C,0,-1.708057,0.533303,0.549055\C,0,-0.209963, 0.843064,0.41771\C,0,0.355555,-0.518378,0.10555 \C,0,-1.87884,-0.74267,-0.281905\H,0,-2.316674, 1.364367,0.18579\H,0,-1.958591,0.343079,1.59753 \H,0,0.218339,1.255111,1.339598\H,0,-2.629092,-1.439991,0.095198\H,0,-2.075747,-0.529708,-1.337706\H,0,2.021383,-1.85121,-0.242801\C,0,1.737353,-0.812398,0.005831\O,0,2.591241,0.081738,0.176867\O,0,-0.000718,1.740784,-0.67326\H,0,0.965293, 1.816266,-0.767011\O,0,-0.590672,-1.419453,-0.209612\\Version=AM64L-G03RevD.01\State=2-A\HF=-418.0924276\MP2=-419.6013255\RMSD=2.865e-09\Thermal=0.\PG=C01 [X(C5H7O3)]\\@

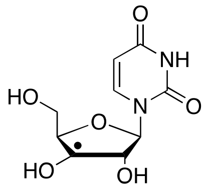

007

1\1\GINC-NODE18\SP\ROMP2-FC\6-311+G(3df,2p)\C9H11N2O6(2)\ZIP06\14-Jul-2011\0\\#p ROMP2 (FC)/6-311+G(3df,2p) scf=tight\\ROMP2(FC)/6-311+G(3df,2p) Rad3UriU_007\\0,2\O,0,1.926511,-2.208371,1.270194 \C,0,2.709744,-2.139184,0.087528\H,0,2.365779,-2.95339,-0.557732\H,0,3.778963,-2.298107,0.294731 \C,0,2.544047,-0.80789,-0.644567\H,0,3.234483,-0.759161,-1.496184\O,0,1.17333,-0.743813,-1.157184\C,0,0.513272,0.40414,-0.690454\H,0,0.482601,1.188906,-1.457331\N,0,-0.891939, 0.051416,-0.379314\C,0,-1.344513,-1.25198,-0.343406\H,0,-0.575114,-1.990514,-0.519673\C,0,-2.638505, -1.564437,-0.106666 \H,0,-2.971959,-2.593144,-0.081572\C,0,-3.620423,-0.518652,0.123742\O,0,-4.812207,-0.654663,0.338662\N,0,-3.049345,0.781065,0.089612 \H,0,-3.677625,1.553038,0.28485\C,0,-1.735581,1.121361,-0.112543\O,0,-1.330734,2.285896,-0.060652\C,0,2.693405,0.412059,0.225716\C,0, 1.308881,0.931299,0.537925\H,0,0.891726,0.462515,1.443286\O,0,1.354012,2.3338,0.644967\H,0,0.443208,2.653772,0.470274\H,0,2.324759,-1.592021,1.906627\ O,0,3.620478,1.334468,-0.143735\H,0, 3.259085,2.201913,0.138957\\Version=AM64L-G03RevD.01 \State=2-A\HF=-905.5846596\MP2=-908.8218201\RMSD=2.893e-09\Thermal=0.\PG=C01 [X(C9H11N2O6)]\\@

022

1\1\GINC-NODE18\SP\ROMP2-FC\6-311+G(3df,2p)\C9H11N2O6(2)\ZIP06\15-Jul- 2011\0\\#p ROMP2 (FC)/6-311+G(3df,2p) scf=tight\\ROMP2(FC)/6-311+G(3df, 2p) Rad3UriU_022\\0,2\O,0,-2.285706,-2.329372,0.061985\C,0,-2.712415,- 1.499611,1.139199\H,0,-2.283535,-1.82963,2.095077\H,0,-3.799001,-1.618481,1.198228\C,0,-2.379694,-0.014546,0.945042\H,0,-3.060586,0.566498,1.589601\O,0,-1.032984, 0.263293,1.415604\C,0,-0.353773,1.137433,0.541875\H,0,-0.185804,2.102782,1.030555\N,0,0.996293, 0.605223,0.266919\C,0,2.11017,1.307757,0.688737\H,0,1.888563,2.254118,1.169389\C,0,3.377228,0.874128,0.51973\H,0,4.223897,1.455729,0.858585\C,0,3.628949,-0.40681,-0.120709\O,0,4.710451,-0.923813,-0.337337\N,0,2.432933,-1.063455,-0.49 9637\H,0,2.541269,-1.964415,-0.953142\C,0,1.132787,-0.638642,-0.346678\O,0,0.179322,-1.303814,-0.742073\C,0,-2.394334,0.469212,-0.479975\C,0 ,-1.237311,1.384129,-0.709398\H,0,-0.721012,1.128239,-1.642779\O,0,-1.551316,2.787505,-0.674686\H,0,-2.304928,2.920136,-1.271989\H,0,-1.333783,-2.153304,-0.094505\O,0,-2.904985,-0.259628,-1.499694\H,0,-2.917544,-1.205714,-1.207328\\Version=AM64L-G03RevD.01\State=2-A\HF=-905.5789256\MP2=-908.8202557 \RMSD=2.968e-09\Thermal=0.\PG=C01 [X(C9H11N2O6)]\\@

029

1\1\GINC-NODE18\SP\ROMP2-FC\6-311+G(3df,2p)\C9H11N2O6(2)\ZIP06\16-Jul-2011\0\\#p ROMP2 (FC)/6-311+G(3df,2p) scf=tight\\ROMP2(FC)/6-311+G(3df,2p) Rad3UriU_029\\0,2\O,0,-3.174629, 2.897774,-0.242141\C,0,-3.170195,1.697932,0.510757\H,0,-4.213764,1.47463,0.752579\H,0,-2.616707, 1.804125,1.457313\C,0,-2.586445,0.52603,-0.273961 \H,0,-3.129985,0.42653,-1.226013\O,0,-1.1923, 0.85591,-0.556944\C,0,-0.42012,-0.323468,-0.539013\H,0,-0.422948,-0.826197,-1.51643\N,0,0.979307, 0.052376,-0.231956\C,0,1.369564,1.358389,-0.006733\H,0,0.557225,2.070491,-0.017804\C,0,2.656142, 1.712322,0.207152\H,0,2.933211,2.742843,0.384382\C,0,3.70765,0.70896,0.198886\O,0,4.900405,0.885477,0.371439\N,0,3.207492,-0.596737,-0.039808\H,0,3.890856,-1.346452,-0.041757\C,0,1.906244,-0.984855, -0.243021\O,0,1.585608,-2.160842,-0.420297\C,0,-2.517006,-0.793069,0.441742\C,0,-1.084568,-1.265823, 0.502139\H,0,-0.620412,-1.089413,1.488322\O,0,-1.055857,-2.632618,0.162322\H,0,-0.118933,-2.847979,-0.024154\H,0,-2.257682,3.06579,-0.511001\O,0,-3.490327,-1.707722,0.201333\H,0,-3.05925,-2.586172, 0.243524\\Version=AM64L-G03RevD.01\State=2-A \HF=-905.5852974\MP2=-908.8197226 \RMSD=2.897e-09\Thermal=0.\PG=C01 [X(C9H11N2O6)]\\@

001

1\1\GINC-NODE21\SP\ROMP2-FC\6-311+G(3df,2p)\C9H11N2O6(2)\ZIP06\13-Jul-2011\0\\#p ROMP2 (FC)/6-311+G(3df,2p) scf=tight\\ROMP2(FC)/6-311+G(3df,2p) Rad3UriU_001\\0,2\O,0,-1.494062, 2.134936,1.206941\C,0,-2.513195,2.208292,0.217125\H,0,-2.325978,3.035297,-0.485554\H,0,-3.50771, 2.354962,0.664074\C,0,-2.530506,0.895289,-0.553957\H,0,-3.286934,0.955786,-1.344657\O,0,-1.221435, 0.718342,-1.196053\C,0,-0.571312,-0.423583,-0.697468\H,0,-0.551434,-1.219578,-1.451854\N,0,0.843855, -0.086432,-0.396115\C,0,1.326314,1.204235,-0.423435\H,0,0.573984,1.94908,-0.638759\C,0,2.626157, 1.500751,-0.194222\H,0,2.982885,2.521624,-0.221371\C,0,3.583188,0.447606,0.094965\O,0,4.776492, 0.56899,0.314457\N,0,2.98487,-0.838911,0.114788 \H,0,3.595835,-1.615517,0.343236\C,0,1.665103,-1.161679,-0.085907\O,0,1.240847,-2.316597,0.002576\C,0,-2.730701,-0.345299,0.268285\C,0,-1.367255,-0.927084,0.546202\H,0,-0.922141,-0.488798,1.45055\O,0,-1.468919,-2.330354,0.625902\H,0,-0.563468,-

2.676769,0.483439\H,0,-1.566982,2.911715,1.780729\O,0,-3.682175,-1.217094,-0.169824\H,0,-3.36188,-2.107355,0.087943\\Version=AM64L-G03RevD.01\State=2-A\HF=-905.5848686\MP2=-908.8215667 \RMSD=3.343e-09\Thermal=0.\PG=C01 [X(C9H11N2O6)]\\@

016

1\1\GINC-NODE18\SP\ROMP2-FC\6-311+G(3df,2p)\C9H11N2O6(2)\ZIP06\14-Jul-2011\0\\#p ROMP2 (FC)/6-311+G(3df,2p) scf=tight\\ROMP2(FC)/6-311+G(3df,2p) Rad3UriU_016\\0,2\O,0,-4.211665,-1.877897,-0.678377\C,0,-2.843603,-1.920309,-0.325315 \H,0,-2.184327,-1.963903,-1.207399\H,0,-2.696776,-2.838755,0.250894\C,0,-2.463526,-0.70393,0.527388\H,0,-3.089326,-0.675897,1.42685\O,0,-1.059814,-0.851885,0.919598\C,0,-0.342333,0.32417,0.631498\H,0,-0.295974,0.995608,1.500082\N,0, 1.050728,-0.061133,0.296355\C,0,1.471028,-1.377611,0.268384\H,0,0.685443,-2.095247,0.458477 \C,0,2.753939,-1.730532,0.031112\H,0,3.05569,-2.769167,0.011788\C,0,3.768888,-0.715501,-0.195286 \O,0,4.95384,-0.888897,-0.417686\N,0,3.24084,0.600287,-0.135169\H,0,3.897553,1.356287,-0.297227 \C,0,1.942256,0.984079,0.086676\O,0,1.594597,2.167184,0.097624\C,0,-2.529536,0.609157,-0.200261 \C,0,-1.11379,1.040966,-0.515429\H,0,-0.761137,0.632949,-1.477431\O,0,-1.055366,2.444877,-0.4883 \H,0,-0.117285,2.680076,-0.321199\H,0,-4.351261,-1.057097,-1.18011\O,0,-3.360347,1.565962, 0.289652\H,0,-2.933479,2.423368,0.074275\\Version=AM64L-G03RevD.01\State=2-A\HF=-905.583035 \MP2=-908.8191918\RMSD=4.619e-09\Thermal=0.\PG=C01 [X(C9H11N2O6)]\\@

006

1\1\GINC-NODE21\SP\ROMP2-FC\6-311+G(3df,2p)\C9H11N2O6(2)\ZIP06\14-Jul-2011\0\\#p ROMP2 (FC)/6-311+G(3df,2p) scf=tight\\ROMP2(FC)/6-311+G(3df,2p) Rad3UriU_006\\0,2\O,0,1.991577,-2.16485,1.420652\C,0,2.813306,-2.110952,0.267428\H,0,2.669809,-2.996248,-0.372276\H,0,3.849477,-2.111647,0.618523\C,0,2.573974,-0.847801,-0.560294\H,0,3.205838,-0.860472,-1.457712\O,0,1.160025,-0.882362,-0.979959\C,0,0.524796,0.339176,-0.673613\H,0,0.534368,1.024956,-1.532361\N,0,-0.896705, 0.032732,-0.356996\C,0,-1.430608,-1.230966,-0.535689\H,0,-0.715078,-1.968419,-0.870676\C,0,-2.733577,-1.518884,-0.321954\H,0,-3.118441,-2.519504,-0.466548\C,0,-3.658915,-0.477767,0.092492\ O,0,-4.84963,-0.593516,0.321491 \N,0,-3.027707,0.785523,0.212703\H,0,-3.621433,1.560564,0.48827\C,0,-1.708288,1.104413,0.000746\O,0,-1.286545,2.255263,0.121009\C,0, 2.72265,0.45357,0.167362\C,0,1.34507,0.979754,0.488878\H,0,0.973791,0.574198,1.443509\O,0,1.390823 ,2.383254,0.48018\H,0,0.461188,2.686769,0.416847 \H,0,1.077082,-2.23499,1.105441\O,0,3.617297, 1.345067,-0.340608\H,0,3.275459,2.230919,-0.097216\\Version=AM64L-G03RevD.01\State=2-A\HF=-905.5821793\MP2=-908.819524\RMSD=3.431e-09\Thermal=0.\PG=C01 [X(C9H11N2O6)]\\@

011

1\1\GINC-NODE21\SP\ROMP2-FC\6-311+G(3df,2p)\C9H11N2O6(2)\ZIP06\14-Jul-2011\0\\#p ROMP2 (FC)/6-311+G(3df,2p) scf=tight\\ROMP2(FC)/6-311+G(3df,2p) Rad3UriU_011\\0,2\O,0,1.776105, 3.144469,-0.202218\C,0,2.31611,1.963217,-0.772488\H,0,3.286592,2.145546,-1.260383\H,0,1.609171, 1.665808,-1.553478\C,0,2.49143,0.823163,0.238872\H,0,3.299403,1.053443,0.94285\O,0,1.268349, 0.68722,1.035416\C,0,0.561975,-0.469772,0.675698\H,0,0.521429,-1.172126,1.516422\N,0,-0.840571,-0.10292,0.346799\C,0,-1.28229,1.20542,0.315519\H,0,-0.506918,1.942152,0.490274\C,0,-2.574148,

1.525602,0.074063\H,0,-2.900213,2.556697,0.047173\C,0,-3.564435,0.486829,-0.153144\O,0,-4.754226, 0.632514,-0.374148\N,0,-3.007112,-0.818144,-0.102503 \H,0,-3.645771,-1.586638,-0.276441\C,0,-1.698183,-1.169583,0.115952\O,0,-1.308493,-2.34024,0.106638\C,0,2.685956,-0.520263,-0.396137\C,0,1.314476,-1.145837,-0.515771\H,0,0.817301,-0.857235,-1.455509\O,0,1.427132,-2.542107,-0.397926\H,0,0.515849,-2.868415,-0.246473\H,0,2.367117,3.437353,0.509659\O,0,3.677672,-1.310367,0.095382\H,0,3.372154,-2.233701,-0.024714\\Version=AM64L-G03RevD.01\State=2-A\HF=-905.5825103\MP2=-908.8192463\RMSD=3.193e-09\Thermal=0.\PG=C01 [X(C9H11N2O6)]\\@

002

1\1\GINC-NODE21\SP\ROMP2-FC\6-311+G(3df,2p)\C9H11N2O6(2)\ZIP06\13-Jul-2011\0\\#p ROMP2(FC)/6-311+G(3df,2p) scf=tight\\ROMP2(FC)/6-311+G(3df,2p) Rad3UriU_002\\0,2\O,0,1.969722, 3.114173,-0.080116\C,0,2.347893,1.933193,-0.772509\H,0,3.314121,2.049253,-1.287468\H,0,1.598805, 1.653446,-1.531106\C,0,2.493772,0.805694,0.243993\H,0,3.293321,1.042517,0.951401\O,0,1.258622, 0.686325,1.02592\C,0,0.550975,-0.471698,0.677818\H,0,0.507096,-1.167282,1.524462\N,0,-0.851602,-0.099477,0.346358\C,0,-1.291429,1.209081,0.350019\H,0,-0.51471,1.935974,0.555931\C,0,-2.580835, 1.540123,0.109339\H,0,-2.905805,2.571937,0.113374\C,0,-3.572175,0.51046,-0.152256\O,0,-4.759567, 0.666158,-0.380205\N,0,-3.019467,-0.796478,-0.126819\H,0,-3.660364,-1.55948,-0.315985\C,0,-1.71344,-1.158294,0.096391\O,0,-1.332169,-2.331144,0.073539\C,0,2.675403,-0.543094,-0.386155\C,0,1.300544,-1.160273,-0.507534\H,0,0.808686,-0.872557,-1.450471\O,0,1.403489,-2.556697,-0.383378\H,0,0.488336,-2.876605,-0.243188\H,0,1.946364,3.841118,-0.720499\O,0,3.660989,-1.338539,0.109102\H,0,3.348709,-2.259985,-0.006772\\Version=AM64L-G03RevD.01\State=2-A\HF=-905.582677\MP2=-908.8191042\RMSD=2.513e-09\Thermal=0.\PG=C01 [X(C9H11N2O6)]\\@

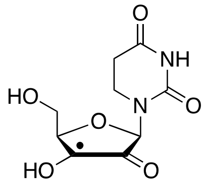

018

1\1\GINC-NODE25\SP\ROMP2-FC\6-311+G(3df,2p)\C9H11N2O6(2)\ZIP06\06-Jul-2011\0\\#p ROMP2(FC)/6-311+G(3df,2p) scf=tight\\ROMP2(FC)/6-311+G(3df,2p) RadKet2dU_018\\0,2\O,0, 2.380385,2.183409,-0.356937\C,0,2.898946,1.598852,0.807955\H,0,2.588377,2.12476,1.727119\H,0, 3.991065,1.664836,0.739789\C,0,2.520632,0.121157,0.996575 \H,0,3.187283,-0.311671,1.761325\O,0,1.152983,0.015572,1.473204\C,0,0.396285,-0.950392,0.741433\H,0,0.258255,-1.849711,1.357699\N,0,-0.940244,-0.48386,0.437261\C,0,-2.055439,-0.85152,1.320637\H,0,-1.828659,-1.824812,1.761688\C,0,-3.3567,-0.923025,0.525033\H,0,-3.316352,-1.756047,-0.189193\C,0,-3.5895,0.3508,-0.264727\O,0,-4.676169,0.78112,-0.590623\N,0,-2.410124,1.001698,-0.623158 \H,0,-2.50089,1.805849,-1.234944\C,0,-1.092063,0.57544,-0.422662\O,0,-0.172859,1.139511,-1.005255 \C,0,2.513852,-0.695856,-0.247483\H,0,1.434238,1.954449,-0.434792 \O,0,3.56023,-0.851856,-1.047093\H,0,3.24845,-1.411578,-1.791861\C,0,1.261634,-1.310813,-0.474338\O,0,0.976611,-2.055041,-1.424666\H,0,-4.217673,-1.083468,1.178403\H,0,-2.146855,-0.127052,2.142463\\Version=AM64L-G03RevD.01\State=2-A\HF=-905.6074012\MP2=-908.8484744\RMSD=5.701e-09\Thermal=0.\PG=C01 [X(C9H11N2O6)]\\@

006

1\1\GINC-NODE23\SP\ROMP2-FC\6-311+G(3df,2p)\C9H11N2O6(2)\ZIP06\06-Jul-2011\0\\#p ROMP2 (FC)/6-311+G(3df,2p) scf=tight\\ROMP2(FC)/6-311+G(3df,2p) RadKet2dU_006\\0,2\O,0,-1.904104, 2.416484,-0.122618\C,0,-2.901022,1.712474,-0.837258\H,0,-3.011391,2.102736,-1.859571\H,0,-3.848311, 1.858273,-0.309526\C,0,-2.581344,0.209028,-0.918447\H,0,-3.264024,-0.256211,-1.649791\O,0,-1.215856, 0.058606,-1.35793\C,0,-0.483962,-0.913821,-0.567334 \H,0,-0.305958,-1.806401,-1.173128\N,0,0.80639,-0.405795,-0.194345\C,0,0.932395,0.523271,0.93782 \H,0,0.003812,1.095018,1.007066\C,0,2.116923, 1.462937,0.726835\H,0,1.914711,2.151341,-0.105179 \C,0,3.384125,0.700701,0.386495\O,0,4.51077, 1.08047,0.634489\N,0,3.147772,-0.491844,-0.289137 \H,0,3.961826,-0.983807,-0.641034\C,0,1.923119,-1.011003,-0.739593\O,0,1.89645,-1.926708,-1.544656\C,0,-2.613022,-0.529027,0.372112\H,0,-1.081696, 2.261628,-0.616494\O,0,-3.662777,-0.573504,1.183428\H,0,-3.391272,-1.134794,1.94355\C,0,-1.400187,-1.207455,0.623658\O,0,-1.158744,-1.894624,1.631402\H,0,2.308594,2.067198,1.616641\H,0,1.046512,-0.039943,1.873188\\Version=AM64L-G03RevD.01\State=2-A\HF=-905.60394\MP2=-908.8461554\ RMSD=3.694e-09\Thermal=0.\PG=C01 [X(C9H11N2O6)]\\@

034

1\1\GINC-NODE25\SP\ROMP2-FC\6-311+G(3df,2p)\C9H11N2O6(2)\ZIP06\07-Jul-2011\0\\#p ROMP2 (FC)/6-311+G(3df,2p) scf=tight\\ROMP2(FC)/6-311+G(3df,2p) RadKet2dU_034\\0,2\O,0, 2.547871,2.897959,0.179326\C,0,2.616446,1.661049,-0.506161\H,0,3.593343,1.621183,-0.998378\H,0, 1.82998,1.561206,-1.264527\C,0,2.499438,0.495224,0.487179 \H,0,3.303339,0.597429,1.23146\ O,0,1.231442,0.602773,1.18977\C,0,0.427351,-0.578669,1.059643 \H,0,0.298833,-1.019902,2.055712\ N,0,-0.908291,-0.301081,0.577661\C,0,-1.959266,0.034069,1.547748\H,0,-1.758539,-0.522754,2.466409\ C,0,-3.330453,-0.341783,0.990888\H,0,-3.413961,-1.432938,0.900845\C,0,-3.549605,0.25194,-0.387806\ O,0,-4.632681,0.523897,-0.864067\N,0,-2.369717,0.443394,-1.101716\H,0,-2.465219,0.744813,-2.065548\ C,0,-1.064207,0.080306,-0.7385\O,0,-0.163361,0.110364,-1.563572\C,0,2.453123,-0.861976,-0.108479\ H,0,1.690017,2.905254,0.634666\O,0,3.421286,-1.394061,-0.8482 \H,0,3.088756,-2.277545,-1.121973\ C,0,1.241521,-1.53219,0.174917\O,0,0.950014,-2.681906,-0.184124\H,0,-4.136167,0.002149,1.64397\H,0, -1.926982,1.104876,1.796115\\Version=AM64L-G03RevD.01\State=2-A \HF=-905.6058278\MP2=-908.8460842\RMSD=6.535e-09\Thermal=0.\PG=C01 [X(C9H11N2O6)]\\@

040

1\1\GINC-NODE10\SP\ROMP2-FC\6-311+G(3df,2p)\C9H11N2O6(2)\ZIP06\08-Jul-2011\0\\#p ROMP2 (FC)/6-311+G(3df,2p) scf=tight\\ROMP2(FC)/6-311+G(3df,2p) RadKet2dU_040\\0,2\O,0,-2.238057, 2.305596,0.16735\C,0,-2.841011,1.651976,-0.938862\H,0,-2.45567,2.13828,-1.839259\H,0,-3.934766, 1.765269,-0.927431\C,0,-2.499204,0.155036,-1.028288\H,0,-3.155744,-0.294798,-1.793332\O,0,-1.127496, -0.029358,-1.41765\C,0,-0.420386,-0.95976,-0.570254\H,0,-0.207227,-1.881355,-1.120086\N,0,0.850461,-0.416573,-0.180866\C,0,0.900751,0.746929,0.711955\H,0,-0.022724,1.313545,0.572237\C,0,2.104882, 1.622662,0.373538\H,0,1.95747,2.097215,-0.605809 \C,0,3.386121,0.813805,0.304234\O,0,4.49655, 1.245809,0.542865\N,0,3.188233,-0.501125,-0.104713 \H,0,4.020397,-1.050906,-0.287638\C,0,1.99226,-1.109585,-0.522136\O,0,2.013101,-2.169028,-1.128084\C,0,-2.590631,-0.569859,0.267017\H,0,-2.746826, 2.075852,0.960799\O,0,-3.650072,-0.550126,1.074823\H,0,-3.394353,-1.095194,1.853638\C,0,-1.377758,-

1.215789,0.598205\O,0,-1.175395,-1.838815,1.653812\H,0,2.240081,2.417707,1.110672\H,0,0.949303, 0.413158,1.757886\\Version=AM64L-G03RevD.01\State=2-A\HF=-905.6041633\MP2=-908.8447738\ RMSD=8.639e-09\Thermal=0.\PG=C01 [X(C9H11N2O6)]\\@

031

1\1\GINC-NODE23\SP\ROMP2-FC\6-311+G(3df,2p)\C9H11N2O6(2)\ZIP06\07-Jul-2011\0\\#p ROMP2 (FC)/6-311+G(3df,2p) scf=tight\\ROMP2(FC)/6-311+G(3df,2p) RadKet2dU_031\\0,2\O,0,-3.874328,-1.922175,0.036233\C,0,-2.542602,-1.711881,0.470867\H,0,-1.797805,-1.960153,-0.294292\H,0,-2.385208,-2.360353,1.337675\C,0,-2.341035,-0.248153,0.919895\H,0,-3.110961,-0.020751,1.672198\O,0,-1.037686,-0.068494,1.517831\C,0,-0.263503,0.944703,0.863285\H,0,-0.079086,1.757141,1.577128\N,0,1.043615, 0.473729,0.456461\C,0,2.149018,0.537081,1.422037\H,0,2.007146,1.429562,2.036604\C,0,3.48584,0.6096 01,0.687851\H,0,3.569178,1.563945,0.151157\C,0,3.618972,-0.504584,-0.332777\O,0,4.670243,-0.978813, -0.712282\N,0,2.397537,-0.938898,-0.840295\H,0,2.433305,-1.614422,-1.596075\C,0,1.117519,-0.424134,-0.587377\O,0,0.167859,-0.769509,-1.27528\C,0,-2.352759,0.733758,-0.192467\H,0,-3.992531,-1.442398,-0.799128\O,0,-3.3536,0.879896,-1.062104\H,0,-3.04363,1.551866,-1.709694 \C,0,-1.140806,1.452974,-0.288761\O,0,-0.888085,2.335104,-1.121541\H,0,4.327956,0.541675,1.380857\H,0,2.124479,-0.334199, 2.092237\\Version=AM64L-G03RevD.01\State=2-A\HF=-905.6051507\MP2=-908.8448253\RMSD= 7.742e-09\Thermal=0.\PG=C01 [X(C9H11N2O6)]\\@

025

1\1\GINC-NODE25\SP\ROMP2-FC\6-311+G(3df,2p)\C9H11N2O6(2)\ZIP06\07-Jul-2011\0\\#p ROMP2 (FC)/6-311+G(3df,2p) scf=tight\\ROMP2(FC)/6-311+G(3df,2p) RadKet2dU_025\\0,2\O,0,2.871162, 2.790272,0.668527\C,0,2.988855,1.685528,-0.200514\H,0,4.03624,1.627046,-0.512283\H,0,2.366543, 1.8014,-1.103118\C,0,2.587113,0.38938,0.515771\H,0,3.248327,0.258281,1.387334\O,0,1.224163, 0.541496,0.974697\C,0,0.429162,-0.642358,0.731059\H,0,0.278504,-1.189805,1.666376\N,0,-0.875687,-0.280663,0.257261\C,0,-1.037776,0.233379,-1.105602\H,0,-0.109892,0.742582,-1.37834\C,0,-2.20707, 1.212774,-1.173589\H,0,-1.963276,2.129798,-0.620713\C,0,-3.464834,0.629263,-0.556707\O,0,-4.597382, 0.927517,-0.87809\N,0,-3.209921,-0.275834,0.467814\H,0,-4.011876,-0.605627,0.993786\C,0,-1.970611,-0.638985,1.019847\O,0,-1.914335,-1.228101,2.08608\C,0,2.531258,-0.831702,-0.32875\H,0,1.963345, 2.771499,1.015369\O,0,3.539243,-1.292376,-1.061819\H,0,3.200432,-2.104274,-1.501247\C,0,1.264575,-1.458872,-0.262049\O,0,0.937418,-2.476767,-0.895219\H,0,-2.431118,1.495647,-2.204818\H,0,-1.184158,-0.597857,-1.808088\\Version=AM64L-G03RevD.01\State=2-A\HF=-905.6041869\MP2=-908.8441937\ RMSD=6.157e-09\Thermal=0.\PG=C01 [X(C9H11N2O6)]\\@

022

1\1\GINC-NODE23\SP\ROMP2-FC\6-311+G(3df,2p)\C9H11N2O6(2)\ZIP06\07-Jul-2011\0\\#p ROMP2 (FC)/6-311+G(3df,2p) scf=tight\\ROMP2(FC)/6-311+G(3df,2p) RadKet2dU_022\\0,2\O,0,-2.13975, 2.240101,0.187081\C,0,-2.847586,1.67525,-0.90654\H,0,-2.565025,2.140846,-1.861605\H,0,-3.936151, 1.77657,-0.777207\C,0,-2.513667,0.187165,-0.995279\H,0,-3.182147,-0.252555,-1.755488\O,0,-1.148084,-0.001069,-1.401391\C,0,-0.439771,-0.940602,-0.564287\H,0,-0.238299,-1.85849,-1.124397\N,0,0.840575,-0.412275,-0.183012\C,0,0.912011,0.715611,0.753024\H,0,-0.009394,1.292086,0.648464\C,0,2.118306,

1.594736,0.43119\H,0,1.962586,2.106298,-0.528275\C,0,3.39297,0.780931,0.314056\O,0,4.509744, 1.198157,0.551173\N,0,3.17926,-0.51629,-0.137732\H,0,4.00418,-1.066018,-0.350944 \C,0,1.972364,-1.101586,-0.56076\O,0,1.980758,-2.13972,-1.202947\C,0,-2.603277,-0.552145,0.294583\H,0,-2.294965, 3.196261,0.189183\O,0,-3.682436,-0.599073,1.06672\H,0,-3.425596,-1.143318,1.84461\C,0,-1.390187,-1.205516,0.607561\O,0,-1.183071,-1.863015,1.64176\H,0,2.270263,2.360371,1.195923\H,0,0.971439, 0.341787,1.784482\\Version=AM64L-G03RevD.01\State=2-A\HF=-905.6050505\MP2=-908.8451302\ RMSD=1.827e-09\Thermal=0.\PG=C01 [X(C9H11N2O6)]\\@

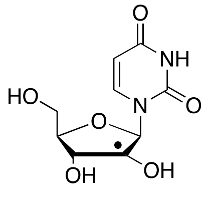

025

1\1\GINC-NODE23\SP\ROMP2-FC\6-311+G(3df,2p)\C9H11N2O6(2)\ZIP06\03-Jul-2011\0\\#p ROMP2 (FC)/6-311+G(3df,2p) scf=tight\\ROMP2(FC)/6-311+G(3df,2p) Rad2Uri_025\\0,2\O,0,1.478998, 3.101465,-0.234749\C,0,2.063416,1.953218,-0.828769\C,0,2.423557,0.865544,0.187417\O,0,1.322933, 0.627276,1.105252\C,0,1.399135,-1.150694,-0.365092\O,0,1.274066,-2.448102,-0.713936\C,0,0.611734,-0.520407,0.748392\N,0,-0.805077,-0.146733,0.356519\C,0,-1.664187,-1.204162,0.124762\O,0,-1.284466,-2.382858,0.124267\N,0,-2.97099,-0.854272,-0.098112\C,0,-3.527613,0.451866,-0.144659\O,0,-4.716662, 0.598561,-0.369278\C,0,-2.536202,1.486103,0.092347\C,0,-1.245531,1.16055,0.338053\H,0,0.358206,-2.71995,-0.453137\H,0,-3.609089,-1.622444,-0.275856\H,0,1.317971,1.575362,-1.536456\H,0,2.967529, 2.20845,-1.404927\H,0,3.264258,1.184682,0.812446\H,0,0.496811,-1.172738,1.625072\H,0,-2.859201, 2.518358,0.071537\H,0,-0.473036,1.899058,0.520553\H,0,2.091843,3.44307,0.435945\C,0,2.730188,-0.485266,-0.466572\O,0,3.746701,-1.145008,0.303929\H,0,3.707759,-2.08578,0.06465\H,0,3.069885,-0.372217,-1.507827\\Version=AM64L-G03RevD.01\State=2-A\HF=-905.585114\MP2=-908.823878\ RMSD=3.299e-09\Thermal=0.\PG=C01 [X(C9H11N2O6)]\\@

006

1\1\GINC-NODE23\SP\ROMP2-FC\6-311+G(3df,2p)\C9H11N2O6(2)\ZIP06\02-Jul-2011\0\\#p ROMP2 (FC)/6-311+G(3df,2p) scf=tight\\ROMP2(FC)/6-311+G(3df,2p) Rad2Uri_006\\0,2\O,0,-1.279837,2.0781, 1.234506\C,0,-2.307953,2.233634,0.265808\C,0,-2.468426,0.924916,-0.48904\O,0,-1.269788,0.645808,-1.269736\C,0,-1.41362,-1.002877,0.344618\O,0,-1.326586,-2.271477,0.801008\C,0,-0.601038,-0.490064,-0.804572\N,0,0.823874,-0.126819,-0.426236\C,0,1.647895,-1.183148,-0.08741\O,0,1.240687,-2.349368,-0.007624\N,0,2.957425,-0.847704,0.14753\C,0,3.546918,0.443216,0.112034\O,0,4.732764,0.579156, 0.361827\C,0,2.591075,1.478197,-0.236619\C,0,1.29941,1.161763,-0.489525\H,0,-0.416769,-2.588333, 0.576896\H,0,3.568725,-1.615408,0.403704\H,0,-3.274865,2.480102,0.732684\H,0,-2.067704,3.031306,-0.454071\H,0,-3.28976,1.028973,-1.204942\H,0,-0.489368,-1.220639,-1.618906\H,0,2.942115,2.499908,-0.29268\H,0,0.551775,1.893422,-0.75802\H,0,-1.202387,2.903684,1.735659\C,0,-2.726937,-0.307036, 0.391775\O,0,-3.780824,-1.075445,-0.216505\H,0,-3.707725,-1.973484,0.1471\H,0,-3.018401,-0.023676,

1.413292\\Version=AM64L-G03RevD.01\State=2-A\HF=-905.5858681\MP2=-908.8234801\RMSD=2.073e-09\Thermal=0.\PG=C01 [X(C9H11N2O6)]\\@

017

1\1\GINC-NODE10\SP\ROMP2-FC\6-311+G(3df,2p)\C9H11N2O6(2)\ZIP06\02-Jul-2011\0\\#p ROMP2(FC)/6-311+G(3df,2p) scf=tight\\ROMP2(FC)/6-311+G(3df,2p) Rad2Uri_017\\0,2\O,0,1.524007,3.085727,-0.148082\C,0,2.001503,1.941789,-0.842357\C,0,2.404379,0.890654,0.186326\O,0,1.321404,0.625104,1.118631\C,0,1.416639,-1.14779,-0.355714\O,0,1.312734,-2.447843,-0.702732\C,0,0.622527,-0.526839,0.758834\N,0,-0.798647,-0.159325,0.365241\C,0,-1.648593,-1.215662,0.097705\O,0,-1.263991,-2.391939,0.071087\N,0,-2.955503,-0.867136,-0.132033\C,0,-3.522527,0.43464,-0.137309\O,0,-4.709915,0.581201,-0.371778\C,0,-2.543333,1.46712,0.152705\C,0,-1.252164,1.142333,0.398462\H,0,0.39474,-2.727254,-0.459671\H,0,-3.586675,-1.634397,-0.33642\H,0,1.232988,1.526963,-1.514602\H,0,2.887069,2.179562,-1.452699\H,0,3.234608,1.257035,0.794017\H,0,0.51033,-1.181715,1.633936\H,0,-2.878366,2.495526,0.177242\H,0,-0.487057,1.876691,0.624419\H,0,1.3046,3.763497,-0.805285\C,0,2.736472,-0.459496,-0.457487\O,0,3.761335,-1.095062,0.321004\H,0,3.736023,-2.03912,0.093492\H,0,3.076783,-0.349567,-1.498909\\Version=AM64L-G03RevD.01\State=2-A\HF=-905.5848883\MP2=-908.8237596\RMSD=3.375e-09\Thermal=0.\PG=C01 [X(C9H11N2O6)]\\@

014

1\1\GINC-NODE11\SP\ROMP2-FC\6-311+G(3df,2p)\C9H11N2O6(2)\ZIP06\02-Jul-2011\0\\#p ROMP2(FC)/6-311+G(3df,2p) scf=tight\\ROMP2(FC)/6-311+G(3df,2p) Rad2Uri_014\\0,2\O,0,1.805872,2.417917,-0.699503\C,0,2.816684,1.970853,0.197659\C,0,2.520498,0.538412,0.595697\O,0,1.265969,0.488725,1.321872\C,0,1.308959,-1.339903,-0.09132\O,0,0.839431,-2.370601,-0.818751\C,0,0.498641,-0.616814,0.936426\N,0,-0.849949,-0.129869,0.417415\C,0,-1.823652,-1.100913,0.274638\O,0,-1.579466,-2.306219,0.421223\N,0,-3.073256,-0.631313,-0.037889\C,0,-3.459478,0.712461,-0.288535\O,0,-4.616335,0.969804,-0.57491\C,0,-2.348736,1.640089,-0.171041\C,0,-1.115588,1.200198,0.173228\H,0,-0.019168,-2.6519,-0.417386\H,0,-3.79454,-1.335765,-0.149118\H,0,3.811073,1.982906,-0.273497\H,0,2.853299,2.599685,1.099567\H,0,3.31845,0.201804,1.272002\H,0,0.237907,-1.227543,1.809515\H,0,-2.53725,2.687492,-0.365703\H,0,-0.256351,1.852643,0.262592\H,0,2.058273,3.291649,-1.032978\C,0,2.420657,-0.472504,-0.572762\O,0,3.655038,-1.132355,-0.836387\H,0,3.742171,-1.84658,-0.182669\H,0,2.176592,0.044428,-1.511289\\Version=AM64L-G03RevD.01\State=2-A\HF=-905.5830933\MP2=-908.8218777\RMSD=1.571e-09\Thermal=0.\PG=C01 [X(C9H11N2O6)]\\@

010

1\1\GINC-NODE9\SP\ROMP2-FC\6-311+G(3df,2p)\C9H11N2O6(2)\ZIP06\02-Jul-2011\0\\#p ROMP2(FC)/6-311+G(3df,2p) scf=tight\\ROMP2(FC)/6-311+G(3df,2p) Rad2Uri_010\\0,2\O,0,1.87114,3.121195,-0.03723\C,0,2.232003,1.954595,-0.762513\C,0,2.49767,0.783289,0.174951\O,0,1.332379,0.637376,1.05564\C,0,1.33358,-1.165827,-0.390247\O,0,1.14986,-2.461399,-0.722149\C,0,0.582301,-0.497063,0.723054\N,0,-0.832604,-0.105419,0.339201\C,0,-1.720769,-1.158108,0.200003\O,0,-1.363035,-2.340054,0.275313\N,0,-3.024281,-0.796233,-0.022814\C,0,-3.550832,0.513163,-0.17984\O,0,-4.73956,0.671991,-0.392587\C,0,-2.527952,1.5392,-0.066049\C,0,-1.243207,1.200853,0.188031\H,0,0.246358,-2.712746,-

0.4077\H,0,-3.681643,-1.561772,-0.12794\H,0,1.443571,1.678102,-1.481792\H,0,3.137883,2.187285,-1.333845\H,0,3.340678,1.00399,0.834694\H,0,0.457366,-1.132539,1.610307\H,0,-2.823501,2.572692,-0.187155\H,0,-0.457338,1.938027,0.277431\H,0,1.441824,2.822676,0.780916\C,0,2.694248,-0.568743,-0.512337\O,0,3.687062,-1.304771,0.218204\H,0,3.59343,-2.234483,-0.048019\H,0,3.014541,-0.452523,-1.559458\\Version=AM64L-G03RevD.01\State=2-A\HF=-905.5825299\MP2=-908.8220833\RMSD=2.476e-09\Thermal=0.\PG=C01 [X(C9H11N2O6)]\\@

005

1\1\GINC-NODE17\SP\ROMP2-FC\6-311+G(3df,2p)\C9H11N2O6(2)\ZIP06\02-Jul-2011\0\\#p ROMP2 (FC)/6-311+G(3df,2p) scf=tight\\ROMP2(FC)/6-311+G(3df,2p) Rad2Uri_005\\0,2\O,0,1.607728,-2.089323,1.491564\C,0,2.430633,-2.200478,0.342147\C,0,2.494865,-0.926119,-0.491446\O,0,1.219634,-0.734607,-1.188604\C,0,1.406294,0.995094,0.33181\O,0,1.325158,2.275597,0.750304\C,0,0.576529,0.443827,-0.794309\N,0,-0.854336,0.102176,-0.411435\C,0,-1.668602,1.15425,-0.026473\O,0,-1.25777,2.313841,0.088233\N,0,-2.977713,0.819077,0.214881\C,0,-3.591492,-0.454946,0.102851\O,0,-4.773685,-0.590959,0.362451\C,0,-2.661293,-1.478621,-0.344157\C,0,-1.369154,-1.164767,-0.592268\H,0,0.400691,2.576612,0.569778\H,0,-3.576053,1.582323,0.512821\H,0,3.436321,-2.426363,0.712316\H,0,2.119022,-3.035417,-0.304915\H,0,3.259276,-1.031261,-1.267891\H,0,0.483262,1.13682,-1.644573\H,0,-3.035396,-2.48324,-0.489509\H,0,-0.649494,-1.883029,-0.962601\H,0,0.697235,-1.935221,1.195615\C,0,2.752951,0.353563,0.310112\O,0,3.71348,1.143807,-0.41071\H,0,3.662979,2.040515,-0.039862\H,0,3.125754,0.131827,1.319196\\Version=AM64L-G03RevD.01\State=2-A\HF=-905.5833756\MP2=-908.8218906\RMSD=4.376e-09\Thermal=0.\PG=C01 [X(C9H11N2O6)]\\@

020

1\1\GINC-NODE17\SP\ROMP2-FC\6-311+G(3df,2p)\C9H11N2O6(2)\ZIP06\03-Jul-2011\0\\#p ROMP2 (FC)/6-311+G(3df,2p) scf=tight\\ROMP2(FC)/6-311+G(3df,2p) Rad2Uri_020\\0,2\O,0,-3.438729,-2.241953,-1.136308\C,0,-2.237713,-1.965239,-0.435233\C,0,-2.379049,-0.743957,0.479062\O,0,-1.154017,-0.565929,1.250364\C,0,-1.228678,1.117976,-0.335059\O,0,-1.057196,2.377194,-0.789296\C,0,-0.417682,0.532563,0.788957\N,0,0.969179,0.068439,0.377956\C,0,1.855857,1.052207,-0.02337\O,0,1.531347,2.239931,-0.138697\N,0,3.131918,0.61755,-0.279327\C,0,3.643815,-0.70207,-0.177028\O,0,4.809477,-0.928677,-0.450081\C,0,2.639981,-1.650519,0.273327\C,0,1.379427,-1.237295,0.538333\H,0,-0.111746,2.613931,-0.619422\H,0,3.784187,1.330985,-0.586928\H,0,-1.898951,-2.82574,0.161885\H,0,-1.479677,-1.769071,-1.200878\H,0,-3.180539,-0.903011,1.208314\H,0,-0.244116,1.239498,1.613366\H,0,2.932826,-2.683981,0.401773\H,0,0.605647,-1.899982,0.901278\H,0,-4.108178,-2.520565,-0.491586\C,0,-2.612498,0.564207,-0.273088\O,0,-3.490641,1.38678,0.514902\H,0,-3.414441,2.288045,0.16041\H,0,-3.05539,0.381951,-1.261094\\Version=AM64L-G03RevD.01\State=2-A\HF=-905.5844946\MP2=-908.8216169\RMSD=2.857e-09\Thermal=0.\PG=C01 [X(C9H11N2O6)]\\@

018

1\1\GINC-NODE14\SP\ROMP2-FC\6-311+G(3df,2p)\C9H11N2O6(2)\ZIP06\03-Jul-2011\0\\#p ROMP2 (FC)/6-311+G(3df,2p) scf=tight\\ROMP2(FC)/6-311+G(3df,2p) Rad2Uri_018\\0,2\O,0,-3.443297,-2.227503,-1.040688\C,0,-2.185561,-1.963341,-0.439273\C,0,-2.375076,-0.761243,0.482702\O,0,-1.160031,

-0.557208,1.262648\C,0,-1.247171,1.107967,-0.342307\O,0,-1.087281,2.366285,-0.804855\C,0,-0.431269, 0.540173,0.788505\N,0,0.959222,0.079357,0.381769\C,0,1.838377,1.059753,-0.043605\O,0,1.50812, 2.243214,-0.18169\N,0,3.115859,0.626665,-0.297673\C,0,3.636778,-0.686657,-0.1679\O,0,4.801947,- 0.912846,-0.444132\C,0,2.642329,-1.629899,0.312784\C,0,1.380341,-1.218302,0.574333\H,0,-0.14191, 2.608818,-0.644485\H,0,3.762318,1.337474,-0.623065\H,0,-1.831523,-2.815058,0.162048\H,0,-1.412857,- 1.730883,-1.189924\H,0,-3.179651,-0.961248,1.193968\H,0,-0.261892,1.257115,1.604947\H,0,2.943647,- 2.65725,0.468072\H,0,0.613426,-1.876003,0.96046\H,0,-3.333585,-2.957581,-1.66779\C,0,-2.628942, 0.54906,-0.25896\O,0,-3.488317,1.366407,0.552316\H,0,-3.421371,2.269092,0.199811\H,0,-3.090146, 0.371519,-1.239194\\Version=AM64L-G03RevD.01\State=2-A\HF=-905.5847246\MP2=-908.8216348\ RMSD=2.905e-09\Thermal=0.\PG=C01 [X(C9H11N2O6)]\\@

004

1\1\GINC-NODE25\SP\ROMP2-FC\6-311+G(3df,2p)\C9H11N2O6(2)\ZIP06\02-Jul-2011\0\\#p ROMP2 (FC)/6-311+G(3df,2p) scf=tight\\ROMP2(FC)/6-311+G(3df,2p) Rad3Uri_004\\0,2\O,0,2.081264, 2.534533,-0.288824\C,0,2.675618,1.870251,0.804749\C,0,2.372512,0.374407,0.849744\O,0,1.016812, 0.211328,1.335168\C,0,1.229616,-1.232734,-0.46707\O,0,1.321817,-2.528637,-0.853606\C,0,0.375771,- 0.878345,0.706465\N,0,-1.023617,-0.491844,0.397951\C,0,-1.253801,0.572247,-0.477129\O,0,-0.359126, 1.216608,-1.012274\N,0,-2.587039,0.841616,-0.702735\C,0,-3.724778,0.199911,-0.159806\O,0,-4.845601, 0.567589,-0.466795\C,0,-3.372872,-0.876221,0.752981\C,0,-2.074733,-1.163068,0.988753\H,0,2.242554,- 2.638904,-1.172453\H,0,-2.767667,1.611326,-1.338257\H,0,3.759613,2.007265,0.705927\H,0,2.374649, 2.307752,1.770378\H,0,3.06125,-0.132701,1.540542\H,0,0.277673,-1.737093,1.388696\H,0,-4.170935,- 1.426989,1.232217\H,0,-1.777276,-1.957285,1.665194\H,0,1.14895,2.249772,-0.352861\C,0,2.43966,- 0.35032,-0.515351\O,0,3.598555,-1.185637,-0.665831\H,0,4.219394,-0.739214,-1.259889\H,0,2.394662, 0.38464,-1.328712\\Version=AM64L-G03RevD.01\State=2-A\HF=-905.5825082\MP2=-908.819764\ RMSD=2.868e-09\Thermal=0.\PG=C01 [X(C9H11N2O6)]\\@

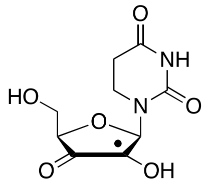

004

1\1\GINC-NODE20\SP\ROMP2-FC\6-311+G(3df,2p)\C9H11N2O6(2)\ZIP06\23-Jan-2012\0\\#p ROMP2 (FC)/6-311+G(3df,2p) scf=tight\\RadKet3dU_004\\0,2\O,0,-1.782781,-2.151486,-0.928858\C,0,-2.735654,- 1.913757,0.098916\C,0,-2.602528,-0.529717,0.728356\O,0,-1.36037,-0.389424,1.458359\C,0,-0.5837, 0.676042,0.975965\N,0,0.72371,0.164347,0.436968\C,0,1.667238,1.11189,0.215035\O,0,1.425306,2.33134 2,0.217855\N,0,2.966416,0.667767,-0.015812\C,0,3.365608,-0.635441,-0.324314\O,0,4.523412,-0.89672,- 0.573057\C,0,2.211337,-1.615943,-0.367341\C,0,1.090744,-1.250073,0.604304\H,0,3.656431,1.402031,- 0.132529\H,0,-3.764595,-2.015095,-0.27376\H,0,-2.567633,-2.680641,0.860796\H,0,-3.424274,-0.387111, 1.443868\H,0,1.823402,-1.621292,-1.395098\H,0,1.399562,-1.437902,1.642146\H,0,-2.048625,-1.617221,- 1.69508\C,0,-1.412091,1.337174,-0.090429\H,0,2.598748,-2.615554,-0.157759\H,0,0.207233,-1.84837, 0.391466\O,0,-1.042343,2.418764,-0.751053\H,0,-0.121154,2.653352,-0.444997\C,0,-2.604138,0.5911,-

0.31476\O,0,-3.467241,0.732198,-1.191277\H,0,-0.338738,1.357955,1.802719\\Version=AM64L-G03RevD.01\State=2-A\HF=-905.6046285\MP2=-908.848015\RMSD=2.310e-09\Thermal=0.\PG=C01 [X(C9H11N2O6)]\\@

008

1\1\GINC-NODE23\SP\ROMP2-FC\6-311+G(3df,2p)\C9H11N2O6(2)\ZIP06\23-Jan-2012\0\\#p ROMP2(FC)/6-311+G(3df,2p) scf=tight\\RadKet3dU_008\\0,2\O,0,-2.027162,-2.033635,-1.121415\C,0,-2.802521,-1.90517,0.06183\C,0,-2.576734,-0.577068,0.781799\O,0,-1.272306,-0.51829,1.410252\C,0,-0.529581,0.592742,0.975502\N,0,0.769192,0.141395,0.384175\C,0,1.682667,1.108651,0.129602\O,0,1.451011,2.324567,0.243489\N,0,2.941313,0.678302,-0.280613\C,0,3.445114,-0.625007,-0.238619\O,0,4.590366,-0.864877,-0.557129\C,0,2.440652,-1.645222,0.263481\C,0,1.001647,-1.240977,-0.057365\H,0,3.590392,1.416451,-0.530453\H,0,-3.877512,-1.993492,-0.14929\H,0,-2.514205,-2.730824,0.719569\H,0,-3.329846,-0.467041,1.574461\H,0,2.688106,-2.612664,-0.180208\H,0,0.289198,-1.876937,0.462102\H,0,-2.42907,-1.449328,-1.785213\C,0,-1.413071,1.319654,-0.001091\H,0,2.576649,-1.734549,1.349903\H,0,0.797702,-1.332467,-1.131073\O,0,-1.069139,2.440088,-0.610378\H,0,-0.135668,2.658829,-0.330985\C,0,-2.633424,0.610283,-0.183038\O,0,-3.555178,0.818592,-0.983395\H,0,-0.258515,1.225271,1.83225\\Version=AM64L-G03RevD.01\State=2-A\HF=-905.604426\MP2=-908.8474979\RMSD=4.828e-09\Thermal=0.\PG=C01 [X(C9H11N2O6)]\\@

007

1\1\GINC-NODE15\SP\ROMP2-FC\6-311+G(3df,2p)\C9H11N2O6(2)\ZIP06\23-Jan-2012\0\\#p ROMP2(FC)/6-311+G(3df,2p) scf=tight\\RadKet3dU_007\\0,2\O,0,-4.208018,-1.654846,-0.540462\C,0,-2.862312,-1.754544,-0.132929\C,0,-2.479148,-0.515588,0.688575\O,0,-1.146878,-0.622446,1.247152\C,0,-0.358494,0.500159,0.931454\N,0,0.928749,0.051355,0.315718\C,0,1.888605,0.996912,0.160752\O,0,1.701845,2.206849,0.368056\N,0,3.137512,0.5461,-0.255559\C,0,3.587221,-0.777221,-0.303242\O,0,4.729322,-1.041197,-0.611418\C,0,2.529002,-1.791116,0.091568\C,0,1.118117,-1.297164,-0.231065\H,0,3.823042,1.272814,-0.431358\H,0,-2.767045,-2.655105,0.48277\H,0,-2.169524,-1.852357,-0.986858\H,0,-3.193701,-0.413299,1.515001\H,0,2.749108,-2.730017,-0.422256\H,0,0.366708,-1.942707,0.219928\H,0,-4.305979,-0.778233,-0.961882\C,0,-1.203884,1.351746,0.020923\H,0,2.628321,-1.971804,1.170318\H,0,0.954253,-1.288729,-1.317709\O,0,-0.802424,2.497202,-0.498603\H,0,0.138261,2.649742,-0.201401\C,0,-2.475024,0.741832,-0.17515\O,0,-3.408419,1.084206,-0.919696\H,0,-0.075214,1.039267,1.846775\\Version=AM64L-G03RevD.01\State=2-A\HF=-905.6022208\MP2=-908.8445872\RMSD=6.706e-09\Thermal=0.\PG=C01 [X(C9H11N2O6)]\\@

003

1\1\GINC-NODE12\SP\ROMP2-FC\6-311+G(3df,2p)\C9H11N2O6(2)\ZIP06\23-Jan-2012\0\\#p ROMP2(FC)/6-311+G(3df,2p) scf=tight\\RadKet3dU_003\\0,2\O,0,-4.108054,-1.712927,-0.639541\C,0,-2.788443,-1.76546,-0.147072\C,0,-2.490541,-0.501092,0.67164\O,0,-1.19171,-0.55445,1.307121\C,0,-0.385453,0.54562,0.961609\N,0,0.90219,0.046569,0.36212\C,0,1.867216,0.988961,0.182797\O,0,1.658189,2.210586,0.248547\N,0,3.154069,0.530404,-0.080446\C,0,3.536768,-0.770078,-0.422537\O,0,4.683562,-1.032676,-0.71333\C,0,2.379459,-1.748228,-0.427845\C,0,1.297865,-1.35578,0.575741\H,0,3.852732,1.258342,-

0.18737\H,0,-2.707258,-2.65148,0.491388\H,0,-2.039992,-1.858067,-0.953698\H,0,-3.252399,-0.405916,
1.455113\H,0,1.964265,-1.764131,-1.444617\H,0,1.659582,-1.493614,1.605219\H,0,-4.208088,-0.844115,-
1.076386\C,0,-1.210782,1.366457,0.002314\H,0,2.770561,-2.745819,-0.214624\H,0,0.412138,-1.975966,
0.454694\O,0,-0.823674,2.499921,-0.548468\H,0,0.125834,2.653523,-0.278351\C,0,-2.469745,0.736315,-
0.214952\O,0,-3.377085,1.051771,-1.00204\H,0,-0.119065,1.110337,1.868438\\Version=AM64L-G03Rev
D.01\State=2-A\HF=-905.6020679\MP2=-908.8443338\RMSD=3.219e-09\Thermal=0.\PG=C01 [X(C9H11
N2O6)]\\@

014

1\1\GINC-NODE10\SP\ROMP2-FC\6-311+G(3df,2p)\C9H11N2O6(2)\ZIP06\23-Jan-2012\0\\#p ROMP2
(FC)/6-311+G(3df,2p) scf=tight\\RadKet3dU_014\\0,2\O,0,-2.265449,2.27832,0.288339\C,0,-
2.824266,1.697812,-0.883384\C,0,-2.495609,0.209702,-1.032005\O,0,-1.125617,0.014265,-1.429582\C,0,-
0.435305,-0.950863,-0.614594\N,0,0.831522,-0.421629,-0.17135\C,0,1.980526,-1.104574,-0.5094\O,0,
2.009354,-2.167775,-1.109226\N,0,3.16824,-0.482716,-0.092609\C,0,3.35165,0.841883,0.297533\O,0,
4.457743,1.287099,0.529256\C,0,2.061789,1.637729,0.3581\C,0,0.868297,0.752197,0.709135\H,0,4.00722
8,-1.023279,-0.272446\H,0,-3.916805,1.815961,-0.902947\H,0,-2.403901,2.240118,-1.734691\H,0,-
3.155163,-0.225197,-1.799012\H,0,2.188671,2.443315,1.085162\H,0,-0.063579,1.305506,0.571674\H,0,-
2.79012,1.958327,1.040534\C,0,-1.413141,-1.228758,0.482039\H,0,1.906833,2.098353,-0.626638\H,0,
0.92897,0.429994,1.758551\O,0,-1.151938,-1.97994,1.544397\H,0,-1.965397,-1.95861,2.095939\C,0,-
2.634846,-0.542682,0.284079\O,0,-3.58587,-0.52993,1.087336\H,0,-0.19543,-1.845052,-1.201598\\Version=
AM64L-G03RevD.01\State=2-A\HF=-905.6070849\MP2=-908.8469492\RMSD=8.225e-09\Thermal=
0.\PG=C01 [X(C9H11N2O6)]\\@

019

1\1\GINC-NODE27\SP\ROMP2-FC\6-311+G(3df,2p)\C9H11N2O6(2)\ZIP06\23-Jan-2012\0\\#p ROMP2
(FC)/6-311+G(3df,2p) scf=tight\\RadKet3dU_019\\0,2\O,0,-1.586294,-2.097143,-0.891025\C,0,-2.690163,-
1.951516,-0.001903\C,0,-2.600433,-0.594909,0.675984\O,0,-1.369818,-0.444333,1.420119\C,0,-0.608679,
0.644245,0.959146\N,0,0.717577,0.16395,0.43838\C,0,1.637158,1.131917,0.205685\O,0,1.373849,2.34625
8,0.19828\N,0,2.94649,0.713391,-0.026173\C,0,3.373705,-0.58188,-0.326691\O,0,4.53309,-0.817077,-
0.595572\C,0,2.246092,-1.5937,-0.324893\C,0,1.139127,-1.227293,0.660893\H,0,3.616829,1.462698,-
0.160135\H,0,-3.648892,-1.971084,-0.538786\H,0,-2.694589,-2.748807,0.756374\H,0,-3.431446,-0.51392,
1.38958\H,0,1.834119,-1.632582,-1.342461\H,0,1.485097,-1.354259,1.696921\H,0,-1.802676,-2.792495,-
1.529162\C,0,-1.446936,1.312359,-0.09609\H,0,2.665605,-2.576803,-0.098667\H,0,0.272484,-
1.864448,0.50232\O,0,-1.108443,2.427606,-0.718674\H,0,-0.184724,2.662468,-0.425092\C,0,-
2.646798,0.576075,-0.312009\O,0,-3.565979,0.782036,-1.110431\H,0,-0.389951,1.317278,1.801354
\\Version=AM64L-G03RevD.01\State=2-A\HF=-905.6031292\MP2=-908.845734\RMSD=4.053e-
09\Thermal=0.\PG=C01 [X(C9H11N2O6)]\\@

017

1\1\GINC-NODE17\SP\ROMP2-FC\6-311+G(3df,2p)\C9H11N2O6(2)\ZIP06\23-Jan-2012\0\\#p ROMP2
(FC)/6-311+G(3df,2p) scf=tight\\RadKet3dU_017\\0,2\O,0,-2.35045,2.248426,-0.088419\C,0,-2.890466,

1.452481,-1.13558\C,0,-2.54324,-0.034894,-1.003078\O,0,-1.189538,-0.302319,-1.41895\C,0,-0.436722,-1.043331,-0.441565\N,0,0.834922,-0.409011,-0.178504\C,0,1.975234,-1.057051,-0.616057\O,0,1.999324,-2.171721,-1.11231\N,0,3.161805,-0.332164,-0.425804\C,0,3.362998,0.809797,0.346514\O,0,4.473754,1.270752,0.516693\C,0,2.087221,1.368766,0.949107\C,0,0.873438,1.036203,0.087006\H,0,3.994261,-0.799379,-0.768347\H,0,-3.984034,1.550985,-1.185265\H,0,-2.468806,1.830595,-2.070951\H,0,-3.226932,-0.620125,-1.636565\H,0,1.974089,0.933462,1.951209\H,0,0.910463,1.588514,-0.860704\H,0,-2.886202,2.072513,0.702332\C,0,-1.358552,-1.090297,0.733875\H,0,2.210553,2.448077,1.067647\H,0,-0.057449,1.325826,0.577345\O,0,-1.039059,-1.597511,1.917474\H,0,-1.837031,-1.486157,2.48078\C,0,-2.612645,-0.503289,0.443372\O,0,-3.538081,-0.353204,1.26268\H,0,-0.197191,-2.041778,-0.826229\\Version=AM64L-G03RevD.01\State=2-A\HF=-905.6067022\MP2=-908.8460953\RMSD=6.418e-09\Thermal=0.\PG=C01 [X(C9H11N2O6)]\\@

034

1\1\GINC-NODE19\SP\ROMP2-FC\6-311+G(3df,2p)\C9H11N2O6(2)\ZIP06\23-Jan-2012\0\\#p ROMP2(FC)/6-311+G(3df,2p) scf=tight\\RadKet3dU_034\\0,2\O,0,-3.910386,-1.881475,-0.102551\C,0,-2.567979,-1.767264,0.329672\C,0,-2.332998,-0.364566,0.913954\O,0,-1.012855,-0.225098,1.483975\C,0,-0.283526,0.872006,0.920918\N,0,1.039963,0.478787,0.46477\C,0,1.12637,-0.298929,-0.674969\O,0,0.193199,-0.527096,-1.429552\N,0,2.399605,-0.81711,-0.945788\C,0,3.61151,-0.500186,-0.336513\O,0,4.661595,-0.960704,-0.734302\C,0,3.471923,0.475924,0.816283\C,0,2.100275,0.368885,1.47724\H,0,2.446214,-1.396882,-1.776996\H,0,-2.411734,-2.519725,1.108856\H,0,-1.843729,-1.946382,-0.477421\H,0,-3.080081,-0.184474,1.698345\H,0,4.275778,0.27927,1.529818\H,0,1.962811,1.180498,2.196791\H,0,-4.067384,-1.155665,-0.733989\C,0,-1.19054,1.405044,-0.137645\H,0,3.620948,1.487301,0.41545\H,0,2.008477,-0.578904,2.026155\O,0,-0.885971,2.412445,-0.946166\H,0,-1.650079,2.503895,-1.55647\C,0,-2.415953,0.698349,-0.164634\O,0,-3.339151,0.900178,-0.97629\H,0,-0.104136,1.617748,1.709835\\Version=AM64L-G03RevD.01\State=2-A\HF=-905.6065649\MP2=-908.8454791\RMSD=5.825e-09\Thermal=0.\PG=C01 [X(C9H11N2O6)]\\@

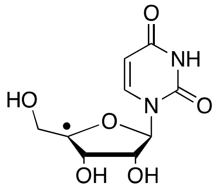

025

1\1\GINC-NODE15\SP\ROMP2-FC\6-311+G(3df,2p)\C9H11N2O6(2)\ZIP06\20-Dec-2011\0\\#p ROMP2(FC)/6-311+G(3df,2p) scf=tight\\Rad4Uri_025\\0,2\O,0,3.993842,-1.46874,-1.054374\C,0,3.417181,-1.759179,0.221276\C,0,2.279625,-0.851376,0.545007\O,0,1.120017,-1.055478,-0.201698\C,0,1.000382,1.12934,0.722022\O,0,1.027024,2.471147,0.308362\C,0,0.395931,0.151778,-0.321681\N,0,-1.043545,-0.143049,-0.128885\C,0,-1.915294,0.916646,-0.350338\O,0,-1.519621,2.053731,-0.614634\N,0,-3.246142,0.599763,-0.249429\C,0,-3.826628,-0.656253,0.066683\O,0,-5.037124,-0.771146,0.13131\C,0,-2.828515,-1.691007,0.28269\C,0,-1.511798,-1.408517,0.173165\H,0,0.142115,2.661259,-0.070129\H,0,-3.891174,1.367729,-0.40237\H,0,3.082168,-2.800393,0.174915\H,0,4.162393,-1.680796,1.028534\H,0,0.522073,

0.560201,-1.330654\H,0,-3.170084,-2.688731,0.523461\H,0,-0.735929,-2.149431,0.308022\H,0,4.116354,-0.502567,-1.08192\C,0,2.444198,0.605786,0.841986\O,0,3.271106,1.21122,-0.165173\H,0,2.879957,2.092267,-0.329174\H,0,0.472871,0.998607,1.678597\H,0,2.874636,0.795666,1.833718\\Version=AM64L-G03RevD.01\State=2-A\HF=-905.587357\MP2=-908.8236613\RMSD=4.050e-09\Thermal=0.\PG=C01 [X(C9H11N2O6)]\\@

008

1\1\GINC-NODE24\SP\ROMP2-FC\6-311+G(3df,2p)\C9H11N2O6(2)\ZIP06\19-Dec-2011\0\\#p ROMP2(FC)/6-311+G(3df,2p) scf=tight\\Rad4Uri_008\\0,2\O,0,4.629618,-1.20646,0.061795\C,0,3.364937,-1.81383,-0.133613\C,0,2.243646,-0.890086,0.252132\O,0,1.064948,-1.0225,-0.486093\C,0,0.995541,1.062196,0.657753\O,0,0.998889,2.437973,0.374739\C,0,0.331587,0.186684,-0.439982\N,0,-1.093792,-0.136549,-0.190048\C,0,-1.979365,0.932432,-0.261756\O,0,-1.602231,2.091242,-0.445477\N,0,-3.301298,0.597331,-0.110818\C,0,-3.858553,-0.685967,0.127407\O,0,-5.062905,-0.815922,0.252123\C,0,-2.846547,-1.727638,0.194028\C,0,-1.539513,-1.426147,0.030734\H,0,0.093658,2.660301,0.070597\H,0,-3.956154,1.370888,-0.155164\H,0,3.205066,-2.136321,-1.178511\H,0,3.374011,-2.725991,0.477832\H,0,0.393992,0.693949,-1.4087\H,0,-3.170318,-2.744799,0.369123\H,0,-0.754704,-2.169521,0.055604\H,0,4.604085,-0.342489,-0.387658\C,0,2.440938,0.534913,0.645747\O,0,3.189795,1.205948,-0.383407\H,0,2.85072,2.122168,-0.400298\H,0,0.522273,0.837609,1.625375\H,0,2.942073,0.651147,1.613729\\Version=AM64L-G03RevD.01\State=2-A\HF=-905.5849064\MP2=-908.8207562\RMSD=4.730e-09\Thermal=0.\PG=C01 [X(C9H11N2O6)]\\@

036

1\1\GINC-NODE17\SP\ROMP2-FC\6-311+G(3df,2p)\C9H11N2O6(2)\ZIP06\20-Dec-2011\0\\#p ROMP2(FC)/6-311+G(3df,2p) scf=tight\\Rad4Uri_036\\0,2\O,0,-4.057457,-1.15067,1.199118\C,0,-3.338131,-1.79424,0.106833\C,0,-2.248686,-0.925853,-0.407851\O,0,-1.062662,-0.973849,0.301901\C,0,-1.045018,0.997732,-1.046052\O,0,-0.95796,2.391902,-0.980903\C,0,-0.367806,0.285132,0.155797\N,0,1.051825,0.034176,0.03298\C,0,1.903451,0.56322,1.023936\O,0,1.501436,1.219115,1.968093\N,0,3.238034,0.272361,0.817233\C,0,3.82826,-0.50476,-0.203459\O,0,5.034946,-0.674311,-0.232587\C,0,2.851013,-1.042176,-1.141229\C,0,1.541089,-0.761981,-0.985714\H,0,-1.665266,2.668528,-0.367931\H,0,3.871913,0.655619,1.509853\H,0,-2.951076,-2.768712,0.427989\H,0,-4.094554,-1.956101,-0.665252\H,0,-0.497168,0.875043,1.063616\H,0,3.205453,-1.670716,-1.947015\H,0,0.784572,-1.164849,-1.649053\H,0,-3.456932,-1.142669,1.96329\C,0,-2.479923,0.445466,-0.95546\O,0,-3.177295,1.303125,-0.027287\H,0,-3.759499,0.730242,0.509757\H,0,-0.576084,0.682584,-1.984835\H,0,-3.003941,0.447539,-1.919397\\Version=AM64L-G03RevD.01\State=2-A\HF=-905.5841759\MP2=-908.822036\RMSD=4.141e-09\Thermal=0.\PG=C01 [X(C9H11N2O6)]\\@

048

1\1\GINC-NODE10\SP\ROMP2-FC\6-311+G(3df,2p)\C9H11N2O6(2)\ZIP06\20-Dec-2011\0\\#p ROMP2(FC)/6-311+G(3df,2p) scf=tight\\Rad4Uri_048\\0,2\O,0,4.03891,-1.095009,-1.048878\C,0,2.917101,-1.825408,-0.45684\C,0,1.840534,-0.906987,-0.01971\O,0,0.944527,-0.530992,-1.003417\C,0,1.068275,1.305007,0.531486\O,0,1.80793,2.373819,-0.015524\C,0,0.259671,0.667526,-0.621416\N,0,-1.155112,

0.390317,-0.365205\C,0,-1.51738,-0.322249,0.787939\O,0,-0.708812,-0.662618,1.636214\N,0,-2.867035,-0.595066,0.883498\C,0,-3.900439,-0.280982,-0.027961\O,0,-5.051221,-0.606361,0.205506\C,0,-3.414156,0.434292,-1.199306\C,0,-2.101917,0.72281,-1.318509\H,0,2.712024,2.246605,0.345144\H,0,-3.143912,-1.103418,1.716416\H,0,2.540617,-2.573268,-1.164174\H,0,3.35307,-2.34119,0.401762\H,0,0.285306,1.344589,-1.47595\H,0,-4.127983,0.720221,-1.959959\H,0,-1.707803,1.249649,-2.180798\H,0,3.709251,-0.700253,-1.873496\C,0,2.036308,0.166422,1.009191\O,0,3.387072,0.656445,1.073483\H,0,3.87886,0.261874,0.322449\H,0,0.412332,1.649391,1.337427\H,0,1.768018,-0.165807,2.014815\\Version=AM64L-G03RevD.01\State=2-A\HF=-905.5802325\MP2=-908.8218973\RMSD=6.622e-09\Thermal=0.\PG=C01 [X(C9H11N2O6)]\\@

015

1\1\GINC-NODE10\SP\ROMP2-FC\6-311+G(3df,2p)\C9H11N2O6(2)\ZIP06\19-Dec-2011\0\\#p ROMP2 (FC)/6-311+G(3df,2p) scf=tight\\Rad4Uri_015\\0,2\O,0,-2.245878,2.56792,0.488496\C,0,-2.702254,1.93699,-0.709068\C,0,-2.33901,0.487207,-0.731745\O,0,-1.078394,0.210471,-1.240496\C,0,-1.293146,-0.96019,0.833624\O,0,-1.262964,-2.264898,1.349193\C,0,-0.453858,-0.811324,-0.466767\N,0,0.95774,-0.477347,-0.284647\C,0,1.3008,0.727027,0.344859\O,0,0.47705,1.520744,0.782123\N,0,2.656712,0.947128,0.443504\C,0,3.723681,0.130631,-0.000808\O,0,4.878381,0.47788,0.170663\C,0,3.259784,-1.087277,-0.647508\C,0,1.938044,-1.328979,-0.764683\H,0,-1.959332,-2.75181,0.870204\H,0,2.917538,1.814601,0.900325\H,0,-3.789774,2.060621,-0.714365\H,0,-2.300339,2.42452,-1.609516\H,0,-0.47742,-1.758928,-1.013736\H,0,3.997144,-1.779581,-1.030735\H,0,1.559415,-2.22439,-1.244596\H,0,-1.285965,2.403955,0.553191\C,0,-2.679648,-0.487391,0.347117\O,0,-3.314976,-1.683562,-0.163968\H,0,-3.910424,-1.41905,-0.882316\H,0,-0.915626,-0.278857,1.597081\H,0,-3.288483,-0.048159,1.144244\\Version=AM64L-G03RevD.01\State=2-A\HF=-905.5844272\MP2=-908.8222872\RMSD=3.944e-09\Thermal=0.\PG=C01 [X(C9H11N2O6)]\\@

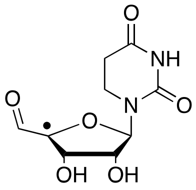

023

1\1\GINC-NODE6\SP\ROMP2-FC\6-311+G(3df,2p)\C9H11N2O6(2)\ZIP06\16-Jan-2012\0\\#p ROMP2 (FC)/6-311+G(3df,2p) scf=tight\\RadKet5dU_023\\0,2\C,0,-2.098624,-0.589396,0.554295\O,0,-0.985858,-0.260961,1.226708\C,0,-0.270482,0.819589,0.522463\N,0,1.094989,0.446773,0.273498\C,0,1.365106,-0.519963,-0.679262\O,0,0.529982,-0.999998,-1.428426\N,0,2.702542,-0.926082,-0.752294\C,0,3.818734,-0.356452,-0.141798\O,0,4.939879,-0.762349,-0.365309\C,0,3.478588,0.80386,0.773287\C,0,2.073617,0.664712,1.353138\H,0,2.880768,-1.65138,-1.439004\H,0,4.23236,0.849356,1.562977\H,0,1.791833,1.578299,1.881897\C,0,-1.173477,1.217866,-0.673551\H,0,3.555777,1.727834,0.185035\H,0,2.035257,-0.162784,2.075536\H,0,-0.586381,1.377771,-1.583324\O,0,-1.86119,2.390542,-0.29088\H,0,-2.793521,2.233162,-0.544035\C,0,-2.171904,0.040976,-0.810639\H,0,-0.252967,1.659177,1.21726\H,0,-1.817284,-0.667277,-1.567412\O,0,-3.460604,0.53647,-1.134444\H,0,-4.089552,-0.13342,-0.789103\C,0,-3.105296,-

1.402457,1.122197\O,0,-4.176241,-1.616402,0.505687\H,0,-2.924322,-1.826404,2.124286\\Version=AM64L-G03RevD.01\State=2-A\HF=-905.5999428\MP2=-908.8443837\RMSD=6.744e-09\Thermal=0.\PG=C01 [X(C9H11N2O6)]\\@

025

1\1\GINC-NODE37\SP\ROMP2-FC\6-311+G(3df,2p)\C9H11N2O6(2)\ZIP06\17-Jan-2012\0\\#p ROMP2 (FC)/6-311+G(3df,2p) scf=tight\\RadKet5dU_025\\0,2\C,0,-2.048707,-0.65731,0.536114\O,0,-0.925398,-0.32922,1.191451\C,0,-0.278095,0.818687,0.543248\N,0,1.092053,0.500826,0.236518\C,0,1.375143,-0.371131,-0.791549\O,0,0.531128,-0.873113,-1.517621\N,0,2.735859,-0.655053,-0.974147\C,0,3.781798,-0.4568,-0.07474\O,0,4.903861,-0.856216,-0.305377\C,0,3.354145,0.254764,1.194825\C,0,2.180974,1.198185,0.938776\H,0,2.934094,-1.241729,-1.777807\H,0,3.070608,-0.512421,1.927545\H,0,2.513702,2.064864,0.350496\C,0,-1.238156,1.259414,-0.595298\H,0,4.216828,0.793709,1.593623\H,0,1.787392,1.574433,1.885469\H,0,-0.686817,1.503928,-1.509368\O,0,-1.958439,2.374145,-0.112953\H,0,-2.891418,2.1972,-0.35053\C,0,-2.192107,0.05428,-0.782201\H,0,-0.264937,1.615769,1.286634\H,0,-1.832822,-0.588852,-1.593121\O,0,-3.509584,0.514123,-1.034371\H,0,-4.099534,-0.200802,-0.710999\C,0,-3.003245,-1.543526,1.083799\O,0,-4.086807,-1.760157,0.490592\H,0,-2.771068,-2.022384,2.049853\\Version=AM64L-G03RevD.01\State=2-A\HF=-905.5978935\MP2=-908.8421603\RMSD=5.718e-09\Thermal=0.\PG=C01 [X(C9H11N2O6)]\\@

008

1\1\GINC-NODE38\SP\ROMP2-FC\6-311+G(3df,2p)\C9H11N2O6(2)\ZIP06\17-Jan-2012\0\\#p ROMP2 (FC)/6-311+G(3df,2p) scf=tight\\RadKet5dU_008\\0,2\C,0,-2.13155,-0.379559,0.711751\O,0,-1.021846,0.209871,1.167935\C,0,-0.298331,0.874582,0.0523\N,0,1.019378,0.332768,-0.073059\C,0,2.034419,1.013128,0.598693\O,0,1.892593,2.08663,1.153629\N,0,3.281324,0.3748,0.582078\C,0,3.686927,-0.727296,-0.165382\O,0,4.82818,-1.137782,-0.1266\C,0,2.584653,-1.307422,-1.029691\C,0,1.207487,-1.085036,-0.408724\H,0,4.002928,0.860838,1.103912\H,0,2.638713,-0.817695,-2.010991\H,0,1.082926,-1.708987,0.48716\C,0,-1.236692,0.738667,-1.169453\H,0,2.787293,-2.370809,-1.177016\H,0,0.434283,-1.376334,-1.122769\H,0,-0.664291,0.542366,-2.086582\O,0,-1.973322,1.933342,-1.271882\H,0,-2.885407,1.658174,-1.492375\C,0,-2.186047,-0.42585,-0.793281\H,0,-0.200746,1.918231,0.341231\H,0,-1.77832,-1.387667,-1.148967\O,0,-3.470748,-0.211335,-1.335001\H,0,-4.091581,-0.654929,-0.714573\C,0,-3.14496,-0.868955,1.567075\O,0,-4.197388,-1.357441,1.089772\H,0,-2.985903,-0.795991,2.655289\\Version=AM64L-G03RevD.01\State=2-A\HF=-905.5960179\MP2=-908.8415184\RMSD=5.710e-09\Thermal=0.\PG=C01 [X(C9H11N2O6)]\\@

007

1\1\GINC-NODE40\SP\ROMP2-FC\6-311+G(3df,2p)\C9H11N2O6(2)\ZIP06\17-Jan-2012\0\\#p ROMP2 (FC)/6-311+G(3df,2p) scf=tight\\RadKet5dU_007\\0,2\C,0,-2.117755,-0.599303,0.556045\O,0,-1.012178,-0.188226,1.188822\C,0,-0.294077,0.809377,0.366047\N,0,1.011844,0.312232,0.052079\C,0,2.10183,1.046951,0.495661\O,0,2.023931,2.098829,1.104633\N,0,3.350309,0.496825,0.169142\C,0,3.641132,-0.806548,-0.222879\O,0,4.78293,-1.188083,-0.375455\C,0,2.406798,-1.67162,-0.389552\C,0,1.196047,-0.848117,-0.826201\H,0,4.139609,1.082165,0.421222\H,0,2.632413,-2.458498,-1.112961\H,0,0.299364,-

1.467832,-0.766704\C,0,-1.254002,1.118939,-0.808674\H,0,2.203857,-2.152239,0.576587\H,0,1.312666,-0.523641,-1.870074\H,0,-0.698634,1.285647,-1.74187\O,0,-2.008708,2.25075,-0.447976\H,0,-2.91827, 2.065351,-0.754887\C,0,-2.180088,-0.118458,-0.871361\H,0,-0.165098,1.696063,0.983293\H,0,-1.757314,-0.883737,-1.544658\O,0,-3.471716,0.250819,-1.302392\H,0,-4.082063,-0.392594,-0.878019\C,0,-3.119729, -1.366986,1.19208\O,0,-4.167615,-1.677781,0.576539\H,0,-2.955697,-1.669943,2.239147\\Version=AM64 L-G03RevD.01\State=2-A\HF=-905.5951227\MP2=-908.8406424\RMSD=3.743e-09\Thermal=0.\PG=C01 [X(C9H11N2O6)]\\@

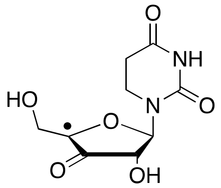

017

1\1\GINC-NODE23\SP\ROMP2-FC\6-311+G(3df,2p)\C9H11N2O6(2)\ZIP06\19-Jan-2012\0\\#p ROMP2 (FC)/6-311+G(3df,2p) scf=tight\\Rad4Ket3dU_017\\0,2\O,0,-4.409935,-1.674555,-0.205464\C,0,-3.323856,-1.657103,0.693364\C,0,-2.299259,-0.633541,0.315225\O,0,-1.044438,-0.741219,0.774005\C,0,-0.297631,0.522071,0.463156\N,0,1.041891,0.188602,0.115896\C,0,2.002442,0.324623,1.116268\O,0,1.790786,0.80304,2.215084\N,0,3.280834,-0.120851,0.758664\C,0,3.758486,-0.475038,-0.501553\O,0,4.926205, -0.751176,-0.682073\C,0,2.696171,-0.447121,-1.584034\C,0,1.307632,-0.717111,-1.010485\H,0,3.971422,-0.037086,1.497089\H,0,-3.645325,-1.461832,1.734462\H,0,-2.867607,-2.654723,0.69338\H,0,2.725561, 0.544015,-2.05559\H,0,1.220821,-1.761266,-0.681787\H,0,-4.621808,-0.74839,-0.429133\C,0,-1.156729, 1.243116,-0.586536\H,0,2.964022,-1.18496,-2.344137\H,0,0.54593,-0.553189,-1.775539\H,0,-0.769483, 1.080937,-1.606181\O,0,-1.237712,2.621748,-0.302719\H,0,-2.110344,2.907384,-0.630349\C,0,-2.51056, 0.545094,-0.441454\O,0,-3.582542,1.000074,-0.885475\H,0,-0.27136,1.101608,1.385308\\Version=AM64 L-G03RevD.01\State=2-A\HF=-905.6000371\MP2=-908.8404581\RMSD=9.634e-09\Thermal=0.\PG=C01 [X(C9H11N2O6)]\\@

011

1\1\GINC-NODE11\SP\ROMP2-FC\6-311+G(3df,2p)\C9H11N2O6(2)\ZIP06\19-Jan-2012\0\\#p ROMP2 (FC)/6-311+G(3df,2p) scf=tight\\Rad4Ket3dU_011\\0,2\O,0,-4.37659,-1.433196,0.435468\C,0,-3.031607,-1.711029,0.76016\C,0,-2.132446,-0.554526,0.456048\O,0,-0.983728,-0.407722,1.133707\C,0,-0.258298, 0.785692,0.626966\N,0,1.09955,0.430253,0.332628\C,0,1.319689,-0.46141,-0.706766\O,0,0.450811,-0.84734,-1.470898\N,0,2.642231,-0.899189,-0.841619\C,0,3.783391,-0.427058,-0.195608\O,0,4.887657,-0.847553,-0.470827\C,0,3.495552,0.654002,0.829013\C,0,2.093521,0.512666,1.415827\H,0,2.787191,-1.565957,-1.592496\H,0,-2.978986,-1.947261,1.830085\H,0,-2.651037,-2.599598,0.220901\H,0,4.260501, 0.599234,1.607184\H,0,1.8516,1.384814,2.027879\H,0,-4.374486,-0.895448,-0.380579\C,0,-1.11236, 1.36333,-0.514819\H,0,3.598235,1.625265,0.327343\H,0,2.033268,-0.375584,2.06081\H,0,-0.585048, 1.347033,-1.473453\O,0,-1.497181,2.685166,-0.154238\H,0,-2.272145,2.898217,-0.702791\C,0,-2.321403, 0.427208,-0.546511\O,0,-3.309589,0.572993,-1.29307\H,0,-0.248833,1.499814,1.451059\\Version=AM64

L-G03RevD.01\State=2-A\HF=-905.6015276\MP2=-908.8417578\RMSD=3.090e-09\Thermal=0.\PG=C01 [X(C9H11N2O6)]\\@

014

1\1\GINC-NODE15\SP\ROMP2-FC\6-311+G(3df,2p)\C9H11N2O6(2)\ZIP06\19-Jan-2012\0\\#p ROMP2 (FC)/6-311+G(3df,2p) scf=tight\\Rad4Ket3dU_014\\0,2\O,0,-4.352017,-1.776475,0.126884\C,0,-3.22915,-1.622322,0.965416\C,0,-2.256094,-0.623554,0.42152\O,0,-0.978341,-0.64105,0.829115\C,0,-0.295894,0.607719,0.356423\N,0,1.045777,0.278669,0.024375\C,0,2.058498,0.790836,0.82311\O,0,1.882442,1.559265,1.750907\N,0,3.347432,0.36961,0.466626\C,0,3.706961,-0.735621,-0.300954\O,0,4.866687,-1.06337,-0.44233\C,0,2.517299,-1.476471,-0.881307\C,0,1.349458,-0.531977,-1.160828\H,0,4.091403,0.820145,0.988747\H,0,-3.511543,-1.31319,1.99019\H,0,-2.741449,-2.600241,1.061842\H,0,2.840801,-1.987734,-1.790868\H,0,0.459373,-1.112999,-1.412394\H,0,-4.604562,-0.889759,-0.193311\C,0,-1.211022,1.15562,-0.75173\H,0,2.21432,-2.242291,-0.155117\H,0,1.581455,0.118117,-2.015464\H,0,-0.852614,0.852672,-1.749778\O,0,-1.323094,2.55712,-0.668081\H,0,-2.220179,2.768982,-0.985901\C,0,-2.537001,0.45016,-0.458585\O,0,-3.639686,0.822509,-0.903107\H,0,-0.275727,1.298015,1.199558\\Version=AM64L-G03RevD.01\State=2-A\HF=-905.5994991\MP2=-908.8398747\RMSD=4.190e-09\Thermal=0.\PG=C01 [X(C9H11N2O6)]\\@

026

1\1\GINC-NODE23\SP\ROMP2-FC\6-311+G(3df,2p)\C9H11N2O6(2)\ZIP06\24-Jan-2012\0\\#p ROMP2 (FC)/6-311+G(3df,2p) scf=tight\\Rad4Ket3dU_026\\0,2\O,0,-4.600966,-1.364972,0.155143\C,0,-3.270918,-1.834618,0.137941\C,0,-2.280588,-0.712304,0.088327\O,0,-1.039524,-0.890941,0.56008\C,0,-0.304569,0.415129,0.522172\N,0,1.046614,0.16949,0.144947\C,0,1.983529,0.103057,1.174705\O,0,1.74397,0.351447,2.341473\N,0,3.272526,-0.260356,0.765307\C,0,3.780722,-0.355955,-0.528405\O,0,4.953449,-0.590538,-0.73353\C,0,2.74338,-0.111944,-1.608121\C,0,1.343313,-0.49168,-1.133279\H,0,3.945342,-0.325606,1.521818\H,0,-3.104127,-2.426814,1.045773\H,0,-3.083729,-2.511649,-0.718037\H,0,2.778552,0.953718,-1.87024\H,0,1.253675,-1.580789,-1.023711\H,0,-4.644347,-0.570253,-0.410792\C,0,-1.148275,1.323311,-0.385074\H,0,3.03231,-0.682118,-2.494328\H,0,0.599401,-0.176606,-1.868177\H,0,-0.712327,1.40404,-1.394544\O,0,-1.286556,2.601866,0.19655\H,0,-2.126155,2.960834,-0.142934\C,0,-2.480178,0.574867,-0.46914\O,0,-3.528553,1.059752,-0.938821\H,0,-0.305631,0.79921,1.541479\\Version=AM64L-G03RevD.01\State=2-A\HF=-905.5989218\MP2=-908.8395743\RMSD=3.520e-09\Thermal=0.\PG=C01 [X(C9H11N2O6)]\\@

024

1\1\GINC-NODE26\SP\ROMP2-FC\6-311+G(3df,2p)\C9H11N2O6(2)\ZIP06\19-Jan-2012\0\\#p ROMP2 (FC)/6-311+G(3df,2p) scf=tight\\Rad4Ket3dU_024\\0,2\O,0,-4.543554,-1.355332,0.649221\C,0,-3.205159,-1.802167,0.649879\C,0,-2.251626,-0.707741,0.28168\O,0,-0.980911,-0.746512,0.708521\C,0,-0.306136,0.543393,0.35204\N,0,1.050594,0.260921,0.037221\C,0,2.030606,0.734174,0.898661\O,0,1.813403,1.429823,1.873671\N,0,3.337563,0.365702,0.54923\C,0,3.742227,-0.670179,-0.288625\O,0,4.912363,-0.961965,-0.422967\C,0,2.585366,-1.39108,-0.954563\C,0,1.402274,-0.454629,-1.19457\H,0,4.057175,0.791913,1.123367\H,0,-2.967524,-2.170105,1.655109\H,0,-3.05511,-2.654014,-0.041331\H,0,2.943968,-1.825093,-

1.890743\H,0,0.532195,-1.03543,-1.509132\H,0,-4.646097,-0.721572,-0.086535\C,0,-1.197542,1.159859,-0.738937\H,0,2.28322,-2.216024,-0.295864\H,0,1.638749,0.260042,-1.994772\H,0,-0.802068,0.943117,-1.745622\O,0,-1.334919,2.549111,-0.547483\H,0,-2.210201,2.782613,-0.906566\C,0,-2.516383,0.406916,-0.552477\O,0,-3.600168,0.757236,-1.05803\H,0,-0.322896,1.16803,1.244941\\Version=AM64L-G03RevD.01\State=2-A\HF=-905.5984338\MP2=-908.8388548\RMSD=6.161e-09\Thermal=0.\PG=C01 [X(C9H11N2O6)]\\@

016

1\1\GINC-NODE12\SP\ROMP2-FC\6-311+G(3df,2p)\C9H11N2O6(2)\ZIP06\19-Jan-2012\0\\#p ROMP2(FC)/6-311+G(3df,2p) scf=tight\\Rad4Ket3dU_016\\0,2\O,0,4.337368,-1.540346,-0.465663\C,0,2.973113,-1.810073,-0.706797\C,0,2.112107,-0.616853,-0.437126\O,0,0.936562,-0.487747,-1.071043\C,0,0.267694,0.754703,-0.629988\N,0,-1.097428,0.446705,-0.306502\C,0,-1.34774,-0.3459,0.794288\O,0,-0.479247,-0.768311,1.540966\N,0,-2.700489,-0.640395,1.016626\C,0,-3.76373,-0.513992,0.125059\O,0,-4.877456,-0.907247,0.402909\C,0,-3.36956,0.111591,-1.200522\C,0,-2.201104,1.082099,-1.039655\H,0,-2.879243,-1.167194,1.865052\H,0,2.864062,-2.114276,-1.75495\H,0,2.602855,-2.652905,-0.092406\H,0,-3.094232,-0.703321,-1.883161\H,0,-2.531231,1.988944,-0.513878\H,0,4.385638,-0.954239,0.314734\C,0,1.165941,1.368617,0.462056\H,0,-4.246774,0.612495,-1.61673\H,0,-1.826423,1.388483,-2.019069\H,0,0.668368,1.384774,1.437441\O,0,1.545154,2.675304,0.047962\H,0,2.349067,2.893803,0.551024\C,0,2.36518,0.421549,0.490949\O,0,3.389687,0.596358,1.180193\H,0,0.265376,1.424738,-1.491086\\Version=AM64L-G03RevD.01\State=2-A\HF=-905.5993795\MP2=-908.8392185\RMSD=3.672e-09\Thermal=0.\PG=C01 [X(C9H11N2O6)]\\@

047

1\1\GINC-NODE17\SP\ROMP2-FC\6-311+G(3df,2p)\C9H11N2O6(2)\ZIP06\24-Jan-2012\0\\#p ROMP2(FC)/6-311+G(3df,2p) scf=tight\\Rad4Ket3dU_047\\0,2\O,0,-4.113125,-1.876381,0.04757\C,0,-3.052549,-1.669774,0.950897\C,0,-2.135786,-0.572123,0.51403\O,0,-0.932841,-0.453266,1.096738\C,0,-0.263019,0.773441,0.607817\N,0,1.097418,0.451893,0.284737\C,0,1.341717,-0.370802,-0.796615\O,0,0.470655,-0.809841,-1.529309\N,0,2.693411,-0.674812,-1.014525\C,0,3.759509,-0.529858,-0.129589\O,0,4.871066,-0.934561,-0.399791\C,0,3.371526,0.131913,1.180115\C,0,2.206594,1.102457,0.996431\H,0,2.867441,-1.224725,-1.849201\H,0,-3.415947,-1.4326,1.970314\H,0,-2.485318,-2.60613,1.037786\H,0,3.094755,-0.663487,1.884811\H,0,2.538395,1.993174,0.444868\H,0,-4.334018,-1.014757,-0.356587\C,0,-1.174694,1.35561,-0.490249\H,0,4.251818,0.640225,1.580582\H,0,1.836678,1.437116,1.968371\H,0,-0.715946,1.27896,-1.481621\O,0,-1.48394,2.702429,-0.159677\H,0,-2.318188,2.902599,-0.620279\C,0,-2.415035,0.467007,-0.405011\O,0,-3.490942,0.702976,-0.991004\H,0,-0.252032,1.471984,1.446314\\Version=AM64L-G03RevD.01\State=2-A\HF=-905.5987366\MP2=-908.8386118\RMSD=5.839e-09\Thermal=0.\PG=C01 [X(C9H11N2O6)]\\@

031

1\1\GINC-NODE10\SP\ROMP2-FC\6-311+G(3df,2p)\C9H11N2O6(2)\ZIP06\24-Jan-2012\0\\#p ROMP2(FC)/6-311+G(3df,2p) scf=tight\\Rad4Ket3dU_031\\0,2\O,0,-3.60716,-2.465747,0.128985\C,0,-3.416178,-1.460776,-0.858726\C,0,-2.413217,-0.437344,-0.44325\O,0,-1.170131,-0.867882,-0.148703\C,0,-0.410107,

0.243279,0.50661\N,0,0.965148,0.13086,0.15368\C,0,1.799832,-0.495332,1.076711\O,0,1.454036,-0.856855,2.186237\N,0,3.118776,-0.674875,0.640539\C,0,3.746882,-0.132464,-0.478661\O,0,4.932066,-0.29564,-0.682043\C,0,2.817831,0.677019,-1.362879\C,0,1.376768,0.185022,-1.255533\H,0,3.716528,-1.147637,1.31016\H,0,-3.119587,-1.901665,-1.825588\H,0,-4.376893,-0.958517,-0.995417\H,0,2.884394,1.72622,-1.046222\H,0,1.271236,-0.80591,-1.717559\H,0,-2.748028,-2.891825,0.279063\C,0,-1.166624,1.512527,0.099719\H,0,3.184749,0.615734,-2.390312\H,0,0.708156,0.864377,-1.78735\H,0,-0.726901,1.97135,-0.802231\O,0,-1.219101,2.444772,1.151864\H,0,-2.091213,2.875367,1.071748\C,0,-2.557898,0.965242,-0.254066\O,0,-3.572206,1.66995,-0.313512\H,0,-0.493661,0.088786,1.582009\\Version=AM64L-G03RevD.01\State=2-A\HF=-905.5998286\MP2=-908.8384723\RMSD=7.536e-09\Thermal=0.\PG=C01 [X(C9H11N2O6)]\\@

004

1\1\GINC-NODE15\SP\ROMP2-FC\6-311+G(3df,2p)\C9H11N2O6(2)\ZIP06\18-Jan-2012\0\\#p ROMP2 (FC)/6-311+G(3df,2p) scf=tight\\Rad4Ket3dU_004\\0,2\O,0,-4.409833,-1.550782,-0.679203\C,0,-3.41656,-1.714054,0.305965\C,0,-2.351606,-0.668668,0.208058\O,0,-1.136912,-0.914775,0.747429\C,0,-0.339579,0.302632,0.710627\N,0,1.00293,-0.07386,0.266178\C,0,1.88255,0.972887,0.110524\O,0,1.566806,2.159198,0.162753\N,0,3.212132,0.615457,-0.123486\C,0,3.732367,-0.65303,-0.383317\O,0,4.903889,-0.81216,-0.653349\C,0,2.692164,-1.753376,-0.315509\C,0,1.550666,-1.393806,0.632151\H,0,3.846212,1.400513,-0.227939\H,0,-3.838539,-1.689629,1.329979\H,0,-2.969615,-2.709182,0.181497\H,0,2.303385,-1.909823,-1.330426\H,0,1.902847,-1.385678,1.674807\H,0,-4.543902,-0.589847,-0.800683\C,0,-1.088835,1.300177,-0.214599\H,0,3.188877,-2.675819,-0.005135\H,0,0.747662,-2.124042,0.556029\H,0,-0.616045,1.300638,-1.209038\O,0,-1.192936,2.586301,0.322775\H,0,-0.277853,2.925574,0.326437\C,0,-2.46958,0.636613,-0.336356\O,0,-3.489977,1.130755,-0.842023\H,0,-0.326284,0.701583,1.733303\\Version=AM64L-G03RevD.01\State=2-A\HF=-905.5970504\MP2=-908.8360205\RMSD=7.000e-09\Thermal=0.\PG=C01 [X(C9H11N2O6)]\\@

009

1\1\GINC-NODE12\SP\ROMP2-FC\6-311+G(3df,2p)\C9H11N2O6(2)\ZIP06\24-Jan-2012\0\\#p ROMP2 (FC)/6-311+G(3df,2p) scf=tight\\Rad4Ket3dU_009\\0,2\O,0,3.526615,-2.397575,-0.833233\C,0,3.196754,-1.696719,0.360788\C,0,2.273622,-0.550447,0.122932\O,0,1.128662,-0.804765,-0.54099\C,0,0.376413,0.458566,-0.742066\N,0,-1.007108,0.24268,-0.430901\C,0,-1.333797,-0.070247,0.88011\O,0,-0.542858,-0.048114,1.807884\N,0,-2.674709,-0.41824,1.088368\C,0,-3.747516,-0.309194,0.206346\O,0,-4.881275,-0.57227,0.549705\C,0,-3.349715,0.18691,-1.170874\C,0,-1.910065,-0.192069,-1.508875\H,0,-2.896803,-0.666306,2.046738\H,0,2.756163,-2.37418,1.111779\H,0,4.128239,-1.299304,0.771152\H,0,-4.051932,-0.226106,-1.898953\H,0,-1.597012,0.304118,-2.430497\H,0,2.696763,-2.739697,-1.201457\C,0,1.133983,1.54025,0.042673\H,0,-3.463681,1.278923,-1.178906\H,0,-1.825856,-1.276922,-1.667851\H,0,0.570442,1.876741,0.918741\O,0,1.437376,2.621413,-0.82611\H,0,2.232846,3.039486,-0.449612\C,0,2.414271,0.817152,0.481188\O,0,3.387806,1.390912,0.986392\H,0,0.449261,0.690158,-1.805917\\Version=AM64L-G03RevD.01\State=2-A\HF=-905.6012463\MP2=-908.8393586\RMSD=8.232e-09\Thermal=0.\PG=C01 [X(C9H11N2O6)]\\@

003

1\1\GINC-NODE16\SP\ROMP2-FC\6-311+G(3df,2p)\C9H11N2O6(2)\ZIP06\17-Jan-2012\0\\#p ROMP2 (FC)/6-311+G(3df,2p) scf=tight\\Rad4Ket3dU_003\\0,2\O,0,-4.678673,-1.283883,-0.063991\C,0,-3.368178,-1.79586,0.005432\C,0,-2.344779,-0.708248,0.086538\O,0,-1.127586,-0.983858,0.60534\C,0,-0.345884,0.243654,0.667009\N,0,1.013993,-0.092541,0.245055\C,0,1.883005,0.970669,0.148764 \O,0,1.552125,2.150841,0.231646\N,0,3.222935,0.637306,-0.062377\C,0,3.767352,-0.614964,-0.349496 \O,0,4.946777,-0.749309,-0.597778\C,0,2.742385,-1.731651,-0.338323\C,0,1.57265,-1.414112, 0.589353\H,0,3.84844,1.433605,-0.125494\H,0,-3.293445,-2.434873,0.894877\H,0,-3.130518,-2.441475,-0.863203\H,0,2.381793,-1.865577,-1.36678\H,0,1.898616,-1.428645,1.640368\H,0,-4.64255,-0.453912,-0.580224\C,0,-1.082241,1.283303,-0.221079\H,0,3.245132,-2.655053,-0.040706\H,0,0.782565,-2.153243, 0.473753\H,0,-0.587574,1.338044,-1.203354\O,0,-1.208883,2.539573,0.37792\H,0,-0.29802,2.888411, 0.411756\C,0,-2.452614,0.615636,-0.413463\O,0,-3.448956,1.112633,-0.963086\H,0,-0.371543,0.580812, 1.711438\\Version=AM64L-G03RevD.01\State=2-A\HF=-905.5966147\MP2=-908.8356604\RMSD= 6.850e-09\Thermal=0.\PG=C01 [X(C9H11N2O6)]\\@

029

1\1\GINC-NODE15\SP\ROMP2-FC\6-311+G(3df,2p)\C9H11N2O6(2)\ZIP06\24-Jan-2012\0\\#p ROMP2 (FC)/6-311+G(3df,2p) scf=tight\\Rad4Ket3dU_029\\0,2\O,0,-3.458831,-2.414519,0.992852\C,0,-3.34262,-1.770399,-0.269468\C,0,-2.38892,-0.623672,-0.240053\O,0,-1.118441,-0.877339,0.137562\C,0,-0.417495, 0.42024,0.387358\N,0,0.961139,0.247729,0.083701\C,0,1.869074,0.307003,1.131087\O,0,1.571814,0.5308 88,2.290371\N,0,3.205214,0.10507,0.754992\C,0,3.68979,-0.48282,-0.409958\O,0,4.872626,-0.701797,-0.570489\C,0,2.597669,-0.844938,-1.398384\C,0,1.412414,0.114468,-1.306115\H,0,3.873302,0.243181, 1.505754\H,0,-3.043106,-2.483961,-1.055944\H,0,-4.331769,-1.382048,-0.524025\H,0,3.028911,-0.846396, -2.402076\H,0,0.579698,-0.274194,-1.896576\H,0,-2.575577,-2.733826,1.237637\C,0,-1.22917,1.439965,-0.422868\H,0,2.270856,-1.868664,-1.172476\H,0,1.685016,1.097756,-1.71342\H,0,-0.818527,1.548875,-1.441409\O,0,-1.302368,2.687113,0.220066\H,0,-2.200819,3.020732,0.035024\C,0,-2.600553,0.75546,-0.519878\O,0,-3.64848,1.361006,-0.771158\H,0,-0.504157,0.634297,1.452689\\Version=AM64L-G03RevD.01\State=2-A\HF=-905.5993728\MP2=-908.8379142\RMSD=6.049e-09\Thermal=0.\PG=C01 [X(C9H11N2O6)]\\@

061

1\1\GINC-NODE11\SP\ROMP2-FC\6-311+G(3df,2p)\C9H11N2O6(2)\ZIP06\19-Jan-2012\0\\#p ROMP2 (FC)/6-311+G(3df,2p) scf=tight\\Rad4Ket3dU_061\\0,2\O,0,-4.396567,-1.604723,-0.448901\C,0,-3.434096,-1.599969,0.580152\C,0,-2.350367,-0.599755,0.329594\O,0,-1.143581,-0.778568,0.913748\C,0,-0.325292,0.396912,0.674372\N,0,1.01991,-0.05227,0.332733\C,0,1.947498,0.956175,0.280426\O,0, 1.690333,2.130393,0.54079\N,0,3.235054,0.571149,-0.096005\C,0,3.724925,-0.724417,-0.266839 \O,0,4.890231,-0.928411,-0.533906\C,0,2.678113,-1.804405,-0.06005\C,0,1.269798,-1.308517,-0.387445 \H,0,3.906385,1.328818,-0.160778\H,0,-3.884051,-1.388845,1.569859\H,0,-2.99995,-2.605807,0.647517 \H,0,2.950118,-2.663679,-0.677752\H,0,0.524731,-2.03527,-0.066408\H,0,-4.530689,-0.676263,-0.724179 \C,0,-1.062731,1.266122,-0.377879\H,0,2.729436,-2.119818,0.990423\H,0,1.156684,-1.162765,-1.472074 \H,0,-0.58101,1.155421,-1.364532\O,0,-1.153436,2.611603,0.010374\H,0,-0.233093,2.907407,0.146999

\C,0,-2.446464,0.603066,-0.42154\O,0,-3.456963,1.014534,-1.014748\H,0,-0.271738,0.958743,1.610226\\Version=AM64L-G03RevD.01\State=2-A\HF=-905.5963948\MP2=-908.8355369\RMSD=4.552e-09\Thermal=0.\PG=C01 [X(C9H11N2O6)]\\@

057

1\1\GINC-NODE23\SP\ROMP2-FC\6-311+G(3df,2p)\C9H11N2O6(2)\ZIP06\24-Jan-2012\0\\#p ROMP2 (FC)/6-311+G(3df,2p) scf=tight\\Rad4Ket3dU_057\\0,2\O,0,-2.771579,-2.84113,0.153472\C,0,-3.275773,-1.590838,0.611329\C,0,-2.339313,-0.458885,0.357563\O,0,-1.138144,-0.497459,0.96951\C,0,-0.37862,0.740638,0.659642\N,0,0.984036,0.410301,0.363004\C,0,1.239801,-0.337736,-0.777115\O,0,0.39744,-0.631743,-1.60725\N,0,2.572064,-0.744851,-0.929575\C,0,3.692456,-0.337296,-0.208979\O,0,4.809226,-0.706005,-0.508019\C,0,3.366065,0.608811,0.931044\C,0,1.956424,0.373479,1.467371\H,0,2.742164,-1.310417,-1.754607\H,0,-4.197342,-1.396462,0.057878\H,0,-3.520795,-1.630943,1.686163\H,0,4.117718,0.474616,1.712582\H,0,1.688005,1.158392,2.178311\H,0,-1.914418,-2.983213,0.585455\C,0,-1.195267,1.489494,-0.403743\H,0,3.45799,1.634828,0.551121\H,0,1.902712,-0.589112,1.996239\H,0,-0.71017,1.459157,-1.38457\O,0,-1.415609,2.823603,0.028335\H,0,-2.232725,3.107186,-0.420381\C,0,-2.511138,0.70166,-0.446363\O,0,-3.52726,1.096218,-1.031348\H,0,-0.386254,1.333976,1.57571\\Version=AM64L-G03RevD.01\State=2-A\HF=-905.6001105\MP2=-908.8382875\RMSD=5.392e-09\Thermal=0.\PG=C01 [X(C9H11N2O6)]\\@

005

1\1\GINC-NODE10\SP\ROMP2-FC\6-311+G(3df,2p)\C9H11N2O6(2)\ZIP06\24-Jan-2012\0\\#p ROMP2 (FC)/6-311+G(3df,2p) scf=tight\\Rad4Ket3dU_005\\0,2\O,0,-2.493436,-2.665534,-0.032106\C,0,-2.94768,-1.678295,0.895361\C,0,-2.282163,-0.373704,0.628891\O,0,-1.104888,-0.147278,1.257008\C,0,-0.390291,0.96183,0.612259\N,0,0.957025,0.539361,0.311781\C,0,1.114705,-0.576663,-0.486366\O,0,0.193959,-1.158911,-1.045503\N,0,2.431688,-1.023203,-0.633299\C,0,3.610501,-0.381217,-0.253765\O,0,4.698629,-0.845314,-0.521141\C,0,3.377071,0.928271,0.47529\C,0,2.046295,0.924211,1.22351\H,0,2.530579,-1.858568,-1.200669\H,0,-4.025702,-1.576615,0.743222\H,0,-2.762824,-1.984386,1.935553\H,0,4.215822,1.095637,1.155113\H,0,1.827929,1.923853,1.60586\H,0,-1.553133,-2.495543,-0.215699\C,0,-1.259573,1.413035,-0.570735\H,0,3.386667,1.732808,-0.271872\H,0,2.089204,0.238328,2.081928\H,0,-0.777865,1.180825,-1.528855\O,0,-1.542238,2.797383,-0.456902\H,0,-2.381974,2.929454,-0.933322\C,0,-2.526579,0.560217,-0.42163\O,0,-3.550979,0.739889,-1.085974\H,0,-0.331793,1.773107,1.339415\\Version=AM64L-G03RevD.01\State=2-A\HF=-905.5990101\MP2=-908.8379664\RMSD=2.634e-09\Thermal=0.\PG=C01 [X(C9H11N2O6)]\\@

007

1\1\GINC-NODE17\SP\ROMP2-FC\6-311+G(3df,2p)\C9H11N2O6(2)\ZIP06\24-Jan-2012\0\\#p ROMP2 (FC)/6-311+G(3df,2p) scf=tight\\Rad4Ket3dU_007\\0,2\O,0,4.686256,-1.223387,-0.141151\C,0,3.384741,-1.731325,-0.31766\C,0,2.343261,-0.662673,-0.215829\O,0,1.134577,-0.86806,-0.787361\C,0,0.33214,0.331945,-0.631272\N,0,-1.030145,-0.075437,-0.305476\C,0,-1.94083,0.949213,-0.332274\O,0,-1.6548,2.105062,-0.639898\N,0,-3.246516,0.60367,0.01988\C,0,-3.765067,-0.674343,0.232825\O,0,-4.941573,-0.846739,0.471437\C,0,-2.732033,-1.780012,0.108007\C,0,-1.326697,-1.293024,0.460769\H,0,-3.905764,

1.374581,0.027799\H,0,3.327921,-2.200855,-1.308223\H,0,3.148635,-2.526704,0.417259\H,0,-3.039875,-2.606488,0.753007\H,0,-0.584408,-2.045715,0.198223\H,0,4.632818,-0.497877,0.512406\C,0,1.058194,1.240138,0.395493\H,0,-2.754226,-2.140407,-0.9289\H,0,-1.248107,-1.099995,1.541147\H,0,0.553059,1.181568,1.375056\O,0,1.17764,2.564627,-0.05172\H,0,0.265772,2.864536,-0.23022\C,0,2.428936,0.558605,0.506099\O,0,3.414095,0.958805,1.148709\H,0,0.315375,0.846125,-1.595467\\Version=AM64L-G03RevD.01\State=2-A\HF=-905.5956741\MP2=-908.8348492\RMSD=5.558e-09\Thermal=0.\PG=C01 [X(C9H11N2O6)]\\@

018

1\1\GINC-NODE20\SP\ROMP2-FC\6-311+G(3df,2p)\C9H11N2O6(2)\ZIP06\19-Jan-2012\0\\#p ROMP2 (FC)/6-311+G(3df,2p) scf=tight\\Rad4Ket3dU_018\\0,2\O,0,3.01006,2.746151,-0.104404\C,0,3.413463,1.553342,0.568554\C,0,2.420953,0.486075,0.275776\O,0,1.163477,0.631504,0.72377\C,0,0.392807,-0.617916,0.456591\N,0,-0.945673,-0.261703,0.116682\C,0,-1.899675,-0.370042,1.12379\O,0,-1.69425,-0.850944,2.223346\N,0,-3.1706,0.105871,0.77472\C,0,-3.648697,0.469801,-0.481728\O,0,-4.811172,0.773984,-0.654183\C,0,-2.595479,0.414026,-1.571877\C,0,-1.196187,0.649217,-1.009513\H,0,-3.85697,0.040093,1.518702\H,0,4.391457,1.18981,0.223447\H,0,3.463206,1.710544,1.657992\H,0,-2.654164,-0.576594,-2.041943\H,0,-1.076916,1.690805,-0.68399\H,0,3.630121,3.445999,0.153433\C,0,1.222305,-1.376018,-0.586386\H,0,-2.850259,1.157662,-2.330871\H,0,-0.444546,0.463996,-1.779474\H,0,0.844296,-1.194051,-1.606499\O,0,1.259335,-2.75676,-0.310397\H,0,2.134555,-3.057723,-0.618628\C,0,2.604924,-0.724539,-0.45202\O,0,3.645554,-1.232057,-0.885866\H,0,0.362611,-1.17841,1.390779\\Version=AM64L-G03RevD.01\State=2-A\HF=-905.5994099\MP2=-908.8375249\RMSD=6.357e-09\Thermal=0.\PG=C01 [X(C9H11N2O6)]\\@

010

1\1\GINC-NODE27\SP\ROMP2-FC\6-311+G(3df,2p)\C9H11N2O6(2)\ZIP06\24-Jan-2012\0\\#p ROMP2 (FC)/6-311+G(3df,2p) scf=tight\\Rad4Ket3dU_010\\0,2\O,0,3.399141,-2.517667,-0.864502\C,0,3.183366,-1.766274,0.324202\C,0,2.286871,-0.593813,0.113544\O,0,1.095731,-0.825404,-0.473942\C,0,0.393575,0.452676,-0.695082\N,0,-0.999471,0.26112,-0.396226\C,0,-1.363109,-0.002295,0.90893\O,0,-0.57492,-0.081728,1.837024\N,0,-2.740119,-0.178666,1.114136\C,0,-3.729477,-0.410735,0.162162\O,0,-4.875145,-0.654261,0.480125\C,0,-3.21675,-0.365199,-1.265727\C,0,-2.021098,0.574834,-1.404844\H,0,-3.00244,-0.314326,2.084699\H,0,2.774772,-2.400944,1.128796\H,0,4.156071,-1.392129,0.652915\H,0,-2.930199,-1.387002,-1.547973\H,0,-2.345856,1.619962,-1.304519\H,0,2.533734,-2.84621,-1.155194\C,0,1.188791,1.51471,0.08381\H,0,-4.040203,-0.058988,-1.915244\H,0,-1.567378,0.466296,-2.392803\H,0,0.669449,1.805683,1.004083\O,0,1.43904,2.633818,-0.749172\H,0,2.274944,3.01518,-0.423643\C,0,2.487264,0.77654,0.431616\O,0,3.506151,1.339464,0.852005\H,0,0.477653,0.678865,-1.760448\\Version=AM64L-G03RevD.01\State=2-A\HF=-905.5993994\MP2=-908.8370766\RMSD=5.198e-09\Thermal=0.\PG=C01 [X(C9H11N2O6)]\\@

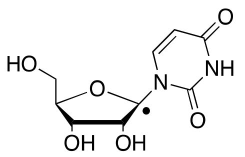

022

1\1\GINC-NODE12\SP\ROMP2-FC\6-311+G(3df,2p)\C9H11N2O6(2)\ZIP06\22-Jan-2012\0\\#p ROMP2 (FC)/6-311+G(3df,2p) scf=tight\\Rad1Uri_022\\0,2\O,0,3.067888,-2.398965,0.774638\C,0,3.349793,-1.711992,-0.433387\C,0,2.480007,-0.480626,-0.622081\O,0,1.082132,-0.909183,-0.645103\C,0,1.138896,1.230444,0.439703\O,0,1.295421,2.398307,-0.363857\C,0,0.336152,0.158985,-0.235386\N,0,-1.03335,-0.096974,-0.073543\C,0,-1.898067,1.017687,-0.024133\O,0,-1.492043,2.173575,-0.078104\N,0,-3.226318,0.696814,0.098614\C,0,-3.836959,-0.583186,0.099805\O,0,-5.049266,-0.68304,0.186584\C,0,-2.868844,-1.657749,-0.011921\C,0,-1.545546,-1.388104,-0.087381\H,0,0.388574,2.735149,-0.494772\H,0,-3.856599,1.490527,0.144108\H,0,4.392219,-1.384071,-0.37479\H,0,3.250004,-2.375055,-1.306419\H,0,2.70345,0.009098,-1.578082\H,0,-3.233413,-2.676176,-0.027144\H,0,-0.793961,-2.160926,-0.171949\H,0,2.144363,-2.691608,0.720091\C,0,2.531341,0.557143,0.519669\O,0,3.604231,1.453365,0.407982\H,0,3.262829,2.208497,-0.106993\H,0,0.742308,1.499761,1.426861\H,0,2.630373,0.017843,1.467138\\Version=AM64L-G03RevD.01\State=2-A\HF=-905.584607\MP2=-908.8244957\RMSD=5.506e-09\Thermal=0.\PG=C01 [X(C9H11N2O6)]\\@

009

1\1\GINC-NODE20\SP\ROMP2-FC\6-311+G(3df,2p)\C9H11N2O6(2)\ZIP06\21-Jan-2012\0\\#p ROMP2 (FC)/6-311+G(3df,2p) scf=tight\\Rad1Uri_009\\0,2\O,0,4.627901,-1.368915,-0.248786\C,0,3.28622,-1.800749,-0.109591\C,0,2.346948,-0.639283,-0.396162\O,0,0.959529,-1.068669,-0.403057\C,0,1.009567,1.174782,0.451224\O,0,1.260025,2.241207,-0.459891\C,0,0.215725,0.049868,-0.148788\N,0,-1.161906,-0.167431,-0.008482\C,0,-2.003319,0.954972,-0.155132\O,0,-1.567473,2.08519,-0.348141\N,0,-3.341938,0.672818,-0.053255\C,0,-3.975042,-0.587324,0.101142\O,0,-5.191477,-0.658899,0.144056\C,0,-3.023914,-1.680003,0.191408\C,0,-1.693787,-1.442259,0.140477\H,0,0.37528,2.56997,-0.710089\H,0,-3.959298,1.472169,-0.149005\H,0,3.131611,-2.609386,-0.831091\H,0,3.078353,-2.203234,0.896606\H,0,2.58125,-0.206358,-1.376235\H,0,-3.408382,-2.68456,0.307071\H,0,-0.950928,-2.22608,0.205868\H,0,4.711307,-0.503574,0.190326\C,0,2.36269,0.467255,0.678892\O,0,3.489358,1.308373,0.610928\H,0,3.26154,2.007507,-0.031294\H,0,0.567578,1.560227,1.37884\H,0,2.365158,-0.004251,1.66948\\Version=AM64L-G03RevD.01\State=2-A\HF=-905.5842575\MP2=-908.8237197\RMSD=8.384e-09\Thermal=0.\PG=C01 [X(C9H11N2O6)]\\@

073

1\1\GINC-NODE7\SP\ROMP2-FC\6-311+G(3df,2p)\C9H11N2O6(2)\ZIP06\24-Jan-2012\0\\#p ROMP2 (FC)/6-311+G(3df,2p) scf=tight\\Rad1Uri_073\\0,2\O,0,4.624015,-1.350982,-0.377662\C,0,3.316699,-1.779424,-0.039212\C,0,2.323245,-0.665124,-0.333166\O,0,0.95326,-1.111592,-0.155445\C,0,1.046965,1.196585,0.475821\O,0,1.133921,2.040037,-0.677577\C,0,0.226853,-0.03703,0.291448\N,0,-1.163979,-0.175219,0.165231\C,0,-1.980092,0.96038,-0.009111\O,0,-1.537613,2.102899,-0.093476\N,0,-3.324924,

0.688781,-0.061436\C,0,-3.984924,-0.564594,-0.044444\O,0,-5.199636,-0.620337,-0.133544\C,0,-3.05686,-1.672361,0.086384\C,0,-1.728908,-1.448329,0.19017\H,0,0.241,2.427133,-0.768139\H,0,-3.918728,1.502824,-0.180196\H,0,3.099068,-2.661339,-0.650276\H,0,3.234744,-2.076299,1.020279\H,0,2.439761,-0.331808,-1.371382\H,0,-3.456037,-2.677644,0.106521\H,0,-1.006573,-2.246467,0.295727\H,0,4.735238,-0.440373,-0.050859\C,0,2.439848,0.548986,0.611409\O,0,3.515016,1.40315,0.301093\H,0,3.187007,1.992728,-0.404997\H,0,0.750675,1.778823,1.354316\H,0,2.58749,0.197559,1.639521\\Version=AM64L-G03RevD.01\State=2-A\HF=-905.5822146\MP2=-908.8231906\RMSD=7.369e-09\Thermal=0.\PG=C01 [X(C9H11N2O6)]\\@

010

1\1\GINC-NODE23\SP\ROMP2-FC\6-311+G(3df,2p)\C9H11N2O6(2)\ZIP06\21-Jan-2012\0\\#p ROMP2(FC)/6-311+G(3df,2p) scf=tight\\Rad1Uri_010\\0,2\O,0,-3.397478,2.68209,-0.721065\C,0,-3.375162,1.620894,0.217057\C,0,-2.499368,0.466003,-0.224502\O,0,-1.11418,0.933674,-0.266268\C,0,-1.056398,-1.360627,0.424494\O,0,-1.262442,-2.372371,-0.557473\C,0,-0.322088,-0.166301,-0.106597\N,0,1.049253,0.098311,0.029006\C,0,1.935135,-0.970288,-0.222536\O,0,1.545729,-2.100458,-0.496722\N,0,3.262653,-0.637907,-0.123988\C,0,3.845421,0.631871,0.120523\O,0,5.058353,0.754079,0.144041\C,0,2.850864,1.669703,0.321469\C,0,1.531434,1.377074,0.27691\H,0,-0.365045,-2.63585,-0.838246\H,0,3.910652,-1.399366,-0.295957\H,0,-3.04863,1.962595,1.213419\H,0,-4.403405,1.256836,0.30635\H,0,-2.773568,0.142327,-1.235044\H,0,3.194471,2.67691,0.515943\H,0,0.758429,2.118558,0.427534\H,0,-2.482704,2.98661,-0.829861\C,0,-2.449474,-0.749644,0.725417\O,0,-3.513783,-1.6442,0.553102\H,0,-3.211538,-2.284823,-0.118153\H,0,-0.581667,-1.787928,1.317275\H,0,-2.47212,-0.393325,1.76328\\Version=AM64L-G03RevD.01\State=2-A\HF=-905.5852829\MP2=-908.8237039\RMSD=8.143e-09\Thermal=0.\PG=C01 [X(C9H11N2O6)]\\@

014

1\1\GINC-NODE24\SP\ROMP2-FC\6-311+G(3df,2p)\C9H11N2O6(2)\ZIP06\21-Jan-2012\0\\#p ROMP2(FC)/6-311+G(3df,2p) scf=tight\\Rad1Uri_014\\0,2\O,0,2.914641,-2.373053,0.727429\C,0,3.304202,-1.71623,-0.470715\C,0,2.467744,-0.465529,-0.636637\O,0,1.068873,-0.838593,-0.753375\C,0,1.129727,1.227968,0.459499\O,0,1.319884,2.431774,-0.283084\C,0,0.329351,0.205812,-0.288548\N,0,-1.032585,-0.077769,-0.092356\C,0,-1.911437,1.022116,-0.065203\O,0,-1.520431,2.182016,-0.149809\N,0,-3.234325,0.682531,0.072927\C,0,-3.818253,-0.609145,0.12211\O,0,-5.028645,-0.728917,0.216962\C,0,-2.830429,-1.66992,0.050683\C,0,-1.512883,-1.379407,-0.044069\H,0,0.417579,2.756677,-0.466238\H,0,-3.880055,1.464312,0.100294\H,0,4.356841,-1.395423,-0.443351\H,0,3.16706,-2.367474,-1.347596\H,0,2.763894,0.053413,-1.557306\H,0,-3.176673,-2.694225,0.086315\H,0,-0.739213,-2.134872,-0.09028\H,0,3.552983,-3.079778,0.903183\C,0,2.508969,0.530432,0.546938\O,0,3.600262,1.41223,0.490421\H,0,3.282206,2.189344,-0.006571\H,0,0.708456,1.454501,1.447342\H,0,2.576677,-0.040043,1.477519\\Version=AM64L-G03RevD.01\State=2-A\HF=-905.5862912\MP2=-908.8241676\RMSD=6.095e-09\Thermal=0.\PG=C01 [X(C9H11N2O6)]\\@

003

1\1\GINC-NODE17\SP\ROMP2-FC\6-311+G(3df,2p)\C9H11N2O6(2)\ZIP06\21-Jan-2012\0\\#p ROMP2 (FC)/6-311+G(3df,2p) scf=tight\\Rad1Uri_003\\0,2\O,0,-2.021785,2.122614,0.920067\C,0,-2.696972, 1.923815,-0.304723\C,0,-2.52176,0.506752,-0.846946\O,0,-1.120625,0.337625,-1.234447\C,0,-1.395401,- 0.856567,0.802209\O,0,-1.185276,-2.239426,1.125444\C,0,-0.504295,-0.460487,-0.331567\N,0,0.90041,- 0.298099,-0.227408\C,0,1.440686,0.951292,0.127913\O,0,0.756459,1.931775,0.393569\N,0,2.816152, 0.983428,0.157575\C,0,3.730442,-0.079041,-0.04608\O,0,4.929067,0.117919,0.048152\C,0,3.06961,- 1.337465,-0.356165\C,0,1.724447,-1.395861,-0.439871\H,0,-1.440664,-2.36971,2.053073\H,0,3.222563, 1.879531,0.405013\H,0,-3.764768,2.092208,-0.11844\H,0,-2.374918,2.640792,-1.074237\H,0,-3.118687, 0.352332,-1.7499\H,0,3.684586,-2.210776,-0.526859\H,0,1.183936,-2.304398,-0.676158\H,0,-1.062995, 2.177229,0.728614\C,0,-2.796365,-0.602113,0.182392\O,0,-3.249257,-1.764703,-0.480239\H,0,-2.750998,- 2.504021,-0.082764\H,0,-1.242671,-0.229905,1.69068\H,0,-3.522343,-0.268969,0.936478\\Version=AM64 L-G03RevD.01\State=2-A\HF=-905.5868954\MP2=-908.8259405\RMSD=4.963e-09\Thermal=0.\PG=C01 [X(C9H11N2O6)]\\@

005

1\1\GINC-NODE12\SP\ROMP2-FC\6-311+G(3df,2p)\C9H11N2O6(2)\ZIP06\21-Jan-2012\0\\#p ROMP2 (FC)/6-311+G(3df,2p) scf=tight\\Rad1Uri_005\\0,2\O,0,2.027476,-1.958136,1.590289\C,0,2.765228,- 2.076042,0.381019\C,0,2.544394,-0.899595,-0.562997\O,0,1.134107,-0.886945,-0.951104\C,0,1.425931, 0.974835,0.510128\O,0,1.412705,2.381251,0.405884\C,0,0.513049,0.215904,-0.440341\N,0,-0.878968,- 0.011496,-0.237168\C,0,-1.691981,1.065803,0.157979\O,0,-1.2548,2.185549,0.409626\N,0,-3.029151, 0.773698,0.254311\C,0,-3.701326,-0.439209,-0.034152\O,0,-4.911209,-0.517828,0.094812\C,0,-2.788214,- 1.47628,-0.473233\C,0,-1.46027,-1.235639,-0.557368\H,0,0.46738,2.637838,0.373568\H,0,-3.612677, 1.549234,0.550005\H,0,3.82287,-2.106354,0.663712\H,0,2.532021,-3.0148,-0.142172\H,0,3.126575,- 1.031869,-1.47912\H,0,-3.196255,-2.443954,-0.733475\H,0,-0.755271,-1.981049,-0.895388\H,0,1.100631,- 2.155934,1.386271\C,0,2.81736,0.485619,0.03289\O,0,3.302457,1.326845,-0.989337\H,0,2.871638, 2.190107,-0.829968\H,0,1.246036,0.653121,1.548242\H,0,3.52297,0.429964,0.873416\\Version=AM64L- G03RevD.01\State=2-A\HF=-905.583776\MP2=-908.8231324\RMSD=2.560e-09\Thermal=0.\PG=C01 [X(C9H11N2O6)]\\@

015

1\1\GINC-NODE17\SP\ROMP2-FC\6-311+G(3df,2p)\C9H11N2O6(2)\ZIP06\22-Jan-2012\0\\#p ROMP2 (FC)/6-311+G(3df,2p) scf=tight\\Rad1Uri_015\\0,2\O,0,-1.98882,2.155079,0.916583\C,0,-2.643657, 1.960479,-0.317412\C,0,-2.491003,0.529202,-0.831231\O,0,-1.09843,0.339952,-1.22837\C,0,-1.372867,- 0.918986,0.785792\O,0,-1.262835,-2.301151,1.097948\C,0,-0.483932,-0.485202,-0.34647\N,0,0.922085,- 0.315944,-0.239593\C,0,1.45784,0.934974,0.118538\O,0,0.772006,1.917131,0.373597\N,0,2.83307 ,0.969519,0.160063\C,0,3.751247,-0.092977,-0.021889\O,0,4.948664,0.106737,0.081805\C,0,3.094855,- 1.355307,-0.324407\C,0,1.750908,-1.414327,-0.424466\H,0,-1.5535,-2.763379,0.291211\H,0,3.234572, 1.867502,0.408715\H,0,-3.710568,2.158202,-0.155826\H,0,-2.287034,2.654897,-1.092326\H,0,-3.101779, 0.359021,-1.722461\H,0,3.711389,-2.23124,-0.474498\H,0,1.21717,-2.328,-0.655073\H,0,-1.025075, 2.178607,0.743039\C,0,-2.778857,-0.530022,0.241891\O,0,-3.39441,-1.655321,-0.384465\H,0,-3.720203,-

2.230025,0.327012\H,0,-1.150716,-0.382563,1.714575\H,0,-3.416936,-0.124464,1.033489\\Version=AM64L-G03RevD.01\State=2-A\HF=-905.5863383\MP2=-908.8246616\RMSD=1.780e-09\Thermal=0.\PG=C01 [X(C9H11N2O6)]\\@

023

1\1\GINC-NODE20\SP\ROMP2-FC\6-311+G(3df,2p)\C9H11N2O6(2)\ZIP06\22-Jan-2012\0\\#p ROMP2(FC)/6-311+G(3df,2p) scf=tight\\Rad1Uri_023\\0,2\O,0,-3.419777,2.70968,-0.615552\C,0,-3.386293,1.591668,0.254382\C,0,-2.478699,0.471183,-0.218177\O,0,-1.102114,0.934596,-0.251014\C,0,-1.038584,-1.365501,0.422177\O,0,-1.228107,-2.38114,-0.56072\C,0,-0.310859,-0.165109,-0.10271\N,0,1.059835,0.105609,0.024284\C,0,1.951397,-0.963031,-0.199365\O,0,1.570104,-2.102603,-0.448688\N,0,3.276706,-0.621655,-0.103783\C,0,3.852316,0.657394,0.108469\O,0,5.065127,0.784656,0.134181\C,0,2.851857,1.695226,0.272291\C,0,1.533522,1.395711,0.231179\H,0,-0.324693,-2.644339,-0.822846\H,0,3.92913,-1.383742,-0.254401\H,0,-3.021946,1.96929,1.21569\H,0,-4.386176,1.160073,0.419235\H,0,-2.750091,0.146896,-1.232362\H,0,3.189011,2.710489,0.433301\H,0,0.755065,2.137837,0.348534\H,0,-3.856924,2.437634,-1.437583\C,0,-2.438074,-0.764768,0.710782\O,0,-3.497027,-1.659429,0.502571\H,0,-3.165208,-2.308504,-0.146737\H,0,-0.569897,-1.790122,1.319268\H,0,-2.4792,-0.42591,1.753991\\Version=AM64L-G03RevD.01\State=2-A\HF=-905.583747\MP2=-908.8222318\RMSD=8.018e-09\Thermal=0.\PG=C01 [X(C9H11N2O6)]\\@

017

1\1\GINC-NODE19\SP\ROMP2-FC\6-311+G(3df,2p)\C9H11N2O6(2)\ZIP06\22-Jan-2012\0\\#p ROMP2(FC)/6-311+G(3df,2p) scf=tight\\Rad1Uri_017\\0,2\O,0,-3.440138,2.582021,-0.747969\C,0,-3.395015,1.572594,0.247388\C,0,-2.476196,0.469112,-0.218291\O,0,-1.09968,0.940502,-0.234477\C,0,-1.034428,-1.364853,0.417837\O,0,-1.225342,-2.372783,-0.571983\C,0,-0.307693,-0.159369,-0.096982\N,0,1.063441,0.109776,0.026954\C,0,1.954678,-0.958866,-0.199079\O,0,1.573359,-2.097985,-0.44996\N,0,3.280281,-0.618042,-0.103414\C,0,3.856919,0.66046,0.10867\O,0,5.070073,0.786827,0.134696\C,0,2.857166,1.69847,0.27221\C,0,1.538416,1.399266,0.232037\H,0,-0.322659,-2.63304,-0.838872\H,0,3.931996,-1.380323,-0.255927\H,0,-3.028388,1.962564,1.211069\H,0,-4.382628,1.115327,0.418058\H,0,-2.742139,0.161768,-1.236192\H,0,3.194695,2.713883,0.431438\H,0,0.76045,2.141873,0.348458\H,0,-4.065316,3.262421,-0.456221\C,0,-2.432853,-0.765061,0.711602\O,0,-3.491904,-1.661213,0.513729\H,0,-3.17496,-2.294709,-0.157783\H,0,-0.565841,-1.79617,1.311839\H,0,-2.468189,-0.425198,1.754706\\Version=AM64L-G03RevD.01\State=2-A\HF=-905.5838807\MP2=-908.8223483\RMSD=8.555e-09\Thermal=0.\PG=C01 [X(C9H11N2O6)]\\@

026

1\1\GINC-NODE23\SP\ROMP2-FC\6-311+G(3df,2p)\C9H11N2O6(2)\ZIP06\22-Jan-2012\0\\#p ROMP2(FC)/6-311+G(3df,2p) scf=tight\\Rad1Uri_026\\0,2\O,0,-2.068793,3.088585,0.294291\C,0,-2.591716,1.852187,0.747731\C,0,-2.534695,0.74862,-0.311571\O,0,-1.199784,0.707114,-0.880708\C,0,-1.354691,-1.149435,0.60842\O,0,-1.311092,-2.551033,0.434701\C,0,-0.509965,-0.341068,-0.355418\N,0,0.865863,-0.041328,-0.150504\C,0,1.75013,-1.118766,0.004285\O,0,1.374307,-2.287545,0.069355\N,0,3.073131,-0.766159,0.086007\C,0,3.651724,0.526146,0.011939\O,0,4.860931,0.660056,0.089874\C,0,2.655748,1.570

247,-0.148717\C,0,1.341454,1.265211,-0.219473\H,0,-0.375105,-2.762649,0.227359\H,0,3.719189,-1.539635,0.201741\H,0,-1.979454,1.578281,1.614027\H,0,-3.632578,1.945949,1.097646\H,0,-3.215896,0.963295,-1.140373\H,0,2.994236,2.596574,-0.199834\H,0,0.564055,2.010628,-0.329804\H,0,-2.593182,3.379048,-0.469049\C,0,-2.78488,-0.660088,0.241003\O,0,-3.329119,-1.470528,-0.771183\H,0,-2.857302,-2.324051,-0.689129\H,0,-1.108435,-0.88906,1.652661\H,0,-3.438743,-0.637486,1.126393\\Version=AM64L-G03RevD.01\State=2-A\HF=-905.5837544\MP2=-908.821493\RMSD=1.109e-09\Thermal=0.\PG=C01 [X(C9H11N2O6)]\\@

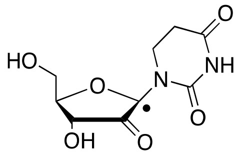

003

1\1\GINC-LIEBIG\SP\ROMP2-FC\6-311+G(3df,2p)\C9H11N2O6(2)\FLORIAN\23-Jan-2012\0\\#P ROMP2(FC)/6-311+G(3df,2p) scf=tight\\Rad1Ket2dU_003\\0,2\O,0,-2.115596,2.019358,1.015402\C,0,-2.846896,1.845217,-0.17795\C,0,-2.574076,0.48076,-0.81225\O,0,-1.127987,0.464635,-1.101434\C,0,-2.835315,-0.722295,0.103189\O,0,-3.492232,-1.750062,-0.630902\C,0,-0.530857,-0.341257,-0.214696\N,0,0.848411,-0.25337,-0.103878\C,0,1.415167,0.999238,0.117907 \O,0,0.781255,2.033744,0.26636\N,0,2.808618,0.998287,0.170032\C,0,3.691777,-0.041037,-0.134962\O,0,4.891666,0.133775,-0.120905\C,0,3.00442,-1.345559,-0.490955\C,0,1.641637,-1.467168,0.186792 \H,0,3.224361,1.902526,0.367392\H,0,-3.911499,1.909295,0.076264\H,0,-2.626631,2.630409,-0.916217\H,0,-3.083619,0.365483,-1.771805\H,0,-3.414758,-0.430259,0.989759\H,0,-3.440678,-2.551124,-0.081837\H,0,2.891713,-1.377923,-1.582765\H,0,1.746405,-1.592629,1.270137\H,0,-1.17573,2.148574,0.777136\C,0,-1.42812,-1.153782,0.53303\O,0,-1.160333,-2.057915,1.338756\H,0,3.665477,-2.166414,-0.201987\H,0,1.085153,-2.325346,-0.18799\\Version=AM64L-G03RevD.01\State=2-A\HF=-905.6040379\MP2=-908.8481619\RMSD=3.624e-09\Thermal=0.\PG=C01 [X(C9H11N2O6)]\\@

004

1\1\GINC-LIEBIG\SP\ROMP2-FC\6-311+G(3df,2p)\C9H11N2O6(2)\FLORIAN\23-Jan-2012\0\\#P ROMP2(FC)/6-311+G(3df,2p) scf=tight\\Rad1Ket2dU_004\\0,2\O,0,-1.993429,1.956306,1.186675\C,0,-2.866546,1.834063,0.085877\C,0,-2.653027,0.513081,-0.656469\O,0,-1.244927,0.538506,-1.091983\C,0,-2.807593,-0.749331,0.202393\O,0,-3.535264,-1.735236,-0.52136\C,0,-0.549922,-0.309042,-0.323369\N,0,0.833968,-0.209325,-0.322451\C,0,1.417972,1.031281,-0.116422\O,0,0.79518,2.064976,0.084511\N,0,2.814912,1.028747,-0.157458\C,0,3.670633,-0.05602,0.053506\O,0,4.873169,0.091272,0.104278\C,0,2.93802,-1.366611,0.261303\C,0,1.640937,-1.428961,-0.538737\H,0,3.236711,1.950311,-0.108699\H,0,-3.891812,1.860456,0.473312\H,0,-2.751181,2.664006,-0.626578\H,0,-3.258648,0.4494,-1.563515\H,0,-3.288691,-0.521448,1.163644\H,0,-3.414958,-2.570971,-0.03868\H,0,3.607878,-2.184604,-0.012672\H,0,1.034887,-2.275997,-0.2152\H,0,-1.094676,2.125252,0.839132\C,0,-1.360106,-1.191529,0.447328\O,0,-1.003695,-2.153868,1.142192\H,0,2.718495,-1.459152,1.333464\H,0,1.849363,-1.530448,-1.611808\\Version=AM64L-G03RevD.01\State=2-A\HF=-905.6025749\MP2=-908.8463553\RMSD=3.929e-09\Thermal=0.\PG=C01 [X(C9H11N2O6)]\\@

012

1\1\GINC-LIEBIG\SP\ROMP2-FC\6-311+G(3df,2p)\C9H11N2O6(2)\FLORIAN\23-Jan-2012\0\\#P
ROMP2(FC)/6-311+G(3df,2p) scf=tight\\Rad1Ket2dU_012\\0,2\O,0,-2.407629,2.805452,0.052948\C,0,-
2.918086,1.566973,0.481548\C,0,-2.602308,0.442234,-0.510205\O,0,-1.160998,0.554732,-0.791551\C,0,-
2.799763,-0.964934,0.05159\O,0,-3.27875,-1.835848,-0.965999\C,0,-0.518625,-0.36312,-0.062719\
N,0,0.849874,-0.231036,0.053429\C,0,1.41905,1.044285,0.168596\O,0,0.794705,2.082585,0.293643\N,0,
2.816012,1.041213,0.146716\C,0,3.686746,0.000913,-0.189304\O,0,4.882396,0.186804,-0.269553\C,0,
2.988025,-1.320167,-0.444166\C,0,1.684502,-1.418426,0.343254\H,0,3.235678,1.956822,0.270179\H,0,-
2.527664,1.283807,1.474551\H,0,-4.005108,1.66985,0.570848\H,0,-3.10109,0.59025,-1.468552\H,0,-
3.476169,-0.963135,0.919636\H,0,-3.187768,-2.739978,-0.619882\H,0,2.791747,-1.398712,-1.521657
\H,0,1.882594,-1.471152,1.420113\H,0,-1.437018,2.715889,0.024076\C,0,-1.377469,-1.357281,0.490654
\O,0,-1.079079,-2.37723,1.13683\H,0,3.675083,-2.125887,-0.173611\H,0,1.110926,-2.303819,0.077253\\
Version=AM64L-G03RevD.01\State=2-A\HF=-905.5998748\MP2=-908.8459416\RMSD=4.602e-09\
Thermal=0.\PG=C01 [X(C9H11N2O6)]\\@

006

1\1\GINC-LIEBIG\SP\ROMP2-FC\6-311+G(3df,2p)\C9H11N2O6(2)\FLORIAN\23-Jan-2012\0\\#P
ROMP2(FC)/6-311+G(3df,2p) scf=tight\\Rad1Ket2dU_006\\0,2\O,0,-2.475069,2.780811,0.276802\C,0,-
2.893865,1.513461,0.720762\C,0,-2.66946,0.433833,-0.342482\O,0,-1.273485,0.602039,-0.781478\C,0,-
2.767903,-1.000541,0.175525\O,0,-3.365357,-1.83471,-0.809113\C,0,-0.527402,-0.340845,-0.199247
\N,0,0.846526,-0.196113,-0.230662\C,0,1.425225,1.071956,-0.134803\O,0,0.809707,2.11468,0.000707
\N,0,2.823665,1.065023,-0.20397\C,0,3.69688,0.007644,0.063924\O,0,4.89737,0.175997,0.09923
\C,0,2.985133,-1.301369,0.335855\C,0,1.678879,-1.403007,-0.443049\H,0,3.238011,1.991133,-0.226929
\H,0,-2.376793,1.211285,1.648295\H,0,-3.964105,1.578853,0.945091\H,0,-3.279293,0.603893,-1.230433
\H,0,-3.321487,-1.051592,1.125259\H,0,-3.215898,-2.751623,-0.52193\H,0,3.658909,-2.121677,0.078211
\H,0,1.094402,-2.258919,-0.105397\H,0,-1.517684,2.718141,0.102512\C,0,-1.294939,-1.383053,0.402322
\O,0,-0.901241,-2.42925,0.946181\H,0,2.786273,-1.360981,1.414331\H,0,1.875638,-1.505897,-1.51806\\
Version=AM64L-G03RevD.01\State=2-A\HF=-905.598543\MP2=-908.8442775\RMSD=6.941e-09\
Thermal=0.\PG=C01 [X(C9H11N2O6)]\\@

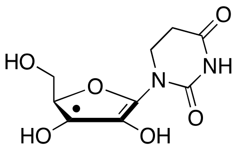

013

1\1\GINC-NODE24\SP\ROMP2-FC\6-311+G(3df,2p)\C9H11N2O6(2)\ZIP06\26-Jan-2012\0\\#p ROMP2
(FC)/6-311+G(3df,2p) scf=tight\\Rad3EndU_013\\0,2\O,0,4.563001,-1.376069,0.709404\C,0,3.165075,-
1.548001,0.842428\C,0,2.429004,-0.916516,-0.353956\O,0,1.004018,-1.182773,-0.256906\C,0,2.494457,
0.576948,-0.383714\O,0,3.688944,1.239242,-0.353874\C,0,1.222026,1.107303,-0.358956\O,0,0.966906,
2.438509,-0.386094\C,0,0.329082,0.02696,-0.316443\N,0,-1.060643,-0.065031,-0.221542\C,0,-1.804872,
0.926984,0.36104\O,0,-1.34243,1.964697,0.847914\N,0,-3.18907,0.723048,0.35661\C,0,-3.879776,-

0.471636,0.161803\O,0,-5.083948,-0.539404,0.299439\C,0,-2.970504,-1.629092,-0.200737\C,0,-1.740586,-1.14924,-0.965069\H,0,0.112737,2.560001,0.097603\H,0,-3.726674,1.506564,0.711473\H,0,2.975933,-2.625846,0.86126\H,0,2.77093,-1.113226,1.774013\H,0,2.805359,-1.389509,-1.274927\H,0,3.490083,2.188695,-0.266143\H,0,-2.665286,-2.124023,0.731032\H,0,-2.018986,-0.778774,-1.960896\H,0,4.73452,-0.420187,0.661473\H,0,-1.020148,-1.956091,-1.090972\H,0,-3.545843,-2.349597,-0.786834\\Version=AM64L-G03RevD.01\State=2-A\HF=-905.5755672\MP2=-908.830722\RMSD=1.681e-09\Thermal=0.\PG=C01 [X(C9H11N2O6)]\\@

015

1\1\GINC-NODE25\SP\ROMP2-FC\6-311+G(3df,2p)\C9H11N2O6(2)\ZIP06\26-Jan-2012\0\\#p ROMP2(FC)/6-311+G(3df,2p) scf=tight\\Rad3EndU_015\\0,2\O,0,3.149027,2.802278,-0.691096\C,0,3.277818,1.396581,-0.798368\C,0,2.588861,0.677246,0.365694\O,0,1.167314,1.011478,0.3223\C,0,2.591654,-0.815297,0.295927\O,0,3.747454,-1.529249,0.283924\C,0,1.294094,-1.286357,0.273432\O,0,0.980975,-2.605777,0.246442\C,0,0.444003,-0.173772,0.320422\N,0,-0.941402,-0.024442,0.235837\C,0,-1.722337,-0.945715,-0.412392\O,0,-1.298771,-1.9591,-0.978213\N,0,-3.099357,-0.695886,-0.380509\C,0,-3.74762,0.503279,-0.092069\O,0,-4.948879,0.624048,-0.218768\C,0,-2.797971,1.597099,0.356184\C,0,-1.582747,1.015407,1.071302\H,0,0.129225,-2.671059,-0.250628\H,0,-3.665106,-1.431377,-0.790257\H,0,2.865296,1.014497,-1.744733\H,0,4.348029,1.169213,-0.774574\H,0,2.991817,1.079552,1.308979\H,0,3.501479,-2.46736,0.196253\H,0,-2.480321,2.154866,-0.53508\H,0,-1.871133,0.572885,2.034294\H,0,2.196383,2.989604,-0.692907\H,0,-0.835279,1.784364,1.261955\H,0,-3.346568,2.286858,1.001896\\Version=AM64L-G03RevD.01\State=2-A\HF=-905.5765639\MP2=-908.8313908\RMSD=8.959e-09\Thermal=0.\PG=C01 [X(C9H11N2O6)]\\@

050

1\1\GINC-NODE17\SP\ROMP2-FC\6-311+G(3df,2p)\C9H11N2O6(2)\ZIP06\26-Jan-2012\0\\#p ROMP2(FC)/6-311+G(3df,2p) scf=tight\\Rad3EndU_050\\0,2\O,0,-3.277416,2.815654,0.010707\C,0,-3.363441,1.457921,0.403065\C,0,-2.577905,0.543536,-0.544045\O,0,-1.16753,0.92118,-0.486892\C,0,-2.558593,-0.903702,-0.176231\O,0,-3.69107,-1.653035,-0.156752\C,0,-1.279437,-1.279765,0.179364\O,0,-0.967626,-2.521702,0.625944\C,0,-0.45268,-0.157945,0.020984\N,0,0.93176,0.019449,0.07401\C,0,1.80033,-1.02104,-0.13339\O,0,1.466655,-2.182256,-0.391013\N,0,3.158331,-0.706578,-0.002189\C,0,3.755363,0.551641,-0.041414\O,0,4.961958,0.68446,-0.041589\C,0,2.747476,1.682265,-0.106616\C,0,1.447549,1.303599,0.595855\H,0,-0.048171,-2.698456,0.30872\H,0,3.778408,-1.506262,-0.072786\H,0,-2.999648,1.299768,1.430546\H,0,-4.42188,1.182008,0.36968\H,0,-2.920147,0.735272,-1.572204\H,0,-3.429299,-2.556126,0.09703\H,0,3.196864,2.571686,0.341347\H,0,0.679694,2.05584,0.421369\H,0,-2.332078,3.035386,-0.010118\H,0,1.600573,1.215084,1.679974\H,0,2.553299,1.902233,-1.164971\\Version=AM64L-G03RevD.01\State=2-A\HF=-905.5760939\MP2=-908.8306331\RMSD=6.113e-09\Thermal=0.\PG=C01 [X(C9H11N2O6)]\\@

009

1\1\GINC-NODE21\SP\ROMP2-FC\6-311+G(3df,2p)\C9H11N2O6(2)\ZIP06\26-Jan-2012\0\\#p ROMP2(FC)/6-311+G(3df,2p) scf=tight\\Rad3EndU_009\\0,2\O,0,4.671526,-1.208635,0.608708\C,0,3.294791,-

1.41796,0.857568\C,0,2.452995,-0.911964,-0.328687\O,0,1.048082,-1.216801,-0.107389\C,0,2.456149, 0.574688,-0.482073\O,0,3.623342,1.277283,-0.593982\C,0,1.170086,1.0644,-0.392775\O,0,0.864685, 2.380888,-0.502029\C,0,0.327662,-0.038581,-0.191937\N,0,-1.050354,-0.170493,-0.012122\C,0,-1.796124, 0.846813,0.521985\O,0,-1.340069,1.931042,0.905608\N,0,-3.172403,0.604927,0.610964\C,0,-3.917204,- 0.383437,-0.02563\O,0,-5.127765,-0.42507,0.060788\C,0,-3.064292,-1.336445,-0.840367\C,0,-1.670283,- 1.494825,-0.241983\H,0,0.044179,2.515309,0.033609\H,0,-3.693965,1.344742,1.068391\H,0,3.151527,- 2.496619,0.975667\H,0,2.950123,-0.921867,1.778099\H,0,2.78066,-1.44857,-1.23315\H,0,3.393925, 2.22319,-0.559095\H,0,-3.578625,-2.298974,-0.893748\H,0,-1.014945,-2.052114,-0.909517\H,0,4.799819,- 0.254743,0.470141\H,0,-1.715578,-2.039343,0.709223\H,0,-2.998908,-0.937839,-1.861751\\Version= AM64L-G03RevD.01\State=2-A\HF=-905.5733717\MP2=-908.8293879\RMSD=1.152e-09\Thermal= 0.\PG=C01 [X(C9H11N2O6)]\\@

077

1\1\GINC-NODE21\SP\ROMP2-FC\6-311+G(3df,2p)\C9H11N2O6(2)\ZIP06\26-Jan-2012\0\\#p ROMP2 (FC)/6-311+G(3df,2p) scf=tight\\Rad3EndU_077\\0,2\O,0,4.597661,-1.413273,0.320344\C,0,3.207541,- 1.67256,0.328208\C,0,2.473552,-0.708538,-0.625106\O,0,1.058002,-1.034278,-0.680922\C,0,2.474395, 0.712444,-0.161634\O,0,3.643935,1.374237,0.089812\C,0,1.189509,1.110221,0.145933\O,0,0.895124, 2.313019,0.697024\C,0,0.345063,0.033124,-0.162738\N,0,-1.042739,-0.118846,-0.182202\C,0,-1.879679, 0.961201,-0.277999\O,0,-1.509715,2.135964,-0.39566\N,0,-3.248869,0.670758,-0.222941\C,0,-3.866195,- 0.499559,0.207336\O,0,-5.073917,-0.587819,0.299441\C,0,-2.878157,-1.591374,0.570122\C,0,-1.599996,- 1.487914,-0.255597\H,0,-0.009418,2.546403,0.371774\H,0,-3.848175,1.476731,-0.363874\H,0,3.073482,- 2.703157,-0.01575\H,0,2.766021,-1.583987,1.333651\H,0,2.888553,-0.843088,-1.634874\H,0,3.409032, 2.249371,0.446874\H,0,-2.650661,-1.495963,1.640411\H,0,-1.79426,-1.738254,-1.305676\H,0,4.72245,- 0.484711,0.580974\H,0,-0.837085,-2.173006,0.110431\H,0,-3.363596,-2.558781,0.420611\\Version=AM64 L-G03RevD.01\State=2-A\HF=-905.5729932\MP2=-908.829325\RMSD=3.465e-09\Thermal=0.\PG=C01 [X(C9H11N2O6)]\\@

010

1\1\GINC-NODE23\SP\ROMP2-FC\6-311+G(3df,2p)\C9H11N2O6(2)\ZIP06\26-Jan-2012\0\\#p ROMP2 (FC)/6-311+G(3df,2p) scf=tight\\Rad3EndU_010\\0,2\O,0,3.380506,-2.650903,0.716889\C,0,3.431451,- 1.236261,0.739126\C,0,2.61263,-0.624089,-0.401465\O,0,1.218882,-1.031298,-0.222431\C,0,2.53232, 0.867438,-0.414841\O,0,3.640805,1.642771,-0.547658\C,0,1.21563,1.268666,-0.305883\O,0,0.829409, 2.568724,-0.329483\C,0,0.433656,0.109934,-0.214173\N,0,-0.933313,-0.106322,-0.034412\C,0,-1.721332, 0.818542,0.59858\O,0,-1.31184,1.877366,1.089723\N,0,-3.08617,0.508327,0.658939\C,0,-3.789008,- 0.43599,-0.082624\O,0,-4.996581,-0.53924,-0.005215\C,0,-2.897476,-1.258142,-0.993484\C,0,-1.497376,- 1.421498,-0.410357\H,0,0.018218,2.621181,0.232824\H,0,-3.638268,1.173239,1.189707\H,0,3.072054,- 0.824092,1.694447\H,0,4.480741,-0.950772,0.616229\H,0,2.960466,-1.061424,-1.351072\H,0,3.347192, 2.569533,-0.492392\H,0,-3.370988,-2.229748,-1.153438\H,0,-0.819641,-1.873955,-1.132942\H,0,2.444854, -2.892388,0.810688\H,0,-1.521111,-2.068459,0.475422\H,0,-2.849554,-0.748116,-1.965034\\Version= AM64L-G03RevD.01\State=2-A\HF=-905.5743457\MP2=-908.8300332\RMSD=5.049e-09\Thermal= 0.\PG=C01 [X(C9H11N2O6)]\\@

029

1\1\GINC-NODE27\SP\ROMP2-FC\6-311+G(3df,2p)\C9H11N2O6(2)\ZIP06\26-Jan-2012\0\\#p ROMP2 (FC)/6-311+G(3df,2p) scf=tight\\Rad3EndU_029\\0,2\O,0,3.343681,-2.766396,-0.060406\C,0,3.361273,-1.440122,0.435271\C,0,2.632413,-0.4737,-0.504985\O,0,1.23073,-0.883063,-0.589502\C,0,2.545558,0.942513,-0.039343\O,0,3.654203,1.709394,0.130613\C,0,1.23145,1.268977,0.227983\O,0,0.850902,2.469997,0.730695\C,0,0.451177,0.145115,-0.079566\N,0,-0.927826,-0.06577,-0.139111\C,0,-1.800527,0.971567,-0.341152\O,0,-1.469998,2.149441,-0.52041\N,0,-3.159354,0.630823,-0.319422\C,0,-3.749898,-0.533592,0.16043\O,0,-4.956104,-0.665904,0.207891\C,0,-2.738628,-1.557998,0.637907\C,0,-1.428502,-1.457371,-0.136951\H,0,-0.036269,2.657981,0.337391\H,0,-3.782064,1.401474,-0.536242\H,0,2.910829,-1.36608,1.437626\H,0,4.411212,-1.140848,0.510254\H,0,3.059369,-0.584864,-1.51324\H,0,3.349505,2.588215,0.419004\H,0,-2.563918,-1.381817,1.707835\H,0,-1.562533,-1.792517,-1.172795\H,0,2.409655,-3.003411,-0.177795\H,0,-0.6586,-2.08151,0.314784\H,0,-3.180138,-2.552077,0.534293\\Version=AM64L-G03RevD.01\State=2-A\HF=-905.5741291\MP2=-908.8292525\RMSD=4.495e-09\Thermal=0.\PG=C01 [X(C9H11N2O6)]\\@

052

1\1\GINC-NODE14\SP\ROMP2-FC\6-311+G(3df,2p)\C9H11N2O6(2)\ZIP06\26-Jan-2012\0\\#p ROMP2 (FC)/6-311+G(3df,2p) scf=tight\\Rad3EndU_052\\0,2\O,0,3.099946,-1.7933,1.265299\C,0,3.360149,-1.738531,-0.121112\C,0,2.502593,-0.694094,-0.847495\O,0,1.096288,-1.033666,-0.662372\C,0,2.583015,0.705016,-0.331228\O,0,3.751583,1.391662,-0.330628\C,0,1.352702,1.11289,0.13589\O,0,1.14137,2.326441,0.702734\C,0,0.462099,0.041548,-0.042871\N,0,-0.930477,-0.063855,0.027461\C,0,-1.745786,1.036423,-0.031869\O,0,-1.35882,2.200954,-0.176276\N,0,-3.114978,0.781275,0.119336\C,0,-3.784439,-0.428914,-0.04307\O,0,-4.996072,-0.497947,-0.009199\C,0,-2.84447,-1.593301,-0.283617\C,0,-1.503645,-1.373041,0.409333\H,0,0.213443,2.574602,0.469685\H,0,-3.689175,1.616277,0.164336\H,0,4.414216,-1.466922,-0.237446\H,0,3.203387,-2.715922,-0.606479\H,0,2.723735,-0.759447,-1.926933\H,0,3.567179,2.268847,0.050018\H,0,-3.327635,-2.507145,0.069892\H,0,-0.787938,-2.138969,0.113777\H,0,2.147709,-1.954052,1.36146\H,0,-1.618555,-1.409668,1.501283\H,0,-2.695835,-1.690672,-1.367402\\Version=AM64L-G03RevD.01\State=2-A\HF=-905.5749295\MP2=-908.829675\RMSD=2.625e-09\Thermal=0.\PG=C01 [X(C9H11N2O6)]\\@

064

1\1\GINC-NODE9\SP\ROMP2-FC\6-311+G(3df,2p)\C9H11N2O6(2)\ZIP06\26-Jan-2012\0\\#p ROMP2 (FC)/6-311+G(3df,2p) scf=tight\\Rad3EndU_064\\0,2\O,0,3.319754,-2.802872,0.101235\C,0,3.337969,-1.43142,0.45715\C,0,2.557452,-0.541145,-0.521535\O,0,1.15069,-0.899201,-0.51066\C,0,2.546347,0.911503,-0.166427\O,0,3.686434,1.653324,-0.133969\C,0,1.259654,1.302938,0.146551\O,0,0.944113,2.550065,0.574606\C,0,0.432524,0.187565,-0.040939\N,0,-0.945653,-0.013398,0.052451\C,0,-1.83375,1.015092,-0.117164\O,0,-1.523256,2.187509,-0.357731\N,0,-3.183642,0.674337,0.030023\C,0,-3.758766,-0.593581,-0.026595\O,0,-4.96319,-0.746794,-0.009231\C,0,-2.732861,-1.70463,-0.133788\C,0,-1.427091,-1.320157,0.554298\H,0,0.012717,2.708179,0.281981\H,0,-3.818881,1.463903,-0.011998\H,0,2.870559,-1.361739,1.443665\H,0,4.362446,-1.035263,0.537859\H,0,2.930414,-0.730313,-1.543482\H,0,3.426961,2.560422,0.106694\H,0,-3.158972,-2.611464,0.301872\H,0,-0.646712,-2.052253,0.351901\H,0,3.838997,-

2.907455,-0.711447\H,0,-1.564037,-1.255009,1.642186\H,0,-2.553654,-1.898807,-1.199867\\Version=AM64L-G03RevD.01\State=2-A\HF=-905.5740501\MP2=-908.8291477\RMSD=7.324e-09\Thermal=0.\PG=C01 [X(C9H11N2O6)]\\@

032

1\1\GINC-NODE12\SP\ROMP2-FC\6-311+G(3df,2p)\C9H11N2O6(2)\ZIP06\26-Jan-2012\0\\#p ROMP2(FC)/6-311+G(3df,2p) scf=tight\\Rad3EndU_032\\0,2\O,0,3.010539,-1.883761,1.226772\C,0,3.37955,-1.708944,-0.124727\C,0,2.574201,-0.608248,-0.826671\O,0,1.159951,-0.969719,-0.79418\C,0,2.592007,0.740237,-0.185297\O,0,3.749549,1.425748,-0.015261\C,0,1.319176,1.103966,0.197517\O,0,1.042379,2.263355,0.844801\C,0,0.462004,0.049387,-0.15597\N,0,-0.92962,-0.073633,-0.200062\C,0,-1.74828,1.0247,-0.229803\O,0,-1.360654,2.198251,-0.27165\N,0,-3.123307,0.754331,-0.193325\C,0,-3.762046,-0.430166,0.156808\O,0,-4.971336,-0.503903,0.24243\C,0,-2.794543,-1.562119,0.4445\C,0,-1.512407,-1.422453,-0.369984\H,0,0.142428,2.535136,0.539916\H,0,-3.707967,1.57833,-0.281846\H,0,4.436575,-1.42443,-0.131838\H,0,3.272506,-2.641582,-0.70268\H,0,2.885695,-0.578625,-1.88538\H,0,3.522435,2.264887,0.423398\H,0,-2.569114,-1.545441,1.519317\H,0,-1.708198,-1.595995,-1.435192\H,0,2.056166,-2.059136,1.232997\H,0,-0.764823,-2.148092,-0.05317\H,0,-3.296607,-2.507866,0.226644\\Version=AM64L-G03RevD.01\State=2-A\HF=-905.572716\MP2=-908.8280928\RMSD=5.892e-09\Thermal=0.\PG=C01 [X(C9H11N2O6)]\\@

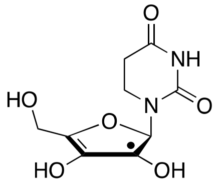

004

1\1\GINC-NODE14\SP\ROMP2-FC\6-311+G(3df,2p)\C9H11N2O6(2)\ZIP06\31-Jan-2012\0\\#p ROMP2(FC)/6-311+G(3df,2p) scf=tight\\Rad2EndU_004\\0,2\O,0,-2.658126,-2.699227,-0.420464\C,0,-3.275838,-1.644204,0.346013\C,0,-2.441395,-0.42798,0.440126\O,0,-1.294157,-0.51114,1.240148\C,0,-2.408559,0.755143,-0.305756\O,0,-3.38604,1.122111,-1.183133\C,0,-1.249587,1.438782,0.004176\O,0,-0.869239,2.645589,-0.474708\C,0,-0.460781,0.599461,0.964102\N,0,0.842828,0.100908,0.420947\C,0,1.821452,1.030604,0.278695\O,0,1.673087,2.23591,0.523053\N,0,3.053574,0.550128,-0.168631\C,0,3.442495,-0.784012,-0.310674\O,0,4.574953,-1.076579,-0.634101\C,0,2.336692,-1.779981,-0.0061\C,0,0.95363,-1.18705,-0.274217\H,0,0.048089,2.805501,-0.148886\H,0,3.769778,1.255172,-0.304387\H,0,-4.256424,-1.374585,-0.067992\H,0,-3.430047,-2.080191,1.337988\H,0,-3.138703,1.985099,-1.554001\H,0,-0.211116,1.116252,1.898678\H,0,2.513461,-2.67597,-0.606387\H,0,0.159619,-1.837293,0.09229\H,0,-2.591785,-2.376106,-1.333584\H,0,0.804021,-1.04768,-1.355041\H,0,2.425831,-2.065345,1.050567\\Version=AM64L-G03RevD.01\State=2-A\HF=-905.5786289\MP2=-908.8304122\RMSD=5.598e-09\Thermal=0.\PG=C01 [X(C9H11N2O6)]\\@

002

1\1\GINC-NODE27\SP\ROMP2-FC\6-311+G(3df,2p)\C9H11N2O6(2)\ZIP06\31-Jan-2012\0\\#p ROMP2 (FC)/6-311+G(3df,2p) scf=tight\\Rad2EndU_002\\0,2\O,0,-2.507204,-2.735465,-0.478363\C,0,-3.218182,-1.706115,0.241294\C,0,-2.438097,-0.459846,0.386209\O,0,-1.329611,-0.508618,1.242057\C,0,-2.413957,0.735189,-0.333655\O,0,-3.364515,1.099687,-1.239994\C,0,-1.287161,1.450873,0.028055\O,0,-0.959654,2.683015,-0.415801\C,0,-0.499046,0.606332,0.987577\N,0,0.806329,0.100456,0.416951\C,0,1.774627,1.036775,0.257088\O,0,1.580549,2.257549,0.352041\N,0,3.059976,0.565997,-0.020945\C,0,3.420637,-0.724426,-0.408498\O,0,4.566042,-1.000656,-0.699134\C,0,2.244755,-1.679813,-0.470234\C,0,1.155389,-1.323865,0.539692\H,0,-0.009944,2.821422,-0.17737\H,0,3.771399,1.285158,-0.094491\H,0,-4.177896,-1.474659,-0.239286\H,0,-3.423067,-2.14943,1.220947\H,0,-3.098742,1.958459,-1.608986\H,0,-0.264163,1.107072,1.936283\H,0,1.840461,-1.63506,-1.490739\H,0,1.494605,-1.544145,1.562685\H,0,-2.385318,-2.406232,-1.383647\H,0,0.253722,-1.908227,0.358225\H,0,2.616162,-2.694244,-0.30727\\Version=AM64L-G03RevD.01\State=2-A\HF=-905.5768597\MP2=-908.8297656\RMSD=3.647e-09\Thermal=0.\PG=C01 [X(C9H11N2O6)]\\@

003

1\1\GINC-NODE11\SP\ROMP2-FC\6-311+G(3df,2p)\C9H11N2O6(2)\ZIP06\31-Jan-2012\0\\#p ROMP2 (FC)/6-311+G(3df,2p) scf=tight\\Rad2EndU_003\\0,2\O,0,4.184328,-1.876367,-0.869581\C,0,3.399741,-1.59378,0.298151\C,0,2.368243,-0.551504,0.10373\O,0,1.266731,-0.855835,-0.710319\C,0,2.289273,0.778368,0.520509\O,0,3.221945,1.382832,1.309645\C,0,1.134594,1.336988,0.006911\O,0,0.72946,2.617647,0.164196\C,0,0.428972,0.285173,-0.796282\N,0,-0.942639,-0.073128,-0.305343\C,0,-1.898873,0.87449,-0.472915\O,0,-1.685264,1.99843,-0.948548\N,0,-3.18966,0.520629,-0.075022\C,0,-3.649746,-0.747412,0.282057\O,0,-4.818184,-0.943795,0.543932\C,0,-2.566229,-1.810619,0.273323\C,0,-1.186385,-1.222901,0.571372\H,0,-0.15315,2.693271,-0.271352\H,0,-3.882956,1.25581,-0.160696\H,0,2.935947,-2.553631,0.552995\H,0,4.03312,-1.288815,1.143779\H,0,2.923795,2.293347,1.47009\H,0,0.295366,0.561899,-1.850708\H,0,-2.83849,-2.579054,1.001027\H,0,-0.403038,-1.954552,0.379439\H,0,4.593046,-1.039344,-1.143268\H,0,-1.117977,-0.919275,1.625863\H,0,-2.569244,-2.27827,-0.720363\\Version=AM64L-G03RevD.01\State=2-A\HF=-905.5769308\MP2=-908.8291285\RMSD=7.016e-09\Thermal=0.\PG=C01 [X(C9H11N2O6)]\\@

001

1\1\GINC-NODE14\SP\ROMP2-FC\6-311+G(3df,2p)\C9H11N2O6(2)\ZIP06\30-Jan-2012\0\\#p ROMP2 (FC)/6-311+G(3df,2p) scf=tight\\Rad2EndU_001\\0,2\O,0,4.140322,-2.001602,-0.686917\C,0,3.350579,-1.60728,0.444078\C,0,2.359335,-0.547616,0.156674\O,0,1.271952,-0.867527,-0.666995\C,0,2.299195,0.800231,0.495617\O,0,3.226521,1.443244,1.258094\C,0,1.161585,1.357854,-0.061815\O,0,0.850354,2.670295,-0.009979\C,0,0.442803,0.275038,-0.818312\N,0,-0.929343,-0.075308,-0.293926\C,0,-1.876196,0.898568,-0.379587\O,0,-1.647836,2.083793,-0.654736\N,0,-3.195532,0.505259,-0.142442\C,0,-3.642532,-0.7042,0.390311\O,0,-4.814929,-0.889097,0.642069\C,0,-2.523368,-1.695773,0.635354\C,0,-1.361353,-1.483237,-0.331333\H,0,-0.100557,2.74146,-0.273125\H,0,-3.879569,1.249348,-0.228453\H,0,2.850066,-2.529576,0.761095\H,0,3.981782,-1.262268,1.275835\H,0,2.916931,2.356865,1.37767\H,0,0.331814,0.492408,-1.891976\H,0,-2.182063,-1.564003,1.670939\H,0,-1.658381,-1.762836,-1.353692\H,0,4.582942,-

1.201737,-1.013851\H,0,-0.507568,-2.098476,-0.055678\H,0,-2.932616,-2.705196,0.547605\\Version=AM64L-G03RevD.01\State=2-A\HF=-905.5754418\MP2=-908.8291756\RMSD=6.229e-09\Thermal=0.\PG=C01 [X(C9H11N2O6)]\\@

009

1\1\GINC-NODE14\SP\ROMP2-FC\6-311+G(3df,2p)\C9H11N2O6(2)\ZIP06\01-Feb-2012\0\\#p ROMP2(FC)/6-311+G(3df,2p) scf=tight\\Rad2EndU_009\\0,2\O,0,2.720018,2.764587,-0.193069\C,0,3.373161,1.554269,0.228695\C,0,2.452372,0.39854,0.296771\O,0,1.34869,0.536054,1.15398\C,0,2.366153,-0.807444,-0.397157\O,0,3.300172,-1.234861,-1.291948\C,0,1.211133,-1.457752,-0.002459\O,0,0.785755,-2.675184,-0.418256\C,0,0.478351,-0.565955,0.951835\N,0,-0.83664,-0.064858,0.439134\C,0,-1.834157,-0.987443,0.387437\O,0,-1.701049,-2.171237,0.723438\N,0,-3.066416,-0.522464,-0.075765\C,0,-3.428713,0.798189,-0.3477\O,0,-4.561188,1.084645,-0.676375\C,0,-2.294207,1.793059,-0.171746\C,0,-0.930006,1.140372,-0.393697\H,0,-0.107395,-2.816135,-0.028152\H,0,-3.79839,-1.222042,-0.133767\H,0,4.14845,1.340707,-0.511549\H,0,3.870982,1.722684,1.197636\H,0,2.992822,-2.080898,-1.656599\H,0,0.251829,-1.037546,1.915332\H,0,-2.460935,2.621411,-0.864934\H,0,-0.117301,1.80813,-0.111195\H,0,2.19293,3.069407,0.561483\H,0,-0.795634,0.88434,-1.454358\H,0,-2.359689,2.194155,0.848483\\Version=AM64L-G03RevD.01\State=2-A\HF=-905.5773879\MP2=-908.8298582\RMSD=4.861e-09\Thermal=0.\PG=C01 [X(C9H11N2O6)]\\@

014

1\1\GINC-NODE11\SP\ROMP2-FC\6-311+G(3df,2p)\C9H11N2O6(2)\ZIP06\01-Feb-2012\0\\#p ROMP2(FC)/6-311+G(3df,2p) scf=tight\\Rad2EndU_014\\0,2\O,0,2.59341,2.649153,-0.407124\C,0,3.296296,1.607275,0.298732\C,0,2.43612,0.415948,0.386885\O,0,1.314074,0.508882,1.220449\C,0,2.389552,-0.775552,-0.340393\O,0,3.350056,-1.162854,-1.224864\C,0,1.237233,-1.453645,0.006731\O,0,0.844977,-2.666275,-0.448246\C,0,0.467353,-0.596937,0.966137\N,0,-0.838316,-0.092721,0.431244\C,0,-1.822031,-1.018528,0.304095\O,0,-1.682311,-2.22092,0.568802\N,0,-3.052157,-0.539424,-0.151201\C,0,-3.432368,0.792924,-0.323658\O,0,-4.562773,1.0855,-0.655384\C,0,-2.321256,1.787871,-0.037077\C,0,-0.93955,1.184135,-0.287718\H,0,-0.070545,-2.8137,-0.112197\H,0,-3.771445,-1.243454,-0.274836\H,0,4.222825,1.32796,-0.221861\H,0,3.560591,1.953333,1.310685\H,0,3.067559,-2.007253,-1.612899\H,0,0.223469,-1.100382,1.909306\H,0,-2.490712,2.671852,-0.656955\H,0,-0.145442,1.837298,0.072091\H,0,3.103453,3.467705,-0.294255\H,0,-0.780154,1.026871,-1.363905\H,0,-2.413851,2.095944,1.013059\\Version=AM64L-G03RevD.01\State=2-A\HF=-905.5780587\MP2=-908.8295389\RMSD=6.014e-09\Thermal=0.\PG=C01 [X(C9H11N2O6)]\\@

013

1\1\GINC-NODE27\SP\ROMP2-FC\6-311+G(3df,2p)\C9H11N2O6(2)\ZIP06\01-Feb-2012\0\\#p ROMP2(FC)/6-311+G(3df,2p) scf=tight\\Rad2EndU_013\\0,2\O,0,2.453783,2.678842,-0.456963\C,0,3.238102,1.663118,0.200235\C,0,2.428433,0.44169,0.335663\O,0,1.348789,0.498752,1.227016\C,0,2.388381,-0.758464,-0.370728\O,0,3.319116,-1.138931,-1.288446\C,0,1.269735,-1.468483,0.027627\O,0,0.925677,-2.702944,-0.398037\C,0,0.503879,-0.609756,0.992056\N,0,-0.804218,-0.097252,0.43483\C,0,-1.781085,-1.024725,0.287234\O,0,-1.601405,-2.24638,0.402182\N,0,-3.060867,-0.543211,-0.001109\C,0,-3.402085,

0.741485,-0.423534\O,0,-4.542953,1.025666,-0.725419\C,0,-2.212931,1.678568,-0.504354\C,0,-1.134068,
1.3337,0.520571\H,0,-0.021633,-2.830296,-0.145035\H,0,-3.780387,-1.255214,-0.063766\H,0,4.140349,
1.419345,-0.377583\H,0,3.551415,2.018749,1.194839\H,0,3.017195,-1.977202,-1.676197\H,0,0.277028,-
1.101005,1.947451\H,0,-1.801544,1.601959,-1.519989\H,0,-1.477051,1.581934,1.536128\H,0,2.931889,
3.518599,-0.361412\H,0,-0.224508,1.901662,0.328251\H,0,-2.571388,2.701864,-0.370002\\Version=AM64
L-G03RevD.01\State=2-A\HF=-905.5760925\MP2=-908.828692\RMSD=3.715e-09\Thermal=0.\PG=C01
[X(C9H11N2O6)]\\@

026

1\1\GINC-NODE12\SP\ROMP2-FC\6-311+G(3df,2p)\C9H11N2O6(2)\ZIP06\01-Feb-2012\0\\#p ROMP2
(FC)/6-311+G(3df,2p) scf=tight\\Rad2EndU_026\\0,2\O,0,-4.584502,-0.970685,-0.740125\C,0,-3.25002,-
1.489685,-0.928627\C,0,-2.282751,-0.664225,-0.159603\O,0,-1.085459,-1.200999,0.270939\C,0,-2.329593,
0.714272,0.074219\O,0,-3.35331,1.561103,-0.233481\C,0,-1.155606,1.086191,0.692324\O,0,-0.772096,
2.292408,1.191433\C,0,-0.304542,-0.133478,0.89624\N,0,1.018953,-0.068665,0.319535\C,0,2.051567,-
0.682311,1.00406\O,0,1.954559,-1.194326,2.106682\N,0,3.286672,-0.665323,0.332727\C,0,3.654184,
0.081431,-0.782049\O,0,4.794601,0.07929,-1.201563\C,0,2.515023,0.893321,-1.369901\C,0,1.164812,
0.231722,-1.107376\H,0,-1.507823,2.914177,1.058203\H,0,4.033389,-1.142882,0.825578\H,0,-3.175407,-
2.547281,-0.639427\H,0,-3.09618,-1.438993,-2.016202\H,0,-4.082589,0.997871,-0.564618\H,0,-0.157596,-
0.39669,1.948562\H,0,2.541805,1.888729,-0.907024\H,0,1.064028,-0.687464,-1.700829\H,0,-4.815924,-
1.124811,0.191592\H,0,0.347239,0.899464,-1.38892\H,0,2.700898,1.01803,-2.439548\\Version=AM64L-
G03RevD.01\State=2-A\HF=-905.5771559\MP2=-908.8284112\RMSD=9.274e-09\Thermal=0.\PG=C01
[X(C9H11N2O6)]\\@

011

1\1\GINC-NODE15\SP\ROMP2-FC\6-311+G(3df,2p)\C9H11N2O6(2)\ZIP06\01-Feb-2012\0\\#p ROMP2
(FC)/6-311+G(3df,2p) scf=tight\\Rad2EndU_011\\0,2\O,0,4.215267,-1.74929,-0.912781\C,0,3.42922,-
1.577462,0.275752\C,0,2.374336,-0.548242,0.152448\O,0,1.28674,-0.864969,-0.684792\C,0,2.281573,
0.78335,0.555783\O,0,3.190978,1.394272,1.363233\C,0,1.131216,1.329668,0.019372\O,0,0.701099,2.6031
03,0.17976\C,0,0.443993,0.272662,-0.791474\N,0,-0.932813,-0.087878,-0.321696\C,0,-1.890343,0.853212,
-0.527255\O,0,-1.674572,1.959621,-1.039569\N,0,-3.182528,0.510169,-0.12472\C,0,-3.637802,-0.74034,
0.294281\O,0,-4.806817,-0.930381,0.558134\C,0,-2.548577,-1.796591,0.349332\C,0,-1.17288,-1.186356,
0.619543\H,0,-0.156336,2.679919,-0.300512\H,0,-3.878254,1.238224,-0.244987\H,0,2.984159,-2.540822,
0.575553\H,0,4.128486,-1.271006,1.057535\H,0,2.90378,2.313866,1.486992\H,0,0.325081,0.541633,-
1.849458\H,0,-2.820637,-2.524514,1.117638\H,0,-0.385339,-1.923998,0.47217\H,0,3.595126,-1.965379,-
1.627197\H,0,-1.109366,-0.823486,1.655449\H,0,-2.545288,-2.318719,-0.61684\\Version=AM64L-
G03RevD.01\State=2-A\HF=-905.5772541\MP2=-908.8284132\RMSD=7.275e-09\Thermal=0.\PG=C01
[X(C9H11N2O6)]\\@

005

1\1\GINC-NODE11\SP\ROMP2-FC\6-311+G(3df,2p)\C9H11N2O6(2)\ZIP06\31-Jan-2012\0\\#p ROMP2
(FC)/6-311+G(3df,2p) scf=tight\\Rad2EndU_005\\0,2\O,0,-2.596316,2.792232,0.2642\C,0,-3.316587,

1.611919,-0.13565\C,0,-2.444506,0.423598,-0.254441\O,0,-1.37271,0.529571,-1.156893\C,0,-2.373844,-0.794606,0.412743\O,0,-3.288194,-1.218209,1.328724\C,0,-1.250071,-1.475588,-0.025358\O,0,-0.876834,-2.71661,0.358007\C,0,-0.509686,-0.576428,-0.969565\N,0,0.804443,-0.075294,-0.411253\C,0,1.796449,-0.997098,-0.350469\O,0,1.623611,-2.209673,-0.540543\N,0,3.078701,-0.519176,-0.067212\C,0,3.416452,0.737953,0.432212\O,0,4.562503,1.021902,0.712634\C,0,2.216417,1.644639,0.626952\C,0,1.104667,1.361917,-0.382446\H,0,0.057893,-2.832595,0.060948\H,0,3.80859,-1.22338,-0.076769\H,0,-4.064056,1.42038,0.638551\H,0,-3.85281,1.810159,-1.077989\H,0,-2.966432,-2.059485,1.692976\H,0,-0.287138,-1.030834,-1.943711\H,0,1.844897,1.481241,1.647634\H,0,1.398556,1.709469,-1.384158\H,0,-2.121626,3.104093,-0.521402\H,0,0.193803,1.889168,-0.100661\H,0,2.554095,2.681527,0.560928\\Version=AM64L-G03RevD.01\State=2-A\HF=-905.5749828\MP2=-908.8286255\RMSD=1.715e-09\Thermal=0.\PG=C01 [X(C9H11N2O6)]\\@

032

1\1\GINC-NODE27\SP\ROMP2-FC\6-311+G(3df,2p)\C9H11N2O6(2)\ZIP06\01-Feb-2012\0\\#p ROMP2(FC)/6-311+G(3df,2p) scf=tight\\Rad2EndU_032\\0,2\O,0,-4.273899,-1.528824,-0.563263\C,0,-3.461761,-1.454318,0.625686\C,0,-2.324151,-0.526612,0.397954\O,0,-1.167388,-0.662549,1.173383\C,0,-2.310651,0.674309,-0.319792\O,0,-3.297576,1.115922,-1.141629\C,0,-1.116178,1.319349,-0.050821\O,0,-0.670527,2.496165,-0.536067\C,0,-0.33873,0.465391,0.914514\N,0,0.985024,-0.006364,0.406779\C,0,1.965805,0.934596,0.351231\O,0,1.808898,2.116523,0.678271\N,0,3.209563,0.492904,-0.106769\C,0,3.607451,-0.820215,-0.354402\O,0,4.742884,-1.083929,-0.692391\C,0,2.506234,-1.841958,-0.132762\C,0,1.117527,-1.245492,-0.364708\H,0,0.201748,2.671745,-0.117266\H,0,3.920195,1.212659,-0.181248\H,0,-4.15782,-1.103636,1.401511\H,0,-3.101263,-2.444706,0.940837\H,0,-3.985545,0.421004,-1.142731\H,0,-0.115535,0.974792,1.860902\H,0,2.693494,-2.690518,-0.795507\H,0,0.341996,-1.929258,-0.021801\H,0,-3.718961,-1.920767,-1.258859\H,0,0.954968,-1.051736,-1.434864\H,0,2.592192,-2.202116,0.901113\\Version=AM64L-G03RevD.01\State=2-A\HF=-905.5729715\MP2=-908.8266122\RMSD=8.075e-09\Thermal=0.\PG=C01 [X(C9H11N2O6)]\\@

008

1\1\GINC-NODE27\SP\ROMP2-FC\6-311+G(3df,2p)\C9H11N2O6(2)\ZIP06\31-Jan-2012\0\\#p ROMP2(FC)/6-311+G(3df,2p) scf=tight\\Rad2EndU_008\\0,2\O,0,4.194282,-1.853595,-0.758887\C,0,3.387775,-1.598607,0.400812\C,0,2.366718,-0.547417,0.202342\O,0,1.286502,-0.875688,-0.638631\C,0,2.299037,0.801455,0.530447\O,0,3.210441,1.447986,1.305668\C,0,1.163546,1.349523,-0.040877\O,0,0.826388,2.655493,0.023744\C,0,0.454439,0.263381,-0.799148\N,0,-0.922077,-0.081496,-0.277929\C,0,-1.87284,0.879456,-0.432619\O,0,-1.639913,2.049445,-0.763196\N,0,-3.195213,0.490536,-0.205471\C,0,-3.644932,-0.690239,0.385521\O,0,-4.821701,-0.874417,0.616242\C,0,-2.522637,-1.65127,0.72239\C,0,-1.338028,-1.492349,-0.22756\H,0,-0.107466,2.721613,-0.292891\H,0,-3.881929,1.22339,-0.348216\H,0,2.907951,-2.532183,0.738752\H,0,4.078837,-1.272582,1.181985\H,0,2.908949,2.366856,1.401823\H,0,0.344958,0.475364,-1.873728\H,0,-2.208053,-1.446239,1.754517\H,0,-1.605671,-1.847665,-1.23453\H,0,3.583892,-2.082882,-1.477485\H,0,-0.486478,-2.077745,0.113042\H,0,-2.920419,-2.668588,0.693178\\Version=AM64L-G03RevD.01\State=2-A\HF=-905.5751469\MP2=-908.8277617\RMSD=6.551e-09\Thermal=0.\PG=C01 [X(C9H11N2O6)]\\@

034

1\1\GINC-NODE11\SP\ROMP2-FC\6-311+G(3df,2p)\C9H11N2O6(2)\ZIP06\01-Feb-2012\0\\#p ROMP2 (FC)/6-311+G(3df,2p) scf=tight\\Rad2EndU_034\\0,2\O,0,-4.283093,-0.807617,-1.396277\C,0,-3.407556,-1.562937,-0.530485\C,0,-2.30639,-0.689786,-0.048518\O,0,-1.097171,-1.234937,0.338193\C,0,-2.356443,0.678674,0.237602\O,0,-3.394558,1.528785,-0.005408\C,0,-1.155289,1.043208,0.805832\O,0,-0.718055,2.260278,1.229556\C,0,-0.295176,-0.178002,0.955549\N,0,1.005827,-0.089428,0.333656\C,0,2.067359,-0.71076,0.965142\O,0,2.013137,-1.256032,2.054397\N,0,3.279029,-0.660317,0.253399\C,0,3.602607,0.127188,-0.846424\O,0,4.728255,0.151507,-1.303489\C,0,2.436665,0.946047,-1.368707\C,0,1.102945,0.259593,-1.085889\H,0,-1.449391,2.891326,1.118241\H,0,4.046151,-1.145318,0.706061\H,0,-4.066513,-1.910872,0.277584\H,0,-3.007722,-2.457323,-1.028905\H,0,-4.070936,1.005019,-0.481261\H,0,-0.108115,-0.469026,1.994262\H,0,2.466747,1.924845,-0.871917\H,0,0.993642,-0.640677,-1.706524\H,0,-3.775254,-0.601759,-2.199195\H,0,0.270185,0.926767,-1.319403\H,0,2.586774,1.110011,-2.438714\\Version=AM64L-G03RevD.01\State=2-A\HF=-905.5767216\MP2=-908.8280655\RMSD=7.835e-09\Thermal=0.\PG=C01 [X(C9H11N2O6)]\\@

020

1\1\GINC-NODE14\SP\ROMP2-FC\6-311+G(3df,2p)\C9H11N2O6(2)\ZIP06\01-Feb-2012\0\\#p ROMP2 (FC)/6-311+G(3df,2p) scf=tight\\Rad2EndU_020\\0,2\O,0,4.587868,-1.232116,0.155699\C,0,3.2583,-1.744466,-0.065943\C,0,2.303041,-0.619862,-0.225637\O,0,1.167178,-0.78449,-1.024976\C,0,2.298199,0.625526,0.396451\O,0,3.263047,1.103479,1.225152\C,0,1.148611,1.300799,0.010091\O,0,0.819558,2.574989,0.285416\C,0,0.362522,0.378812,-0.88765\N,0,-0.982177,-0.039248,-0.337992\C,0,-1.933108,0.932919,-0.326893\O,0,-1.705982,2.129835,-0.541764\N,0,-3.243081,0.521496,-0.06392\C,0,-3.660029,-0.712977,0.431538\O,0,-4.82183,-0.925478,0.71037\C,0,-2.521457,-1.698424,0.609048\C,0,-1.394365,-1.449295,-0.390616\H,0,-0.111545,2.698307,-0.014562\H,0,-3.932867,1.264885,-0.08722\H,0,3.214461,-2.413007,-0.938412\H,0,3.06342,-2.360479,0.824067\H,0,3.99093,0.451181,1.202821\H,0,0.176519,0.804164,-1.884675\H,0,-2.145663,-1.588451,1.635232\H,0,-1.719997,-1.715304,-1.407775\H,0,4.842393,-0.753788,-0.651839\H,0,-0.526217,-2.061674,-0.155043\H,0,-2.923618,-2.709514,0.50929\\Version=AM64L-G03RevD.01\State=2-A\HF=-905.5698433\MP2=-908.8264528\RMSD=6.211e-09\Thermal=0.\PG=C01 [X(C9H11N2O6)]\\@

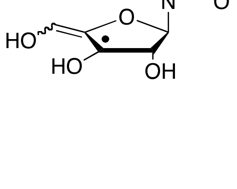

016

1\1\GINC-NODE17\SP\ROMP2-FC\6-311+G(3df,2p)\C9H11N2O6(2)\ZIP06\04-Feb-2012\0\\#p ROMP2 (FC)/6-311+G(3df,2p) scf=tight\\Rad3_2_EndU_016\\0,2\O,0,4.644801,-1.502826,0.221184\C,0,3.373608,-1.731423,-0.207844\H,0,3.214406,-2.721051,-0.614896\C,0,2.37844,-0.777234,-0.124829\O,0,1.081475,-1.080409,-0.562364\C,0,0.355904,0.149664,-0.646776\H,0,0.390236,0.515039,-1.684444\N,0,-1.030722,-0.123537,-0.244548\C,0,-1.604644,-1.458661,-0.483464\H,0,-0.82302,-2.19147,-0.294942\C,0,-2.794893,-

1.682579,0.4459\H,0,-2.452955,-1.738991,1.488051\C,0,-3.799974,-0.552037,0.351116\O,0,-4.988117,-0.648599,0.580038\N,0,-3.2252,0.666046,-0.007212\H,0,-3.831405,1.479102,0.003025\C,0,-1.869913,0.956558,-0.202607\O,0,-1.512167,2.129146,-0.317782\C,0,2.426883,0.522905,0.338424\C,0,1.096814,1.184974,0.251515\H,0,0.598921,1.262285,1.234051\O,0,1.237465,2.467334,-0.327689\H,0,0.325656,2.815692,-0.377576\H,0,4.706584,-0.583851,0.543951\O,0,3.540946,1.110703,0.876939\H,0,3.478744,2.065389,0.693734\H,0,-1.915483,-1.556633,-1.53476\H,0,-3.308801,-2.618865,0.214915\\Version=AM64L-G03RevD.01\State=2-A\HF=-905.5770904\MP2=-908.8298603\RMSD=4.700e-09\Thermal=0.\PG=C01 [X(C9H11N2O6)]\\@

007

1\1\GINC-NODE9\SP\ROMP2-FC\6-311+G(3df,2p)\C9H11N2O6(2)\ZIP06\03-Feb-2012\0\\#p ROMP2(FC)/6-311+G(3df,2p) scf=tight\\Rad3_2_EndU_003\\0,2\O,0,4.637114,-1.504687,0.062439\C,0,3.367913,-1.691544,-0.389008\H,0,3.209948,-2.636771,-0.891134\C,0,2.371831,-0.748865,-0.220577\O,0,1.07898,-1.012625,-0.69477\C,0,0.341689,0.207913,-0.630411\H,0,0.325252,0.680969,-1.619496\N,0,-1.041106,-0.105752,-0.255635\C,0,-1.406793,-1.340801,0.449196\H,0,-1.33329,-1.196691,1.537417\C,0,-2.829345,-1.745068,0.064049\H,0,-3.181835,-2.585166,0.667437\C,0,-3.808763,-0.597806,0.233342\O,0,-4.994848,-0.721547,0.460253\N,0,-3.225937,0.658901,0.077173\H,0,-3.847819,1.459636,0.097713\C,0,-1.903662,0.950408,-0.276429\O,0,-1.580907,2.100849,-0.578559\C,0,2.422172,0.501423,0.367623\C,0,1.096474,1.172663,0.334127\H,0,0.605367,1.195077,1.325151\O,0,1.219147,2.490671,-0.172689\H,0,0.301855,2.797809,-0.314865\H,0,4.696655,-0.620657,0.472551\O,0,3.542625,1.017901,0.967698\H,0,3.51729,1.984197,0.85149\H,0,-2.859864,-2.055635,-0.988891\H,0,-0.690239,-2.109248,0.165402\\Version=AM64L-G03RevD.01\State=2-A\HF=-905.5763901\MP2=-908.8287356\RMSD=4.572e-09\Thermal=0.\PG=C01 [X(C9H11N2O6)]\\@

012

1\1\GINC-NODE11\SP\ROMP2-FC\6-311+G(3df,2p)\C9H11N2O6(2)\ZIP06\03-Feb-2012\0\\#p ROMP2(FC)/6-311+G(3df,2p) scf=tight\\Rad3_2_EndU_012\\0,2\O,0,-3.302322,2.811167,-0.35395\C,0,-3.512588,1.5433,0.103612\H,0,-4.513592,1.359459,0.467157\C,0,-2.53143,0.577436,0.101654\O,0,-1.251291,0.966361,-0.352705\C,0,-0.482313,-0.218858,-0.575795\H,0,-0.544113,-0.493518,-1.639549\N,0,0.90905,0.073432,-0.200238\C,0,1.42745,1.444008,-0.342208\H,0,0.632706,2.12844,-0.053353\C,0,2.648753,1.632747,0.554576\H,0,2.349996,1.5876,1.610383\C,0,3.686973,0.553489,0.322545\O,0,4.879829,0.676227,0.509585\N,0,3.138353,-0.651211,-0.11275\H,0,3.771067,-1.439702,-0.194765\C,0,1.786795,-0.974655,-0.27946\O,0,1.465367,-2.14601,-0.478542\C,0,-2.514317,-0.763243,0.444566\C,0,-1.152645,-1.344872,0.260269\H,0,-0.616372,-1.471825,1.21777\O,0,-1.262605,-2.587616,-0.404663\H,0,-0.341648,-2.898576,-0.503625\H,0,-2.398149,2.845533,-0.710731\O,0,-3.560771,-1.483202,0.923362\H,0,-3.364522,-2.416059,0.721808\H,0,1.687794,1.647296,-1.392307\H,0,3.119274,2.604274,0.384878\\Version=AM64L-G03RevD.01\State=2-A\HF=-905.5766668\MP2=-908.8285703\RMSD=8.088e-09\Thermal=0.\PG=C01 [X(C9H11N2O6)]\\@

011

1\1\GINC-NODE18\SP\ROMP2-FC\6-311+G(3df,2p)\C9H11N2O6(2)\ZIP06\03-Feb-2012\0\\#p ROMP2 (FC)/6-311+G(3df,2p) scf=tight\\Rad3_2_EndU_011\\0,2\O,0,3.304924,-2.754308,-0.604532\C,0, 3.506846,-1.530779,-0.038062\H,0,4.502931,-1.376626,0.351854\C,0,2.522346,-0.570361,0.02882\O,0, 1.253647,-0.925649,-0.480434\C,0,0.468454,0.262911,-0.570359\H,0,0.48379,0.640804,-1.59888\N,0,- 0.919535,-0.070965,-0.234112\C,0,-1.262832,-1.198483,0.641612\H,0,-1.235539,-0.888645,1.696959\C,0,- 2.653264,-1.718569,0.27839\H,0,-2.99614,-2.47237,0.991357\C,0,-3.681975,-0.602687,0.240137\O,0,- 4.870666,-0.744423,0.440312\N,0,-3.141944,0.642689,-0.077508\H,0,-3.793465,1.412272,-0.18588\C,0,- 1.817829,0.941899,-0.41514\O,0,-1.52265,2.05829,-0.843793\C,0,2.499734,0.735815,0.491562\C,0, 1.141668,1.329495,0.337718\H,0,0.603066,1.409777,1.30179\O,0,1.233444,2.608586,-0.266908 \H,0,0.315167,2.862486,-0.485122\H,0,2.406389,-2.758797,-0.977718\O,0,3.546453,1.39318,1.056576 \H,0,3.384888,2.343713,0.922074\H,0,-2.632837,-2.185374,-0.715454\H,0,-0.507991,-1.970151,0.502532\\ Version=AM64L-G03RevD.01\State=2-A\HF=-905.576559\MP2=-908.8280772\RMSD=3.803e-09\ Thermal=0.\PG=C01 [X(C9H11N2O6)]\\@

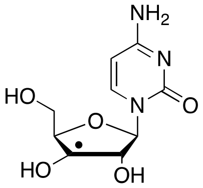

007

1\1\GINC-NODE22\SP\ROMP2-FC\6-311+G(3df,2p)\C9H12N3O5(2)\ZIP06\15-Mar-2013\0\\#p ROMP2 (FC)/6-311+G(3df,2p) scf=tight\\Rad3Cy_007\\0,2\O,0,2.096544,-2.223216,1.248379\C,0,2.821556,- 2.101873,0.033468\H,0,2.485671,-2.922682,-0.607774\H,0,3.905377,-2.214934,0.191592\C,0,2.566961,- 0.769519,-0.670675\H,0,3.226035,-0.677251,-1.544132\O,0,1.178579,-0.76112,-1.127787\C,0,0.506833, 0.388912,-0.66728\H,0,0.475826,1.173555,-1.433846\N,0,-0.890564,0.035403,-0.361444\C,0,-1.317103,- 1.251834,-0.284901\H,0,-0.553892,-2.005166,-0.428105\C,0,-2.627706,-1.531414,-0.042336\C,0,- 3.505992,-0.410423,0.099057\N,0,-3.091892,0.848683,0.057614\C,0,-1.776008,1.124212,-0.115608\O,0,- 1.298451,2.267379,-0.080333\C,0,2.69662,0.443678,0.214815\C,0,1.302817,0.91686,0.559685\H,0, 0.912549,0.41492,1.459716\O,0,1.305324,2.315595,0.695198\H,0,0.385808,2.602661,0.480152\H,0,2.4609 23,-1.557496,1.855497\O,0,3.579335,1.405632,-0.162968\H,0,3.175313,2.25293,0.128684\H,0,-2.97721,- 2.554152,0.029731\N,0,-4.833631,-0.610909,0.318044\H,0,-5.431508,0.202858,0.280944\H,0,-5.250808,- 1.513244,0.150774\\Version=AM64L-G03RevD.01\State=2-A\HF=-885.7308364\MP2=-888.9455584 \RMSD=4.674e-09\Thermal=0.\PG=C01 [X(C9H12N3O5)]\\@

016

1\1\GINC-NODE16\SP\ROMP2-FC\6-311+G(3df,2p)\C9H12N3O5(2)\ZIP06\15-Mar-2013\0\\#p ROMP2 (FC)/6-311+G(3df,2p) scf=tight\\Rad3Cy_016\\0,2\O,0,-4.269333,-1.846802,-0.639561\C,0,-2.898629,- 1.912752,-0.298075\H,0,-2.249441,-1.9759,-1.186638\H,0,-2.763865,-2.829776,0.28362\C,0,-2.484561,- 0.696641,0.538281\H,0,-3.096054,-0.649252,1.447015\O,0,-1.078196,-0.864147,0.906571\C,0,-0.353519, 0.310348,0.606036\H,0,-0.314115,0.995324,1.463574\N,0,1.036889,-0.068922,0.289698\C,0,1.437939,-

1.365536,0.213658\H,0,0.661275,-2.103579,0.362389\C,0,2.741697,-1.678627,-0.021916\C,0,3.649861,-0.58032,-0.154934\N,0,3.269075,0.687902,-0.088341\C,0,1.965825,0.999346,0.113799\O,0,1.536002,2.160394,0.152608\C,0,-2.542786,0.609725,-0.204406\C,0,-1.124039,1.006102,-0.553973\H,0,-0.791881,0.556997,-1.505037\O,0,-1.033691,2.406994,-0.57363\H,0,-0.09612,2.614853,-0.342818\H,0,-4.398387,-1.013402,-1.123655\O,0,-3.324919,1.597295,0.30542\H,0,-2.862045,2.433726,0.073808\H,0,3.067108,-2.710451,-0.075227\N,0,4.97639,-0.812404,-0.333404\H,0,5.314974,-1.729464,-0.578642\H,0,5.562447,-0.014848,-0.536139\\Version=AM64L-G03RevD.01\State=2-A\HF=-885.7292998\MP2=-888.9432443 \RMSD=6.856e-09\Thermal=0.\PG=C01 [X(C9H12N3O5)]\\@

029

1\1\GINC-NODE12\SP\ROMP2-FC\6-311+G(3df,2p)\C9H12N3O5(2)\ZIP06\15-Mar-2013\0\\#p ROMP2 (FC)/6-311+G(3df,2p) scf=tight\\Rad3Cy_029\\0,2\O,0,-3.054492,2.943221,-0.169457\C,0,-3.142524,1.714988,0.532781\H,0,-4.200788,1.556304,0.76158\H,0,-2.590701,1.746079,1.4864\C,0,-2.630276,0.543421,-0.299794\H,0,-3.19695,0.485298,-1.238768\O,0,-1.22703,0.842416,-0.617557\C,0,-0.457321,-0.338,-0.500403\H,0,-0.455171,-0.917036,-1.433504\N,0,0.939949,0.038449,-0.221788\C,0,1.317595,1.31817,0.035469\H,0,0.517891,2.045384,0.063172\C,0,2.627001,1.633378,0.232453\C,0,3.566422,0.558124,0.125897\N,0,3.209296,-0.695378,-0.115019\C,0,1.900733,-1.017156,-0.259275\O,0,1.49093,-2.172771,-0.429671\C,0,-2.592938,-0.793503,0.384581\C,0,-1.14676,-1.179357,0.609479\H,0,-0.771798,-0.830892,1.587279\O,0,-1.018834,-2.571429,0.469327\H,0,-0.097132,-2.726576,0.153275\H,0,-2.138873,3.01627,-0.482811\O,0,-3.416442,-1.766327,-0.086341\H,0,-2.921593,-2.605663,0.040211\H,0,2.930995,2.650886,0.445964\N,0,4.892908,0.795237,0.298178\H,0,5.25668,1.73522,0.29305\H,0,5.527718,0.035311,0.097017 \\Version=AM64L-G03RevD.01\State=2-A\HF=-885.7300836\MP2=-888.9434718\RMSD=6.565e-09\ Thermal=0.\PG=C01 [X(C9H12N3O5)]\\@

006

1\1\GINC-NODE18\SP\ROMP2-FC\6-311+G(3df,2p)\C9H12N3O5(2)\ZIP06\15-Mar-2013\0\\#p ROMP2 (FC)/6-311+G(3df,2p) scf=tight\\Rad3Cy_006\\0,2\O,0,2.305603,-2.237822,1.29682\C,0,3.024207,-2.028824,0.094047\H,0,2.904944,-2.877523,-0.599249\H,0,4.081665,-1.959377,0.365601\C,0,2.610635,-0.740686,-0.617167\H,0,3.168642,-0.63938,-1.558197\O,0,1.177082,-0.878251,-0.922689\C,0,0.505759,0.328178,-0.609989\H,0,0.514391,1.028519,-1.456398\N,0,-0.906595,0.001889,-0.323351\C,0,-1.38427,-1.270287,-0.382879\H,0,-0.655653,-2.033576,-0.619474\C,0,-2.702714,-1.535988,-0.174076\C,0,-3.549319,-0.409661,0.078379\N,0,-3.10122,0.836364,0.121096\C,0,-1.785072,1.099571,-0.071795\O,0,-1.305728,2.239586,-0.040549\C,0,2.70812,0.526124,0.183169\C,0,1.310654,0.955845,0.566958\H,0,0.983401,0.474566,1.503375\O,0,1.272709,2.356864,0.641889\H,0,0.339563,2.610274,0.447101\H,0,1.368203,-2.266218,1.04871\O,0,3.522976,1.501923,-0.303832\H,0,3.104162,2.344974,-0.022623\H,0,-3.083371,-2.548668,-0.228307\N,0,-4.883997,-0.588202,0.256789\H,0,-5.268051,-1.504909,0.424093\H,0,-5.424348,0.216937,0.540634\\Version=AM64L-G03RevD.01\State=2-A\HF=-885.7281643\MP2=-888.9430036\RMSD=1.688e-09\Thermal=0.\PG=C01 [X(C9H12N3O5)]\\@

022

1\1\GINC-NODE25\SP\ROMP2-FC\6-311+G(3df,2p)\C9H12N3O5(2)\ZIP06\15-Mar-2013\0\\#p ROMP2 (FC)/6-311+G(3df,2p) scf=tight\\Rad3Cy_022\\0,2\O,0,-2.201766,-2.370892,0.019317\C,0,-2.595467,-1.587027,1.139813\H,0,-2.087059,-1.912088,2.058112\H,0,-3.668275,-1.765081,1.268313\C,0,-2.358193,-0.075365,0.971027\H,0,-3.075049,0.443219,1.63069\O,0,-1.037855,0.294767,1.442371\C,0,-0.385194,1.160455,0.539183\H,0,-0.235841,2.139183,1.008778\N,0,0.970405,0.648575,0.261317\C,0,2.053776,1.351371,0.689325\H,0,1.843844,2.298066,1.176419\C,0,3.32043,0.891771,0.510336\C,0,3.444194,-0.384004,-0.129575\N,0,2.403366,-1.089408,-0.542995\C,0,1.139125,-0.619694,-0.37596\O,0,0.134143,-1.217546,-0.767123\C,0,-2.419046,0.427712,-0.445532\C,0,-1.284638,1.362372,-0.704519\H,0,-0.772798,1.098169,-1.637168\O,0,-1.631253,2.762015,-0.700996\H,0,-2.402074,2.855949,-1.283337\H,0,-1.262004,-2.157642,-0.18244\O,0,-2.969169,-0.290262,-1.45245\H,0,-2.91914,-1.244277,-1.186069\H,0,4.177766,1.456089,0.856332\N,0,4.672751,-0.936279,-0.308939\H,0,5.509702,-0.384893,-0.201826\H,0,4.722565,-1.777107,-0.86703\\Version=AM64L-G03RevD.01\State=2-A\HF=-885.7240426\MP2=-888.9425999 \RMSD=4.219e-09\Thermal=0.\PG=C01 [X(C9H12N3O5)]\\@

001

1\1\GINC-NODE19\SP\ROMP2-FC\6-311+G(3df,2p)\C9H12N3O5(2)\ZIP06\15-Mar-2013\0\\#p ROMP2 (FC)/6-311+G(3df,2p) scf=tight\\Rad3Cy_001\\0,2\O,0,-1.672205,2.193357,1.159434\C,0,-2.658714,2.166096,0.133357\H,0,-2.498855,2.982234,-0.588878\H,0,-3.67538,2.262861,0.54193\C,0,-2.567208,0.831757,-0.595251\H,0,-3.294364,0.825672,-1.416329\O,0,-1.224413,0.718612,-1.171314\C,0,-0.560146,-0.417503,-0.663344\H,0,-0.535049,-1.223465,-1.406515\N,0,0.844595,-0.070706,-0.370698\C,0,1.286954,1.211256,-0.333499\H,0,0.531703,1.968594,-0.493703\C,0,2.602053,1.4825,-0.10092\C,0,3.466239,0.356465,0.07368\N,0,3.037965,-0.897805,0.067514\C,0,1.717477,-1.162992,-0.097873\O,0,1.228919,-2.299651,-0.034215\C,0,-2.735647,-0.395935,0.255809\C,0,-1.355693,-0.90981,0.583407\H,0,-0.946565,-0.417168,1.47715\O,0,-1.395292,-2.311078,0.714058\H,0,-0.480275,-2.617723,0.514246\H,0,-1.862643,2.941332,1.744129\O,0,-3.624345,-1.328356,-0.191201\H,0,-3.247478,-2.191366,0.088432\H,0,2.964646,2.50251,-0.060199\N,0,4.797835,0.547846,0.292274\H,0,5.225768,1.433106,0.068545\H,0,5.382527,-0.276019,0.261268\\Version=AM64L-G03RevD.01\State=2-A\HF=-885.7300314\MP2=-888.9442329 \RMSD=9.791e-10\Thermal=0.\PG=C01 [X(C9H12N3O5)]\\@

011

1\1\GINC-NODE7\SP\ROMP2-FC\6-311+G(3df,2p)\C9H12N3O5(2)\ZIP06\15-Mar-2013\0\\#p ROMP2 (FC)/6-311+G(3df,2p) scf=tight\\Rad3Cy_011\\0,2\O,0,1.933503,3.140314,-0.189791\C,0,2.438679,1.937946,-0.750974\H,0,3.431713,2.081139,-1.20589\H,0,1.744527,1.676809,-1.555974\C,0,2.530683,0.785358,0.256178\H,0,3.326058,0.976966,0.986725\O,0,1.277115,0.698403,1.006104\C,0,0.557658,-0.458986,0.651071\H,0,0.520205,-1.16493,1.488827\N,0,-0.83834,-0.086934,0.337519\C,0,-1.251605,1.206245,0.276096\H,0,-0.481399,1.95395,0.42821\C,0,-2.559553,1.499093,0.031965\C,0,-3.448246,0.388223,-0.123029\N,0,-3.051368,-0.875887,-0.070951\C,0,-1.741149,-1.167509,0.125103\O,0,-1.284132,-2.319375,0.136009\C,0,2.693138,-0.560537,-0.384458\C,0,1.303312,-1.134366,-0.544083\H,0,0.831212,-0.802084,-1.482707\O,0,1.362401,-2.534492,-0.458506\H,0,0.437123,-2.811523,-0.263964\H,0,2.496681,3.382286,0.562616\O,0,3.630759,-1.396457,0.138519\H,0,3.27295,-2.301528,0.012003\H,0,-2.899267,

2.525749,-0.03172\N,0,-4.770031,0.604776,-0.366173\H,0,-5.178399,1.511171,-0.198337\H,0,-5.377907,-0.201622,-0.328446\\Version=AM64L-G03RevD.01\State=2-A\HF=-885.7286848\MP2=-888.942766 \RMSD=4.899e-09\Thermal=0.\PG=C01 [X(C9H12N3O5)]\\@

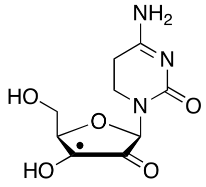

018

1\1\GINC-EVGENIX\SP\ROMP2-FC\6-311+G(3df,2p)\C9H12N3O5(2)\FLORIAN\03-Feb-2012\0\\#p ROMP2(FC)/6-311+G(3df,2p) scf=tight\\RadKet2dCy_018\\0,2\O,0,2.33028,2.225795,-0.381409\C,0,2.827076,1.670526,0.805335\H,0,2.463733,2.190513,1.709074\H,0,3.917959,1.779909,0.776326\C,0,2.505761,0.178401,1.003631\H,0,3.191116,-0.217979,1.772446\O,0,1.146921,0.014178,1.482964\C,0,0.417257,-0.967774,0.738135\H,0,0.297093,-1.869528,1.356002\N,0,-0.920866,-0.532485,0.415019\C,0,-2.027699,-0.952316,1.271401\H,0,-1.814223,-1.952948,1.656961\C,0,-3.310779,-0.962999,0.446154\H,0,-3.26835,-1.761994,-0.305786\C,0,-3.408293,0.369857,-0.262411\N,0,-2.377397,1.054421,-0.649525\C,0,-1.092186,0.552615,-0.441883\O,0,-0.12942,1.05598,-1.014392\C,0,2.537317,-0.649987,-0.231384\H,0,1.396476,1.95719,-0.495922\O,0,3.594208,-0.784512,-1.023838\H,0,3.297038,-1.362601,-1.760841\C,0,1.309869,-1.312567,-0.460943\O,0,1.071527,-2.088777,-1.39898\H,0,-4.183253,-1.143205,1.083482\H,0,-2.140307,-0.278339,2.134596\N,0,-4.645936,0.861726,-0.495959\H,0,-4.728181,1.704605,-1.048549\H,0,-5.477774,0.33289,-0.287504\\Version=AM64L-G03RevD.01\State=2-A\HF=-885.7422647\MP2=-888.9590225 \RMSD=3.885e-09\Thermal=0.\PG=C01 [X(C9H12N3O5)]\\@

034

1\1\GINC-BORIX\SP\ROMP2-FC\6-311+G(3df,2p)\C9H12N3O5(2)\FLORIAN\02-Feb-2012\0\\#P ROMP2(FC)/6-311+G(3df,2p) scf=tight\\RadKet2dCy_034\\0,2\O,0,2.42656,2.938012,0.281788\C,0,2.551032,1.733638,-0.455231\H,0,3.527169,1.759964,-0.950274\H,0,1.765679,1.628069,-1.213184 \C,0,2.492043,0.5265,0.493651\H,0,3.304666,0.629444,1.228939\O,0,1.23476,0.560574,1.219448\C,0,0.455226,-0.63624,1.034177\H,0,0.349226,-1.124296,2.011664\N,0,-0.883437,-0.3737,0.568705\C,0,-1.937953,-0.1623,1.556803\H,0,-1.750027,-0.812197,2.416337\C,0,-3.282267,-0.498152,0.918549\H,0,-3.346013,-1.576514,0.72163\C,0,-3.363481,0.244592,-0.397114\N,0,-2.333712,0.51839,-1.133895\C,0,-1.060151,0.097512,-0.738562\O,0,-0.118148,0.133424,-1.519575\C,0,2.484004,-0.807248,-0.153543 \H,0,1.57048,2.878531,0.736924\O,0,3.4621,-1.285592,-0.919672\H,0,3.144044,-2.165014,-1.222884 \C,0,1.295577,-1.526019,0.109967\O,0,1.049052,-2.675573,-0.282876\H,0,-4.109046,-0.225713,1.583508 \H,0,-1.944143,0.877037,1.920927\N,0,-4.589959,0.645265,-0.808339\H,0,-4.6724,1.04298,-1.734465\H,0,-5.428644,0.357565,-0.329494\\Version=AM64L-G03RevD.01\State=2-A\HF=-885.7402254\MP2=-888.9565121\RMSD=5.993e-09\Thermal=0.\PG=C01 [X(C9H12N3O5)]\\@

006

1\1\GINC-STEAK\SP\ROMP2-FC\6-311+G(3df,2p)\C9H12N3O5(2)\FLORIAN\03-Feb-2012\0\\#P ROMP2(FC)/6-311+G(3df,2p) scf=tight\\RadKet2dCy_006\\0,2\O,0,1.87365,2.421812,0.156467\C,0,2.911686,1.72417,0.817069\H,0,3.058279,2.102545,1.839814\H,0,3.834615,1.889509,0.253003\C,0,2.606184,0.215882,0.891692\H,0,3.302105,-0.249352,1.610986\O,0,1.248213,0.058394,1.343039\C,0,0.502037,-0.918191,0.548265\H,0,0.339855,-1.808622,1.161041\N,0,-0.790047,-0.423945,0.196022\C,0,-0.93234,0.494721,-0.932968\H,0,-0.020464,1.092556,-1.017054\C,0,-2.139418,1.397419,-0.693197\H,0,-1.935873,2.098241,0.127629\C,0,-3.304624,0.515473,-0.301679\N,0,-3.181119,-0.569141,0.393748\C,0,-1.916764,-0.980124,0.830404\O,0,-1.791835,-1.805171,1.721024\C,0,2.626357,-0.512879,-0.403837\H,0,1.067717,2.169969,0.63997\O,0,3.665179,-0.553053,-1.231583\H,0,3.376572,-1.114773,-1.985804\C,0,1.414103,-1.195888,-0.64781\O,0,1.177732,-1.875068,-1.663397\H,0,-2.363777,1.986875,-1.588577\H,0,-1.0541,-0.061287,-1.872966\N,0,-4.537642,0.895512,-0.716063\H,0,-5.333705,0.371456,-0.378517\H,0,-4.696398,1.787092,-1.157521\\Version=AM64L-G03RevD.01\State=2-A\HF=-885.7364614\MP2=-888.9555468 \RMSD=2.958e-09\Thermal=0.\PG=C01 [X(C9H12N3O5)]\\@

8. References

1. Zipse, H. Radical Stability—A Theoretical Perspective. *Top. Curr. Chem.* **2006**, *263*, 163–189.
2. Coote, M.L. Reliable Theoretical Procedures for the Calculation of Electronic-Structure Information in Hydrogen Abstarction Reactions. *J. Phys. Chem. A* **2004**, *108*, 3865–3872.
3. Scott, A.P.; Radom, L. Harmonic Vibrational Frequencies: An Evaluation of Hartree-Fock, Møller-Plesser, Quadratic Configuration Interaction, Density Functional Theory, and Semiempirical Scale Factors. *J. Phys. Chem.* **1996**, *100*, 16502–16513.
4. *MacroModel 9.7*; Schrödinger, LLC: New York, NY, USA, 2009.
5. Mohamadi, F.; Richard, N.G.J.; Guida, W.C.; Liskamp, R.; Lipton, M.; Caufield,C.; Chang, G.; Hendrickson, T.; Still, W.C. Macromodel—An integrated software system for modeling organic and bioorganic molecules using molecular mechanics. *J. Comput. Chem.* **1990**, *11*, 440–467.
6. Henry, D.J.; Parkinson, C.J.; Radom, L. An Assesment of the Performance of High-Level Theoretical Procedures in the Computation of the Heats of Formation of Small Open-Shell Molecules. *J. Phys. Chem. A* **2002**, *106*, 7927–7936.
7. Henry, D.J.; Sullivan, M.B.; Radom, L. G3-RAD and G3X-RAD_ Modified Gaussian-3 (G3) and Gaussian-3X (G3X) procedure for radical thermochemistry. *J. Chem. Phys.* **2003**, *118*, 4849–4860.
8. Tomasi, J.; Mennucci, B.; Cammi, R. Quantum Mechanical Continuum Solvation Models *Chem. Rev.* **2005**, *105*, 2999–3094.
9. Cossi, M.; Rega, N.; Scalmani, G.; Barone, V. Energies, structures, and electronic properties of molecules in solution with the C-PCM solvation model. *J. Comput. Chem.* **2003**, *24*, 669–681.
10. Marenich, A.V.; Cramer, C.J.; Truhlar, D.G. Universal Solvation Model Based on Solute Electron Density and on a Continuum Model of the Solvent Defined by the Bulk Dielectric Constant and Atomic Surface Tensions. *J. Phys. Chem. B* **2009**, *113*, 6378–6396.
11. Barone, V.; Cossi, M.; Tomasi, J. A new definition of cavities for the computation of solvation free energies by the polarizable continuum model. *J. Chem. Phys.* **1997**, *107*, 3210–3221.

12. MOLPRO Version 2010.1. A Package of *ab initio* Programs. Available online: http://www.molpro.net/info/authors (accessed on 15 December 2014).
13. Gaussian03 Rev. D.01. Available online: http://www.Gaussian.com/g_misc/g03/citation_g03.htm (accessed on 15 December 2014).
14. Gaussian09 Rev. C.01. Available online: http://www.Gaussian.com/g_tech/g_ur/m_citation.htm (accessed on 15 December 2014).
15. Luo, Y.R. *Comprehensive Handbook of Chemical Bond Energies*; CRC Press: Boca Raton, FL, USA, 2007.
16. Lide, D.R. *CRC Handbook of Chemistry and Physics* 89th ed.; CRC Press: Boca Raton, FL, USA, 2008.
17. Hioe, J.; Zipse, H. Radical Stability—Thermochemical Aspects. In *Encyclopedia of Radicals in Chemistry, Biology and Materials*; Chatgilialoglu, C., Studer, A., Eds.; John Wiley & Sons Ltd.: Chichester, UK, 2012.
18. Bhattacharya, A.; Shivalkar, S. Re-tooling Benson's Group Additivity Method for Estimation of the Enthalpy of Formation of Free Radicals: C/H and C/H/O Groups. *J. Chem. Eng. Data* **2006**, *51*, 1169–1181.
19. Cohen, N. Revised Group Additivity values for Enthalpies of Formation (at 298 K) of Carbon-Hydrogen and Carbon-Hydrogen-Oxygen Compounds. *J. Phys. Chem. Ref. Data* **1996**, *25*, 1411–1479.
20. Berkowitz, J.; Ellison, G.B.; Gutman, D. Three methods to measure RH bond energies. *J. Phys. Chem.* **1994**, *98*, 2744–2765.
21. Tsang, W. Heats of Formation of Organic Free Radicals. In *Energetics of Organic Free Radicals*; Martinho Simoes, J.A., Greenberg, A., Liebman, J.F., Eds.; Blackie Academic and Professional: New York, NY, USA, 1996.
22. Holmes, F.J.L.; Lossing, F.P.; Malcolm, A. Heats of formation of alkyl radicals from appearance energies. *J. Am. Chem. Soc.* **1988**, *110*, 7339–7342.
23. Manion, J.A. Evaluated Enthalpies of Formation of the stable Closed Shell C1 and C2 clorinated Hydrocarbons. *J. Phys. Chem. Ref. Data* **2002**, *31*, 123–172.
24. Holmes, J.; Lossing, F.P.; Mayer, P.M. Heats of formation of oxygen-containing organic free radicals from appearance energy measurements. *J. Am. Chem. Soc.* **1991**, *113*, 9723–9728.
25. Smith, D.M.; Buckel, W.; Zipse, H. Deprotonation of Enoxy Radicals: Theoretical Validation of a 50-Year-Old Mechanistic Proposal. *Angew. Chem. Int. Ed.* **2003**, *42*, 1867–1870.
26. Baboul, A.G.; Curtiss, L.A.; Redfern, P.C.; Raghavachari, K. Gaussian-3 theory using density functional geometries and zero-point energies. *J. Chem. Phys*. **1999**, *110*, 7650–7657.
27. Sun, H.; Bozelli, J.W. Structures, Rotational Barriers, Thermochemical Properties, and Additivity Groups for 2-Propanol, 2-chloro-2-propanol and the Corresponding Alkoxy and Hydroxyalkyl Radicals. *J. Phys. Chem. A* **2002**, *106*, 3947–3956.
28. Cord, M.; Husson, B.; Huerta, J.C.L.; Herbinet, O.; Glaude, P.-A.; Fournet, R.; Sirjean, B.; Battin-Leclerc, F.; Ruiz-Lopez, M.; Wang, Z.; *et al.* Study of the Low Temperature Oxidation of Propane. *J. Phys. Chem. A* **2012**, *116*, 12214–12228.
29. Wood, G.P.F.; Radom, L.; Petersson, G.A.; Barnes, E.C.; Frisch, M.J.; Montgomery, J.A., Jr. A restricted-open-shell complete-basis-set model chemistry. *J. Chem. Phys.* **2006**, *125*, doi:10.1063/1.2335438.
